# Triplet Energy Transfer-Mediated Intermolecular Paternò–Büchi Reaction for the Synthesis of Trifluoromethylated Oxetanes

**DOI:** 10.1021/acs.orglett.6c00113

**Published:** 2026-03-05

**Authors:** Yining Zhu, Anthony J. Fernandes, Egor Zhilin, Dmitry Katayev

**Affiliations:** Department of Chemistry, Biochemistry and Pharmaceutical Sciences, 27210 University of Bern, Freiestrasse 3, 3012 Bern, Switzerland

## Abstract

Oxetanes are increasingly used as compact, polar carbonyl (bio)­isosteres in drug discovery, and the combination with a trifluoromethyl group enables fine-tuning of molecular properties. Here, we report a visible-light-mediated Paternò–Büchi strategy that accesses CF_3_-containing oxetanes via triplet-energy-transfer activation and delivers a broad range of CF_3_-containing oxetane products with excellent regio- and diastereoselectivity. Mechanistic studies indicate that although sensitization of both the alkene and carbonyl occurs in solution, the subsequent stepwise [2 + 2] cycloaddition proceeds preferentially from the triplet excited state of the CF_3_-substituted carbonyl partner.

## Graphical abstract

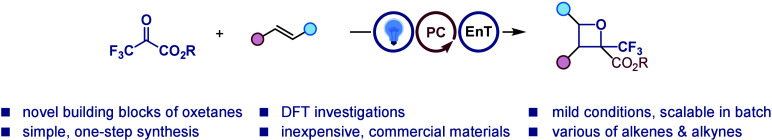

Oxetanes have emerged as compact, polar, and conformationally well-defined motifs that serve as (bio)­isosteres of both *gem*-dimethyl groups and carbonyl derivatives in medicinal chemistry.^1^ Unlike flat carbonyls, the oxetane ring deviates from planarity, enhancing three-dimensionality, conformational rigidity, and vector diversity within small-molecule scaffolds (Figure 1A).^2^ Incorporation of an oxetane typically reduces lipophilicity while maintaining membrane permeability, improves aqueous solubility, and minimizes nonspecific binding.^3^ Consequently, oxetane-containing scaffolds have become increasingly prevalent in drug discovery,^4^ accompanied by a marked rise in patent filings and in commercially available oxetane building blocks that facilitate rapid analogue synthesis.

**Figure 1:**
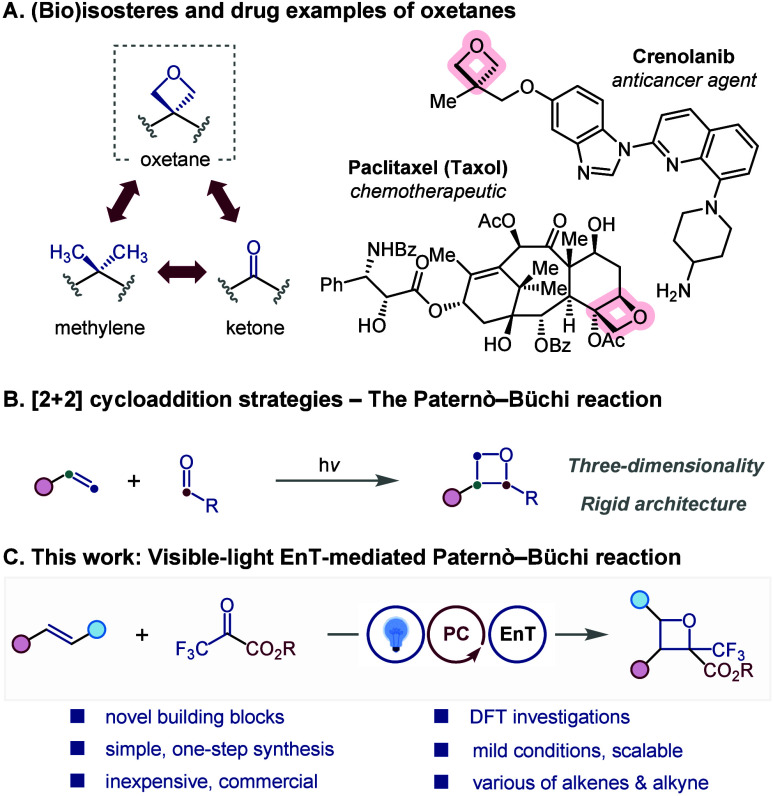
(A) (Bio)­isosteres and drug examples. (B) The Paternò–Büchi reaction. (C) Our approach.

To access oxetane motifs, multiple synthetic strategies have been developed, including ring expansion of epoxides, intramolecular nucleophilic substitution,^5^ and ring contraction of lactone intermediates.^6^ However, these methods often rely on multistep preparation of complex starting materials, limiting their practicality. Meanwhile, the Paternò–Büchi reaction,^7^ known as a photochemical [2 + 2] cycloaddition between carbonyl compounds and alkenes, offers a direct, atom-economical route to oxetane frameworks in a single step.^8^

Traditionally, Paternò–Büchi reactions proceed under ultraviolet irradiation via an n,π* state, yielding carbonyl-based diradical intermediates. This enables direct cycloaddition between excited carbonyls and ground-state alkenes (Figure 1B). However, such processes remain limited by harsh reaction conditions, a narrow substrate scope, and the intrinsic reactivity of carbonyl triplet states (e.g., Norrish type I and type II pathways).^9^ On the other hand, direct excitation of substituted alkenes requires enhanced UV light energy to achieve the related π,π* transition.

In recent years, photocatalytic Dexter energy transfer (EnT) has emerged as a powerful platform for generating diradicals, unlocking access to diverse ring systems under mild conditions.^10^ This general approach has enabled efficient synthesis of oxetanes,^11^ cyclobutanes,^11e,11f,12^ azetidines,^13^ and other ring systems, as elegantly demonstrated by Schindler,^14^ Glorius,^15^ Yoon,^16^ You,^17^ Brown,^18^ Bach,^19^ and others.^20^

Inspired by these contributions, and motivated by our longstanding interest in the introduction of fluorinated motifs for drug discovery,^21^ we herein report a visible-light-mediated photocatalytic approach for synthesizing trifluoromethyl-substituted oxetanes through an intermolecular [2 + 2] cycloaddition using pyruvate **2** as the carbonyl coupling partner (Figure 1C).

We initially assessed the feasibility of the [2 + 2] cycloaddition between *p*-*tert*-butylstyrene **1** and methyl trifluoropyruvate **2** under blue-light irradiation. Gratifyingly, in the presence of Ir­[dF­(CF_3_)­ppy]_2_dtbbpyPF_6_ ([Ir–F]) as the photocatalyst and dimethyl carbonate (DMC) as the solvent, the cycloaddition reaction proceeded smoothly to afford a mixture of cycloaddition products in 71% yield as determined by ^19^F NMR analysis (Table 1, entry 1). NMR analysis revealed the formation of four isomeric oxetane products **3a**, **3a′**, **3b**, and **3b′** in the ratio of 5:5:1.5:1, unambiguously assigned by NOE-assisted ^1^H–^19^F NMR spectroscopy (see Table 1 and SI).

**Table 1:**
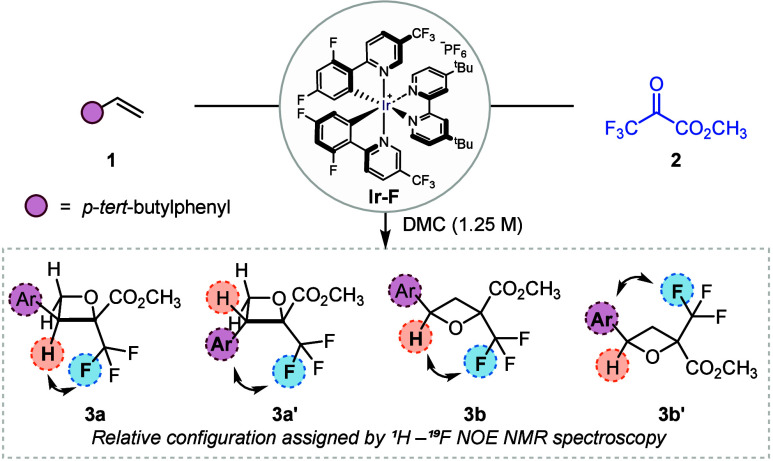
Screening of Reaction Conditions

**Entry** ^a^	**Deviation from above**	**Yield (%)** ^b^
**1** ^c^	**No deviation**	71 (70)
2	MeOH or DMF *instead of DMC*	
3	MeCN *instead of DMC*	16
4	EtOAc *instead of DMC*	58
5	4CzIPN *as PC*	56
6^d^	Thioxanthone *as PC*	42
7	Ir(ppy)_3_ *as PC*	trace
8	Ru(bpy)_3_(PF_6_)_2_ *as PC*	trace
9	With H_2_O (0.5 equiv)	30
10	Under air	57
11	With TEMPO (0.5 equiv)	
12	No PC	trace
13	No light	

a Reaction conditions: **1** (0.5 mmol, 1.0 equiv), **2** (0.75 mmol, 1.5 equiv), [Ir–F] (0.5 mol %), DMC (0.8 mL), blue LEDs, rt, 16 h.

b Combined yield of isomers determined by ^19^F NMR with PhCF_3_ as an external standard.

c Isolated yield of compounds given in parentheses.

d Reaction was irradiated at 390 nm.

With the product structures identified, we continued to examine the reaction conditions. Polar solvents such as methanol and DMF showed no reactivity under the standard conditions (entry 2). Switching to acetonitrile afforded only marginal reactivity (entry 3). Employing ethyl acetate delivered a 58% yield (entry 4), with product selectivity comparable to that observed under the optimized conditions. The photocatalyst proved crucial for accessing the alkene’s triplet state, as substitution of [Ir–F] with 4CzIPN or thioxanthone (*E*(T_1_) = 61.8, 58.3, and 63.4 kcal·mol^–1^, respectively) resulted in only moderate yields (entries 5 and 6).^10b^ However, when a photocatalyst with high reducing ability, such as Ir­(ppy)_3_ (E­[*Ir^III^/Ir^IV^] = −1.73 V vs SCE), was tested, the [2 + 2] cycloaddition was suppressed entirely (entry 7), likely due to the reduction of methyl trifluoropyruvate (*E*_p/2_ = −0.70 V vs SCE, see SI) in this case. As an additional control, Ru­(bpy)_3_(PF_6_)_2_, which possesses similar redox potentials (*E*[*Ru^II^/Ru^III^]: −0.81 V; Ru^3+^/Ru^2+^: +1.29 V vs SCE) as [Ir–F] but a low *E*(T_1_) of 48.4 kcal·mol^–1^, led to no reactivity (entry 8).^22^ This contrast further supports that triplet–triplet energy transfer, rather than a redox pathway, is operative in this transformation. To evaluate the oxygen and moisture tolerance of the reaction, 0.5 equiv of water was introduced into the reaction mixture, resulting in a significant decrease in yield (entry 9). This effect is likely due to hydration of the carbonyl group, which subsequently inhibits the [2 + 2] process. In the meantime, performing the reaction under air showed that the procedure is slightly affected by atmospheric oxygen (entry 10). However, in the presence of radical inhibitors such as TEMPO, the reaction was inhibited entirely (entry 11). Finally, control experiments confirmed the essential roles of both the photocatalyst and visible light (entries 12 and 13).

With the optimal conditions in hand, we proceeded to explore the substrate scope. For terminal alkenes, most substrates delivered four distinct isomers, consistent with the outcome observed for the model substrate (Figure 2). In most cases, the a-type products were obtained in higher amounts than the b-type products. This selectivity likely originates from the greater stability of the benzylic-based diradical intermediate (Figure 3A, **4**), whereas the b-type intermediate lacks comparable stabilization (Figure 3D, **int-1′**). The optimized conditions proved effective for a range of aromatic substrates, delivering the corresponding oxetanes in moderate to good yields (**4** and **6**). 2,4-Dimethylstyrene also furnished the desired product in a good yield (**7**). When a *para*-electron-donating group was introduced, the reaction afforded oxetanes **5** and **8** in 32% and 48% yields, respectively, both with excellent regioselectivity. Furthermore, when the phenolic oxygen was protected with an acyl group, the b-type oxetane reappeared, giving a product distribution comparable to that of electron-neutral styrenes with an overall yield of 51%. Subsequent investigations focused on β-substituted alkenes were carried out and various substituents on β-methylstyrene were first evaluated. The unsubstituted substrate furnished the corresponding oxetanes in a 78% yield (**11**). The *para*-methoxy derivative afforded the product in 42% yield (**12a**), comparable to **5a**, with excellent regioselectivity and diastereoselectivity. The *para*-bromo substrate provided the oxetane in 72% yield, also with outstanding selectivity (**13a**). The reaction further demonstrated compatibility with phthalimide-masked amines (**14a**), furan derivatives (**15a**), acetal (**16a**), and benzoyl-protected phenols (**17a**) in moderate to good yields. A cyclic olefin, such as indene, performed moderately well, yielding product **18a** in 45% yield. For known triplet quenchers such as cyclic 1,3-dienes (*E*(T_1_) ∼ 53 kcal·mol^–1^),^23^ the corresponding oxetane (**19a**) was obtained in 60% yield with excellent stereo- and regioselectivity.

**Figure 2:**
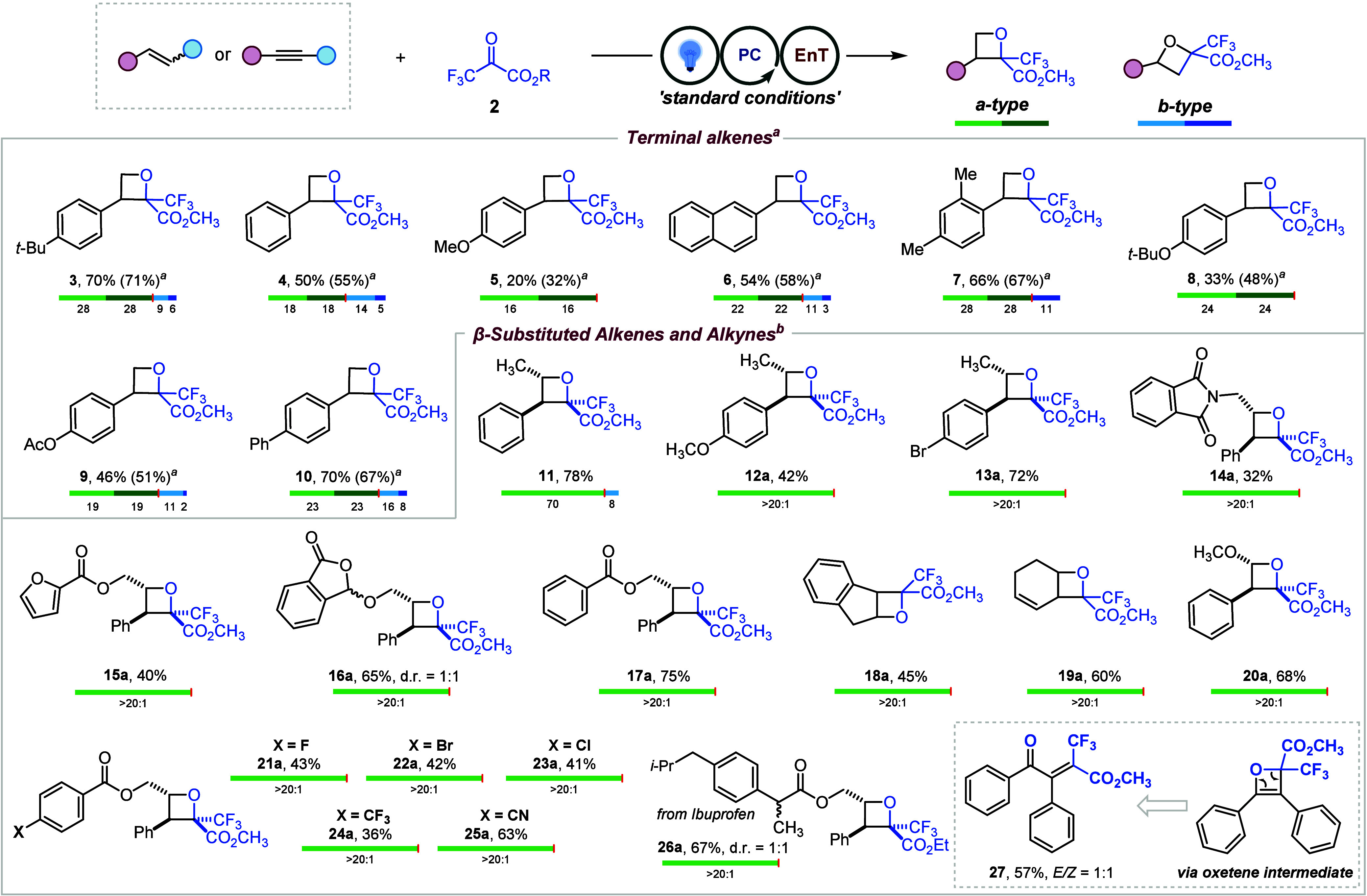
Substrate scope. General reaction conditions: substrate (1.0 equiv), **2** (1.5 equiv), [Ir–F] (0.5 mol %), DMC (1.25 M), blue LEDs, rt, 16 h. Yields of the isolated compounds are shown. ^*a*^Yields and ratios of isomers were determined by ^19^F NMR with PhCF_3_ as an external standard.

**Figure 3:**
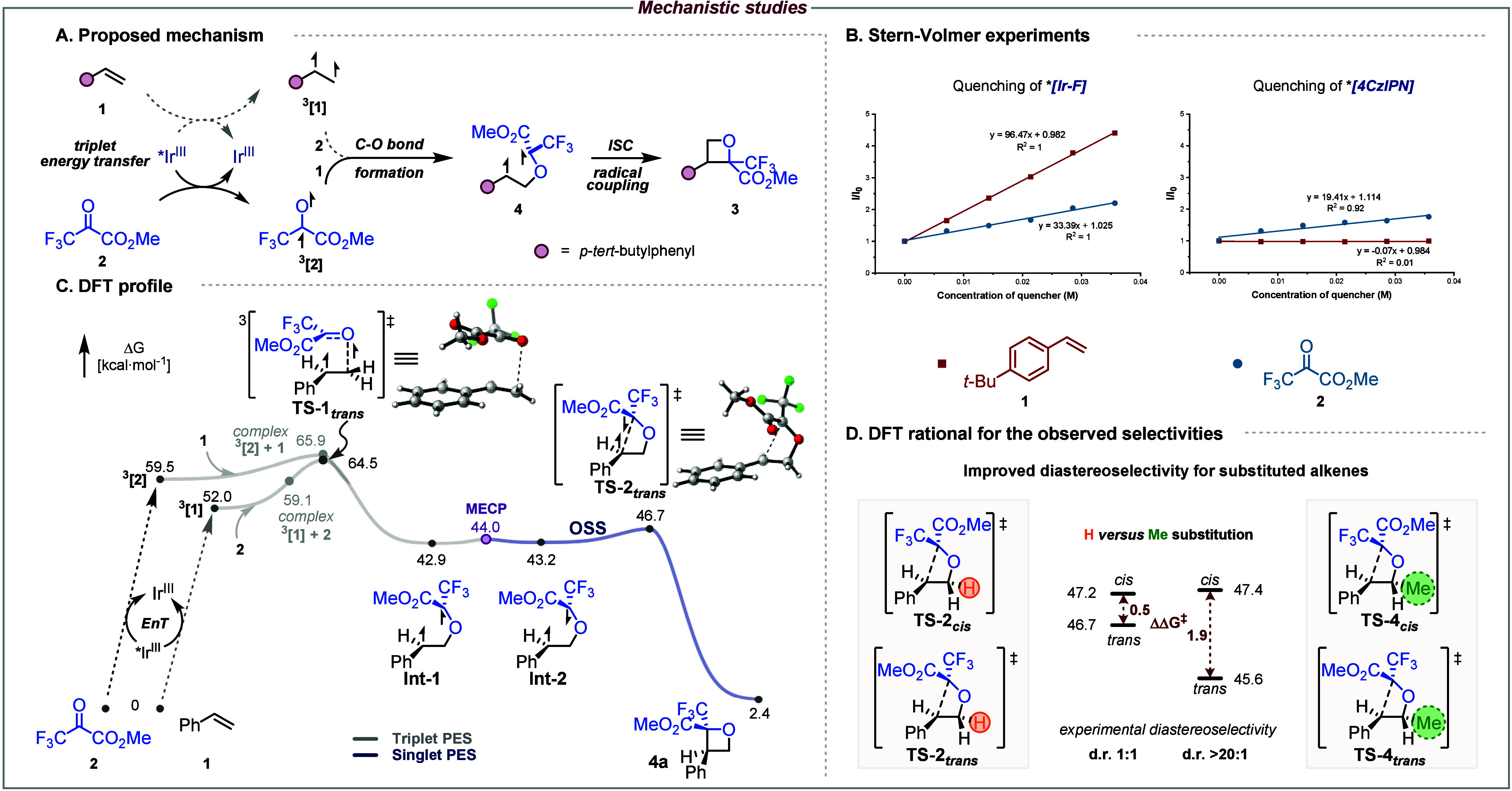
Mechanistic studies. (A) Plausible mechanism. (B) Spectroscopic evidence. (C) DFT profile computed at the (U)­ωB97X-D/def2TZVPP,SMD­(EtOAc)//(U)­ωB97X-D/def2SVP,SMD­(EtOAc) level of theory.^26^

β-Methoxystyrene also reacted smoothly to afford **20a**. Cinnamyl alcohol–derived alkenes bearing a variety of functional groups were well tolerated under the standard conditions. Halogens (**21a**, **22a**, and **23a**), trifluoromethyl (**24a**), and cyano substituents (**25a**) were all compatible, highlighting the robustness of the method. An ibuprofen-derived substrate underwent a smooth transformation to deliver **26a**. Finally, when diphenylacetylene was subjected to the standard conditions, the reaction furnished ring-opened product **27** in a 1:1 *E*/*Z* mixture, presumably arising from a highly strained oxetene intermediate. In the case of tri- or tetrasubstituted alkenes, the reaction was found to proceed with low conversion.

According to our mechanistic hypothesis and reports in the literature,^11c,24^ the [Ir–F] photosensitizer is first excited to its triplet state under blue-light irradiation. A subsequent energy transfer would then promote one substrate to its triplet state, which then reacts with the other partner to form an intramolecular 1,4-diradical intermediate.^16d,25^ The latter finally undergoes intersystem crossing (ISC) followed by radical–radical recombination to furnish the oxetane product (Figure 3A). Initial ^1^H NMR studies showed that when *cis*- and *trans*-alkenes were independently subjected to the photocatalytic [2 + 2] conditions, ^1^H NMR spectra after 2 h of irradiation produced the same isomeric *E*/*Z* mixture, and both reactions converged to the formation of the same oxetane **22a** (see SI). These observations provide compelling evidence for the generation of an alkene triplet diradical. Stern–Volmer quenching experiments were also carried out. In these studies, the quenching profiles clearly showed that *p*-*tert*-butylstyrene **1** quenches the excited state of [Ir–F] more efficiently than methyl trifluoropyruvate **2**, suggesting that both pathways might be competing. Interestingly, when 4CzIPN was used, only **2** proved to quench its luminescence, which was found to be less efficient than with [Ir–F]. Collectively, these results support an energy transfer-mediated mechanism.

The reaction was then investigated using DFT calculations to shine light on both activation pathways (see SI for further details). At the onset of our studies, calculations confirmed that the triplet-state electronic energy of styrene **1** is lower than that of methyl trifluoropyruvate **2**, with *E*(T_1_) values of 56.2 and 62.0 kcal·mol^–1^, respectively (see Table S8). Calculation of the reaction between triplet pyruvate ^**3**^ **[2]** and ground state **1** was first explored (Figure 3C, see SI for details), and the reaction was found to proceed barrierlessly on the electronic potential energy surface. This suggests that the reactant complex (Δ*G* = 65.9 kcal·mol^–1^) readily collapses to form the well-stabilized 1,4-diradical intermediate **Int-1** (Δ_r_ *G* = −16.6 kcal·mol^–1^), exhibiting both a benzylic and a captodative radical. On the other hand, the reaction between the triplet styrene ^**3**^ **[1]** and ground state **2** exhibits a barrier of Δ*G* ^‡^ = 12.5 kcal·mol^–1^ for transition state **TS-1**_***trans***_ involving an exergonic C–O bond formation leading to the same **Int-1** (Δ_r_ *G* = −9.1 kcal·mol^–1^), en route to product **4a**. A transition state, **TS-1-CC**_***trans***_, involving the first C–C bond formation, could also be located but was found to be noncompetitive (Δ*G* ^‡^ = 18.1 kcal·mol^–1^, see Figure S8). Consequently, these calculations support that the most productive pathway involves the triplet carbonyl ^**3**^ **[2]** species, despite the latter being excited less effectively by [Ir–F]; however, cycloaddition arising from styrene sensitization cannot be ruled out. From triplet diradical **Int-1**, a low-lying MECP (Δ*E* = 1.1 kcal·mol^–1^ above **Int-1**, see Figures S12 and S13) yields the open-shell singlet (OSS) diradical **Int-2** (Δ_r_ *G* = 0.3 kcal·mol^–1^), which undergoes rapid recombination through **TS-2**_***trans***_ with a very small overall barrier (Δ*G* ^‡^ = 3.8 kcal·mol^–1^). This radical–radical coupling furnishes oxetane **4a** (Δ_r_ *G* = −40.5 kcal·mol^–1^).

Finally, to better understand the strong influence of β-substitution of styrene on the diastereoselectivity of the reaction (Figure 3D, bottom), we investigated the radical recombination event in greater detail, as it represents the stereodetermining step. For unsubstituted styrene, we located **TS-2**_***cis***_ lying only 0.5 kcal·mol^–1^ higher than the diastereomeric **TS-2**_***trans***_ (ΔG^‡^ = 4.3 kcal·mol^–1^, from **Int-1**). In contrast, for β-methylstyrene, **TS-4**_***trans***_ and **TS-4**_***cis***_ were found to have barriers of 2.5 and 4.4 kcal·mol^–1^ from **Int-3**, respectively. This larger energy gap of 1.9 kcal·mol^–1^ indicates improved selectivity, in excellent agreement with the experimentally observed diastereoselectivity. Overall, DFT studies support a stepwise photosensitized cycloaddition mechanism, involving an initial C–O bond-forming step, followed by a stereodetermining radical recombination (C–C-forming) event.

To demonstrate the protocol’s operational simplicity and scalability, the reaction was carried out on a 10.0 mmol scale under standard batch conditions, and the corresponding product **14a** was isolated in 65% yield (Figure 4A). Given the importance of fluorine-containing building blocks, the synthetic utility of **14a** was further explored through diversification, with the ester and bromo groups serving as practical coupling handles. Chemoselective reduction of the former with NaBH_4_ affords the corresponding alcohol **28** in 61% yield, while Suzuki–Miyaura cross-coupling of the latter furnishes arylated derivatives **29** in 55% yield, thereby enabling modular modification of diverse pharmaceutical scaffolds (Figure 4B).

**Figure 4:**
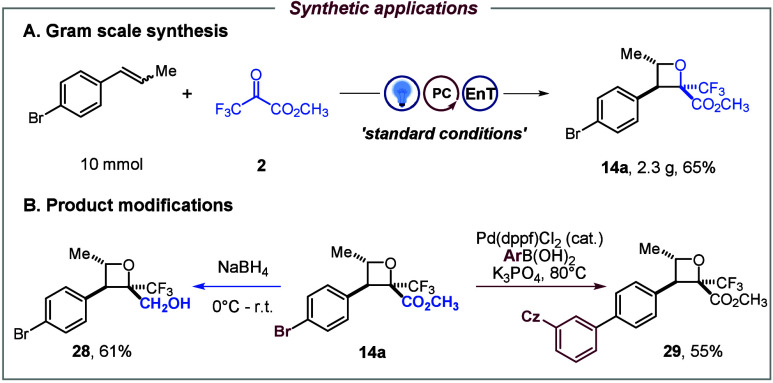
Synthetic applications. (A) Gram-scale synthesis, standard conditions: alkene (10 mmol, 1.0 equiv), [Ir–F] (0.5 mol %), methyl trifluoropyruvate (15.0 mol, 1.5 equiv), DMC (1.0 M), rt, 24 h, N_2_. (B) Product modifications.

In summary, we have developed a general and efficient visible-light-driven methodology for the direct synthesis of a broad range of CF_3_-substituted oxetanes. This transformation proceeds via a triplet energy-transfer manifold, thereby enabling Patern-Büchi reactions with excellent regio- and diastereoselectivity. The protocol accommodates diverse alkene coupling partners and provides straightforward access to CF_3_-containing oxetanes from readily available substrates. We anticipate that this strategy will not only expand the toolkit for constructing oxetane-based (bio)­isosteres but also facilitate streamlined late-stage diversification and accelerate applications in drug discovery.

## Supplementary Material



## Data Availability

The data underlying this study are available in the published article and its Supporting Information.

## Notes

All data supporting the findings of this study are available within the article, including experimental and computational methods, experimental characterization data and NMR spectra of new compounds, energies of optimized structures and Cartesian coordinates (PDF)
