# Supplementary material for: Triplet Energy Transfer-Mediated Intermolecular Paternò–Büchi Reaction for the Synthesis of Trifluoromethylated Oxetanes
Source: Org Lett. 2026 Mar 5;28(11):3388–93. doi: 10.1021/acs.orglett.6c00113 (PMC13010325; doi:10.1021/acs.orglett.6c00113)
Supplement: Supplementary file 1 [file ol6c00113_si_001.pdf]

Supporting Information

Triplet Energy Transfer-Mediated Intermolecular Paternò-Büchi Reaction for the Synthesis of Trifluoromethylated Oxetanes

Yining Zhu, Anthony J. Fernandes, Egor Zhilin, and Dmitry Katayev*

University of Bern, Department of Chemistry, Biochemistry, and Pharmaceutical Sciences, Freiestrasse 3, 3012 Bern

* Corresponding author

Email: dmitry.katayev@unibe.ch

Table of contents

1. Materials and methods --- 3

1.1. High-intensity photoreactor --- 4

2. Development of the reaction conditions --- 5

2.1. Solvent effect --- 5

2.2. Photocatalyst effect --- 6

2.3. Loading of reagents --- 7

2.4. Loading of photocatalyst --- 7

2.5. Concentration effect --- 8

2.6. Control experiment --- 8

3. Availability of starting materials --- 9

3.1. Commercially available starting materials --- 9

3.2. Prepared starting materials --- 9

4. General procedures --- 9

5. Synthesis of olefins --- 10

6. Mechanistic Studies --- 15

6.1. Stern-Volmer quenching studies --- 15

6.2. Cyclic voltammetry --- 19

6.3. Time-resolved ^{1}H NMR experiments --- 20

7. Computational details --- 21

7.1. Computational methods --- 21

7.2. Mechanistic studies --- 22

7.3. Computed energies --- 27

7.4. Computed geometries (xyz coordinates) --- 28

8. NMR description --- 34

9. Scale-up synthesis and product transformations --- 49

10. NMR Spectra --- 52

10.1. 2D NMR Studies and Stereochemical Assignment --- 52

10.2. Copies of NMR spectra --- 58

11. References --- 220

1. Materials and methods

All reactions were performed in flame-dried glassware under an argon atmosphere, using a teflon-coated stirring bar and dry septum. In addition, glassware was dried overnight at 120 °C before use. Starting materials are commercially available and were purchased from Thermoscientific – Acros, Sigma-Aldrich, Apollo Scientific, Fluorochem, TCI, and Chemie Brunschwig AG, unless otherwise noted. Analytical thin-layer chromatography (TLC) was performed on Merck silica gel 60 F254 TLC glass plates and visualized with 254 nm light and potassium permanganate staining solutions, followed by heating when required. Purification of reaction products was carried out by flash chromatography using Brunschwig silica (32-63 µm, 60Å) under 0.3-0.5 bar overpressure. Medium pressure liquid chromatography (MPLC) was performed on a CombiFlash Rf 200 System from Teledyne ISCO with a built-in UV-detector and fraction collector or manually using silica gel SilicaFlash P60, 40-63 µm. Teledyne ISCO RediSep Rf flash columns used have 0.035–0.070 mm particle size and 230–400 mesh. Normal-phase preparatory HPLC purification was performed on a Teledyne Isco CombiFlash EZ Prep system using a Macherey-Nagel VP 250/21 Nucleosil 50-5 column. ^{1}H- and ^{13}C-NMR spectra were recorded on Bruker Ultrashield 300 (operating at 300.1 MHz and 75.5 MHz, respectively), Bruker Ascend 400 (operating at 400.1 MHz and 100.6 MHz, respectively), Bruker AVANCE III 500 (operating at 500.1 MHz and 125.6 MHz, respectively), ^{19}F-NMR spectra on Bruker DPX-300 and Bruk er Ultrashield 300 (at 282 MHz) and Bruker DPX-400 and Bruker Ascend 400 (at 376 MHz) Bruker DPX-500 and Bruker AVANCE III 500 (at 477 MHz). The chemical shifts are reported in parts per million (ppm), and coupling constants (J) are given in Hertz (Hz). 1H-NMR spectra are reported with the solvent resonance as the reference unless noted otherwise ($CDCl_3$ at 7.26 ppm). Peaks are reported as (s = singlet, d = doublet, t = triplet, q = quartet, m = multiplet or unresolved, coupling constant(s) in Hz, integration). ^{13}C-NMR spectra were recorded with ^{1}H-decoupling and are reported with the solvent resonance as the reference unless noted otherwise ($CDCl_3$ at 77.16 ppm). ^{19}F-NMR spectra were recorded with ^{1}H-decoupling or coupled. A Bruker Tensor III spectrometer equipped with a golden gate was used to record infrared spectra HR-MS (ESI^{+}) mass spectra were measured on a Bruker FTMS 4.7T BioAPEX II and Thermo Scientific LTQ Orbitrap XL equipped with a static nanospray ion source and mass spectrometry service operated on VG-TRIBRID for electron impact ionization (EI), or Varian IonSpec Spectrometer for electrospray ionization (ESI) and are reported as (m/z,). Electron impact ionization mass spectra (EI-MS) were run on a gas chromatography – mass spectrometry (GC-MS) instrument of Agilent 8890 series GC system and Agilent 5977B GC/MSD. HRMS are reported for the major isomer unless otherwise stated. Fluorescence spectroscopy was measured using the FS5 Spectrofluorometer from Edinburgh Instruments. Cyclic voltammetry was measured using the Osilla potentiostat, an Ag^{+} (0.01M $AgNO_3$)/Ag reference electrode, a platinum disc working electrode, and a platinum counter electrode. All measurements were carried out in acetonitrile (0.1 M NBu_4PF_6) if not stated otherwise.

1.1. High-intensity photoreactor

The photoreactor (Figure S1) used in the present work is a modified version of the initial design conceived by Dr. B. Jelier in collaboration with the mechanical workshop of the Department of Chemistry and Applied Biosciences at ETH Zurich, as reported here: Jelier, B. J.; Tripet, P. F.; Pietrasiak, E.; Franzoni, I.; Jeschke, G.; Togni, A. Radical Trifluoromethoxylation of Arenes Triggered by a Visible-Light-Mediated N−O Bond Redox Fragmentation. *Angew. Chem., Int. Ed,* **2018**, *57*, 13784-13789.

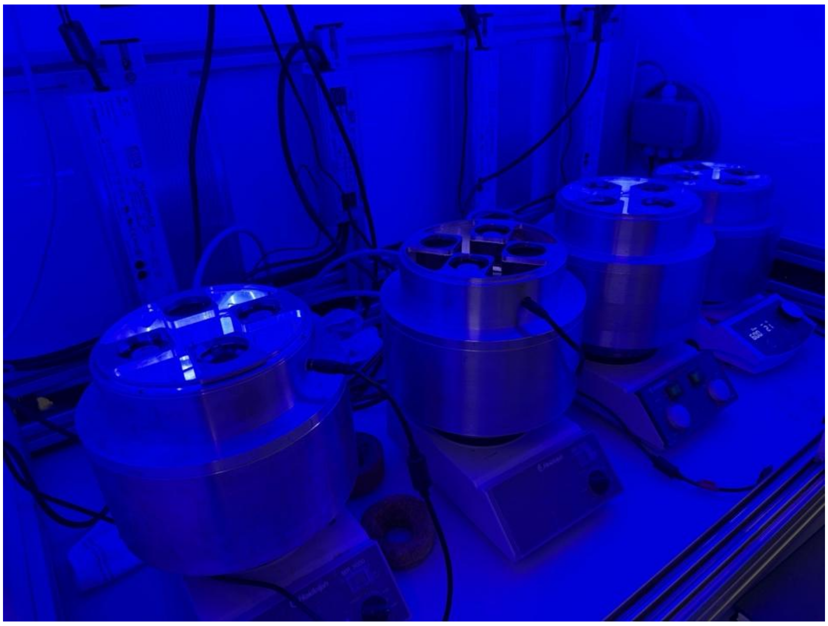

Figure S1. Custom high-intensity, blue LED photoreactors for photocatalytic reactions.

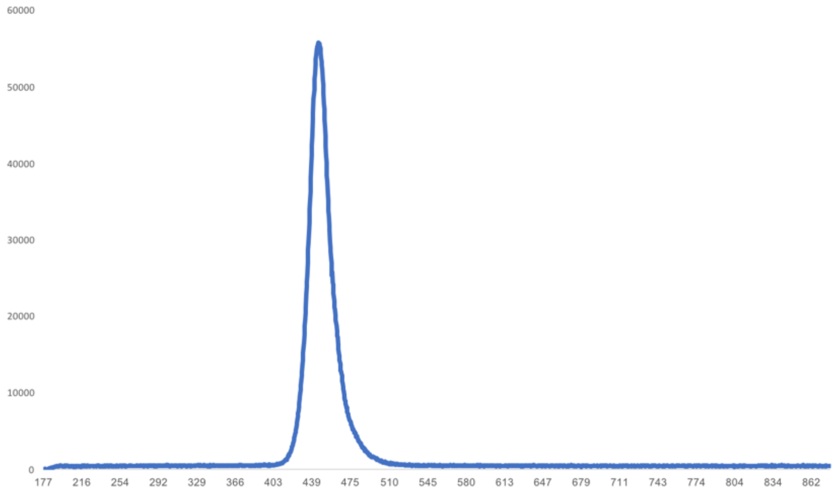

Figure S2. UV-Vis emission spectrum of high-intensity, blue LED photoreactor (λmax = 440 nm, FWHM = 20 nm).[1]

2. Development of the reaction conditions

Standard general conditions: A flame-dried 5 mL crimp cap vial was charged with photocatalyst (*x* mol%) and equipped with a magnetic stirring bar. The vial was then subjected to three vacuum/argon cycles. Dimethyl carbonate (*x* mL) was added under an argon atmosphere, and the yellow solution was degassed for 5 min. 4-*tert*-Butylstyrene (94%, stab. with 50 ppm 4-*tert*-butylcatechol, commercially available at Thermoscientific - Alfa Aesar, 95 µL, 0.5 mmol, 1.0 equiv) and methyl 3,3,3-trifluoro-2-oxopropanoate (*x* equiv) were introduced to the solution *via* microsyringes. The reaction mixture was irradiated under blue LEDs at ambient temperature for 16 h. An internal standard of trifluoromethylbenzene (61 µL, 0.5 mmol, 1.0 equiv) was added *via* a microsyringe once the reaction was completed. An aliquot was taken and analyzed by ^{19}F NMR to determine the yield.

2.1. Solvent effect

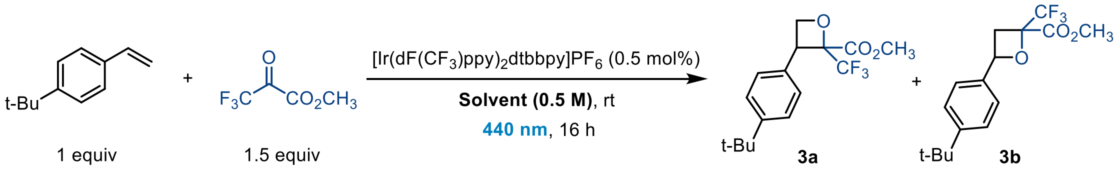

Entry[a]	Solvent	a [%]	a' [%]	b [%]	b' [%]	Yield [%][b]
1	1,2-DCE	9	9	2	3	23
2	CCl_4	12	15	n.d.	n.d.	27
3	$CHCl_3$	7	7	n.d.	n.d.	14
4	EtOAc	24	21	10	3	58
5	Dimethyl Carbonate	27	24	10	6	67
6	Isopropanol	n.d.	n.d.	n.d.	n.d.	n.d.
7	Ethanol	n.d.	n.d.	n.d.	n.d.	n.d.
8	Methanol	n.d.	n.d.	n.d.	n.d.	n.d.
9	MeCN	6	7	2	1	16
10	Acetone	10	10	9	11	40
11	1,4-Dioxane	14	14	6	2	36
12	TBME	12	15	6	2	35
13	THF	9	6	7	5	27
14	Et_2O	8	8	n.d.	n.d.	16
15	DMF	n.d.	n.d.	n.d.	n.d.	n.d.
16	DMSO	6	10	n.d.	n.d.	16
17	Ethylbenzene	13	17	6	5	41
18	Benzene	15	17	6	5	43
19	Toluene	16	18	5	3	42

Table S1. [a] Standard general reaction conditions. [b] Yields are determined by ^{19}F NMR using trifluoromethyl-benzene as an internal standard.

2.2. Photocatalyst effect

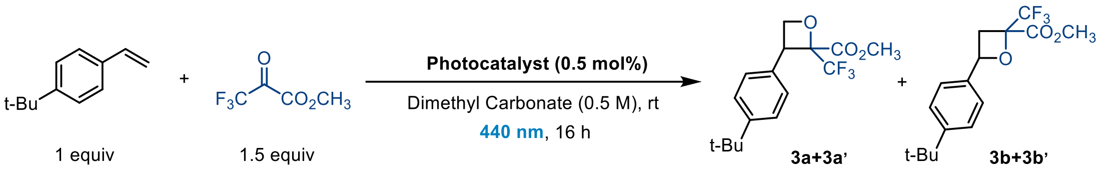

Entry[a]	Photocatalyst	a [%]	a' [%]	b [%]	b' [%]	Yield [%][b]
1	$Ir(ppy)_3$	trace	trace	n.d.	n.d.	trace
2	$Ir(dF(Me)ppy)_2dtbbpyPF_6$	19	18	7	4	48
3	$Ir(dF(CF_3)ppy)_2dtbbpyPF_6$	27	24	10	6	67
4	4CzIPN	25	22	2	7	56
5	Thioxanthone[c]	16	18	2	6	42
6	Benzophenone[c]	trace	trace	n.d.	n.d.	trace
7	$Ru(bpy)_3PF_6$	trace	trace	n.d.	n.d.	trace

Table S2. [a] Standard general reaction conditions. [b]. Yields are determined by ^{19}F NMR against an internal standard of trifluoromethylbenzene; [c] 5 mol% of thioxanthone was added, and the reaction was performed with 390 nm Kessil LED lamps.

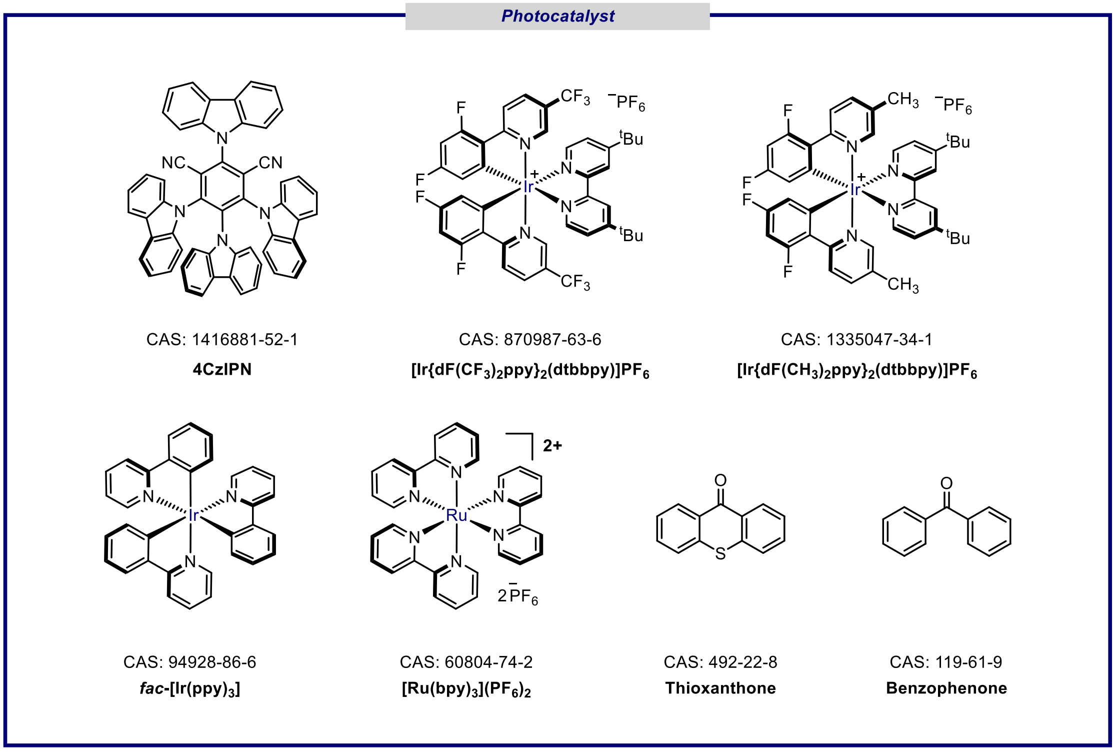

2.3. Loading of reagents

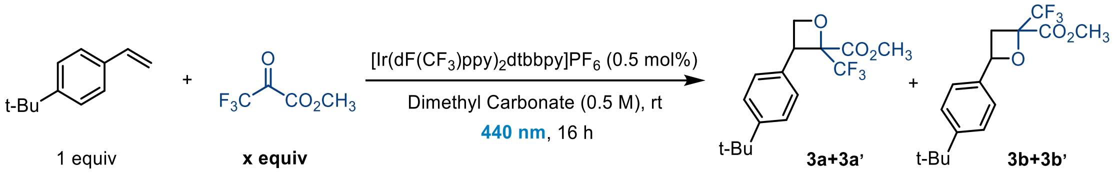

Entry[a]	Reagent Loadings	a [%]	a' [%]	b [%]	b' [%]	Yield [%][b]
1	1 eq	20	19	10	5	54
2	1.2 eq	23	21	9	5	58
3	1.5 eq	27	24	10	6	67
4	2.0 eq	27	25	5	8	65
5	3.0 eq	24	23	2	9	58

Table S3. [a] Standard general reaction conditions. [b] Yields are determined by ^{19}F NMR using trifluoromethylbenzene as an internal standard.

2.4. Loading of photocatalyst

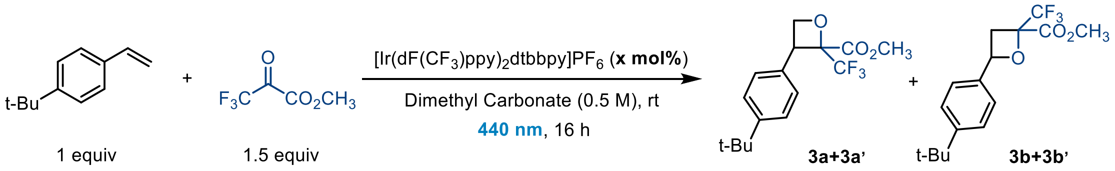

Entry[a]	Photocatalyst Loading [%]	a [%]	a' [%]	b [%]	b' [%]	Yield [%][b]
1	0.1	22	20	7	6	55
2	0.5	27	24	10	6	67
3	1	26	23	5	7	61
4	2	25	22	5	7	59
5	3	25	22	2	7	56
6	4	24	21	3	5	53
7	5	22	21	3	6	52

Table S4. [a] Standard general reaction conditions. [b] Yields are determined by ^{19}F NMR using trifluoromethylbenzene as an internal standard.

2.5. Concentration effect

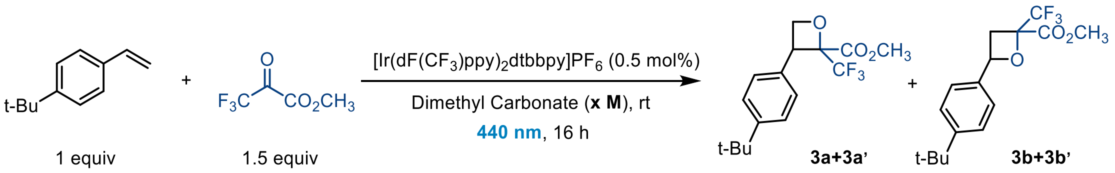

Entry[a]	Concentration	a [%]	a' [%]	b [%]	b' [%]	Yield [%][b]
1	0.25	25	22	13	4	64
2	0.5	27	24	10	6	67
3	1	27	24	11	5	67
4	1.25	28	28	9	6	71(70)
5	1.5	26	25	12	4	67
6	1.75	25	24	10	6	65
7	2	24	23	10	4	61

Table S5. [a] Standard general reaction conditions. [b] Yields are determined by ^{19}F NMR using trifluoromethylbenzene as an internal standard.

2.6. Control experiment

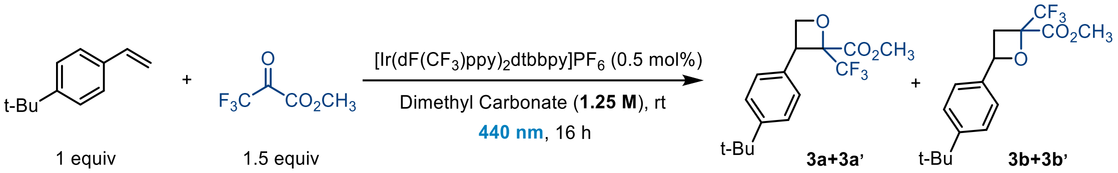

Entry[a]	Variations	a [%]	a' [%]	b [%]	b' [%]	Yield [%][b]
1	Without light	n.d.	n.d.	n.d.	n.d.	n.d.
2	Without photocatalyst	trace	trace	n.d.	n.d.	trace
3	With 0.5 eq TEMPO	trace	trace	n.d.	n.d.	trace
4	With 0.5 eq Et_3N	n.d.	n.d.	n.d.	n.d.	n.d.
5	With 0.5 eq acetic acid	23	23	11	4	61
6	With 0.5 eq water	11	11	6	2	30
7	Under air	23	22	10	2	57

Table S6. [a] Standard general reaction conditions. [b] Yields are determined by ^{19}F NMR using trifluoromethylbenzene as an internal standard.

3. Availability of starting materials

3.1. Commercially available starting materials

Commercially available starting materials were purchased from Thermoscientific – Acros, Sigma Aldrich, Apollo Scientific, Fluorochem, TCI or Chemie-Brunschwig.

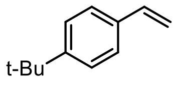

CAS 1746-23-2

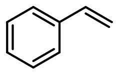

CAS 100-42-5

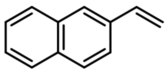

CAS 827-54-3

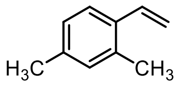

CAS 827-54-3

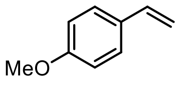

CAS 637-69-4

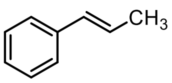

CAS 873-66-5

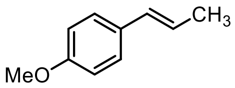

CAS 4180-23-8

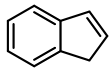

CAS 95-13-6

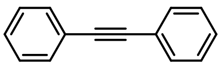

CAS 501-65-5

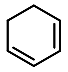

CAS 592-57-4

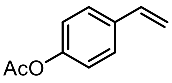

CAS 2628-16-2

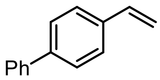

CAS 2350-89-2

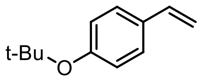

CAS 95418-58-9

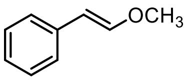

CAS 873-66-5

3.2. Prepared starting materials

Starting materials from S1-S14 were synthesized according to published procedures:

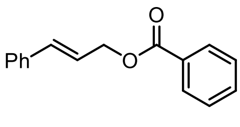

ref 2*, *S-1

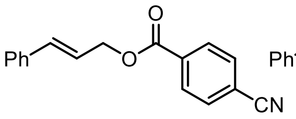

ref 3*, *S-2

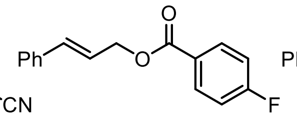

ref 2*, *S-3

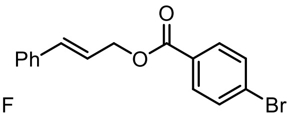

ref 4*, *S-4

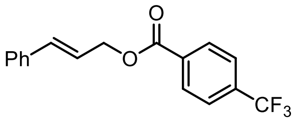

ref 4*, *S-5

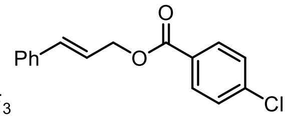

ref 2*, *S-6

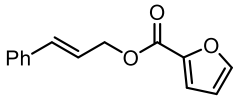

ref 2*, *S-7

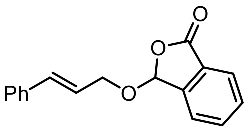

ref 5*, *S-8

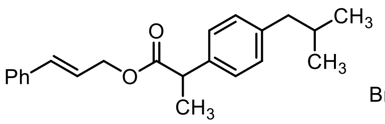

ref 6*, *S-9

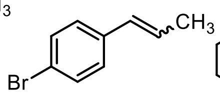

ref 2*, *S-10

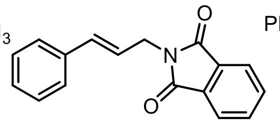

ref 3*, *S-11

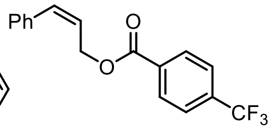

ref 7*, *S-12

4. General procedures

General procedure 1: A flame-dried 8 mL crimp cap vial was charged with Ir[dF(CF_3)ppy]$_2$(dtbbpy)PF_6 (5.6 mg, 5 µmol, 0.5 mol%) and equipped with a magnetic bar. The contents of the vial were then subjected to three vacuum/N_2 cycles. Dimethyl carbonate (0.8 mL) was added under an N_2 atmosphere, and the solution was degassed with N_2 for 5 min. Methyl 3,3,3-

trifluoro-2-oxopropanoate (153 μL, 1.50 mmol, 1.5 equiv.) and olefin (1.00 mmol, 1.0 equiv) were introduced to the solution via microsyringes (solid substrates were added after the photocatalyst, and then the cap was sealed). The reaction mixture was irradiated at room temperature under blue LEDs for 16 h. Reaction contents were purified by flash column chromatography. The isomers were isolated by prep-HPLC to afford the desired products.

General procedure 2: A flame-dried 8 mL crimp cap vial was charged with Ir[dF(CF_3)ppy]$_2$(dtbbpy)PF_6 (2.8 mg, 2.5 μmol, 0.5 mol%) and equipped with a magnetic bar. The contents of the vial were then subjected to three vacuum/N_2 cycles. Dimethyl carbonate (0.4 mL) was added under an N_2 atmosphere, and the solution was degassed with N_2 for 5 min. Methyl 3,3,3-trifluoro-2-oxopropanoate (77 μL, 0.75 mmol, 1.5 equiv.) or Ethyl 3,3,3-trifluoro-2-oxopropanoate (99 μL, 0.75 mmol) and olefin (0.50 mmol, 1.0 equiv) were introduced to the solution via microsyringes (solid substrates were added after the photocatalyst, and then the vial's cap was sealed). The reaction mixture was irradiated at room temperature under blue LEDs for 16 h. Reaction contents were purified by flash column chromatography to afford the desired products.

5. Synthesis of olefins

General procedure A

Under a nitrogen atmosphere, carboxylic acid (5 mmol), 1,3-dicyclohexyl-carbodiimide (1.2 eq), and 4-dimethylaminopyridine (0.1 eq) were added to a 100 mL round-bottom flask equipped with a magnetic stir bar. To this mixture, CH_2Cl_2 (0.2 M) and cinnamyl alcohol (1.1 eq) were added. The resulting solution was stirred under nitrogen for 12 h at room temperature, filtered through a celite pad, and evaporated to near dryness under vacuum. The resulting crude product was purified using flash column chromatography (silica gel), affording the pure alkene.

Cinnamyl benzoate (S1)

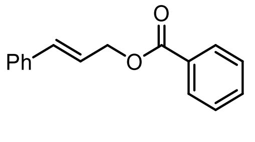

The titled compound was obtained as a white solid (85% yield, 5% ethyl acetate in hexane as eluent) according to general procedure A. The characterization data match the literature.[2]

^{1}H NMR (300 MHz, $CDCl_3$): δ 8.07 – 7.96 (m, 2H), 7.53 – 7.45 (m, 1H), 7.42 – 7.31 (m, 4H), 7.31 – 7.15 (m, 3H), 6.68 (d, *J* = 15.9 Hz, 1H), 6.34 (dt, *J* = 15.9, 6.4 Hz, 1H), 4.92 (dd, *J* = 6.4, 1.4 Hz, 2H).

^{13}C NMR (75 MHz, $CDCl_3$): δ 166.5, 136.4, 134.4, 133.1, 130.4, 129.8, 128.8, 128.5, 128.2, 126.8, 123.4, 65.7.

Cinnamyl 4-cyanobenzoate (S2)

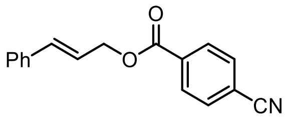

The titled compound was obtained as a white solid (67% yield, 10% ethyl acetate in hexane as eluent) according to a general procedure A. The characterization data match the literature.[3]

^{1}H NMR (300 MHz, $CDCl_3$): δ 8.21 – 8.17 (m, 2H), 7.78 – 7.73 (m, 2H), 7.53 – 7.20 (m, 5H), 6.78 (d, J = 15.9 Hz, 1H), 6.42 (dt, J = 15.8, 6.5 Hz, 1H), 5.04 (d, J = 6.5 Hz, 2H).
^{13}C NMR (75 MHz, $CDCl_3$): δ 164.7, 136.0, 135.1, 134.0, 132.2, 130.2, 128.7, 128.3, 126.7, 122.5, 118.0, 116.4, 66.3.

Cinnamyl 4-fluorobenzoate (S3)

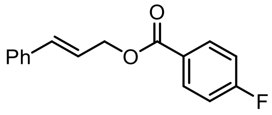

The titled compound was obtained as a white solid (87% yield, 5% ethyl acetate in hexane as eluent) according to a general procedure A. The characterization data match the literature.[2]
^{1}H NMR (300 MHz, $CDCl_3$): δ 8.10 – 7.95 (m, 2H), 7.38 – 7.13 (m, 5H), 7.10 – 6.95 (m, 2H), 6.66 (d, J = 15.9 Hz, 1H), 6.31 (dt, J = 15.9, 6.4 Hz, 1H), 4.89 (dd, J = 6.4, 1.3 Hz, 2H).
^{19}F NMR (282 MHz, $CDCl_3$): δ -105.59 (t, J = 6.2 Hz, 1F)
^{13}C NMR (75 MHz, $CDCl_3$): δ 167.6, 165.6, 164.2, 136.3, 134.6, 132.4, 132.3, 128.8, 128.3, 126.8, 126.6, 126.6, 123.2, 115.8, 115.5, 65.8.

Cinnamyl 4-bromobenzoate (S4)

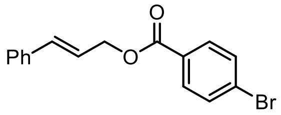

The titled compound was obtained as a white solid (86% yield, 5% ethyl acetate in hexane as eluent) according to a general procedure A. The characterization data match the literature.[4]
^{1}H NMR (300 MHz, $CDCl_3$): δ 8.03 – 7.93 (m, 2H), 7.67 – 7.57 (m, 2H), 7.51 – 7.26 (m, 5H), 6.77 (d, J = 15.9 Hz, 1H), 6.43 (dt, J = 15.9, 6.5 Hz, 1H), 5.01 (dd, J = 6.4, 1.3 Hz, 2H).
^{13}C NMR (75 MHz, $CDCl_3$): δ 165.7, 136.2, 134.7, 131.8, 131.3, 129.2, 128.7, 128.3, 128.2, 126.8, 123.0, 65.9.

Cinnamyl 4-(trifluoromethyl)benzoate (S5)

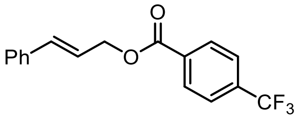

The titled compound was obtained as a white solid (78% yield, 5% ethyl acetate in hexane as eluent) according to general procedure A. The characterization data matches the literature.[4]
^{1}H NMR (300 MHz, $CDCl_3$): δ 8.16 – 8.06 (m, 2H), 7.62 (d, J = 8.2 Hz, 2H), 7.37 – 7.31 (m, 2H), 7.29 – 7.15 (m, 3H), 6.67 (d, J = 15.9 Hz, 1H), 6.32 (dt, J = 15.9, 6.5 Hz, 1H), 4.93 (dd, J = 6.5, 1.3 Hz, 2H).
^{19}F NMR (282 MHz, $CDCl_3$): δ -63.08 (s, 3F)
^{13}C NMR (75 MHz, $CDCl_3$): δ 165.3, 136.2, 135.0, 134.6 (q, J = 32.6 Hz), 133.6, 130.2, 128.8, 128.4, 126.8, 125.6 (q, J = 3.8 Hz), 123.8 (d, J = 272.6 Hz), 122.8, 66.2.

Cinnamyl 4-chlorobenzoate (S6)

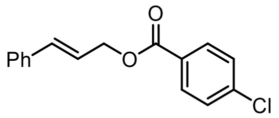

The titled compound was obtained as a white solid (67% yield, 5% ethyl acetate in hexane as eluent) according to a general procedure A. The characterization data matches the literature.[2]
^{1}H NMR (300 MHz, $CDCl_3$): δ 8.05 – 7.99 (m, 2H), 7.46 – 7.39 (m, 4H), 7.38 – 7.23 (m, 3H), 6.74 (d, *J* = 15.9 Hz, 1H), 6.40 (dt, *J* = 15.9, 6.5 Hz, 1H), 4.98 (dd, *J* = 6.4, 1.3 Hz, 2H).
^{13}C NMR (75 MHz, $CDCl_3$): δ 165.7, 139.6, 136.3, 134.8, 131.2, 128.9, 128.8, 128.3, 126.8, 123.1, 65.9.

Cinnamyl furan-2-carboxylate (S7)

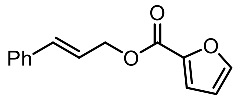

The titled compound was obtained as white solid (56% yield, 10% ethyl acetate in hexane as eluent) according to a general procedure A. The characterization data match the literature.[2]
^{1}H NMR (300 MHz, $CDCl_3$): δ 7.61 (dd, *J* = 1.8, 0.9 Hz, 1H), 7.47 – 7.40 (m, 2H), 7.38 – 7.32 (m, 2H), 7.32 – 7.27 (m, 1H), 7.27 – 7.23 (m, 1H), 6.76 (d, *J* = 15.8 Hz, 1H), 6.54 (dd, *J* = 3.5, 1.7 Hz, 1H), 6.40 (dt, *J* = 15.8, 6.5 Hz, 1H), 4.99 (dd, *J* = 6.5, 1.3 Hz, 2H).
^{13}C NMR (75 MHz, $CDCl_3$): δ 158.6, 146.5, 144.7, 136.2, 134.9, 128.7, 128.3, 126.8, 122.8, 118.2, 112.0, 65.5.

3-(Cinnamyloxy)isobenzofuran-1(3H)-one (S8)

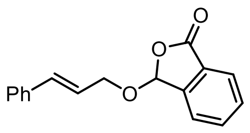

The titled compound was obtained as a white solid (72% yield, 10% ethyl acetate in hexane as eluent) according to a general procedure A. The characterization data match the literature.[5]
^{1}H NMR (300 MHz, $CDCl_3$): δ 7.97 – 7.88 (m, 1H), 7.77 – 7.71 (m, 1H), 7.67 – 7.58 (m, 2H), 7.48 – 7.40 (m, 2H), 7.40 – 7.24 (m, 3H), 6.74 (d, *J* = 15.9 Hz, 1H), 6.49 (s, 1H), 6.38 (ddd, *J* = 15.9, 7.0, 5.8 Hz, 1H), 4.69 – 4.46 (m, 2H).
^{13}C NMR (75 MHz, $CDCl_3$): δ 168.8, 145.2, 136.2, 134.8, 134.5, 131.0, 128.7, 128.3, 127.3, 126.8, 125.6, 123.8, 123.6, 101.3, 70.6.

Cinnamyl 2-(4-isobutylphenyl)propanoate (S9)

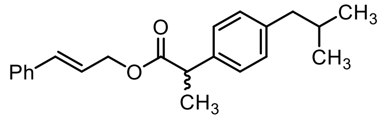

The titled compound was obtained as a colorless oil (73% yield, 5% ethyl acetate in hexane as eluent) according to a general procedure A. The characterization data match the literature.[6]
^{1}H NMR (300 MHz, $CDCl_3$): δ 7.30 – 7.15 (m, 7H), 7.06 (d, *J* = 8.0 Hz, 2H), 6.57 – 6.06 (m, 2H), 4.68 (dd, *J* = 6.1, 1.4 Hz, 2H), 3.70 (q, *J* = 7.1 Hz, 1H), 2.41 (d, *J* = 7.2 Hz, 2H), 1.81 (dp, *J* = 13.5, 6.7 Hz, 1H), 1.48 (d, *J* = 7.2 Hz, 3H), 0.86 (d, *J* = 6.6 Hz, 6H).
^{13}C NMR (75 MHz, $CDCl_3$): δ 174.6, 140.7, 137.8, 136.4, 133.7, 129.5, 128.6, 128.0, 127.3, 126.7, 123.3, 65.1, 45.3, 45.1, 30.3, 22.5, 18.7.

(E)-1-bromo-4-(prop-1-en-1-yl)benzene (S10)

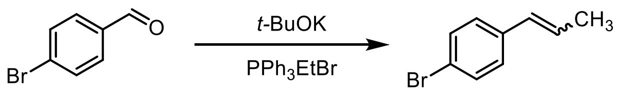

Ethyltriphenylphosphonium bromide (1.2 equiv, 24 mmol) was suspended in dry THF (0.2 M), and the solution was cooled to 0 °C. *tert*-BuOK (1.2 equiv, 24 mmol) was added in one portion, and the reaction was stirred at 0 °C for 30 min. Subsequently, 4-bromobenzaldehyde (1.0 equiv, 20 mmol) was added, and the mixture was warmed to RT and stirred until the starting material was consumed. The reaction was quenched with H_2O and extracted with EtOAc (3 × 80 mL). The combined organic layers were washed with water and dried over Na_2SO_4, filtered, and concentrated under reduced pressure. The crude product was purified by flash column chromatography on SiO_2 to give the alkene in E/Z mixture as a yellowish oil with 70% yield (2.76 g, 14 mmol).
^{1}H NMR (300 MHz, $CDCl_3$): δ 7.49 – 7.37 (m, 2H), 7.21 – 7.13 (m, 2H), 6.40 – 6.30 (m, 1H), 5.82 (dq, *J* = 11.6, 7.2 Hz, 1H), 1.87 (dd, *J* = 7.2, 1.8 Hz, 3H).
^{13}C NMR (75 MHz, $CDCl_3$): δ 137.0, 136.6, 131.7, 131.4, 130.6, 130.1, 128.9, 127.8, 127.5, 126.8, 120.4, 18.6, 14.7.

2-Cinnamylisoindoline-1,3-dione (S11)

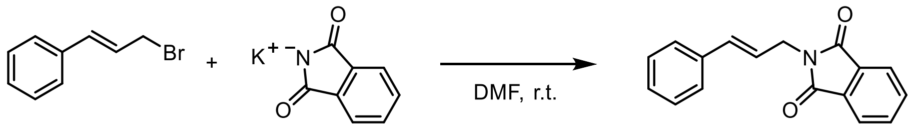

To a solution of the crude allyl bromide in DMF (50 mL) was added phthalimide potassium salt (11 mmol, 2.04 g), and the suspension was stirred at room temperature for 2 h. The resulting mixture was diluted with EtOAc (200 mL) and quenched with H_2O (200 mL). The aqueous layer was extracted with EtOAc (3×100 mL), and the combined organic layers were washed with H_2O (3×200 mL), dried over $MgSO_4$, filtered, and evaporated. The white solid obtained was recrystallized (Hexane/EtOAc).
The titled compound was obtained as a white solid. The characterization data match the literature.[3]
^{1}H NMR (300 MHz, $CDCl_3$): δ 7.89 (dd, *J* = 5.5, 3.1 Hz, 2H), 7.79 – 7.70 (m, 2H), 7.41 – 7.23 (m, 5H), 6.69 (dt, *J* = 15.8, 1.5 Hz, 1H), 6.28 (dt, *J* = 15.8, 6.5 Hz, 1H), 4.47 (dd, *J* = 6.5, 1.4 Hz, 2H).
^{13}C NMR (75 MHz, $CDCl_3$): δ 168.1, 136.4, 134.1, 133.9, 132.3, 128.7, 128.0, 126.7, 123.5, 122.9, 39.8.

Cinnamyl 4-(trifluoromethyl)benzoate (S12)

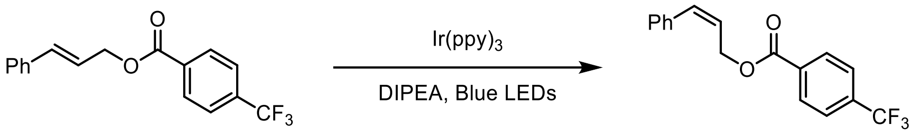

According to a reported procedure[7] In a flame-dried 8 mL crimp cap vial, Ir(ppy)$_3$ (4.6 mg, 7.0 µmol, 0.7 mol%), cinnamyl 4-(trifluoromethyl)benzoate (S5, 1.0 mmol, 306 mg), and equipped with a magnetic bar. The contents of the vial were then subjected to three vacuum/N_2 cycles. Acetonitrile (5 mL) was added under an N_2 atmosphere, and the solution was degassed with N_2 for 1 min. DIPEA (17 µL, 0.75 mmol, 0.1 equiv) were introduced to the solution via microsyringes. The reaction mixture was irradiated at room temperature under blue LEDs for 16 h. Reaction contents were purified by flash column chromatography on silica gel to afford the desired products as a white solid (72% yield, heptane as eluent).

^{1}H NMR (300 MHz, $CDCl_3$): δ 8.13 – 8.04 (m, 2H), 7.62 (d, *J* = 8.2 Hz, 2H), 7.35 – 7.15 (m, 5H), 6.68 (dt, *J* = 11.8, 1.8 Hz, 1H), 5.87 (dt, *J* = 11.7, 6.6 Hz, 1H), 5.04 (dd, *J* = 6.6, 1.6 Hz, 2H).

^{19}F NMR (282 MHz, $CDCl_3$): δ -63.11 (s, 3F).

^{13}C NMR (75 MHz, $CDCl_3$): δ 165.3, 136.1, 134.6 (q, *J* = 32.7 Hz), 133.8, 133.5 (d, *J* = 1.4 Hz), 130.2, 128.9, 128.6, 127.8, 125.6 (q, *J* = 3.8 Hz), 125.4, 122.0, 62.6.

6. Mechanistic Studies

6.1. Stern-Volmer quenching studies

To explore the dynamics of the excited state in greater detail, Stern-Volmer quenching studies were performed. The quenching efficiency is described by the Stern-Volmer equation,

$(I_0 \div I) - 1 = k_{sv}$ [quencher]

Where I_0 is the luminescence intensity in the absence of any quencher, I is the luminescence intensity in the presence of a predefined quencher concentration.

Preparation of stock solutions for Stern-Volmer measurements:

Two stock solutions of photocatalyst was prepared by dissolving $Ir[dF(CF_3)ppy]_2(dtbbpy)PF_6$ (0.59 mg, 0.5 µmol, 50 µM) and 4CzIPN (0.41 mg, 0.5 µmol, 50 µM) respectively in oxygen- and water-free dimethyl carbonate (10 mL) under nitrogen.

Stock solutions of the reaction components were prepared by dissolving the following reagents in 10 mL dimethyl carbonate:

4-*tert*-butylstyrene (**1**) = 40 mg, 2.5 x 10^{-4} mol

Methyl 3,3,3-trifluoro-2-oxopropanoate (**2**) = 40 mg, 2.5 x 10^{-4} mol

For evaluation of the quenching abilities of 4-*tert*-butylstyrene (**1**) and Methyl 3,3,3-trifluoro-2-oxopropanoate (**2**) in two photocatalysts, Quartz cuvettes (3.5 mL, 10 mm x 4 mm, PTFE cap) were filled with photocatalyst stock solution (0.5 mL) the relevant amount of the reagent stock solution, and then fill it up to 1.5 mL with oxygen- and water-free dimethyl carbonate.

Entry	Vol of PC stock (mL)	Vol of **1** stock (mL)	Vol of **2** stock (mL)
1	0.5	0	0
2	0.5	0.2	0.2
3	0.5	0.4	0.4
4	0.5	0.6	0.6
5	0.5	0.8	0.8
6	0.5	1.0	1.0

Table S7. Preparation of solutions for Stern-Volmer quenching studies.

The samples were further put into a secondary container (Schott wide screw cap bottle or a screwcap Falcon). All samples have been kept in the dark and were only taken out of the secondary container directly before mounting the samples on the spectrometer under the exclusion of light. Fluorescence Emission spectra were acquired as fast as possible after sample preparation (excitation at 450 ± 2 nm, 2 nm steps, excitation slit and emission slit: 2 nm).

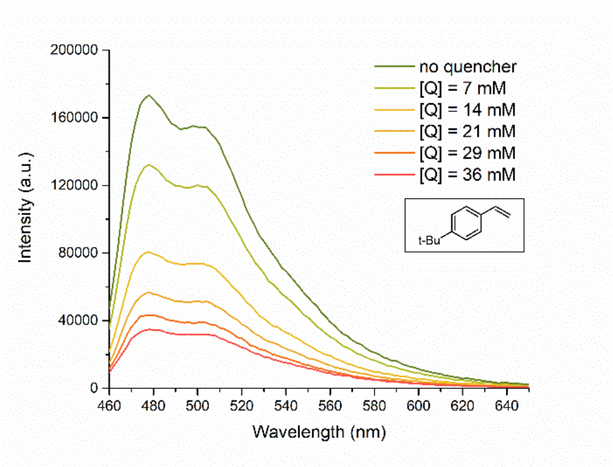

Figure S3. Emission quenching of Ir[dF(CF_3)ppy]$_2$(dtbbpy)PF_6 with **1**, 20 °C, dem = 2 nm, dex = 2 nm, λex = 450 nm).

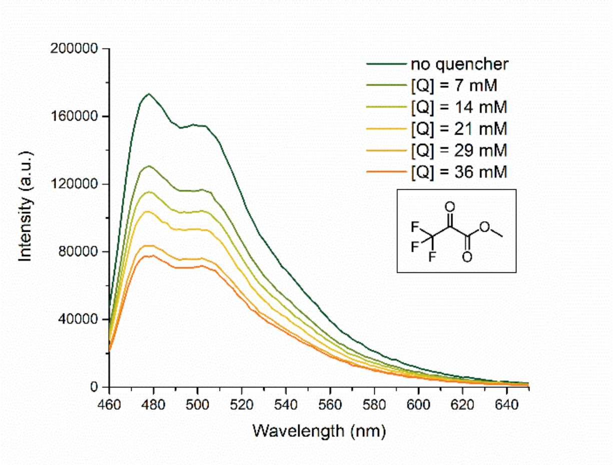

Figure S4. Emission quenching of Ir[dF(CF_3)ppy]$_2$(dtbbpy)PF_6 with **2**, 20 °C, dem = 2 nm, dex = 2 nm, λex = 450 nm).

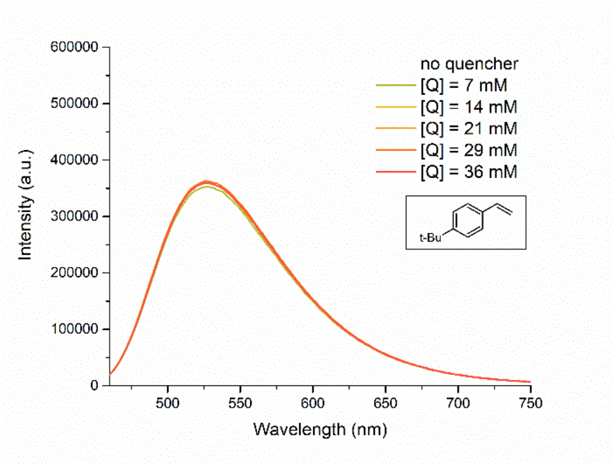

Figure S5. Emission quenching of 4CzIPN with **1**, 20 °C, dem = 2 nm, dex = 2 nm, λex = 450 nm).

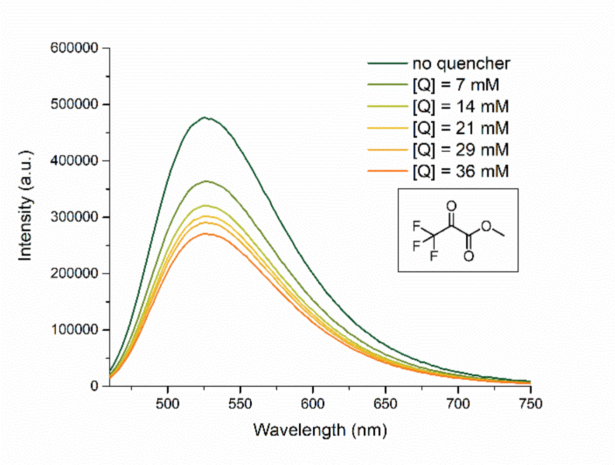

Figure S6. Emission quenching of 4CzIPN with **2**, 20 °C, dem = 2 nm, dex = 2 nm, λex = 450 nm).

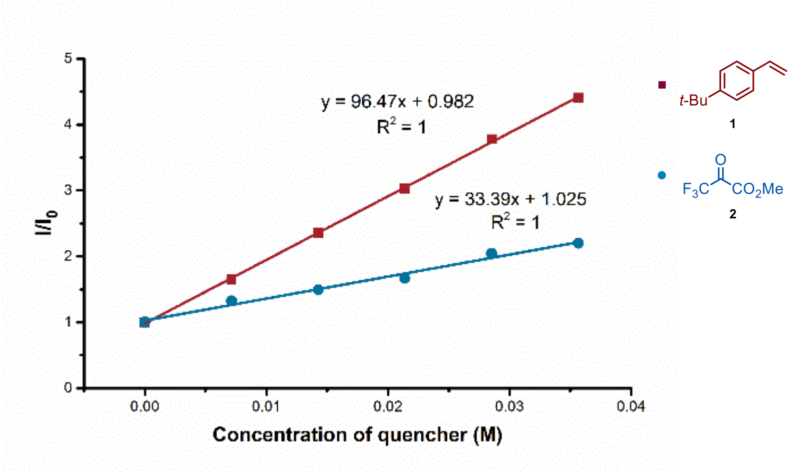

Figure S7. Correlation between emission intensity of Ir[dF(CF3)ppy]2(dtbbpy)PF6 and concentration of reagent **2** and 4-*tert*-butylstyrene.

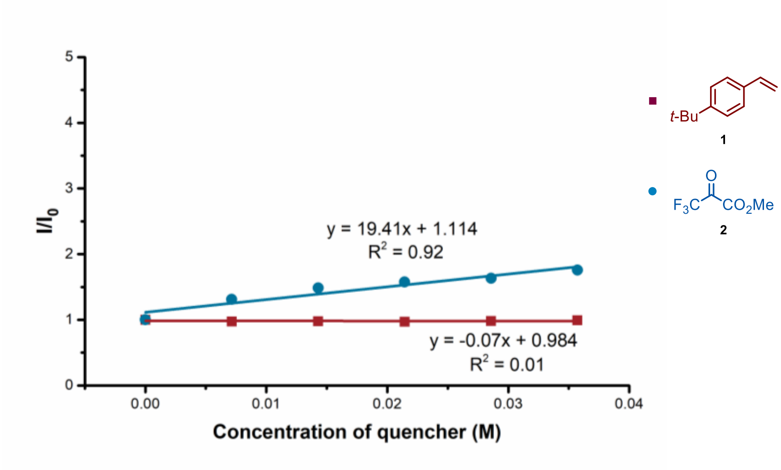

Figure S8. Correlation between emission intensity of 4CzIPN and concentration of reagent **2** and 4-*tert*-butylstyrene.

The influence on the emission of the catalyst by reagent **1** and **2** was investigated. Calculation of the quenching constants were performed for 4-*tert*-butylstyrene (**1**) and Methyl 3,3,3-trifluoro-2-oxopropanoate (**2**) according to the following equation:

For PC = Ir[dF(CF_3)ppy]$_2$(dtbbpy)PF_6	For PC = 4CzIPN
$(I_0 \div I) - 1 = k_{sv}$ [quencher **1**]	$(I_0 \div I) - 1 = k_{sv}$ [quencher **1**]
From slope $k_{sv} = 96\ M^{-1}$	From slope $k_{sv} = 0\ M^{-1}$
$(I_0 \div I) - 1 = k_{sv}$ [quencher **2**]	$(I_0 \div I) - 1 = k_{sv}$ [quencher **2**]
From slope $k_{sv} = 33\ M^{-1}$	From slope $k_{sv} = 19\ M^{-1}$

The emission quenching data clearly shows that the excited state of the catalyst Ir[dF(CF_3)ppy]$_2$(dtbbpy)PF_6 ($E(T_1)$ = 61.8 kcal/mol) was quenched by both substrates, while 4-*tert*-butylstyrene (**1**) showed more quenching efficiency than methyl 3,3,3-trifluoro-2-oxopropanoate (**2**), however, when switching to 4CzIPN ($E(T_1)$ = 58.3 kcal/mol), 4-*tert*-butylstyrene (**1**) showed no quenching while the pyruvate (**2**) demonstrated weaker quenching efficiency.

6.2. Cyclic voltammetry

5.81 g (15.0 mmol, 0.1 M) of NBu_4PF_6 was dissolved in 150 mL anhydrous MeCN in a 250 mL Schlenk tube, and the solution was degassed for 20 min by bubbling N_2. For each measurement, 10 mL of the solution was taken to the cyclovoltammetric cell, and the analyte was added. The resulting solution was stirred for 1 min to ensure homogeneity. Prior to each measurement, the Pt disc working electrode was polished with 0.05 μm alumina slurry, thoroughly rinsed with acetone: ethanol 1:1 solution and deionized water, then dried under a nitrogen stream. The solution was purged with N_2, and the glassy carbon electrode was rotated to homogenize the probe solution. During the measurement, the solution was protected by a positive N_2 stream.

The reduction potential of of methyl trifluoropyruvate was determined to be $E_{p/2}$ = -1.10 V vs Fc^+/Fc, corresponding to -0.70 V vs SCE.[8] Although this value formally lies within the reduction window of *[Ir-F] = -1.0 V and *4CzIPN = -1.04 V vs SCE,[9] no product formation was observed when employing $Ru(bpy)_3(PF_6)_2$ ($E[*Ru^{2+}/Ru^{3+}]$: -0.81 V vs SCE) or $Ir(ppy)_3$ ($E[*Ir^{III}/Ir^{IV}]$ = -1.73 V vs SCE) as photocatalysts, which support an energy transfer pathway.

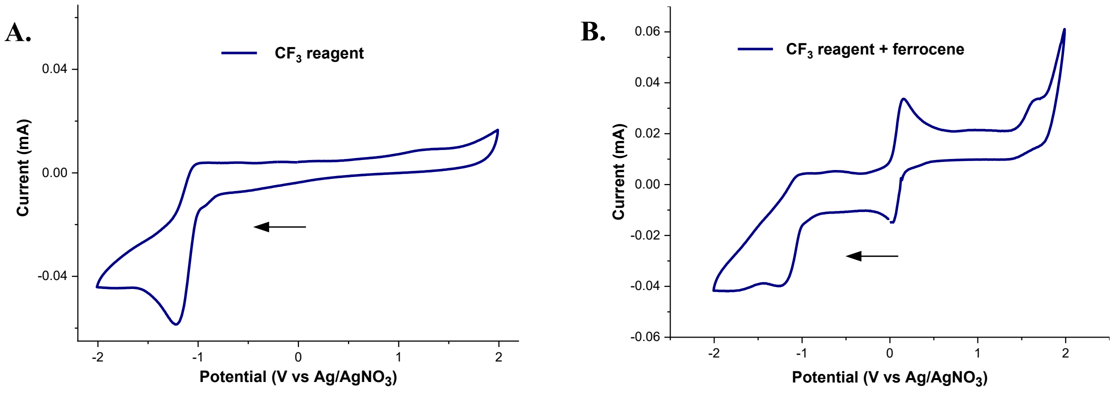

Figure S9. A. Cyclic voltammetry of reagent **2**. **B.** Cyclic voltammetry of reagent **2** in presence of ferrocene (5.0 mg, 0.03 mmol) as reference. Cyclic voltammetry in IUPAC convention, using reagent **2** (0.05 mmol), 10 mL 0.1 M NBu_4PF_6 MeCN solution using Pt disc working electrode (2 mm diameter); Pt counter electrode and an Ag^+ (0.01 M $AgNO_3$ in 0.1 M NBu_4PF_6)/Ag reference electrode, scan rate 100 mV·s^{-1}. Initial potential 0 V, switching potentials 2.0 V and -2.0 V, the direction of the initial scan was reductive.

6.3. Time-resolved ^{1}H NMR experiments

To investigate the nature of the key diradical species and to examine the influence of the alkene E/Z configuration on the product formation, we performed time-resolved ^{1}H NMR experiments under standard conditions using separately prepared E- and Z-isomers of the alkenes. In the dark, the E-type and Z-type alkenes (0.10 mmol each) were placed separately in flame-dried 8 mL crimp-cap vials, followed by the addition of Ir[dF(CF_3)ppy]$_2$(dtbbpy)PF_6 (0.56 mg, 1 µmol, 1 mol%). Each vial was equipped with a magnetic stir bar. The vials were subjected to three vacuum/N_2 cycles. Dimethyl carbonate (0.40 mL) was added under N_2, and the solutions were degassed with N_2 for 1 min. Methyl 3,3,3-trifluoro-2-oxopropanoate (15 µL, 0.15 mmol) was then introduced via microsyringe. The reaction mixtures were irradiated at room temperature under blue LEDs. For NMR analysis, 50 µL of the reaction mixture was withdrawn with a microsyringe and transferred into an NMR tube containing $CDCl_3$. The initial ^{1}H NMR spectra were recorded to confirm the E/Z configurations of the starting materials. Subsequent NMR spectra were collected after 2 h and 4 h of irradiation. *E*/*Z* interconversion was observed during irradiation, indicating that the alkene enters its triplet excited state under the reaction conditions.

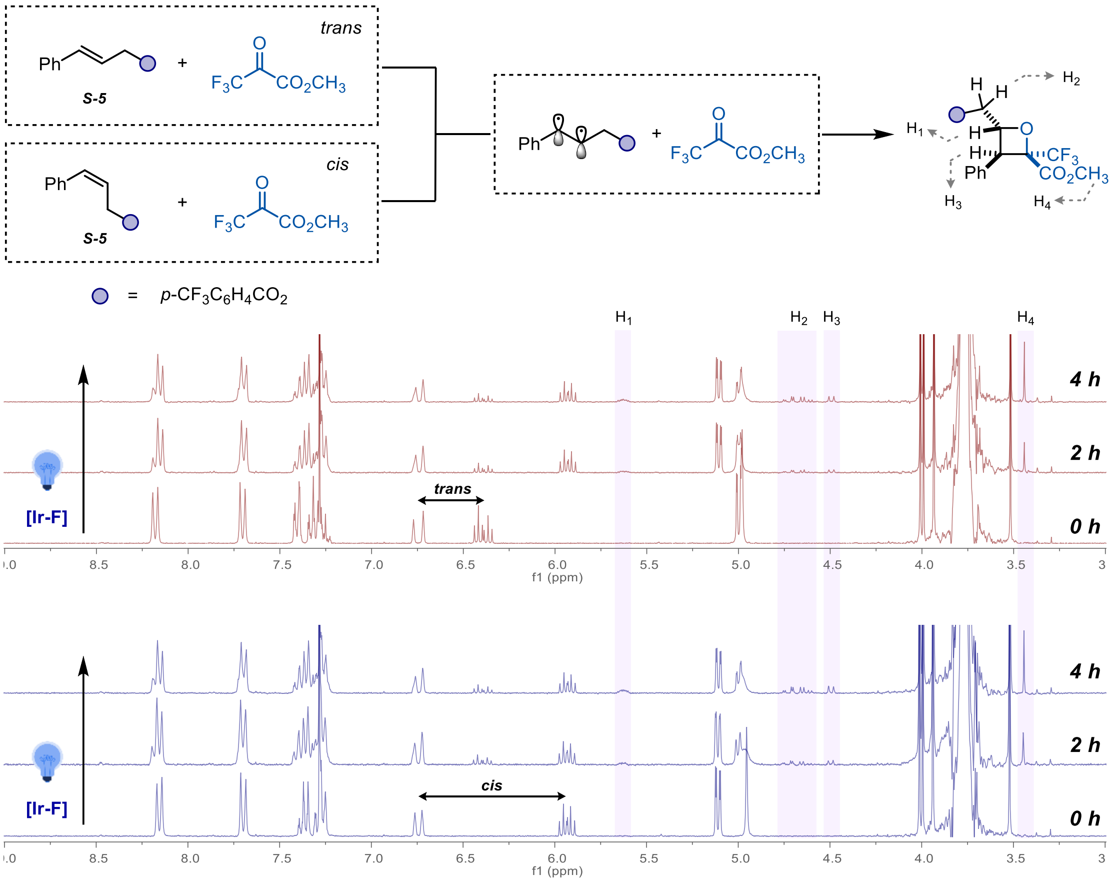

Figure S10. Time-resolved ^{1}H NMR experiments with E- and Z-alkenes to examine the reactive diradical and E/Z effects.

7. Computational details

7.1. Computational methods

The DFT calculations have been performed using the Gaussian 09 package.[10]
The conformational space of all molecules has been initially searched using meta-dynamics simulations based on tight-binding quantum chemical calculations as implemented in the software package Conformer-Rotamer Ensemble Sampling Tool CREST.[11]

The structures located with CREST have been subjected to geometry optimization using the ωB97X-D[12] functional with the def2SVP basis set,[13,14] including the polarizable continuum model (PCM)[15] with SMD parameters[16] to account for solvent effects (SMD parameters for ethyl acetate are available in the used software package). The nature of all stationary points was verified through the computation of the vibrational frequencies. Single-point (SP) energies for these geometries were calculated at the (U)ωB97X-D/def2TZVPP level of theory, including the SMD solvation model (SMD parameters for ethyl acetate are available in the software package used). Calculations were performed using "opt=tight" and "integral=ultrafine" keywords. Open shell singlet calculations were run using "guess=mix" and "stable=opt" additional keywords, and multiplicity of 1. The thermal corrections to the Gibbs free energies were combined with the single-point energies to yield Gibbs free energies (ΔG) at 298.15 K. All energies are reported in kcal·mol^{-1} unless otherwise stated. The choice of the ωB97X-D functional with the def2TZVPP basis set for single-point energies follows the benchmarking on diradical systems reported by Houk, Garg and co-workers for the treatment of diradical systems, who identified this level of theory as providing excellent performance.[17] Visualizations of molecules were prepared with Legault's CYLview20.9.[18]

7.2. Mechanistic studies

Unless otherwise stated, calculations below were performed at the (U)ωB97X-D/def2TZVPP,SMD(EtOAc)//(U)ωB97X-D/def2SVP,SMD(EtOAc) level of theory, taking the energy of ground state styrene as reference (ΔG = 0 kcal·mol⁻¹).

7.2.1. Scan involving triplet carbonyl species

DFT calculations involving the triplet state of the styrene-carbonyl reactant complex systematically led to the formation of triplet styrene and ground state carbonyl. To investigate the reaction between triplet carbonyl and ground state styrene, a fragment approach was employed using gaussian to fix the multiplicity of the pyruvate (triplet) and styrene (singlet). The resulting wavefunction was then used as a guess to probe the potential energy surface along the reaction coordinate. The scan revealed the absence of a barrier for this pathway.

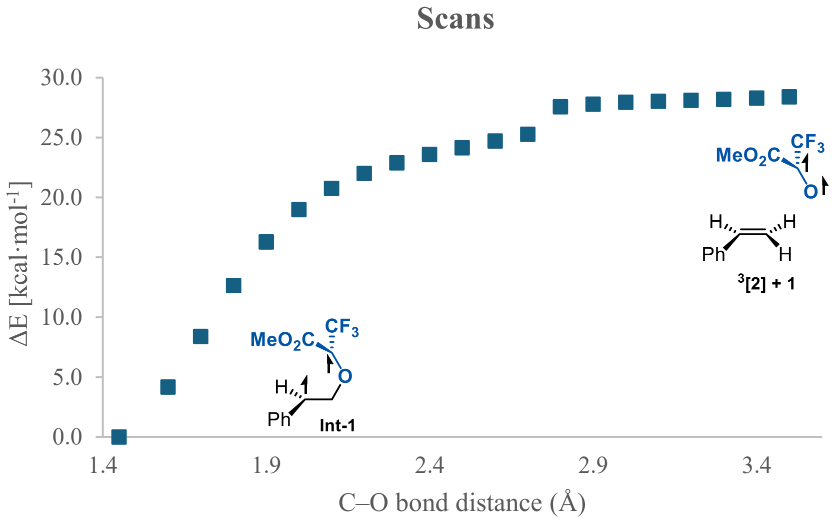

Figure S11. Scan for the addition of triplet state pyruvate onto ground state styrene, computed at the (U)ωB97X-D/def2SVP,SMD(EtOAc) level of theory.

7.2.2. First step: C–O *versus* C–C

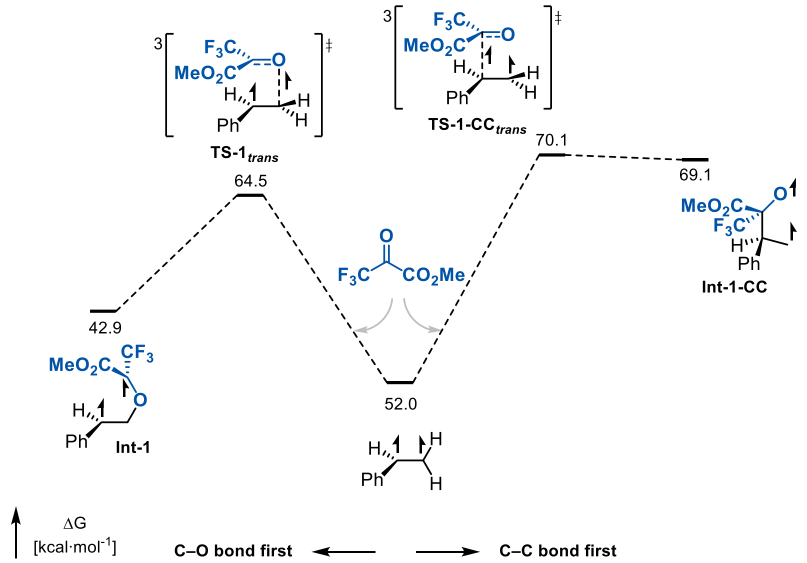

Figure S12. Computed competitive C–O *versus* C–C bond pathways, for the initial addition step involving styrene, leading to key diradical intermediates **Int-1** and **Int-1-CC**, respectively, toward major product **4a**.

DFT calculations were also performed to account for the formation of product **4b**. The competitive pathway to **4b** was found to be higher in energy ($\Delta G^{\ddagger}$ = 14.5 kcal·mol^{-1}), consistent with its formation as the minor product. Interestingly, this addition also proceeds through initial C–O bond formation but generates an unstable diradical intermediate **Int-1'** composed of primary and captodative radicals (ΔG = 4.3 kcal·mol^{-1}). A C–C-first pathway leading to **Int-1'-CC** showed a very similar energetic profile, suggesting that both *pathways a* and *b* may contribute productively to the formation of oxetane **4b** under the reaction conditions.

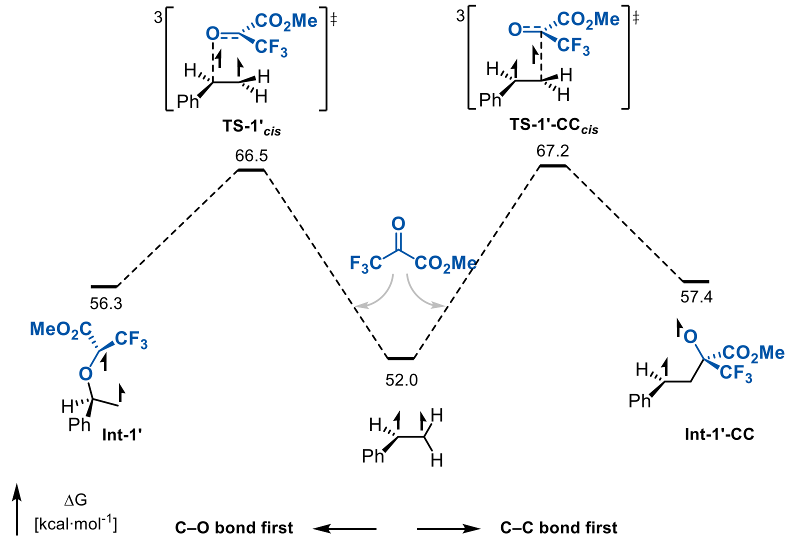

Figure S13. Computed competitive C–O *versus* C–C bond pathways, for the initial addition step involving styrene, leading to key diradical intermediates **Int-1'** and **Int-1'-CC**, respectively, toward minor product **4b**.

7.2.3. First step: facial approaches

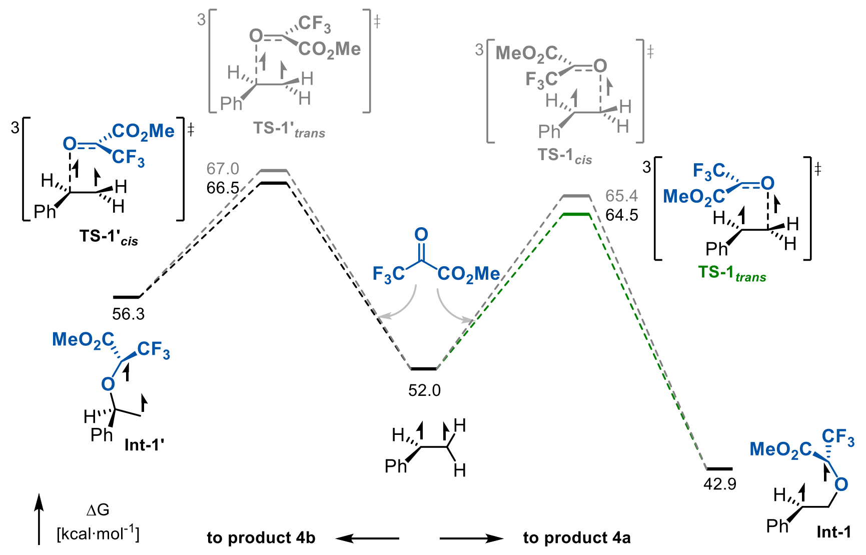

Figure S14. Computed competitive pathways for the initial addition step involving styrene, leading to key diradical intermediates **Int-1** and **Int-1'**, toward formation of product **4a** and **4b**, respectively.

For the pathway toward major product **4a**, these results show that a lower energy transition state is obtained for conformation where the CF_3 substituent is in *trans* relationship with respect to the phenyl ring (*via Si* face approach of the carbonyl). In the case of product **4b**, this is reversed and the conformation where the CF_3 substituent is in *cis* relationship with respect to the phenyl ring (*via Re* face approach of the carbonyl) is favored.

7.2.4. First step: substituted styrene

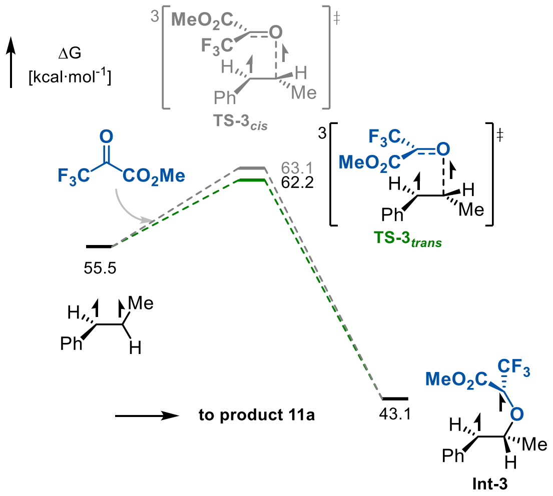

Figure S15. Computed profiles for the initial addition step involving *β*-methylstyrene, leading to key diradical intermediates **Int-3**. Energies referenced to β-methylstyrene (ΔG = 0 kcal·mol⁻¹).

7.2.5. Minimum energy crossing point (MECP)

To estimate the energy of the minimum energy crossing point (MECP) an initial IRC was conducted to obtain geometries along the reaction coordinates for the radical-radical recombination.

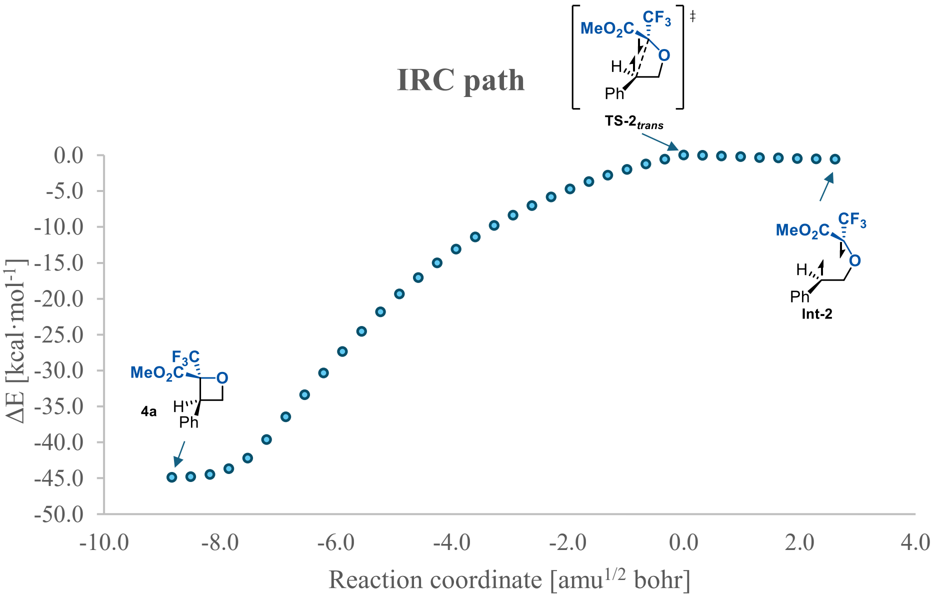

Figure S16. IRC profile obtained for OSS transition state **TS-2*trans***, computed at the (U)ωB97X-D/def2SVP,SMD(EtOAc) level of theory. Energies referenced to **TS-2*trans*** (ΔG = 0 kcal·mol⁻¹).

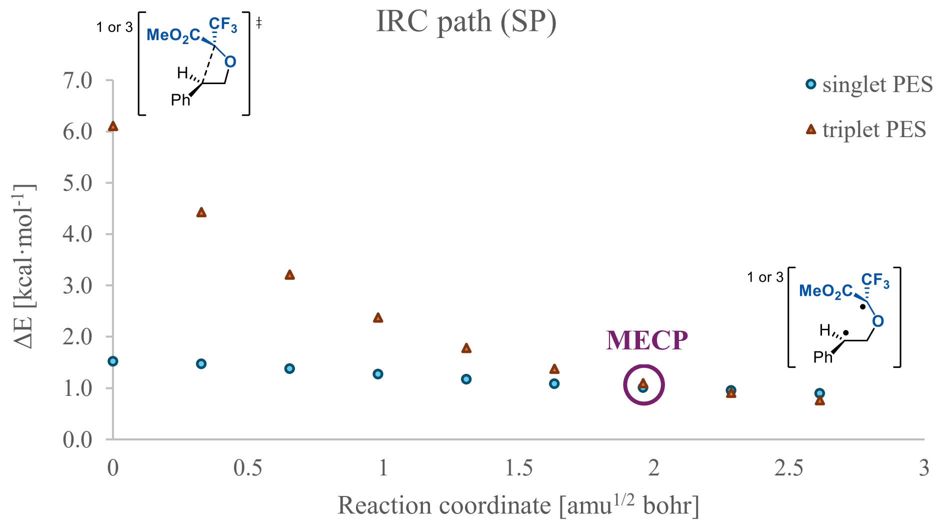

Figure S17. Single-point energies from IRC geometries, computed at the (U)ωB97X-D/def2TZVPP,SMD(EtOAc), for both open shell singlet and triplet states. Energies referenced to **Int-2** (ΔE = 0 kcal·mol⁻¹).

The MECP corresponds to the geometry at which the OSS and triplet potential energy surfaces intersect along the reaction coordinate. In this system, the MECP is located near 1.959 amu$^{1/2}$ bohr, with electronic energies of E_{OSS} = -989.1182072 Hartree and $E_{triplet}$ = -989.1180722 Hartree. The slightly higher energy of the triplet state was taken as a conservative estimate for the crossing point, corresponding to an electronic energy barrier of $\Delta E^{\ddagger}_{MECP}$ = +1.1 kcal·mol⁻¹ from diradical

intermediate **Int-2**.

7.2.6. Second step: radical-radical recombination

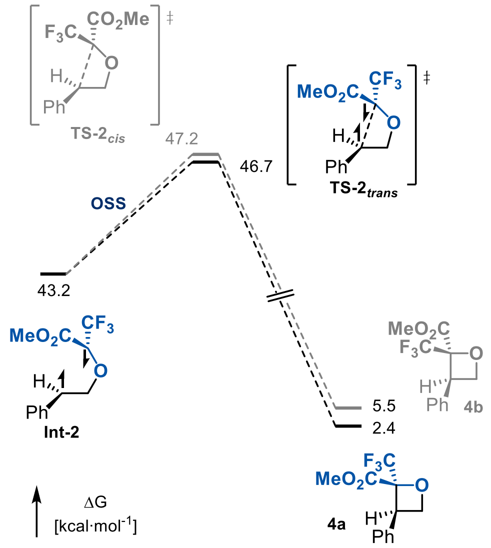

Figure S18. Computed competitive pathways for the radical coupling step from **Int-2**, leading to products **4a** and **4b**.

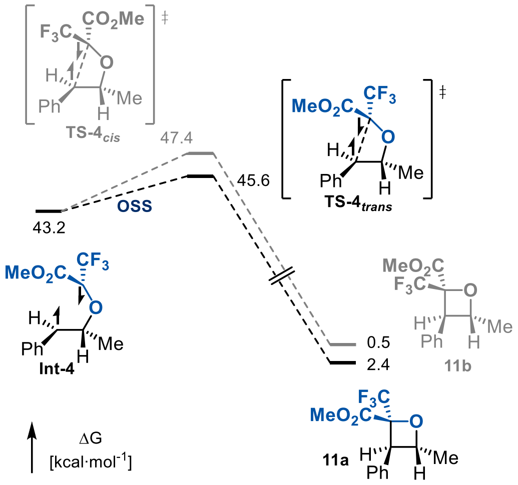

Figure S19. Computed competitive pathways for the radical coupling step from **Int-4**, leading to products **11a** and **11b**.

7.3. Computed energies

Table S8. Computed energies in Hartree unless otherwise stated. Computed at the (U)ωB97X-D/def2TZVPP,SMD(EtOAc)//(U)ωB97X-D/def2SVP,SMD(EtOAc) level of theory. [a] Translates to E(T_1) = 56.2 kcal·mol^{-1}. [b] Translates to E(T_1) = 63.1 kcal·mol^{-1}. [c] Translates to E(T_1) = 62.0 kcal·mol^{-1}.

Name	Im. Freq.	Thermal correction		SP energy	*H*	*G*
		to *H*	to *G*			
**H-styrene derivatives**						
**Styrene (1)**		0.142258	0.103823	-309.6574137	-309.5151557	-309.5535907
^{3}**[1]**[a]		0.137408	0.097198	-309.5679124	-309.4305044	-309.4707144
**2**$_{cis}$		0.088478	0.041177	-679.5103511	-679.4218731	-679.4691741
^{3}**[2**$_{cis}$**]**[b]		0.085975	0.037435	-679.4072436	-679.3212686	-679.3698086
**2**$_{trans}$		0.088462	0.041345	-679.5110987	-679.4226367	-679.4697537
^{3}**[2**$_{trans}$**]**[c]		0.08611	0.03737	-679.4122999	-679.3261899	-679.3749299
^{3}**[2] + 1**		0.230427	0.160403	-989.0787687	-988.8483417	-988.9183657
^{3}**[1] + 2**		0.228127	0.158843	-989.0879769	-988.8598499	-988.9291339
**TS-1**$_{trans}$	-223.83	0.227603	0.162426	-989.0830195	-988.8554165	-988.9205935
**TS-1-CC**	-290.92	0.228554	0.164554	-989.0762662	-988.8477122	-988.9117122
**Int-1-CC**		0.230051	0.165606	-989.0787772	-988.8487262	-988.9131712
**TS-1**$_{cis}$	-221.10	0.227325	0.160629	-989.079696	-988.852371	-988.919067
**TS-1'**$_{cis}$	-165.96	0.228494	0.162922	-989.0803638	-988.8518698	-988.9174418
**TS-1'**$_{trans}$	-181.52	0.228848	0.163781	-989.0804134	-988.8515654	-988.9166324
**Int-1**		0.232393	0.164828	-989.1198227	-988.8874297	-988.9549947
**Int-1'**		0.230606	0.162831	-989.096531	-988.865925	-988.9337
**TS-1'-CC**$_{cis}$	-150.72	0.22753	0.160017	-989.0762359	-988.8487059	-988.9162189
**Int-1'-CC**		0.233228	0.168381	-989.1002657	-988.8670377	-988.9318847
**Int-2**		0.232065	0.164693	-989.1192094	-988.8871444	-988.9545164
**TS-2**$_{\mathrm{trans}}$	-219.57	0.231373	0.168493	-989.1174012	-988.8860282	-988.9489082
**TS-2**$_{cis}$	-339.75	0.230981	0.167027	-989.1152023	-988.8842213	-988.9481753
**4a**		0.235346	0.173629	-989.193079	-988.957733	-989.01945
**4b**		0.235496	0.17452	-989.1890565	-988.9535605	-989.0145365
**4a'**		0.235111	0.172262	-989.1967564	-988.9616454	-989.0244944
**4b'**		0.235137	0.173105	-989.1957017	-988.9605647	-989.0225967
**Me-styrene derivatives**						
**Me-styrene**		0.171858	0.129536	-348.980363	-348.808505	-348.850827
^{3}**[Me-styrene]**		0.167483	0.122674	-348.8919321	-348.7244491	-348.7692581
**TS-3**$_{trans}$	-107.79	0.257352	0.187615	-1028.409133	-1028.151781	-1028.221518
**TS-3**$_{cis}$	-106.57	0.25707	0.185665	-1028.405636	-1028.148566	-1028.219971
**Int-3**		0.261586	0.19138	-1028.443339	-1028.181753	-1028.251959
**Int-4**		0.261414	0.19256	-1028.442849	-1028.181435	-1028.250289
**TS-4**$_{trans}$	-149.17	0.260517	0.193942	-1028.441899	-1028.181382	-1028.247957
**TS-4**$_{cis}$	-74.49	0.26028	0.192965	-1028.43794	-1028.17766	-1028.244975

Name	Im. Freq.	Thermal correction		SP energy	*H*	*G*
		to *H*	to *G*			
**11a**		0.264658	0.199853	-1028.519646	-1028.254988	-1028.319793
**11b**		0.264694	0.199237	-1028.515935	-1028.251241	-1028.316698

7.4. Computed geometries (xyz coordinates)

styrene

C	-1.024658000	-0.323104000	0.024186000
C	-0.968971000	-1.713531000	0.006625000
C	0.263394000	-2.370987000	-0.014633000
C	1.440218000	-1.623954000	-0.016348000
C	1.382919000	-0.230958000	0.001769000
C	0.152702000	0.444030000	0.018864000
C	0.147794000	1.921642000	0.030978000
C	-0.919271000	2.723872000	-0.041215000
H	-1.999101000	0.170143000	0.047454000
H	-1.896383000	-2.291399000	0.012150000
H	0.303645000	-3.462750000	-0.027552000
H	2.409920000	-2.127299000	-0.031251000
H	2.309781000	0.349517000	0.000242000
H	1.140284000	2.381445000	0.102333000
H	-0.800612000	3.810345000	-0.023765000
H	-1.941660000	2.342990000	-0.123843000

^{3}[styrene]

C	-0.997516000	-0.291095000	0.058703000
C	-0.967880000	-1.677248000	0.111978000
C	0.251801000	-2.364281000	0.077975000
C	1.449191000	-1.642120000	-0.009992000
C	1.432167000	-0.256614000	-0.064073000
C	0.205221000	0.463768000	-0.031343000
C	0.195201000	1.883956000	-0.088979000
C	-1.006494000	2.707842000	-0.055608000
H	-1.953923000	0.238722000	0.086716000
H	-1.904937000	-2.235512000	0.181126000
H	0.269663000	-3.455726000	0.119474000
H	2.404012000	-2.173170000	-0.037144000
H	2.370775000	0.300099000	-0.133412000
H	1.170715000	2.386921000	-0.161470000
H	-1.406529000	3.094158000	0.890218000
H	-1.511467000	3.020300000	-0.978171000

2_{cis}

C	1.910824000	1.242382000	0.076724000
C	0.731821000	0.275252000	-0.216840000
C	0.989567000	-1.245201000	-0.193481000
O	2.259793000	-1.520748000	0.016703000
O	0.102709000	-2.033196000	-0.352959000
C	2.629768000	-2.900499000	0.074394000
H	2.420875000	-3.392107000	-0.885944000
H	3.704533000	-2.916534000	0.282178000
H	2.079200000	-3.410127000	0.877123000
O	-0.357211000	0.701707000	-0.448834000
F	2.425314000	1.000546000	1.279464000
F	2.870085000	1.104269000	-0.834301000
F	1.484496000	2.495025000	0.048228000

^{3}[2_{cis}]

C	0.136631000	-1.959832000	-1.380665000
C	-0.520158000	-0.733171000	-0.759272000
C	0.148960000	0.131167000	0.233972000
O	-1.716228000	-0.457194000	-1.156428000
O	-0.350160000	1.116454000	0.715303000
F	-0.699731000	-2.532414000	-2.242577000
F	0.457704000	-2.859259000	-0.456920000
F	1.246642000	-1.634873000	-2.035822000
O	1.360146000	-0.343530000	0.505355000
C	2.139360000	0.391863000	1.447128000
H	3.077066000	-0.163038000	1.558338000
H	2.343200000	1.404921000	1.072365000
H	1.621347000	0.456811000	2.414111000

2_{trans}

C	-1.965612000	-0.357272000	-1.209302000
C	-0.515031000	-0.774901000	-0.847238000
C	0.182823000	0.113729000	0.197452000
O	-0.003844000	-1.723073000	-1.358246000
O	-0.339181000	1.090767000	0.663298000
F	-2.470266000	-1.204101000	-2.092942000
F	-1.986366000	0.862784000	-1.736992000
F	-2.737434000	-0.368933000	-0.126374000
O	1.376713000	-0.348650000	0.481360000
C	2.136393000	0.380270000	1.448687000
H	3.082100000	-0.159921000	1.560255000
H	2.323181000	1.404189000	1.095970000
H	1.602025000	0.414836000	2.408286000

^{3}[2_{trans}]

C	-1.957424000	-0.353519000	-1.217507000
C	-0.550010000	-0.708493000	-0.778094000
C	0.143079000	0.125669000	0.221686000
O	0.041800000	-1.723497000	-1.285560000
O	-0.361581000	1.096426000	0.729731000
F	-2.395520000	-1.239886000	-2.105399000
F	-2.000000000	0.853668000	-1.776489000
F	-2.797249000	-0.344330000	-0.185758000
O	1.359442000	-0.343667000	0.465346000
C	2.143305000	0.366900000	1.425175000
H	3.094624000	-0.171870000	1.490334000
H	2.318358000	1.400554000	1.095326000
H	1.646676000	0.371768000	2.405425000

^{3}[2] + 1

C	-2.787064000	0.992770000	-1.343320000
C	-3.428649000	1.389126000	-0.170867000
C	-2.937926000	0.954498000	1.062888000
C	-1.821196000	0.126614000	1.123929000
C	-1.169022000	-0.288415000	-0.049226000
C	-1.667599000	0.163370000	-1.280459000
C	0.015155000	-1.170873000	-0.041458000
C	0.636048000	-1.675033000	1.030250000
H	-3.158488000	1.330356000	-2.313729000
H	-4.306842000	2.037541000	-0.215525000
H	-3.432073000	1.264070000	1.986989000
H	-1.453196000	-0.200137000	2.098838000
H	-1.165512000	-0.141493000	-2.202500000
H	0.411796000	-1.411809000	-1.033545000
H	1.513982000	-2.315338000	0.913688000
H	0.308683000	-1.473418000	2.054124000
H	0.260691000	4.121515000	-0.046167000
H	-0.832216000	2.716775000	-0.290516000
H	-0.806531000	3.574012000	1.291065000
C	2.570234000	0.925919000	0.792130000
O	2.559268000	0.859821000	2.071340000
C	1.604368000	1.796255000	0.097862000
C	3.622253000	0.164352000	0.014947000
O	1.581971000	1.935979000	-1.100388000
O	0.796567000	2.383122000	0.970325000
C	-0.204276000	3.249497000	0.436089000
F	4.219563000	-0.722926000	0.806807000
F	3.097914000	-0.483741000	-1.018545000
F	4.560776000	0.983059000	-0.465934000

^{3}[1] + 2

C	-2.283604000	0.883225000	-1.609093000
C	-3.227039000	1.279557000	-0.652691000
C	-3.065266000	0.890203000	0.682713000
C	-1.976858000	0.118781000	1.062417000
C	-1.011168000	-0.301594000	0.107238000
C	-1.194535000	0.106391000	-1.242003000
C	0.113098000	-1.088894000	0.484024000
C	0.391273000	-1.526625000	1.845086000
H	-2.403475000	1.187912000	-2.651528000
H	-4.084117000	1.889627000	-0.946723000
H	-3.797621000	1.199570000	1.432454000
H	-1.849152000	-0.176606000	2.107343000
H	-0.458886000	-0.194486000	-1.991824000
H	0.786363000	-1.417384000	-0.320761000

H	0.042596000	-2.499748000	2.211786000
H	1.003658000	-0.918399000	2.521820000
H	0.213355000	4.137165000	0.823863000
H	-0.892624000	2.852613000	0.230477000
H	-0.757170000	3.180180000	1.996445000
C	2.583199000	0.825794000	0.438485000
O	2.902874000	0.495777000	1.540936000
C	1.505205000	1.874264000	0.132236000
C	3.341367000	0.274955000	-0.801378000
O	1.367043000	2.340825000	-0.967619000
O	0.824106000	2.185755000	1.208190000
C	-0.217506000	3.149859000	1.044563000
F	4.140158000	-0.715420000	-0.434485000
F	2.508559000	-0.185428000	-1.731849000
F	4.084851000	1.237597000	-1.341028000

TS-1$_{trans}$

C	-2.725120000	0.364573000	-1.665779000
C	-3.602851000	0.919030000	-0.722724000
C	-3.383678000	0.712850000	0.645168000
C	-2.297111000	-0.030693000	1.075584000
C	-1.395554000	-0.609228000	0.135367000
C	-1.642108000	-0.388813000	-1.251018000
C	-0.262316000	-1.349036000	0.545668000
C	0.068406000	-1.650465000	1.929905000
C	1.826001000	0.264189000	0.476642000
O	2.164169000	-0.154839000	1.567643000
C	0.914683000	1.450495000	0.267380000
C	2.531138000	-0.260404000	-0.793240000
O	0.753132000	1.981316000	-0.805238000
O	0.344369000	1.832332000	1.399154000
C	-0.531593000	2.951183000	1.318756000
F	3.147266000	-1.408666000	-0.525085000
F	1.709497000	-0.478536000	-1.822228000
F	3.458377000	0.614377000	-1.198868000
H	-2.896090000	0.529560000	-2.731736000
H	-4.456669000	1.513538000	-1.055320000
H	-4.068379000	1.146430000	1.377489000
H	-2.128554000	-0.178307000	2.143739000
H	-0.953948000	-0.814573000	-1.983866000
H	0.309619000	-1.868426000	-0.229695000
H	0.399858000	-2.654229000	2.207598000
H	-0.039853000	-0.901574000	2.715754000
H	0.038834000	3.870866000	1.120738000
H	-1.277385000	2.811765000	0.523785000
H	-1.027615000	3.025158000	2.292894000

TS-1-CC

C	-2.272480000	-0.001349000	-1.615196000
C	-3.092672000	0.378856000	-0.549734000
C	-2.715643000	0.084238000	0.762191000
C	-1.522859000	-0.584626000	1.013240000
C	-0.686077000	-0.973891000	-0.051077000
C	-1.079725000	-0.668435000	-1.368966000
C	0.587246000	-1.663287000	0.176602000
C	0.798657000	-2.464300000	1.373967000
C	2.035565000	-0.275647000	0.393241000
O	2.696439000	-0.869230000	1.301332000
C	1.243400000	0.953960000	0.810744000
C	2.727791000	-0.229694000	-0.984330000
O	0.982393000	1.205328000	1.956760000
O	0.852196000	1.674325000	-0.232250000
C	-0.005336000	2.779014000	0.027487000
F	3.402955000	-1.361651000	-1.189895000
F	1.903511000	-0.071512000	-2.022944000
F	3.612200000	0.776501000	-1.024936000
H	-2.567237000	0.224893000	-2.642031000
H	-4.029603000	0.906526000	-0.742770000
H	-3.355321000	0.381081000	1.595987000
H	-1.235489000	-0.792654000	2.044379000
H	-0.434249000	-0.958949000	-2.199688000
H	1.017210000	-2.103126000	-0.728780000
H	1.453795000	-3.335071000	1.325742000
H	0.357273000	-2.194542000	2.333642000
H	-0.889816000	2.456440000	0.594938000
H	0.524500000	3.559016000	0.593233000
H	-0.308617000	3.167779000	-0.950886000

Int-1-CC

C	-2.365068000	-0.754128000	-1.881701000
C	-3.160550000	-0.020538000	-1.002327000
C	-2.702270000	0.240538000	0.289247000
C	-1.455492000	-0.227034000	0.698529000
C	-0.642855000	-0.953526000	-0.182515000
C	-1.115046000	-1.215035000	-1.473753000
C	0.707746000	-1.499058000	0.241440000
C	0.701734000	-2.156955000	1.576124000
C	1.886965000	-0.357170000	0.199854000
O	2.914680000	-0.937202000	0.818371000
C	1.391405000	0.874078000	0.998618000
C	2.294595000	-0.048287000	-1.266774000
O	1.406446000	0.909511000	2.197273000
O	0.943767000	1.828517000	0.207319000
C	0.383136000	2.982392000	0.830479000
F	2.771166000	-1.155657000	-1.842137000
F	1.312239000	0.404662000	-2.037617000
F	3.275352000	0.860100000	-1.278179000
H	-2.718023000	-0.970076000	-2.892673000
H	-4.140024000	0.343293000	-1.321277000
H	-3.322487000	0.807369000	0.987423000
H	-1.121033000	-0.020935000	1.717766000
H	-0.493789000	-1.785351000	-2.168837000
H	1.051237000	-2.219693000	-0.511713000
H	1.171753000	-3.134702000	1.699711000
H	0.361552000	-1.622967000	2.465046000
H	-0.502210000	2.706124000	1.420553000
H	1.122660000	3.466835000	1.482784000
H	0.096167000	3.656878000	0.017023000

TS-1$_{cis}$

C	2.741894000	-1.629471000	0.922742000
C	3.724079000	-0.662760000	0.661475000
C	3.549948000	0.249910000	-0.387169000
C	2.406633000	0.210857000	-1.166861000
C	1.393914000	-0.760332000	-0.917528000
C	1.597629000	-1.684232000	0.149221000
C	0.192909000	-0.800598000	-1.659621000
C	-0.111390000	0.025297000	-2.817147000
C	-1.518487000	0.642703000	-0.275918000
O	-2.030495000	0.984873000	-1.325455000
C	-1.916462000	-0.600433000	0.488847000
C	-0.605957000	1.644626000	0.464550000
O	-1.539757000	-0.853723000	1.607416000
O	-2.746199000	-1.354275000	-0.218924000
C	-3.190112000	-2.557674000	0.397296000
F	0.376317000	1.097402000	1.170431000
F	-0.055006000	2.482314000	-0.414276000
F	-1.347059000	2.377306000	1.309268000
H	2.879850000	-2.338939000	1.741460000
H	4.625000000	-0.620406000	1.277456000
H	4.317252000	1.001186000	-0.586375000
H	2.272225000	0.933212000	-1.974264000
H	0.827161000	-2.430535000	0.356640000
H	-0.490449000	-1.632633000	-1.462360000
H	-0.553180000	-0.428949000	-3.707664000
H	0.129961000	1.089100000	-2.843265000
H	-3.719229000	-2.345297000	1.337335000
H	-3.872237000	-3.032927000	-0.316151000
H	-2.341280000	-3.225458000	0.606392000

TS-1'$_{cis}$

C	3.330208000	1.729586000	-0.348583000
C	4.388084000	1.044455000	0.256728000
C	4.323580000	-0.342730000	0.422756000
C	3.207394000	-1.039891000	-0.014527000
C	2.128680000	-0.364085000	-0.629986000
C	2.210324000	1.038575000	-0.788848000
C	0.987060000	-1.129116000	-1.047345000
C	-0.078536000	-0.616206000	-1.847748000
C	-1.319978000	-0.561491000	0.389274000
O	-0.214697000	-1.040928000	0.762128000
C	-2.439392000	-1.377566000	-0.127631000
C	-1.612061000	0.914137000	0.627264000
O	-3.549356000	-0.951422000	-0.370322000
O	-2.082833000	-2.654720000	-0.306312000
C	-3.087941000	-3.519445000	-0.809561000
F	-0.493824000	1.556624000	0.973700000
F	-2.499943000	1.112546000	1.614639000
F	-2.099756000	1.537319000	-0.457685000
H	3.382042000	2.813085000	-0.473395000
H	5.266452000	1.594558000	0.601932000
H	5.149304000	-0.876158000	0.898088000
H	3.148961000	-2.122788000	0.118272000
H	1.390866000	1.592045000	-1.249012000
H	1.057224000	-2.213609000	-0.951868000
H	-0.762791000	-1.308375000	-2.340413000
H	-0.162646000	0.442944000	-2.095847000
H	-2.626724000	-4.509875000	-0.901142000
H	-3.444391000	-3.184118000	-1.795336000
H	-3.946335000	-3.572919000	-0.123625000

TS-1'$_{trans}$

C	3.315294000	1.801447000	-0.292354000
C	4.379295000	1.119608000	0.305929000
C	4.340435000	-0.272263000	0.430253000
C	3.242801000	-0.977712000	-0.041738000
C	2.158431000	-0.305306000	-0.647432000
C	2.213288000	1.101601000	-0.763251000
C	1.039423000	-1.084262000	-1.108672000
C	-0.028574000	-0.571743000	-1.904311000
C	-1.294811000	-0.687863000	0.311254000
O	-0.193312000	-1.217274000	0.639187000
C	-1.583424000	0.754076000	0.459965000
C	-2.460711000	-1.577292000	-0.093472000
O	-2.661234000	1.270511000	0.249557000
O	-0.500867000	1.434255000	0.849369000
C	-0.646360000	2.837181000	0.977564000
F	-2.030098000	-2.819127000	-0.334308000
F	-3.090019000	-1.160772000	-1.203174000
F	-3.396442000	-1.664790000	0.866449000
H	3.348321000	2.888827000	-0.387337000
H	5.243254000	1.676052000	0.676514000
H	5.171404000	-0.803774000	0.898469000
H	3.205444000	-2.065363000	0.055574000
H	1.387262000	1.651593000	-1.215190000
H	1.152093000	-2.168906000	-1.075602000
H	-0.693768000	-1.263899000	-2.422669000
H	-0.136272000	0.491815000	-2.123817000
H	0.329919000	3.221450000	1.296384000
H	-1.406639000	3.090644000	1.731273000
H	-0.933978000	3.297719000	0.020106000

Int-1

C	-4.073466000	1.764034000	-0.360965000
C	-4.405740000	1.480938000	0.969678000
C	-3.752560000	0.437868000	1.636934000
C	-2.783033000	-0.317631000	0.994043000
C	-2.427232000	-0.049907000	-0.358047000
C	-3.106041000	1.017213000	-1.014336000
C	-1.447247000	-0.784300000	-1.072060000
C	-0.656914000	-1.938815000	-0.570320000
C	1.359715000	-0.657005000	-0.266720000
O	0.760224000	-1.710346000	-0.787732000
C	0.996491000	-0.086275000	1.029418000
C	2.517000000	-0.131866000	-1.078507000
O	0.306611000	-0.650223000	1.852596000
O	1.525009000	1.124437000	1.200755000
C	1.263407000	1.764456000	2.443828000
F	2.811948000	-0.963015000	-2.075175000
F	2.244751000	1.061994000	-1.624732000
F	3.620429000	0.021213000	-0.338735000
H	-4.577754000	2.577377000	-0.888238000
H	-5.168204000	2.070249000	1.483970000
H	-4.005126000	0.214905000	2.676387000
H	-2.277278000	-1.112875000	1.542524000
H	-2.851272000	1.243396000	-2.053117000
H	-1.240096000	-0.487277000	-2.105645000
H	-0.853924000	-2.841833000	-1.165973000
H	-0.828748000	-2.168030000	0.486816000
H	1.788069000	2.725361000	2.407504000
H	0.184450000	1.930732000	2.575899000
H	1.642052000	1.163042000	3.282698000

Int-1'

C	3.483050000	1.537158000	0.030967000
C	4.575088000	0.672236000	-0.059180000
C	4.364438000	-0.688338000	-0.281493000
C	3.066900000	-1.182340000	-0.412628000
C	1.971228000	-0.318352000	-0.327590000
C	2.186216000	1.046898000	-0.106701000
C	0.572709000	-0.862439000	-0.544804000
C	0.124400000	-0.641427000	-1.943425000
C	-1.604289000	-0.165599000	0.295202000
O	-0.289159000	-0.155276000	0.388200000
C	-2.446181000	-1.267885000	-0.160149000
C	-2.268512000	1.125351000	0.707364000
O	-3.634472000	-1.142993000	-0.364833000
O	-1.778932000	-2.415219000	-0.303381000
C	-2.524651000	-3.527792000	-0.783780000
F	-1.377713000	1.969288000	1.226909000
F	-3.225206000	0.935972000	1.618938000
F	-2.837103000	1.743822000	-0.337355000
H	3.642266000	2.602839000	0.211565000
H	5.591014000	1.059274000	0.048607000
H	5.214135000	-1.371991000	-0.345982000
H	2.903256000	-2.250356000	-0.579873000
H	1.332251000	1.722498000	-0.024642000
H	0.548846000	-1.931363000	-0.296441000
H	0.124671000	-1.464559000	-2.659908000
H	-0.060745000	0.379292000	-2.291446000
H	-1.820279000	-4.365293000	-0.836923000
H	-2.936482000	-3.320242000	-1.782019000
H	-3.347772000	-3.772541000	-0.097611000

TS-1'-CC$_{cis}$

C	-3.169188000	-1.187246000	0.206710000
C	-3.454759000	-2.532354000	-0.061002000
C	-2.444430000	-3.500975000	0.076936000
C	-1.172292000	-3.133943000	0.466476000
C	-0.848632000	-1.763726000	0.734896000
C	-1.899473000	-0.801393000	0.601990000
C	0.448788000	-1.406374000	1.118018000
C	0.896880000	-0.034284000	1.394089000
C	0.959092000	0.768496000	-1.234376000
O	0.847452000	-0.364065000	-1.627985000
C	2.326508000	1.336095000	-0.846478000
C	-0.233817000	1.754829000	-1.340717000
O	3.340254000	0.702688000	-0.957587000
O	2.236175000	2.580060000	-0.403284000
C	3.448790000	3.227067000	-0.025091000
F	-0.547593000	2.332602000	-0.180484000
F	-1.310411000	1.127034000	-1.791491000
F	0.066890000	2.723704000	-2.211439000
H	-3.954865000	-0.434495000	0.107351000
H	-4.459599000	-2.829333000	-0.369188000
H	-2.668125000	-4.550757000	-0.127369000
H	-0.387013000	-3.887626000	0.567013000
H	-1.698723000	0.248027000	0.821705000
H	1.183712000	-2.206316000	1.257669000
H	1.967943000	0.173002000	1.347700000
H	0.288975000	0.651395000	1.994503000
H	3.176318000	4.252733000	0.245295000
H	3.903222000	2.718934000	0.837695000
H	4.162170000	3.233173000	-0.861040000

Int-1'-CC

C	-3.132536000	-2.166999000	-0.053301000
C	-2.786994000	-3.489213000	0.249414000
C	-1.513161000	-3.769551000	0.760100000
C	-0.601599000	-2.746643000	0.968400000
C	-0.930471000	-1.392247000	0.674166000
C	-2.228630000	-1.134312000	0.150748000
C	0.026590000	-0.373677000	0.912778000
C	-0.175492000	1.085777000	0.667254000
C	0.277112000	1.492968000	-0.756947000
O	-0.455225000	0.912859000	-1.741868000
C	1.786699000	1.188263000	-1.000130000
C	0.063673000	3.032072000	-0.977719000
O	2.198206000	0.589126000	-1.950163000
O	2.520725000	1.659396000	-0.006755000
C	3.929373000	1.436165000	-0.082753000
F	0.589427000	3.734800000	0.015582000
F	-1.234206000	3.313117000	-1.039048000
F	0.629494000	3.430208000	-2.112833000
H	-4.122068000	-1.943023000	-0.459282000
H	-3.504124000	-4.296581000	0.085141000
H	-1.234595000	-4.799683000	0.995334000
H	0.391150000	-2.972871000	1.366852000
H	-2.513491000	-0.114731000	-0.112808000
H	1.006507000	-0.684063000	1.288882000
H	0.401622000	1.668851000	1.397690000
H	-1.232218000	1.374586000	0.768796000
H	4.356753000	1.890913000	0.817143000
H	4.146474000	0.359141000	-0.102782000
H	4.345256000	1.912309000	-0.981377000

Int-2

C	3.569519000	0.366716000	-1.631939000
C	4.263192000	-0.318707000	-0.623698000
C	3.737344000	-0.357405000	0.673536000
C	2.545978000	0.281841000	0.973680000
C	1.829370000	0.995458000	-0.028472000
C	2.377005000	1.008896000	-1.345730000
C	0.613928000	1.668474000	0.211564000
C	-0.138261000	1.728074000	1.500118000
C	-1.610217000	0.181738000	0.515453000
O	-1.495114000	1.261469000	1.275174000
C	-0.797374000	-1.014382000	0.767696000
C	-2.630856000	0.256038000	-0.586731000
O	-0.214061000	-1.214385000	1.810094000
O	-0.793390000	-1.844890000	-0.271109000
C	-0.080337000	-3.065168000	-0.111462000
F	-2.075173000	0.168674000	-1.804082000
F	-3.288055000	1.411758000	-0.537706000

F	-3.521252000	-0.734948000	-0.489247000
H	3.971152000	0.393492000	-2.647765000
H	5.206224000	-0.822761000	-0.849368000
H	4.272026000	-0.894439000	1.461160000
H	2.153145000	0.230727000	1.990878000
H	1.839475000	1.540864000	-2.134084000
H	0.124112000	2.164576000	-0.636668000
H	-0.268401000	2.766073000	1.846195000
H	0.317201000	1.144633000	2.309443000
H	0.980208000	-2.871260000	0.099374000
H	-0.181418000	-3.603513000	-1.062100000
H	-0.508622000	-3.664738000	0.704875000

TS-2*trans*

C	2.732936000	-0.882474000	-2.839461000
C	3.501028000	0.288554000	-2.855100000
C	2.941632000	1.489199000	-2.406626000
C	1.632293000	1.530754000	-1.945744000
C	0.835383000	0.355124000	-1.918477000
C	1.425263000	-0.853128000	-2.382114000
C	-0.491406000	0.324234000	-1.408991000
C	-1.304949000	1.501876000	-0.957866000
C	-0.943150000	0.458055000	1.039889000
O	-1.827564000	1.171554000	0.339804000
C	0.390369000	0.994693000	1.342182000
C	-1.492623000	-0.750071000	1.760406000
O	0.724089000	2.134869000	1.106913000
O	1.183810000	0.072958000	1.882994000
C	2.507137000	0.479061000	2.210467000
F	-0.844957000	-1.869174000	1.416103000
F	-2.778429000	-0.927576000	1.469749000
F	-1.390792000	-0.634928000	3.091669000
H	3.164343000	-1.823297000	-3.189218000
H	4.531445000	0.264404000	-3.216688000
H	3.537129000	2.405116000	-2.417342000
H	1.222086000	2.477029000	-1.591748000
H	0.829671000	-1.769590000	-2.367871000
H	-1.036168000	-0.624047000	-1.468476000
H	-2.179057000	1.674696000	-1.604902000
H	-0.726800000	2.432983000	-0.898331000
H	3.041591000	0.821882000	1.312950000
H	3.003326000	-0.405223000	2.625268000
H	2.496317000	1.286468000	2.956895000

TS-2*cis*

C	-3.556762000	-0.650904000	-0.367383000
C	-4.077914000	-0.262001000	0.870722000
C	-3.443877000	0.746796000	1.604725000
C	-2.303739000	1.360183000	1.107209000
C	-1.758813000	0.981519000	-0.146220000
C	-2.415392000	-0.043722000	-0.873701000
C	-0.564278000	1.609973000	-0.613700000
C	0.044357000	1.344565000	-1.967642000
C	1.161809000	-0.125768000	-0.647797000
O	0.720430000	0.080010000	-1.894544000
C	2.186857000	0.751473000	-0.059747000
C	0.880275000	-1.495359000	-0.071404000
O	2.682360000	1.691318000	-0.638922000
O	2.484792000	0.392581000	1.187578000
C	3.463764000	1.170557000	1.865119000
F	0.053637000	-2.178646000	-0.859152000
F	0.314019000	-1.414270000	1.135398000
F	1.996215000	-2.226444000	0.065893000
H	-4.047595000	-1.440487000	-0.941078000
H	-4.975747000	-0.744364000	1.263659000
H	-3.847051000	1.054024000	2.572533000
H	-1.809365000	2.147191000	1.682664000
H	-2.014120000	-0.373950000	-1.833677000
H	-0.177590000	2.454703000	-0.037014000
H	0.757466000	2.135114000	-2.236347000
H	-0.702849000	1.255375000	-2.770642000
H	3.116712000	2.206152000	1.994996000
H	4.413563000	1.175165000	1.311701000
H	3.602926000	0.699364000	2.844413000

4a

C	-3.392463000	-0.419963000	-1.538125000
C	-4.114597000	-0.053403000	-0.402563000
C	-3.508351000	-0.121433000	0.851742000
C	-2.188314000	-0.554021000	0.970367000
C	-1.454938000	-0.924556000	-0.164301000
C	-2.072470000	-0.851189000	-1.417874000
C	-0.012802000	-1.345912000	-0.070444000
C	0.483755000	-2.091639000	1.181454000
C	1.020245000	-0.273633000	0.400554000
O	1.356578000	-1.010872000	1.546900000
C	0.451271000	1.103614000	0.752829000
C	2.240399000	-0.152288000	-0.524882000
O	0.298393000	1.494373000	1.876124000
O	0.124294000	1.770349000	-0.342294000
C	-0.507562000	3.035307000	-0.163806000
F	2.865876000	-1.326511000	-0.627483000
F	1.905868000	0.229643000	-1.755931000
F	3.120428000	0.731667000	-0.043914000
H	-3.859499000	-0.370811000	-2.524552000
H	-5.149731000	0.283687000	-0.494343000
H	-4.065866000	0.163392000	1.747035000
H	-1.727448000	-0.589014000	1.960353000
H	-1.509463000	-1.132441000	-2.311746000
H	0.292768000	-1.824740000	-1.009124000
H	1.042135000	-3.016678000	0.972635000
H	-0.270333000	-2.288484000	1.958320000
H	-1.466285000	2.912250000	0.360122000
H	0.137655000	3.715548000	0.409271000
H	-0.678222000	3.435566000	-1.168897000

4b

C	2.609865000	-1.099105000	-2.112811000
C	3.245485000	-0.018066000	-2.726439000
C	2.635465000	1.234265000	-2.716455000
C	1.398890000	1.410405000	-2.093309000
C	0.753469000	0.333609000	-1.479482000
C	1.373373000	-0.923447000	-1.498874000
C	-0.575938000	0.465423000	-0.784626000
C	-1.409824000	1.755670000	-0.799559000
C	-0.667713000	0.649008000	0.761944000
O	-1.585295000	1.704845000	0.626715000
C	-1.268423000	-0.537557000	1.518004000
C	0.594475000	1.162578000	1.473747000
O	-2.353452000	-0.530672000	2.026537000
O	-0.440166000	-1.573197000	1.494049000
C	-0.871563000	-2.764130000	2.149584000
F	0.318717000	1.412471000	2.759843000
F	1.018329000	2.303505000	0.928385000
F	1.602486000	0.296274000	1.438743000
H	3.079699000	-2.085433000	-2.115463000
H	4.214100000	-0.153901000	-3.213191000
H	3.123736000	2.085919000	-3.195854000
H	0.941686000	2.401307000	-2.094798000
H	0.879596000	-1.770146000	-1.015284000
H	-1.203403000	-0.393546000	-1.060987000
H	-2.369367000	1.700669000	-1.333689000
H	-0.875843000	2.668998000	-1.101468000
H	-1.069642000	-2.571084000	3.213106000
H	-0.052100000	-3.483263000	2.045142000
H	-1.781354000	-3.158263000	1.675068000

4a’

C	3.425648000	1.520407000	0.751356000
C	4.590356000	0.962368000	0.224584000
C	4.544098000	-0.296644000	-0.376504000
C	3.338775000	-0.992677000	-0.448070000
C	2.166625000	-0.432024000	0.070870000
C	2.216822000	0.828550000	0.673316000
C	0.863135000	-1.169959000	-0.070542000
C	-0.013226000	-0.826538000	-1.296287000
C	-1.041797000	-0.400835000	-0.236262000
O	-0.162486000	-0.696922000	0.825546000
C	-1.492442000	1.060091000	-0.312005000
C	-2.279421000	-1.304009000	-0.157421000
O	-2.044217000	1.512972000	-1.279995000
O	-1.199811000	1.734212000	0.783919000
C	-1.567838000	3.112093000	0.804530000
F	-1.924907000	-2.577782000	0.013397000
F	-3.013265000	-1.232450000	-1.266007000
F	-3.057072000	-0.958818000	0.872933000
H	3.457361000	2.502523000	1.229328000
H	5.536237000	1.505650000	0.286714000
H	5.454062000	-0.742413000	-0.785243000
H	3.307882000	-1.983500000	-0.910224000
H	1.301974000	1.257426000	1.087250000
H	1.019851000	-2.252513000	0.059792000
H	-0.304773000	-1.670781000	-1.931642000
H	0.356718000	-0.001831000	-1.916486000
H	-1.250208000	3.496812000	1.779513000
H	-2.655220000	3.223435000	0.691258000
H	-1.060067000	3.660157000	-0.001657000

4b’

C	-3.399838000	-1.905395000	-0.442246000
C	-3.478641000	-2.970130000	0.454337000
C	-2.340339000	-3.367015000	1.158917000

C	-1.131701000	-2.702608000	0.963088000
C	-1.051896000	-1.625830000	0.072163000
C	-2.192199000	-1.232027000	-0.631813000
C	0.255531000	-0.901219000	-0.084151000
C	0.571610000	0.252861000	0.891781000
C	0.781132000	1.058823000	-0.403350000
O	0.301055000	0.004134000	-1.202922000
C	2.253661000	1.356886000	-0.715048000
C	-0.096648000	2.308030000	-0.558826000
O	2.918512000	0.695957000	-1.462587000
O	2.691073000	2.393664000	-0.016916000
C	4.058401000	2.760002000	-0.189883000
F	0.101488000	3.177347000	0.429692000
F	-1.389601000	1.983858000	-0.549772000
F	0.153273000	2.930813000	-1.715474000
H	-4.285485000	-1.594343000	-1.001599000
H	-4.425470000	-3.494847000	0.602183000
H	-2.393113000	-4.204305000	1.858935000
H	-0.238690000	-3.023655000	1.507316000
H	-2.126779000	-0.399018000	-1.332954000
H	1.081561000	-1.628360000	-0.154519000
H	1.448094000	0.133708000	1.538909000
H	-0.291632000	0.585125000	1.481584000
H	4.224136000	3.630996000	0.453128000
H	4.720725000	1.936610000	0.112713000
H	4.260055000	3.020908000	-1.238264000

Me-styrene

C	-1.013823000	-0.304963000	-0.013015000
C	-0.975112000	-1.696072000	0.011718000
C	0.248144000	-2.370328000	0.013669000
C	1.433220000	-1.636724000	-0.010807000
C	1.392935000	-0.243313000	-0.035291000
C	0.171949000	0.450130000	-0.033341000
C	0.190581000	1.926444000	-0.053710000
C	-0.863064000	2.748651000	0.042627000
H	-1.983307000	0.198331000	-0.021064000
H	-1.910298000	-2.261301000	0.026785000
H	0.274980000	-3.462418000	0.031777000
H	2.397241000	-2.151326000	-0.011630000
H	2.327574000	0.324303000	-0.054162000
H	1.188372000	2.371964000	-0.151746000
H	-1.869354000	2.326468000	0.151825000
C	-0.784646000	4.241240000	0.017881000
H	-1.202806000	4.672410000	0.942839000
H	0.250461000	4.597538000	-0.091743000
H	-1.381456000	4.652400000	-0.813433000

^{3}[Me-styrene]

C	-1.008847000	-0.273721000	0.087979000
C	-0.996745000	-1.659290000	0.159458000
C	0.212355000	-2.363813000	0.106219000
C	1.417038000	-1.659484000	-0.019934000
C	1.417371000	-0.274813000	-0.092742000
C	0.201560000	0.463713000	-0.041214000
C	0.208330000	1.882515000	-0.117647000
C	-0.989528000	2.722350000	-0.086078000
H	-1.956398000	0.270333000	0.132957000
H	-1.939283000	-2.203638000	0.258862000
H	0.216394000	-3.454737000	0.162475000
H	2.363727000	-2.204029000	-0.061899000
H	2.361612000	0.267751000	-0.191732000
H	1.189135000	2.370144000	-0.224644000
H	-1.305118000	3.159445000	0.870415000
C	-1.677124000	3.150264000	-1.340015000
H	-1.042278000	3.831209000	-1.941409000
H	-1.898066000	2.287909000	-1.994268000
H	-2.619735000	3.676786000	-1.130573000

TS-3$_{trans}$

C	-3.034757000	0.426417000	-1.673227000
C	-4.011596000	0.271738000	-0.679390000
C	-3.736529000	-0.500110000	0.456575000
C	-2.500156000	-1.107385000	0.608282000
C	-1.495628000	-0.971151000	-0.392327000
C	-1.798964000	-0.182815000	-1.538965000
C	-0.217759000	-1.562072000	-0.246868000
C	0.188913000	-2.392991000	0.885024000
C	1.549621000	0.360948000	0.540717000
O	1.941634000	-0.351783000	1.435077000
C	0.381101000	1.319890000	0.666156000
C	2.382908000	0.486405000	-0.757321000
O	0.144990000	2.178465000	-0.147834000
O	-0.304918000	1.101229000	1.772494000
C	-1.449031000	1.923771000	1.979083000
F	3.226618000	-0.537217000	-0.847737000
F	1.648976000	0.504368000	-1.870356000
F	3.102822000	1.611171000	-0.727454000
H	-3.248334000	1.031504000	-2.557168000
H	-4.984686000	0.755543000	-0.789718000
H	-4.497361000	-0.617621000	1.231743000
H	-2.286467000	-1.698360000	1.501403000
H	-1.035173000	-0.054828000	-2.309049000
H	0.462507000	-1.506137000	-1.105190000
H	0.096636000	-1.982271000	1.894625000
H	-2.113255000	1.895824000	1.103826000
H	-1.963800000	1.518869000	2.857016000
H	-1.145450000	2.963526000	2.170237000
C	0.877134000	-3.690432000	0.668348000
H	0.968146000	-4.267859000	1.598702000
H	0.361029000	-4.309528000	-0.086216000
H	1.901034000	-3.521893000	0.278536000

TS-3$_{cis}$

C	-2.554857000	-0.027754000	-1.851995000
C	-3.508380000	0.540235000	-0.994751000
C	-3.349037000	0.439906000	0.393230000
C	-2.249999000	-0.212483000	0.927876000
C	-1.268655000	-0.795508000	0.076395000
C	-1.454824000	-0.685666000	-1.331905000
C	-0.113149000	-1.427571000	0.590576000
C	0.157006000	-1.679039000	2.004815000
C	1.796038000	0.498567000	0.583681000
O	2.280008000	0.002602000	1.574981000
C	2.157626000	0.076856000	-0.830062000
C	0.952021000	1.787321000	0.728043000
O	1.837854000	0.695411000	-1.814404000
O	2.893031000	-1.022997000	-0.825350000
C	3.309129000	-1.511683000	-2.096484000
F	0.417243000	1.834731000	1.946588000
F	1.757353000	2.850103000	0.594953000
F	-0.030519000	1.915865000	-0.153113000
H	-2.679076000	0.052868000	-2.934225000
H	-4.373036000	1.063122000	-1.409488000
H	-4.092161000	0.884507000	1.059186000
H	-2.124477000	-0.276700000	2.010850000
H	-0.705618000	-1.118448000	-1.999479000
H	0.555060000	-1.910201000	-0.133327000
H	0.145005000	-0.841038000	2.707040000
H	2.439067000	-1.773119000	-2.716585000
H	3.915524000	-0.761379000	-2.623185000
H	3.908896000	-2.406579000	-1.898460000
C	0.541352000	-3.035038000	2.474175000
H	-0.139464000	-3.812900000	2.084947000
H	1.552654000	-3.300163000	2.107388000
H	0.557784000	-3.098605000	3.570916000

Int-3

C	-3.954727000	0.151035000	-2.035789000
C	-4.309918000	1.421545000	-1.565769000
C	-3.706198000	1.921858000	-0.405839000
C	-2.762040000	1.173271000	0.281435000
C	-2.384034000	-0.120977000	-0.175986000
C	-3.013531000	-0.607290000	-1.358117000
C	-1.428067000	-0.934379000	0.479635000
C	-0.691678000	-0.605790000	1.734706000
C	1.385618000	-0.308848000	0.523656000
O	0.729902000	-0.875452000	1.516552000
C	1.107544000	1.043275000	0.041780000
C	2.490444000	-1.155561000	-0.056477000
O	0.443859000	1.861362000	0.643265000
O	1.685678000	1.272308000	-1.136818000
C	1.526966000	2.575066000	-1.684574000
F	3.640371000	-0.482280000	-0.163285000
F	2.718028000	-2.223240000	0.705409000
F	2.183672000	-1.602775000	-1.283616000
H	-4.420201000	-0.246278000	-2.941036000
H	-5.052091000	2.018435000	-2.100535000
H	-3.978384000	2.913633000	-0.036601000
H	-2.298179000	1.592000000	1.175621000
H	-2.739649000	-1.598156000	-1.729900000
H	-1.212452000	-1.920581000	0.053487000
H	-0.789825000	0.453370000	1.999045000
H	0.464806000	2.794206000	-1.865763000
H	1.944152000	3.337317000	-1.010692000
H	2.075798000	2.575876000	-2.632682000
C	-1.089417000	-1.477824000	2.910285000
H	-0.469418000	-1.244928000	3.788500000
H	-2.141140000	-1.285159000	3.165494000
H	-0.975691000	-2.544606000	2.667117000

Int-4

C	-3.968194000	-0.665333000	-1.625082000
C	-4.703068000	-0.344877000	-0.476319000
C	-4.083516000	-0.401965000	0.777581000
C	-2.751545000	-0.772028000	0.893601000
C	-1.982661000	-1.099712000	-0.259097000
C	-2.637218000	-1.036495000	-1.523257000
C	-0.616794000	-1.465886000	-0.204882000
C	0.201156000	-1.640798000	1.034778000
C	1.582397000	0.192408000	0.350252000
O	1.511634000	-1.045303000	0.803419000
C	0.650144000	1.248175000	0.750160000
C	2.752911000	0.459867000	-0.562226000
O	-0.084370000	1.182108000	1.712799000
O	0.718182000	2.297363000	-0.068326000
C	-0.105333000	3.412220000	0.251399000
F	3.607182000	-0.560632000	-0.548307000
F	2.361984000	0.632059000	-1.833960000
F	3.428758000	1.557732000	-0.209738000
H	-4.445275000	-0.623150000	-2.607191000
H	-5.752325000	-0.052567000	-0.558142000
H	-4.652230000	-0.152471000	1.676623000
H	-2.292790000	-0.803523000	1.882832000
H	-2.067984000	-1.284285000	-2.423014000
H	-0.108042000	-1.712439000	-1.143536000
H	-0.249069000	-1.123781000	1.890792000
H	-1.167304000	3.127936000	0.238431000
H	0.149079000	3.815559000	1.242086000
H	0.090291000	4.165519000	-0.519607000
C	0.461314000	-3.094530000	1.383284000
H	1.114509000	-3.167382000	2.265412000
H	-0.492689000	-3.588994000	1.615639000
H	0.935898000	-3.622747000	0.543175000

TS-4*$_{trans}$*

C	-3.495911000	-0.517637000	-1.665144000
C	-4.260539000	-0.167344000	-0.544903000
C	-3.725331000	-0.332651000	0.736681000
C	-2.442392000	-0.835121000	0.908637000
C	-1.646717000	-1.193537000	-0.213080000
C	-2.215917000	-1.023492000	-1.506282000
C	-0.309784000	-1.657453000	-0.103662000
C	0.447985000	-1.923197000	1.169405000
C	1.599669000	-0.087958000	0.366347000
O	1.721003000	-1.239200000	1.021964000
C	0.666410000	0.953594000	0.811488000
C	2.631321000	0.172197000	-0.703996000
O	0.105276000	0.941043000	1.884912000
O	0.502448000	1.897417000	-0.113591000
C	-0.399120000	2.951337000	0.202227000
F	3.530057000	-0.808045000	-0.740785000
F	2.076157000	0.251063000	-1.921300000
F	3.290324000	1.319847000	-0.502825000
H	-3.908779000	-0.392204000	-2.668876000
H	-5.270063000	0.230247000	-0.671171000
H	-4.319164000	-0.063679000	1.613464000
H	-2.046660000	-0.948729000	1.918347000
H	-1.620776000	-1.291654000	-2.383134000
H	0.200493000	-1.969939000	-1.021334000
H	-0.063328000	-1.490838000	2.039570000
H	-1.421129000	2.563153000	0.322425000
H	-0.095046000	3.466508000	1.124569000
H	-0.363510000	3.647763000	-0.642768000
C	0.740367000	-3.388760000	1.416612000
H	1.352096000	-3.511810000	2.322389000
H	-0.205152000	-3.931365000	1.561722000
H	1.275024000	-3.832183000	0.563506000

TS-4*$_{cis}$*

C	-4.156972000	-0.456841000	-0.363237000
C	-4.623922000	-0.427834000	0.955518000
C	-3.846161000	0.176662000	1.950851000
C	-2.622026000	0.743440000	1.633775000
C	-2.123977000	0.725164000	0.300532000
C	-2.931972000	0.105498000	-0.692467000
C	-0.853625000	1.294919000	0.028871000
C	-0.225403000	1.454102000	-1.324675000
C	1.401458000	0.045757000	-0.353136000
O	1.156486000	1.018329000	-1.222416000
C	2.384771000	0.335353000	0.701486000
C	0.880032000	-1.355339000	-0.592093000
O	2.838124000	1.436478000	0.905085000
O	2.688881000	-0.751636000	1.410765000
C	3.597795000	-0.579313000	2.490199000
F	1.869615000	-2.206431000	-0.896345000
F	0.022613000	-1.378213000	-1.617087000
F	0.248796000	-1.852323000	0.472594000
H	-4.759446000	-0.928201000	-1.143383000
H	-5.588493000	-0.874469000	1.206742000
H	-4.204218000	0.202311000	2.982732000
H	-2.018056000	1.213008000	2.414430000
H	-2.590822000	0.064776000	-1.728070000
H	-0.329770000	1.797403000	0.849256000
H	-0.721676000	0.824920000	-2.075934000
H	4.569981000	-0.214123000	2.128765000
H	3.717206000	-1.566774000	2.949669000
H	3.198405000	0.131575000	3.228196000
C	-0.201999000	2.891244000	-1.808302000
H	0.318802000	2.963657000	-2.774446000
H	0.310173000	3.536880000	-1.080039000
H	-1.232456000	3.251726000	-1.941071000

11a

C	-3.281606000	-0.439706000	-1.632215000
C	-4.044266000	-0.083191000	-0.519747000
C	-3.480227000	-0.154447000	0.753409000
C	-2.160901000	-0.577487000	0.915284000
C	-1.387282000	-0.938752000	-0.194953000
C	-1.964710000	-0.863989000	-1.468586000
C	0.056255000	-1.341840000	-0.071395000
C	0.612416000	-2.019578000	1.199573000
C	1.119957000	-0.254304000	0.268982000
O	1.645647000	-1.014755000	1.321872000
C	0.523323000	1.066035000	0.768832000
C	2.176186000	-0.031857000	-0.822265000
O	0.400622000	1.338663000	1.930766000
O	0.116932000	1.819179000	-0.240261000
C	-0.571283000	3.022822000	0.087061000
F	2.861440000	-1.153544000	-1.049608000
F	1.629742000	0.339220000	-1.979456000
F	3.055749000	0.907988000	-0.460007000
H	-3.714675000	-0.387760000	-2.633861000
H	-5.077772000	0.248206000	-0.644881000
H	-4.069782000	0.121353000	1.630873000
H	-1.735853000	-0.613947000	1.920493000
H	-1.369122000	-1.136044000	-2.344151000
H	0.356307000	-1.896750000	-0.970775000
H	-0.070201000	-1.953613000	2.062096000
H	-1.496274000	2.796250000	0.636575000
H	0.063507000	3.681852000	0.695585000
H	-0.811627000	3.505999000	-0.865964000
C	1.159034000	-3.415711000	1.063877000
H	1.686000000	-3.714025000	1.982452000
H	0.332151000	-4.122919000	0.896841000
H	1.860337000	-3.479973000	0.219226000

11b

C	2.564911000	-1.116285000	-2.206567000
C	3.241747000	-0.016642000	-2.736586000
C	2.671972000	1.251383000	-2.643428000
C	1.435359000	1.424059000	-2.020142000
C	0.747323000	0.328588000	-1.489906000
C	1.327361000	-0.943200000	-1.593353000
C	-0.580947000	0.455425000	-0.792989000
C	-1.436723000	1.736979000	-0.832131000
C	-0.674993000	0.655054000	0.750171000
O	-1.596748000	1.703137000	0.606246000
C	-1.277463000	-0.535511000	1.498543000
C	0.583681000	1.175452000	1.462089000
O	-2.401802000	-0.567334000	1.912626000
O	-0.407678000	-1.533856000	1.572487000
C	-0.848860000	-2.728704000	2.213266000
F	0.313503000	1.401069000	2.754600000
F	0.985336000	2.332774000	0.933250000
F	1.606787000	0.328580000	1.405621000
H	3.002196000	-2.115142000	-2.274995000
H	4.210992000	-0.149603000	-3.222862000
H	3.192881000	2.118255000	-3.056690000
H	1.013162000	2.427937000	-1.951369000
H	0.801269000	-1.807154000	-1.179327000
H	-1.202858000	-0.411611000	-1.061038000
H	-0.868154000	2.636761000	-1.115724000
H	-1.113205000	-2.530137000	3.261238000
H	-0.008074000	-3.429043000	2.165882000
H	-1.719981000	-3.148903000	1.690930000
C	-2.748904000	1.687259000	-1.568263000
H	-2.564989000	1.641498000	-2.652599000
H	-3.342334000	2.589659000	-1.358153000
H	-3.332339000	0.804511000	-1.26674800

8. NMR description

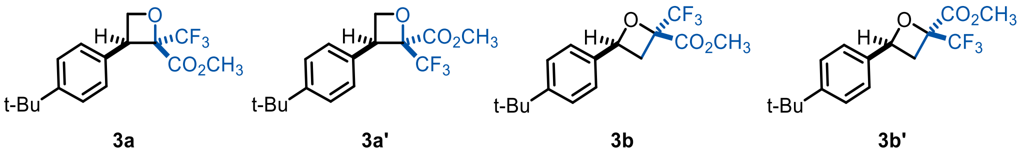

This compound was obtained according to general procedure **1** (1-(tert-butyl)-4-vinylbenzene (183 µL, 1.0 mmol), Ir[dF(CF_3)ppy]$_2$(dtbbpy)PF_6 (5.6 mg, 0.5 mol%), methyl 3,3,3-trifluoro-2-oxopropanoate (153 µL, 1.50 mmol), and dimethyl carbonate (0.8 mL)). After 16 h, the reaction mixture was analysed by NMR (71% yield with **3a**: **3a'**: **3b**: **3b'** = 28%:28%:9%:6% by ^{19}F NMR) and was directly purified by flash column chromatography on silica gel (SiO_2, heptane/EtOAc 100:0 to 90:10) to afford the product as mixture of isomers (221 mg, 70%). The subsequent analytical separation of each isomer was conducted on a Teledyne Isco CombiFlash EZ Prep system. All isomers were isolated as colorless oil.

***Trans*-methyl 3-(4-(tert-butyl)phenyl)-2-(trifluoromethyl)oxetane-2-carboxylate (3a)**
^{1}H NMR (300 MHz, $CDCl_3$): δ 7.43 – 7.36 (m, 2H), 7.30 – 7.22 (m, 2H), 5.08 – 4.95 (m, 2H), 4.46 (dd, J = 8.5, 6.8 Hz, 1H), 3.38 (s, 3H), 1.31 (s, 9H).
^{19}F NMR (282 MHz, $CDCl_3$): δ -78.87 (s, 3F).
^{13}C NMR (75 MHz, $CDCl_3$): δ 165.4, 151.8, 131.4, 127.6, 125.8, 123.3 (q, J = 283.9 Hz), 88.7 (d, J = 31.6 Hz), 73.7, 52.6, 34.7, 31.3.

***Cis*-methyl 3-(4-(tert-butyl)phenyl)-2-(trifluoromethyl)oxetane-2-carboxylate (3a')**
^{1}H NMR (300 MHz, $CDCl_3$): δ 7.43 – 7.36 (m, 2H), 7.27 (d, J = 8.2 Hz, 2H), 5.34 – 5.22 (m, 1H), 4.85 – 4.72 (m, 2H), 3.96 (s, 3H), 1.32 (s, 9H).
^{19}F NMR (282 MHz, $CDCl_3$): δ -74.23 (s, 3F).
^{13}C NMR (75 MHz, $CDCl_3$): δ 167.9, 151.3, 129.4, 128.0, 125.5, 122.8 (q, J = 286.4 Hz), 87.3 (q, J = 29.5 Hz), 71.5, 53.6, 45.0, 34.7, 31.4.

***Trans*-methyl 4-(4-(tert-butyl)phenyl)-2-(trifluoromethyl)oxetane-2-carboxylate (3b)**
^{1}H NMR (300 MHz, $CDCl_3$): δ 7.46 – 7.34 (m, 4H), 5.78 (t, J = 7.4 Hz, 1H), 3.92 (s, 3H), 3.33 (dd, J = 12.5, 7.6 Hz, 1H), 3.07 (ddd, J = 12.6, 7.4, 0.9 Hz, 1H), 1.32 (s, 9H).
^{19}F NMR (282 MHz, $CDCl_3$): δ -79.82 (s, 3F).
^{13}C NMR (75 MHz, $CDCl_3$): δ 167.5, 152.3, 137.3, 125.9, 125.8, 123.9 (q, J = 284.7 Hz), 79.7, 79.7 (d, J = 33.1 Hz), 53.5, 34.9, 34.8, 31.4.

***Cis*-methyl 4-(4-(tert-butyl)phenyl)-2-(trifluoromethyl)oxetane-2-carboxylate (3b')**
^{1}H NMR (300 MHz, $CDCl_3$): δ 7.47 – 7.33 (m, 4H), 5.86 (t, J = 7.7 Hz, 1H), 3.96 (s, 3H), 3.19 (qd, J = 12.1, 7.8 Hz, 2H), 1.33 (s, 9H).
^{19}F NMR (282 MHz, $CDCl_3$): δ -78.59 (s, 3F).
^{13}C NMR (75 MHz, $CDCl_3$): δ 168.2, 152.2, 136.6, 125.7, 125.7, 122.6 (d, J = 282.7 Hz), 79.3, 79.3 (d, J = 67.1 Hz), 53.6, 34.8, 34.4, 34.4, 31.4.

HRMS: (ESI) m/z, $[M]^+$ calcd for $C_{16}H_{19}F_3O_3$ $[M]^+$: 316.1281; found: 316.1282.

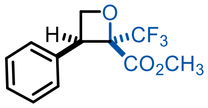

4a

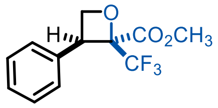

4a'

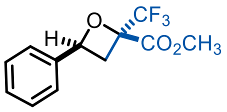

4b

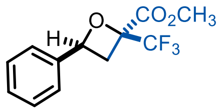

4b'

This compound was obtained according to general procedure **1** ((115 µL, 1.0 mmol), Ir[dF(CF_3)ppy]$_2$(dtbbpy)PF_6 (5.6 mg, 0.5 mol%), methyl 3,3,3-trifluoro-2-oxopropanoate (153 µL, 1.50 mmol), and dimethyl carbonate (0.8 mL)). After 16 h, the reaction mixture was analysed by NMR (55% yield with **4a**: **4a'**: **4b**: **4b'** = 18%:18%:14%:5% by ^{19}F NMR) and was directly purified by flash column chromatography on silica gel (SiO_2, heptane/EtOAc 100:0 to 90:10) to afford the product as mixture of isomers (130 mg, 50%). The subsequent analytical separation of isomers was conducted on a Teledyne Isco CombiFlash EZ Prep system. All isomers were isolated as colorless oil.

***Trans*-methyl 3-phenyl-2-(trifluoromethyl)oxetane-2-carboxylate (4b)**

^{1}H NMR (300 MHz, $CDCl_3$): δ 7.46 – 7.34 (m, 5H), 5.81 (t, J = 7.5 Hz, 1H), 3.92 (s, 3H), 3.36 (dd, J = 12.6, 7.7 Hz, 1H), 3.04 (dd, J = 12.6, 7.3 Hz, 1H).

^{19}F NMR (282 MHz, $CDCl_3$): δ -79.85 (s, 3F).

^{13}C NMR (75 MHz, $CDCl_3$): δ 167.3, 140.2, 128.9, 128.8, 125.7,123.7 (d, J = 284.5 Hz), 79.6, 79.6 (d, J = 33.0 Hz), 53.4, 34.8, 29.7.

Mixture of other 3 isomers: *trans*-methyl-3-phenyl-2-(trifluoromethyl)oxetane-2-carboxylate (4a); *cis*-methyl-4-phenyl-2-(trifluoromethyl)oxetane-2-carboxylate (4a') and *cis*-methyl-4-phenyl-2-(trifluoromethyl)-oxetane-2-carboxylate (4b')

^{1}H NMR (300 MHz, $CDCl_3$): δ 7.47 – 7.29 (m, 15H), 5.89 (t, J = 7.7 Hz, 1H), 5.34 – 5.25 (m, 1H), 5.09 – 4.97 (m, 2H), 4.83 (d, J = 3.9 Hz, 2H), 4.49 (dd, J = 8.4, 6.7 Hz, 1H), 3.96 (d, J = 2.5 Hz, 6H), 3.39 (s, 2H), 3.26 (dd, J = 12.1, 7.6 Hz, 1H), 3.11 (dd, J = 12.1, 7.9 Hz, 1H).

^{19}F NMR (282 MHz, $CDCl_3$): δ -74.31 (s, 3F), -78.68 (s, 3F), -78.91 (s, 3F).

^{13}C NMR (75 MHz, $CDCl_3$): δ 168.0, 167.8, 165.3, 139.7, 134.4, 132.5, 128.9, 128.8, 128.7, 128.6, 128.2, 128.2, 128.1, 127.8, 125.5, 123.3 (d, J = 284.0 Hz), 122.7 (d, J = 286.5 Hz), 122.5 (d, J = 282.7 Hz), 88.6 (d, J = 31.6 Hz), 87.2 (d, J = 29.6 Hz), 79.6 (d, J = 33.5 Hz), 79.2, 73.5, 71.2, 53.6, 53.6, 52.6, 45.1, 44.0, 34.3.

HRMS: (ESI) m/z, $[M]^+$ calcd for $C_{12}H_{11}F_3O_3Na$ $[M+Na]^+$: 283.0553; found: 283.0549.

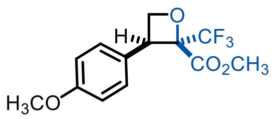

5a

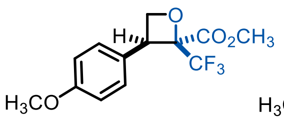

5a'

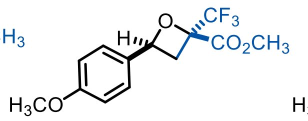

5b

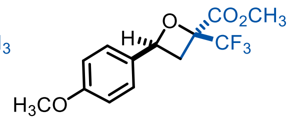

5b'

This compound was obtained according to general procedure **1** (1-methoxy-4-vinylbenzene (133 µL, 1.0 mmol), Ir[dF(CF_3)ppy]$_2$(dtbbpy)PF_6 (5.6 mg, 0.5 mol%), methyl 3,3,3-trifluoro-2-oxopropanoate (153 µL, 1.50 mmol), and dimethyl carbonate (0.8 mL)). After 16 h, the reaction mixture was analyzed by NMR (32% yield with **5a**:**5a'**:**5b**:**5b'** = 16:16:n.d:n.d. by ^{19}F NMR), and was directly purified by flash column chromatography on silica gel (SiO_2, heptane/EtOAc 100:0 to 90:10) to afford the product as a mixture of isomers (58 mg, 20%). The subsequent analytical separation of each isomer was conducted on a Teledyne Isco CombiFlash EZ Prep system. **5a** and

5a’ were isolated as yellowish oil.

***Trans*-methyl-3-(4-methoxyphenyl)-2-(trifluoromethyl)oxetane-2-carboxylate (5a)**

^{1}H NMR (300 MHz, $CDCl_3$): δ 7.27 – 7.23 (m, 2H), 6.92 – 6.87 (m, 2H), 5.04 (dd, *J* = 8.5, 6.2 Hz, 1H), 4.97 (t, *J* = 6.5 Hz, 1H), 4.44 (dd, *J* = 8.5, 6.8 Hz, 1H), 3.81 (s, 3H), 3.45 (s, 3H).

^{19}F NMR (282 MHz, $CDCl_3$): δ -78.84 (s, 3F).

^{13}C NMR (101 MHz, $CDCl_3$): δ 165.4, 159.8, 129.1, 126.4, 123.3 (q, *J* = 284.0 Hz), 114.3, 88.9 (q, *J* = 31.4 Hz), 74.0, 55.4, 52.8, 43.6.

***Cis*-methyl-3-(4-methoxyphenyl)-2-(trifluoromethyl)oxetane-2-carboxylate (5a’)**

^{1}H NMR (300 MHz, $CDCl_3$): δ 7.27 (d, *J* = 8.9 Hz, 2H), 6.94 – 6.88 (m, 2H), 5.25 (dd, *J* = 8.3, 5.2 Hz, 1H), 4.85 – 4.70 (m, 2H), 3.96 (s, 3H), 3.81 (s, 3H).

^{19}F NMR (282 MHz, $CDCl_3$): δ -74.31 (s, 3F).

^{13}C NMR (75 MHz, $CDCl_3$): δ 168.0, 159.5, 129.5, 124.3, 122.8 (d, *J* = 286.5 Hz), 114.1, 87.3 (d, *J* = 29.3 Hz), 71.6, 55.4, 53.6, 44.8.

HRMS: (ESI) m/z, $[M]^+$ calcd for $C_{13}H_{13}O_4F_3Na$ $[M+Na]^+$: 313.0658; found: 313.0656.

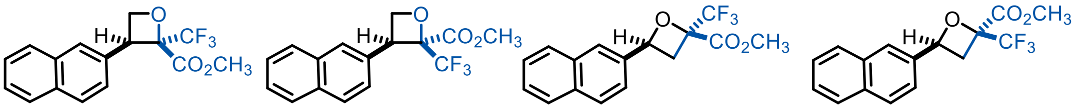

6a **6a'** **6b** **6b'**

This compound was obtained according to general procedure **1** (2-vinylnaphthalene (154 mg, 1.0 mmol), $Ir[dF(CF_3)ppy]_2(dtbbpy)PF_6$ (5.6 mg, 0.5 mol%), methyl 3,3,3-trifluoro-2-oxopropanoate (153 µL, 1.50 mmol), and dimethyl carbonate (0.8 mL)). After 16 h, the reaction mixture was analysed by NMR (58% yield with **6a**: **6a’**: **6b**: **6b’** = 22:22:11:3 by ^{19}F NMR) and was directly purified by flash column chromatography on silica gel (SiO_2, heptane/EtOAc 100:0 to 90:10) to afford the product as mixture of isomers (168 mg, 54%). The subsequent analytical separation of each isomer was conducted on a Teledyne Isco CombiFlash EZ Prep system. All isomers were isolated as colorless oil.

Trans-methyl-3-(naphthalen-2-yl)-2-(trifluoromethyl)oxetane-2-carboxylate (6a)

^{1}H NMR (300 MHz, $CDCl_3$): δ 7.88 – 7.81 (m, 3H), 7.79 (d, *J* = 1.9 Hz, 1H), 7.55 – 7.49 (m, 2H), 7.44 (dd, *J* = 8.5, 1.9 Hz, 1H), 5.14 (d, *J* = 7.5 Hz, 2H), 4.66 (t, *J* = 7.5 Hz, 1H), 3.32 (s, 3H).

^{19}F NMR (282 MHz, $CDCl_3$): δ -78.84 (s, 3F).

^{13}C NMR (101 MHz, $CDCl_3$): δ 165.3, 133.3, 133.2, 131.8, 128.8, 128.1, 127.8, 127.2, 126.8, 126.7, 125.3, 123.3 (q, *J* = 284.2 Hz), 88.6 (q, *J* = 31.6 Hz), 73.7, 52.7, 44.3, 44.3.

Cis-methyl-3-(naphthalen-2-yl)-2-(trifluoromethyl)oxetane-2-carboxylate (6a’)

^{1}H NMR (300 MHz, $CDCl_3$): δ 7.90 – 7.81 (m, 3H), 7.75 (s, 1H), 7.56 – 7.45 (m, 3H), 5.45 (dd, *J* = 8.2, 5.1 Hz, 1H), 5.04 – 4.87 (m, 2H), 4.01 (s, 3H).

^{19}F NMR (282 MHz, $CDCl_3$): δ -74.26 (s, 3F).

^{13}C NMR (101 MHz, $CDCl_3$): δ 167.9, 167.9, 133.2, 133.0, 130.1, 128.4, 128.1, 127.8, 127.0, 126.6, 126.6, 126.1, 122.8 (q, *J* = 286.5 Hz), 87.34 (q, *J* = 29.8 Hz), 71.3, 53.7, 45.4.

Trans-methyl-4-(naphthalen-2-yl)-2-(trifluoromethyl)oxetane-2-carboxylate (6b)

^{1}H NMR (300 MHz, $CDCl_3$): δ 7.88 (m, 4H), 7.57 – 7.47 (m, 3H), 5.99 (t, *J* = 7.5 Hz, 1H), 3.94 (s,

3H), 3.48 – 3.38 (m, 1H), 3.11 (ddd, J = 12.5, 7.3, 0.9 Hz, 1H).
^{19}F NMR (282 MHz, $CDCl_3$): δ -79.80 (s, 3F).
^{13}C NMR (101 MHz, $CDCl_3$): δ 167.4, 137.7, 133.6, 133.2, 129.1, 128.4, 127.9, 126.7, 126.7, 125.2, 123.8 (d, J = 285.8 Hz), 123.0, 122.4, 79.8, 79.8 (q, J = 32.9 Hz), 53.6, 34.9.

Cis-methyl-4-(naphthalen-2-yl)-2-(trifluoromethyl)oxetane-2-carboxylate (6b')
^{1}H NMR (300 MHz, $CDCl_3$): δ 7.93 – 7.82 (m, 4H), 7.56 – 7.48 (m, 3H), 6.06 (t, J = 7.7 Hz, 1H), 3.99 (s, 3H), 3.34 (dd, J = 12.1, 7.6 Hz, 1H), 3.20 (dd, J = 12.1, 7.9 Hz, 1H).
^{19}F NMR (282 MHz, $CDCl_3$): δ -78.58 (s, 3F).
^{13}C NMR (75 MHz, $CDCl_3$): δ 168.1, 137.1, 133.5, 133.2, 128.8, 128.4, 127.9, 126.7, 126.6, 124.9, 122.8, 122.6 (d, J = 282.7 Hz), 79.8 (d, J = 33.9 Hz), 79.4, 53.7, 34.4.

HRMS: (ESI) m/z, $[M]^+$ calcd for $C_{16}H_{13}O_3F_3Na$ $[M+Na]^+$: 333.0709; found: 333.0703.

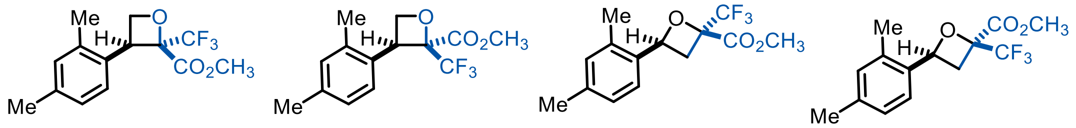

7a **7a'** **7b** **7b'**

This compound was obtained according to general procedure **1** (2,4-dimethyl-1-vinylbenzene (146 μL, 1.0 mmol), $Ir[dF(CF_3)ppy]_2(dtbbpy)PF_6$ (5.6 mg, 0.5 mol%), methyl 3,3,3-trifluoro-2-oxopropanoate (153 μL, 1.50 mmol), and dimethyl carbonate (0.8 mL)). After 16 h, the reaction mixture was analyzed by NMR (67% yield with **7a**: **7a'**: **7b**: **7b'** = 28:28:n.d.:11 by ^{19}F NMR), and was directly purified by flash column chromatography on silica gel (SiO_2, heptane/EtOAc 100:0 to 90:10) to afford the product as mixture of isomers (189 mg, 66%). The subsequent analytical separation of each isomer was conducted on a Teledyne Isco CombiFlash EZ Prep system. All isomers were isolated as colorless oil.

***Trans*-methyl-3-(2,4-dimethylphenyl)-2-(trifluoromethyl)oxetane-2-carboxylate (7a)**
^{1}H NMR (300 MHz, $CDCl_3$): δ 7.43 (d, J = 7.9 Hz, 1H), 7.07 (d, J = 8.6 Hz, 1H), 7.01 (s, 1H), 5.06 (dd, J = 8.4, 6.3 Hz, 1H), 4.95 (t, J = 6.2 Hz, 1H), 4.67 (dd, J = 8.4, 6.1 Hz, 1H), 3.39 (s, 3H), 2.29 (d, J = 7.4 Hz, 6H).
^{19}F NMR (282 MHz, $CDCl_3$): δ -78.57 (s, 3F).
^{13}C NMR (75 MHz, $CDCl_3$): δ 165.3, 138.2, 137.3, 131.6, 129.5, 127.1, 126.1, 123.5 (d, J = 284.9 Hz), 88.7 (d, J = 31.4 Hz), 73.8, 52.6, 39.6, 21.1, 19.5.

***Cis*-methyl-3-(2,4-dimethylphenyl)-2-(trifluoromethyl)oxetane-2-carboxylate (7a')**
^{1}H NMR (300 MHz, $CDCl_3$): δ 7.35 (d, J = 7.9 Hz, 1H), 7.07 (d, J = 7.9 Hz, 1H), 7.03 (s, 1H), 5.36 (dd, J = 7.2, 4.9 Hz, 1H), 4.97 – 4.80 (m, 2H), 3.96 (s, 3H), 2.32 (s, 3H), 2.24 (s, 3H).
^{19}F NMR (282 MHz, $CDCl_3$): δ -74.29 (s, 3F).
^{13}C NMR (75 MHz, $CDCl_3$): δ 167.9, 138.1, 137.8, 131.6, 127.7, 127.6, 122.7 (d, J = 285.5 Hz), 87.6 (d, J = 29.6 Hz) , 72.1, 53.5, 42.3, 21.1, 19.7.

***Cis*-methyl-4-(2,4-dimethylphenyl)-2-(trifluoromethyl)oxetane-2-carboxylate (7b')**
^{1}H NMR (300 MHz, $CDCl_3$): δ 7.53 (d, J = 7.8 Hz, 1H), 7.10 (d, J = 7.8 Hz, 1H), 6.97 (s, 1H), 6.01 (t, J = 7.7 Hz, 1H), 3.97 (s, 3H), 3.31 (dd, J = 12.0, 7.6 Hz, 1H), 2.96 (dd, J = 11.9, 7.8 Hz, 1H),

2.33 (s, 3H), 2.13 (s, 3H).

^{19}F NMR (282 MHz, $CDCl_3$): δ -78.83 (s, 3F).

^{13}C NMR (101 MHz, $CDCl_3$): δ 168.1, 137.9, 135.1, 133.3, 131.0, 127.0, 124.4, 122.5 (q, J = 283.0 Hz), 79.9 (q, J = 33.6 Hz), 77.4, 53.6, 33.6, 21.2, 18.5.

HRMS: (ESI) m/z, $[M]^+$ calcd for $C_{14}H_{15}F_3O_3Na$ $[M+Na]^+$: 311.0866; found: 311.0862.

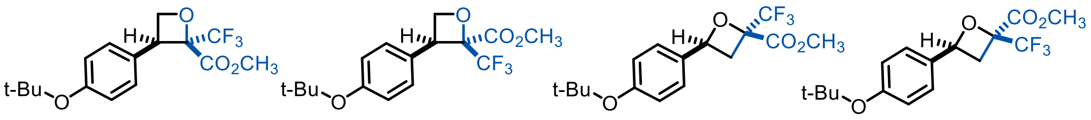

8a **8a'** **8b** **8b'**

This compound was obtained according to general procedure **1** (1-(tert-butoxy)-4-vinylbenzene (188 µL, 1.0 mmol), Ir[dF(CF_3)ppy]$_2$(dtbbpy)PF_6 (5.6 mg, 0.5 mol%), methyl 3,3,3-trifluoro-2-oxopropanoate (153 µL, 1.50 mmol), and dimethyl carbonate (0.8 mL)). After 16 h, the reaction mixture was analyzed by NMR (48% yield with **8a**: **8a': 8b: 8b'** = 24:24:n.d:n.d by ^{19}F NMR), and was directly purified by flash column chromatography on silica gel (SiO_2, heptane/EtOAc 100:0 to 90:10) to afford the product as mixture of isomers (110 mg, 33%). The subsequent analytical separation of each isomer was conducted on a Teledyne Isco CombiFlash EZ Prep system. **8a** and **8a'** were isolated as yellowish oil.

***Trans*-methyl-3-(4-(tert-butoxy)phenyl)-2-(trifluoromethyl)oxetane-2-carboxylate (8a)**

^{1}H NMR (300 MHz, $CDCl_3$): δ 7.26 – 7.20 (m, 2H), 7.02 – 6.96 (m, 2H), 5.07 – 4.94 (m, 2H), 4.45 (dd, J = 8.5, 6.7 Hz, 1H), 3.39 (s, 3H), 1.33 (s, 9H).

^{19}F NMR (282 MHz, $CDCl_3$): δ -78.83;

^{13}C NMR (101 MHz, $CDCl_3$): δ 165.4, 155.8, 129.2, 128.5, 124.5, 123.3 (q, J = 284.1 Hz), 88.7 (q, J = 31.8 Hz), 79.0, 73.8, 52.6, 43.6 (d, J = 1.7 Hz), 29.0.

***Cis*-methyl-3-(4-(tert-butoxy)phenyl)-2-(trifluoromethyl)oxetane-2-carboxylate (8a')**

^{1}H NMR (300 MHz, $CDCl_3$): δ 7.23 (d, J = 8.3 Hz, 2H), 7.02 – 6.96 (m, 2H), 5.25 (dd, J = 7.7, 4.6 Hz, 1H), 4.85 – 4.71 (m, 2H), 3.96 (s, 3H), 1.34 (s, 9H).

^{19}F NMR (282 MHz, $CDCl_3$): δ -74.31;

^{13}C NMR (75 MHz, $CDCl_3$): δ 167.9, 155.5, 128.9 (d, J = 1.4 Hz), 127.1, 124.1, 122.7 (q, J = 286.6 Hz), 87.3 (q, J = 29.3 Hz), 78.9, 71.6, 53.6, 44.9, 29.0.

HRMS: (ESI) m/z, $[M]^+$ calcd for $C_{16}H_{19}O_4F_3Na$ $[M+Na]^+$: 355.1128; found: 355.1123.

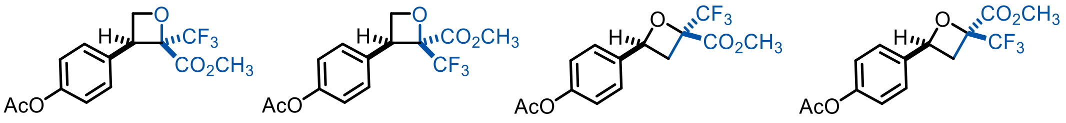

9a **9a'** **9b** **9b'**

This compound was obtained according to general procedure **1** from 4-vinylphenyl acetate (153 µL, 1.0 mmol), Ir[dF(CF_3)ppy]$_2$(dtbbpy)PF_6 (5.6 mg, 0.5 mol%), Methyl 3,3,3-trifluoro-2-oxopropanoate (153 µL, 1.50 mmol), and dimethyl carbonate (0.8 mL). After 16 h, the reaction mixture was analyzed by NMR (51% yield with **9a**: **9a'**: **9b**: **9b'** = 19:19:11:2 by ^{19}F NMR), and was directly purified by flash column chromatography on silica gel (SiO_2, heptane/EtOAc 100:0 to 90:10) to afford the product as mixture of isomers (146 mg, 46%). The subsequent analytical

separation of each isomer was conducted on a Teledyne Isco CombiFlash EZ Prep system. All isomers were isolated as colorless oil.

***Trans*-methyl-3-(4-acetoxyphenyl)-2-(trifluoromethyl)oxetane-2-carboxylate (9a)**

^{1}H NMR (300 MHz, $CDCl_3$): δ 7.39 – 7.30 (m, 2H), 7.15 – 7.06 (m, 2H), 5.11 – 4.92 (m, 2H), 4.48 (dd, J = 8.5, 6.7 Hz, 1H), 3.42 (s, 3H), 2.29 (s, 3H).

^{19}F NMR (282 MHz, $CDCl_3$): δ -78.87 (s, 3F).

^{13}C NMR (75 MHz, $CDCl_3$): δ 169.3, 165.2, 150.9, 132.0, 128.9, 123.2 (q, J = 284.1 Hz), 122.2, 88.5 (q, J = 31.8 Hz), 73.6, 52.8, 43.5 (q, J = 1.7 Hz), 21.2.

***Cis*-methyl-3-(4-acetoxyphenyl)-2-(trifluoromethyl)oxetane-2-carboxylate (9a')**

^{1}H NMR (300 MHz, $CDCl_3$): δ 7.35 (d, J = 8.6 Hz, 2H), 7.14 – 7.08 (m, 2H), 5.31 – 5.18 (m, 1H), 4.87 – 4.73 (m, 2H), 3.96 (s, 3H), 2.29 (s, 3H).

^{19}F NMR (282 MHz, $CDCl_3$): δ -74.30 (s, 3F).

^{13}C NMR (75 MHz, $CDCl_3$): δ 169.3, 167.7, 150.5, 130.0, 129.3, 122.7 (q, J = 286.4 Hz), 121.8, 87.1 (q, J = 29.5 Hz), 71.3, 53.7, 44.6, 21.2.

***Trans*-methyl-4-(4-acetoxyphenyl)-2-(trifluoromethyl)oxetane-2-carboxylate (9b)**

^{1}H NMR (400 MHz, $CDCl_3$): δ 7.52 – 7.44 (m, 2H), 7.17 – 7.09 (m, 2H), 5.80 (t, J = 7.5 Hz, 1H), 3.91 (s, 3H), 3.34 (dd, J = 12.6, 7.7 Hz, 1H), 3.01 (dd, J = 12.2, 6.9 Hz, 1H), 2.31 (s, 3H).

^{19}F NMR (282 MHz, $CDCl_3$): δ -79.81 (s, 3F).

^{13}C NMR (101 MHz, $CDCl_3$): δ 169.5, 167.3, 151.1, 138.0, 127.1, 123.7 (q, J = 284.3 Hz), 122.1, 88.5 (q, J = 31.8 Hz), 79.7 (q, J = 33.0 Hz), 79.2, 53.6, 35.0, 21.2.

***Cis*-methyl-4-(4-acetoxyphenyl)-2-(trifluoromethyl)oxetane-2-carboxylate (9b')**

^{1}H NMR (300 MHz, $CDCl_3$): δ 7.50 – 7.39 (m, 2H), 7.21 – 7.07 (m, 2H), 5.88 (t, J = 7.7 Hz, 1H), 3.96 (s, 3H), 3.25 (dd, J = 12.2, 7.5 Hz, 1H), 3.09 (dd, J = 12.2, 8.0 Hz, 1H), 2.30 (s, 3H).

^{19}F NMR (282 MHz, $CDCl_3$): δ -78.64 (s, 3F).

^{13}C NMR (75 MHz, $CDCl_3$): δ 169.5, 167.9, 151.0, 137.3, 126.9, 122.5 (d, J = 282.7 Hz), 122.0, 79.6 (d, J = 33.6 Hz) ,78.8, 53.7, 34.5, 21.2.

HRMS: (ESI) m/z, $[M]^+$ calcd for $C_{14}H_{13}O_5F_3Na$ $[M+Na]^+$: 341.0607; found: 341.0603.

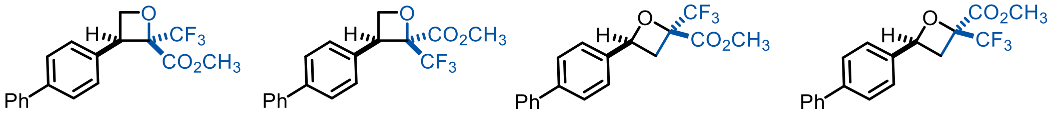

10a **10a'** **10b** **10b'**

This compound was obtained according to general procedure **1** from 4-vinyl-1,1'-biphenyl (180 mg, 1.0 mmol), Ir[dF(CF_3)ppy]$_2$(dtbbpy)PF_6 (5.6 mg, 0.5 mol%), Methyl 3,3,3-trifluoro-2-oxopropanoate (153 µL, 1.50 mmol), and dimethyl carbonate (1.6 mL). After 16 h, the reaction mixture was analysed by NMR (70% yield with **10a**: **10a'**: **10b**: **10b'** = 23:23:16:8 by ^{19}F NMR) and was directly purified by flash column chromatography on silica gel (SiO_2, heptane/EtOAc 100:0 to 90:10) to afford the product as mixture of isomers (225 mg, 67%). The subsequent analytical separation of each isomer was conducted on a Teledyne Isco CombiFlash EZ Prep system. All isomers were isolated as colorless oil.

***Trans*-methyl-3-([1,1'-biphenyl]-4-yl)-2-(trifluoromethyl)oxetane-2-carboxylate (10a)**

^{1}H NMR (300 MHz, $CDCl_3$): δ 7.67 – 7.58 (m, 4H), 7.50 – 7.35 (m, 5H), 5.14 – 5.00 (m, 2H), 4.55 (dd, J = 8.4, 6.7 Hz, 1H), 3.45 (s, 3H).

^{19}F NMR (282 MHz, $CDCl_3$): δ -78.83 (s, 3F).

^{13}C NMR (75 MHz, $CDCl_3$): δ 165.3, 141.4, 140.2, 133.4, 129.0, 128.3, 127.8, 127.5, 127.1, 123.3 (q, J = 284.1 Hz), 88.6 (q, J = 31.6 Hz), 73.6, 52.7, 43.8 (q, J = 1.7 Hz).

***Cis*-methyl-3-([1,1'-biphenyl]-4-yl)-2-(trifluoromethyl)oxetane-2-carboxylate (10a')**

^{1}H NMR (300 MHz, $CDCl_3$): δ 7.64 – 7.60 (m, 4H), 7.49 – 7.34 (m, 5H), 5.40 – 5.28 (m, 1H), 4.87 (d, J = 4.0 Hz, 2H), 4.00 (s, 3H).

^{19}F NMR (282 MHz, $CDCl_3$): δ -74.19 (s, 3F).

^{13}C NMR (75 MHz, $CDCl_3$): δ 167.9, 141.0, 140.4, 131.5, 128.9, 128.6, 127.7, 127.3, 127.2, 122.8 (q, J = 286.5 Hz), 87.3 (q, J = 29.7 Hz), 71.3, 53.7, 45.0.

Trans-methyl-4-([1,1'-biphenyl]-4-yl)-2-(trifluoromethyl)oxetane-2-carboxylate (10b)

^{1}H NMR (300 MHz, $CDCl_3$): δ 7.67 – 7.41 (m, 9H), 7.41 – 7.33 (m, 1H), 5.87 (t, J = 7.5 Hz, 1H), 3.94 (s, 3H), 3.39 (dd, J = 12.6, 7.7 Hz, 1H), 3.09 (ddd, J = 12.6, 7.4, 0.9 Hz, 1H).

^{19}F NMR (282 MHz, $CDCl_3$): δ -79.77 (s, 3F).

^{13}C NMR (75 MHz, $CDCl_3$): δ 167.4, 142.0, 140.6, 139.3, 129.0, 127.7, 127.7, 127.3, 126.4, 123.8 (q, J = 284.4 Hz), 117.8, 79.7 (q, J = 32.9 Hz), 79.5, 53.6, 35.0.

Cis-methyl-4-([1,1'-biphenyl]-4-yl)-2-(trifluoromethyl)oxetane-2-carboxylate (10b')

^{1}H NMR (300 MHz, $CDCl_3$): δ 7.66 – 7.58 (m, 4H), 7.54 – 7.43 (m, 4H), 7.40 – 7.33 (m, 1H), 5.95 (t, J = 7.7 Hz, 1H), 3.98 (s, 3H), 3.30 (dd, J = 12.1, 7.6 Hz, 1H), 3.17 (dd, J = 12.1, 7.9 Hz, 1H).

^{19}F NMR (282 MHz, $CDCl_3$): δ -78.59 (s, 3F).

^{13}C NMR (101 MHz, $CDCl_3$): δ 168.0, 141.9, 140.7, 138.7, 129.0, 127.7, 127.5, 127.3, 126.1, 122.6 (q, J = 282.9 Hz), 79.7 (q, J = 33.6 Hz), 79.1, 53.6, 34.4 (q, J = 1.7 Hz).

HRMS: (EI) m/z, $[M]^+$ calcd for $C_{18}H_{15}O_3F_3Na$ $[M+Na]^+$: 359.0866; found: 359.0859.

Methyl-4-methyl-3-phenyl-2-(trifluoromethyl)oxetane-2-carboxylate (11a).

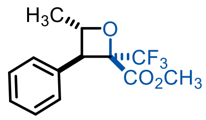

This compound was obtained according to general procedure **2** from (E)-prop-1-en-1-ylbenzene (66 μL, 0.5 mmol), $Ir[dF(CF_3)ppy]_2(dtbbpy)PF_6$ (2.8 mg, 0.5 mol%), Methyl 3,3,3-trifluoro-2-oxopropanoate (77 μL, 0.75 mmol), and dimethyl carbonate (0.4 mL). Isolated as colorless oil (107 mg, 78% yield) after purification by flash column chromatography (24 g SiO_2, heptane/EA 20:1).

Major diastereomer

^{1}H NMR (300 MHz, $CDCl_3$): δ 7.42 – 7.32 (m, 3H), 7.29 – 7.22 (m, 2H), 5.51 – 5.40 (m, 1H), 4.13 (d, J = 7.4 Hz, 1H), 3.44 (s, 3H), 1.62 (d, J = 6.1 Hz, 3H).

^{19}F NMR (282 MHz, $CDCl_3$): δ -77.79 (s, 3F).

^{13}C NMR (75 MHz, $CDCl_3$): δ 165.9, 133.6, 128.9, 128.5, 127.9, 122.7 (q, J = 282.7 Hz), 85.3 (q, J = 31.9 Hz), 80.7, 52.6, 51.0 (d, J = 1.6 Hz), 21.9.

HRMS: (ESI) m/z, $[M]^+$ calcd for $C_{13}H_{13}O_3F_3Na$ $[M+Na]^+$: 297.0709; found: 297.0703.

Methyl-3-(4-methoxyphenyl)-4-methyl-2-(trifluoromethyl)oxetane-2-carboxylate (12a).

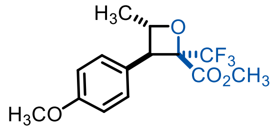

This compound was obtained according to general procedure **2** from (E)-1-methoxy-4-(prop-1-en-1-yl)benzene (75 µL, 0.5 mmol), Ir[dF(CF_3)ppy]$_2$(dtbbpy)PF_6 (2.8 mg, 0.5 mol%), Methyl 3,3,3-trifluoro-2-oxopropanoate (77 µL, 0.75 mmol), and dimethyl carbonate (0.4 mL). Isolated as yellowish oil (64 mg, 42% yield) after purification by flash column chromatography (24 g SiO_2, heptane/EA 20:1).

Major diastereomer

^{1}H NMR (300 MHz, $CDCl_3$): δ 7.19 – 7.13 (m, 2H), 6.91 – 6.84 (m, 2H), 5.36 (p, *J* = 6.2 Hz, 1H), 4.04 (d, *J* = 7.5 Hz, 1H), 3.80 (s, 3H), 3.48 (s, 3H), 1.58 (d, *J* = 6.0 Hz, 3H).

^{19}F NMR (282 MHz, $CDCl_3$): δ -77.73 (s, 3F).

^{13}C NMR (75 MHz, $CDCl_3$): δ 166.1, 159.7, 129.2, 125.6, 122.8 (d, *J* = 282.6 Hz), 114.3, 85.5 (q, *J* = 31.7 Hz), 81.2, 55.4, 52.8, 50.6, 22.0.

HRMS: (ESI) m/z, $[M]^+$ calcd for $C_{14}H_{15}O_4F_3Na$ $[M+Na]^+$: 327.0815; found: 327.0809.

Methyl-3-(4-bromophenyl)-4-methyl-2-(trifluoromethyl)oxetane-2-carboxylate (13a).

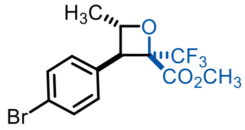

This compound was obtained according to general procedure **2** from (E)-1-bromo-4-(prop-1-en-1-yl)benzene (197 mg, 0.5 mmol), Ir[dF(CF_3)ppy]$_2$(dtbbpy)PF_6 (2.8 mg, 0.5 mol%), Methyl 3,3,3-trifluoro-2-oxopropanoate (77 µL, 0.75 mmol), and dimethyl carbonate (0.4 mL). Isolated as colorless oil (127 mg, 72% yield) after purification by flash column chromatography (24 g SiO_2, heptane/EA 20:1).

^{1}H NMR (300 MHz, $CDCl_3$): δ 7.52 – 7.44 (m, 2H), 7.14 – 7.06 (m, 2H), 5.42 – 5.27 (m, 1H), 4.05 (d, *J* = 7.5 Hz, 1H), 3.48 (s, 3H), 1.58 (d, *J* = 6.2 Hz, 3H).

^{19}F NMR (282 MHz, $CDCl_3$): δ -77.84 (s, 3F).

^{13}C NMR (75 MHz, $CDCl_3$): δ 165.8, 132.7, 132.1, 129.6, 122.7, 122.6 (q, *J* = 282.7 Hz), 85.0 (d, *J* = 32.1 Hz), 80.6, 52.9, 50.4 (q, *J* = 1.6 Hz), 22.0.

HRMS: (ESI) m/z, $[M]^+$ calcd for $C_{13}H_{12}O_3BrF_3Na$ $[M+Na]^+$: 374.9814; found: 374.9807.

Methyl-4-((1,3-dioxoisoindolin-2-yl)methyl)-3-phenyl-2-(trifluoromethyl)oxetane-2-carboxylate (14a).

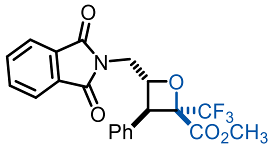

This compound was obtained according to general procedure **2** from 1-(tert-butyl)-4-vinylbenzene (132 mg, 0.5 mmol), Ir[dF(CF_3)ppy]$_2$(dtbbpy)PF_6 (2.8 mg, 0.5 mol%), Methyl 3,3,3-trifluoro-2-oxopropanoate (77 µL, 1.50 mmol), and dimethyl carbonate (1.6 mL). Isolated as white solid (67 mg, 32% yield) after purification by flash column chromatography (24 g SiO_2, heptane/EA 5:1).

^{1}H NMR (300 MHz, $CDCl_3$): δ 7.84 (dd, *J* = 5.5, 3.1 Hz, 2H), 7.71 (dd, *J* = 5.5, 3.1 Hz, 2H), 7.37 – 7.27 (m, 3H), 7.22 (m, 2H), 5.51 (dt, *J* = 7.6, 5.6 Hz, 1H), 4.43 (d, *J* = 7.5 Hz, 1H), 4.30 – 4.05 (m, 2H), 3.41 (s, 3H).

^{19}F NMR (282 MHz, $CDCl_3$): δ -77.51 (s, 3F).

^{13}C NMR (75 MHz, $CDCl_3$): δ 168.1, 165.4, 134.3, 132.8, 131.9, 129.0, 128.7, 127.8, 123.6, 122.5 (q, *J* = 282.9 Hz), 85.8 (q, *J* = 32.1 Hz), 80.5, 52.8, 47.6, 41.4.

HRMS: (ESI) m/z, $[M]^+$ calcd for $C_{21}H_{17}O_5NF_3$ $[M+H]^+$: 420.1053; found: 420.1044.

4-(Methoxycarbonyl)-3-phenyl-4-(trifluoromethyl)oxetan-2-yl)methyl furan-2-carboxylate (15a).

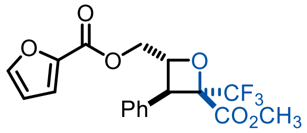

This compound was obtained according to general procedure **2** from cinnamyl furan-2-carboxylate (114 mg, 0.5 mmol), Ir[dF(CF_3)ppy]$_2$(dtbbpy)PF_6 (2.8 mg, 0.5 mol%), Methyl 3,3,3-trifluoro-2-oxopropanoate (77 µL, 0.75 mmol), and dimethyl carbonate (0.4 mL). Isolated as yellowish oil (77 mg, 40% yield) after purification by flash column chromatography (24 g SiO_2, heptane/EA 10:1).

^{1}H NMR (300 MHz, $CDCl_3$): δ 7.53 (dd, *J* = 1.8, 0.9 Hz, 1H), 7.34 – 7.25 (m, 3H), 7.20 – 7.15 (m, 3H), 6.46 (dd, *J* = 3.5, 1.8 Hz, 1H), 5.53 (ddd, *J* = 8.3, 5.5, 3.2 Hz, 1H), 4.66 – 4.39 (m, 3H), 3.38 (s, 3H).

^{19}F NMR (282 MHz, $CDCl_3$): δ -77.29 (s, 3F).

^{13}C NMR (75 MHz, $CDCl_3$): δ 165.4, 158.3, 147.0, 144.0, 132.5, 129.0, 128.8, 127.8, 122.6 (q, *J* = 282.8 Hz), 118.9, 112.1, 86.0 (q, *J* = 32.2 Hz), 80.6, 65.1, 52.9, 45.8 (d, *J* = 1.9 Hz).

HRMS: (ESI) m/z, $[M]^+$ calcd for $C_{18}H_{15}O_6F_3Na$ $[M+Na]^+$: 407.0713; found: 407.0709.

Methyl-4-(((3-oxo-1,3-dihydroisobenzofuran-1-yl)oxy)methyl)-3-phenyl-2-(trifluoromethyl)oxetane-2-carboxylate (16a).

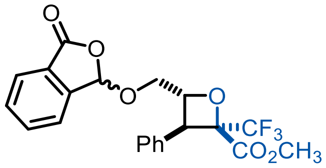

This compound was obtained according to general procedure **2** from 3-(cinnamyloxy)isobenzofuran -1(3H)-one (133 mg, 0.5 mmol), Ir[dF(CF_3)ppy]$_2$(dtbbpy)PF_6 (2.8 mg, 0.5 mol%), Methyl 3,3,3-trifluoro-2-oxopropanoate (77 µL, 0.75 mmol), and dimethyl carbonate (0.4 mL). Isolated as colorless oil (137 mg, 65% yield) after purification by flash column chromatography (24 g SiO_2, heptane/EA 5:1).

Mixture of two diastereomers

^{1}H NMR (300 MHz, $CDCl_3$): δ 7.89 (m, 1H), 7.75 (m, 1H), 7.69 – 7.58 (m, 2H), 7.35 (m, 3H), 7.29 – 7.19 (m, 2H), 6.49 (d, J = 2.8 Hz, 1H), 5.61 – 5.47 (m, 1H), 4.49 (dd, J = 62.4, 7.9 Hz, 1H), 4.28 – 4.07 (m, 2H), 3.45 (d, J = 4.1 Hz, 3H).

^{19}F NMR (282 MHz, $CDCl_3$): δ -76.90, -77.36.

^{13}C NMR (75 MHz, $CDCl_3$): δ 168.3, 168.3, 165.5, 165.3, 144.4, 144.4, 134.6, 134.6, 132.5, 132.5, 131.1, 128.9, 128.8, 128.7, 128.6, 127.8, 127.0, 125.5, 123.8, 122.6 (d, J = 282.8 Hz), 102.2, 102.0, 86.1 (d, J = 32.3 Hz), 86.0 (d, J = 32.3 Hz), 81.4, 81.2, 70.7, 69.4, 52.7, 45.8, 45.8, 45.1.

HRMS: (ESI) m/z, $[M]^+$ calcd for $C_{21}H_{17}O_6F_3Na$ $[M+Na]^+$: 445.0869; found: 445.0870.

Methyl-4-((benzoyloxy)methyl)-3-phenyl-2-(trifluoromethyl)oxetane-2-carboxylate (17a).

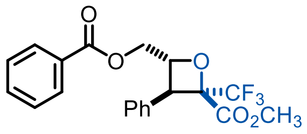

This compound was obtained according to general procedure **2** from cinnamyl benzoate (119 mg, 0.5 mmol), $Ir[dF(CF_3)ppy]_2(dtbbpy)PF_6$ (2.8 mg, 0.5 mol%), Methyl 3,3,3-trifluoro-2-oxopropanoate (77 µL, 0.75 mmol), and dimethyl carbonate (0.4 mL). Isolated as colorless oil (148 mg, 75% yield) after purification by flash column chromatography (24 g SiO_2, heptane/EA 20:1).

^{1}H NMR (300 MHz, $CDCl_3$): δ 8.08 (dd, J = 8.3, 1.2 Hz, 2H), 7.60 – 7.52 (m, 1H), 7.48 – 7.31 (m, 5H), 7.28 (dd, J = 7.6, 1.6 Hz, 2H), 5.67 (ddd, J = 8.4, 5.6, 3.3 Hz, 1H), 4.75 – 4.53 (m, 3H), 3.43 (s, 3H).

^{19}F NMR (282 MHz, $CDCl_3$): δ -77.23 (s, 3F).

^{13}C NMR (75 MHz, $CDCl_3$): δ 166.1, 165.2, 133.3, 132.5, 129.7, 129.4, 128.8, 128.6, 128.4, 127.6, 122.5 (q, J = 282.7 Hz), 85.8 (q, J = 32.1 Hz), 80.6, 65.3, 52.5, 45.7.

HRMS: (ESI) m/z, $[M]^+$ calcd for $C_{20}H_{17}O_5F_3Na$ $[M+Na]^+$: 417.0920; found: 417.0916.

Methyl 2-(trifluoromethyl)-2,2a,7,7a-tetrahydroindeno[2,1-b]oxete-2-carboxylate (18a).

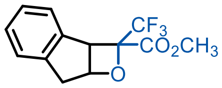

This compound was obtained according to general procedure **2** from 1H-indene (58 µL, 0.5 mmol), $Ir[dF(CF_3)ppy]_2(dtbbpy)PF_6$ (2.8 mg, 0.5 mol%), Methyl 3,3,3-trifluoro-2-oxopropanoate (77 µL, 0.75 mmol), and dimethyl carbonate (0.4 mL). Isolated as colorless oil (61 mg, 45% yield) after purification by flash column chromatography (24 g SiO_2, heptane/EA 20:1).

^{1}H NMR (400 MHz, $CDCl_3$): δ 7.39 – 7.27 (m, 4H), 5.69 (t, J = 5.7 Hz, 1H), 4.43 (d, J = 5.5 Hz, 1H), 3.99 (s, 3H), 3.41 (d, J = 18.1 Hz, 1H), 3.27 (dd, J = 18.0, 6.0 Hz, 1H).

^{19}F NMR (282 MHz, $CDCl_3$): δ -73.68 (s, 3F).

^{13}C NMR (101 MHz, $CDCl_3$): δ 168.6, 143.9, 136.4, 129.1, 127.4, 126.8, 125.6, 121.5 (d, J = 284.6 Hz), 87.1 (q, J = 30.7 Hz), 84.3, 53.7, 52.0, 40.6.

HRMS: (ESI) m/z, $[M]^+$ calcd for $C_{13}H_{11}O_3F_3Na$ $[M+Na]^+$: 295.0553; found: 295.0553.

Methyl 8-(trifluoromethyl)-7-oxabicyclo[4.2.0]oct-2-ene-8-carboxylate (19a).

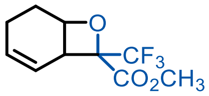

This compound was obtained according to general procedure **2** from cyclohexa-1,3-diene (48 µL, 0.5 mmol), Ir[dF(CF_3)ppy]$_2$(dtbbpy)PF_6 (2.8 mg, 0.5 mol%), Methyl 3,3,3-trifluoro-2-oxopropanoate (77 µL, 0.75 mmol), and dimethyl carbonate (0.4 mL). Isolated as colorless oil (71 mg, 60% yield) after purification by flash column chromatography (24 g SiO_2, heptane/EA 20:1).
^{1}H NMR (300 MHz, $CDCl_3$): δ 6.26 (ddd, J = 9.7, 6.6, 2.4 Hz, 1H), 5.77 (m, 1H), 5.27 (dt, J = 6.7, 3.2 Hz, 1H), 3.91 (s, 3H), 3.50 – 3.40 (m, 1H), 2.37 (m, 1H), 2.18 – 2.02 (m, 2H), 1.39 (m, 1H).
^{19}F NMR (282 MHz, $CDCl_3$): δ -72.80 (s, 3F).
^{13}C NMR (101 MHz, $CDCl_3$): δ 168.8, 134.5 (d, J = 1.2 Hz), 122.0 (q, J = 284.3 Hz), 119.9, 85.8 (q, J = 30.6 Hz), 78.2, 53.4, 39.2, 25.0, 19.4.
HRMS: (ESI) m/z, $[M]^+$ calcd for $C_{10}H_{11}O_3F_3Na$ $[M+Na]^+$: 259.0553; found: 259.0551.

Methyl 4-methoxy-3-phenyl-2-(trifluoromethyl)oxetane-2-carboxylate (20a).

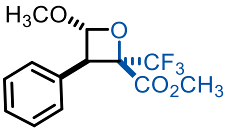

This compound was obtained according to general procedure **2** from (E)-(2-methoxyvinyl)benzene (67 µL, 0.5 mmol), Ir[dF(CF_3)ppy]$_2$(dtbbpy)PF_6 (2.8 mg, 0.5 mol%), Methyl 3,3,3-trifluoro-2-oxopropanoate (77 µL, 0.75 mmol), and dimethyl carbonate (0.4 mL). Isolated as yellowish oil (99 mg, 68% yield) after purification by flash column chromatography (24 g SiO_2, heptane/EA 20:1).
^{1}H NMR (300 MHz, $CDCl_3$): δ 7.41 – 7.31 (m, 3H), 7.24 – 7.18 (m, 2H), 5.87 (d, J = 5.0 Hz, 1H), 4.29 (d, J = 4.9 Hz, 1H), 3.60 (s, 3H), 3.44 (s, 3H).
^{19}F NMR (282 MHz, $CDCl_3$): δ -77.33 (s, 3F).
^{13}C NMR (101 MHz, $CDCl_3$): δ 166.0, 132.1, 129.0, 128.7, 127.9, 122.5 (q, J = 283.0 Hz), 82.0 (q, J = 32.7 Hz), 56.6, 52.9, 52.2 (d, J = 2.1 Hz).
HRMS: (ESI) m/z, $[M]^+$ calcd for $C_{13}H_{13}O_4F_3Na$ $[M+Na]^+$: 313.0658; found: 313.0655.

Methyl-4-(((4-fluorobenzoyl)oxy)methyl)-3-phenyl-2-(trifluoromethyl)oxetane-2-carboxylate (21a).

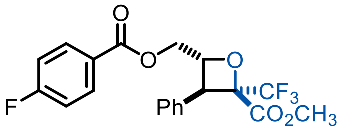

This compound was obtained according to general procedure **2** from cinnamyl 4-fluorobenzoate (128 mg, 0.5 mmol), Ir[dF(CF_3)ppy]$_2$(dtbbpy)PF_6 (2.8 mg, 0.5 mol%), Methyl 3,3,3-trifluoro-2-oxopropanoate (77 µL, 0.75 mmol), and dimethyl carbonate (0.4 mL). Isolated as colorless oil (89 mg, 43% yield) after purification by flash column chromatography (24 g SiO_2, heptane/EA 20:1).

^{1}H NMR (300 MHz, $CDCl_3$): δ 8.06 – 7.95 (m, 2H), 7.32 – 7.24 (m, 3H), 7.20 – 7.16 (m, 2H), 7.09 – 6.98 (m, 2H), 5.55 (ddd, J = 8.4, 5.6, 3.3 Hz, 1H), 4.67 – 4.49 (m, 2H), 4.42 (d, J = 8.0 Hz, 1H), 3.37 (s, 3H).

^{19}F NMR (282 MHz, $CDCl_3$): δ -77.29 (s, 3F).

^{13}C NMR (75 MHz, $CDCl_3$): δ 166.1 (d, J = 254.6 Hz), 165.4, 165.4, 132.5 (d, J = 9.4 Hz), 132.5, 129.0, 128.9, 127.8, 125.8, 125.7, 122.6 (q, J = 282.7 Hz), 115.8 (d, J = 22.0 Hz), 86.0 (q, J = 32.3 Hz), 80.8, 65.5, 52.8, 45.9 (d, J = 1.7 Hz).

HRMS: (ESI) m/z, $[M]^+$ calcd for $C_{20}H_{16}O_5F_4Na$ $[M+Na]^+$: 435.0826; found: 435.0823.

Methyl-4-(((4-bromobenzoyl)oxy)methyl)-3-phenyl-2-(trifluoromethyl)oxetane-2-carboxylate (22a).

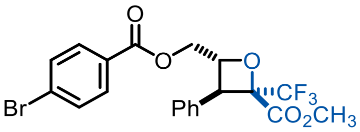

This compound was obtained according to general procedure **2** from cinnamyl 4-bromobenzoate (159 mg, 0.5 mmol), Ir[dF(CF_3)ppy]$_2$(dtbbpy)PF_6 (2.8 mg, 0.5 mol%), Methyl 3,3,3-trifluoro-2-oxopropanoate (77 µL, 0.75 mmol), and dimethyl carbonate (0.4 mL). Isolated as white solid (99 mg, 42% yield) after purification by flash column chromatography (24 g SiO_2, heptane/EA 20:1).

^{1}H NMR (300 MHz, $CDCl_3$): δ 7.87 – 7.81 (m, 2H), 7.54 – 7.48 (m, 2H), 7.34 – 7.25 (m, 3H), 7.21 – 7.15 (m, 2H), 5.55 (ddd, J = 8.4, 5.6, 3.2 Hz, 1H), 4.65 – 4.49 (m, 2H), 4.41 (d, J = 8.0 Hz, 1H), 3.38 (s, 3H).

^{19}F NMR (282 MHz, $CDCl_3$): δ -77.28 (s, 3F).

^{13}C NMR (75 MHz, $CDCl_3$): δ 165.7, 165.4, 132.5, 132.0, 131.4, 129.1, 128.9, 128.7, 128.4, 127.8, 122.6 (q, J = 282.8 Hz), 86.0 (q, J = 32.1 Hz), 80.7, 65.7, 52.9, 46.0 (d, J = 1.8 Hz).

HRMS: (ESI) m/z, $[M]^+$ calcd for $C_{20}H_{16}O_5BrF_3Na$ $[M+Na]^+$: 495.0025; found: 495.0025.

Methyl-4-(((4-chlorobenzoyl)oxy)methyl)-3-phenyl-2-(trifluoromethyl)oxetane-2-carboxylate (23a).

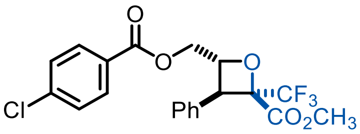

This compound was obtained according to general procedure **2** from cinnamyl 4-chlorobenzoate (136 mg, 0.5 mmol), Ir[dF(CF_3)ppy]$_2$(dtbbpy)PF_6 (2.8 mg, 0.5 mol%), Methyl 3,3,3-trifluoro-2-oxopropanoate (77 µL, 0.75 mmol), and dimethyl carbonate (0.4 mL). Isolated as colorless oil (88 mg, 41% yield) after purification by flash column chromatography (24 g SiO_2, heptane/EA 20:1).

^{1}H NMR (300 MHz, $CDCl_3$): δ 8.04 – 7.96 (m, 2H), 7.44 – 7.40 (m, 2H), 7.36 (m, 3H), 7.25 (dd, J = 7.7, 1.8 Hz, 2H), 5.63 (ddd, J = 8.4, 5.6, 3.2 Hz, 1H), 4.75 – 4.57 (m, 2H), 4.49 (d, J = 8.0 Hz, 1H), 3.45 (s, 3H).

^{19}F NMR (282 MHz, $CDCl_3$): δ -77.28 (s, 3F).

^{13}C NMR (75 MHz, $CDCl_3$): δ 165.5, 165.4, 140.0, 132.5, 131.3, 129.1, 129.0, 128.9, 127.9, 127.8,

122.6 (q, *J* = 282.7 Hz), 86.0 (q, *J* = 32.2 Hz), 80.7, 65.6, 52.9, 46.0 (d, *J* = 1.8 Hz).
HRMS: (ESI) m/z, $[M]^+$ calcd for $C_{20}H_{16}O_5ClF_3Na$ $[M+Na]^+$: 451.0531; found: 451.0524.

Methyl-3-phenyl-2-(trifluoromethyl)-4-(((4-(trifluoromethyl)benzoyl)oxy)methyl)oxetane-2-carboxylate (24a).

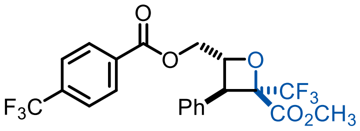

This compound was obtained according to general procedure **2** from cinnamyl 4-(trifluoromethyl)benzoate (153 mg, 0.5 mmol), Ir[dF(CF_3)ppy]$_2$(dtbbpy)PF_6 (2.8 mg, 0.5 mol%), Methyl 3,3,3-trifluoro-2-oxopropanoate (77 µL, 0.75 mmol), and dimethyl carbonate (0.4 mL). Isolated as colorless oil (83 mg, 36% yield) after purification by flash column chromatography (24 g SiO_2, heptane/EA 20:1).
^{1}H NMR (300 MHz, $CDCl_3$): δ 8.08 (dt, *J* = 8.0, 0.8 Hz, 2H), 7.66 – 7.58 (m, 2H), 7.32 – 7.23 (m, 3H), 7.19 – 7.14 (m, 2H), 5.55 (ddd, *J* = 8.4, 5.6, 3.2 Hz, 1H), 4.69 – 4.50 (m, 2H), 4.41 (d, *J* = 8.0 Hz, 1H), 3.36 (s, 3H).
^{19}F NMR (282 MHz, $CDCl_3$): δ -63.20 (s, 3F), -77.31 (s, 3F).
^{13}C NMR (75 MHz, $CDCl_3$): δ 165.4, 165.2, 134.9 (q, J = 32.6 Hz), 132.7, 132.4, 130.3, 129.1, 128.9, 127.8, 125.6 (q, *J* = 3.8 Hz), 123.7 (q, *J* = 272.7 Hz), 122.6 (q, *J* = 282.6 Hz), 86.0 (q, *J* = 32.3 Hz), 80.6, 65.9, 52.9, 45.9, 45.9.
HRMS: (ESI) m/z, $[M]^+$ calcd for $C_{21}H_{16}O_5F_6Na$ $[M+Na]^+$: 485.0794; found: 485.0792.

Methyl-4-(((4-cyanobenzoyl)oxy)methyl)-3-phenyl-2-(trifluoromethyl)oxetane-2-carboxylate (25a).

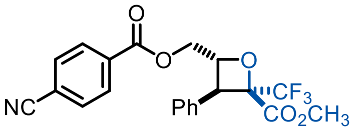

This compound was obtained according to general procedure **2** from cinnamyl 4-cyanobenzoate (132 mg, 0.5 mmol), Ir[dF(CF_3)ppy]$_2$(dtbbpy)PF_6 (2.8 mg, 0.5 mol%), Methyl 3,3,3-trifluoro-2-oxopropanoate (77 µL, 0.75 mmol), and dimethyl carbonate (0.4 mL). Isolated as white solid (132 mg, 63% yield) after purification by flash column chromatography (24 g SiO_2, heptane/EA 5:1).
^{1}H NMR (300 MHz, $CDCl_3$): δ 8.09 – 8.02 (m, 2H), 7.70 – 7.61 (m, 2H), 7.31 – 7.23 (m, 3H), 7.18 – 7.13 (m, 2H), 5.54 (ddd, *J* = 8.4, 5.7, 3.1 Hz, 1H), 4.67 – 4.50 (m, 2H), 4.39 (d, *J* = 8.0 Hz, 1H), 3.35 (s, 3H).
^{19}F NMR (282 MHz, $CDCl_3$): δ -77.27 (s, 3F).
^{13}C NMR (75 MHz, $CDCl_3$): δ 165.3, 164.7, 133.3, 132.4, 132.3, 130.4, 129.1, 129.0, 127.8, 122.5 (d, *J* = 282.7 Hz), 117.9, 116.9, 86.0 (d, *J* = 32.1 Hz), 80.5, 66.0, 52.9, 45.8.
HRMS: (ESI) m/z, $[M]^+$ calcd for $C_{21}H_{17}O_5NF_3$ $[M+H]^+$: 420.1053; found: 420.1048.

Ethyl-4-(((2-(4-isobutylphenyl)propanoyl)oxy)methyl)-3-phenyl-2-(trifluoromethyl)oxetane-2-carboxylate (26a).

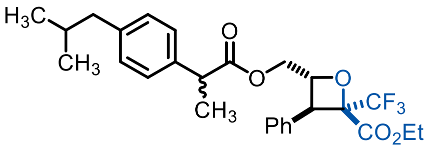

This compound was obtained according to general procedure **2** from cinnamyl 2-(4-isobutylphenyl) propanoate (161 mg, 0.5 mmol), Ir[dF(CF_3)ppy]$_2$(dtbbpy)PF_6 (2.8 mg, 0.5 mol%), Ethyl 3,3,3-trifluoro-2-oxopropanoate (99 µL, 0.75 mmol), and dimethyl carbonate (0.4 mL). Isolated as colorless oil (165 mg, 67% yield) after purification by flash column chromatography (24 g SiO_2, heptane/EA 20:1).

Mixture of two diastereomers

^{1}H NMR (300 MHz, $CDCl_3$): δ 7.32 (m, 3H), 7.21 – 7.04 (m, 6H), 5.53 – 5.36 (m, 1H), 4.54 – 4.23 (m, 3H), 4.00 – 3.69 (m, 3H), 2.46 (dd, *J* = 7.2, 4.6 Hz, 2H), 1.86 (m, 1H), 1.49 (d, *J* = 7.2 Hz, 3H), 0.91 (dd, *J* = 6.6, 2.0 Hz, 6H), 0.85 (t, *J* = 7.1 Hz, 3H).

^{19}F NMR (282 MHz, $CDCl_3$): δ -77.07, -77.33.

^{13}C NMR (75 MHz, $CDCl_3$): δ 174.5, 174.5, 164.9, 164.8, 140.8, 140.7, 137.5, 137.2, 132.7, 132.6, 129.5, 129.4, 128.8, 128.8, 128.6, 128.6, 127.9, 127.2, 127.2, 122.6 (d, *J* = 282.7 Hz), 85.6 (d, *J* = 32.0 Hz), 85.5 (d, *J* = 32.3 Hz), 80.5, 80.4, 65.4, 64.7, 62.9, 62.3, 62.3, 46.2, 45.5, 45.1, 45.0, 44.9, 30.2, 22.5, 22.5, 22.4, 18.5, 18.4, 13.5.

HRMS: (ESI) m/z, $[M]^+$ calcd for $C_{27}H_{31}O_5F_3Na$ $[M+Na]^+$: 515.2016; found: 515.2015.

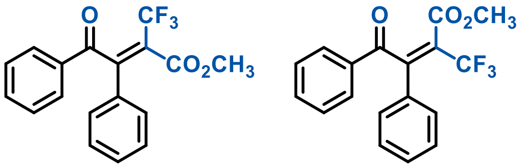

(Z)-27 **(E)-27**

This compound was obtained according to general procedure **1** from 1,2-diphenylethyne (180 µL, 1.0 mmol), Ir[dF(CF_3)ppy]$_2$(dtbbpy)PF_6 (5.6 mg, 0.5 mol%), Methyl 3,3,3-trifluoro-2-oxopropanoate (153 µL, 1.50 mmol), and dimethyl carbonate (0.8 mL). After 16 h, the reaction mixture (57% yield with **(Z)-27**: **(E)-27** = 28:29 by ^{19}F NMR) was purified by HPLC on the Teledyne Isco CombiFlash EZ Prep system using a Macherey-Nagel VP 250/21 Nucleosil 50-5 columns to provide the title compounds.

Methyl (Z)-4-oxo-3,4-diphenyl-2-(trifluoromethyl)but-2-enoate ((Z)-27)

^{1}H NMR (300 MHz, $CDCl_3$): δ 7.96 – 7.89 (m, 2H), 7.62 – 7.55 (m, 1H), 7.52 – 7.40 (m, 4H), 7.37 (m, 3H), 3.61 (s, 3H).

^{19}F NMR (282 MHz, $CDCl_3$): δ -58.69 (s, 3F).

^{13}C NMR (75 MHz, $CDCl_3$): δ 192.5, 162.7, 158.4, 134.2, 134.1, 132.2, 129.9, 129.1, 128.7, 127.7 (q, *J* = 1.9 Hz), 123.4, 121.6 (d, *J* = 276.0 Hz), 119.8, 53.0.

Methyl (E)-4-oxo-3,4-diphenyl-2-(trifluoromethyl)but-2-enoate ((Z)-27)

^{1}H NMR (400 MHz, $CDCl_3$): δ 7.97 – 7.93 (m, 2H), 7.62 – 7.57 (m, 1H), 7.50 – 7.42 (m, 4H), 7.37

(tdd, *J* = 4.2, 2.6, 1.3 Hz, 3H), 3.62 (s, 3H).

^{19}F NMR (282 MHz, $CDCl_3$): δ -58.69 (s, 3F).

^{13}C NMR (101 MHz, $CDCl_3$): δ 191.9, 163.5 (d, *J* = 2.1 Hz), 151.8 (d, *J* = 3.5 Hz), 134.6, 134.3, 132.9, 130.2, 129.7, 129.1, 128.7, 127.6, 123.4 (q, *J* = 32.8 Hz), 121.3 (q, *J* = 275.4 Hz), 53.0.

HRMS: (ESI) m/z, $[M]^+$ calcd for $C_{18}H_{13}O_3F_3Na$ $[M+Na]^+$: 357.0709; found: 357.0706.

9. Scale-up synthesis and product transformations

Batch set-up

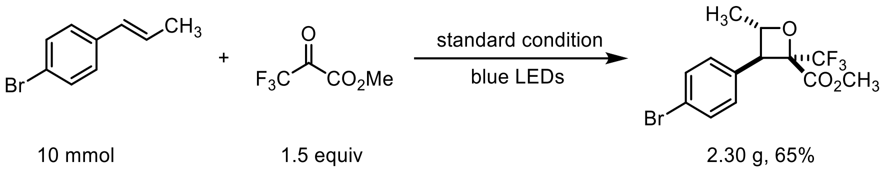

Figure S20. Scale-up reaction.

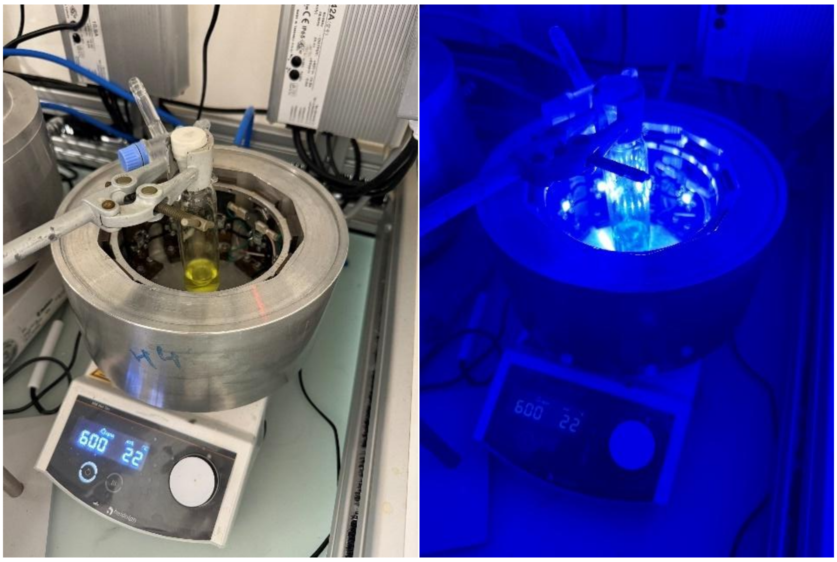

Figure S21. Batch setup for scale-up.

Batch set-up: In an oven dried Schlenk flask, 1-bromo-4-(prop-1-en-1-yl)benzene (**S10**, 1.97 g, 10.0 mmol), methyl 3,3,3-trifluoro-2-oxopropanoate (1.53 mL, 15.0 mmol) and Ir[dF(CF$_3$)ppy]$_2$(dtbbpy)PF$_6$ (56 mg, 50 µmol, 0.5 mol%) were dissolved in 10 mL dimethyl carbonate, then the reaction flask was sealed and subjected to three freeze-pump-thaw cycles and backfilled with N_2. The flask was then placed in the photoreactor and irradiated with 440 nm blue LEDs at room temperature for 24 h. Reaction contents were purified by flash column chromatography to afford methyl-3-(4-bromophenyl)-4-methyl-2-(trifluoromethyl)oxetane-2-carboxylate as a colorless oil (2.30 g, 6.5 mmol, 65% yield).

Product transformations

3-(4-Bromophenyl)-4-methyl-2-(trifluoromethyl)oxetan-2-yl)methanol (28).

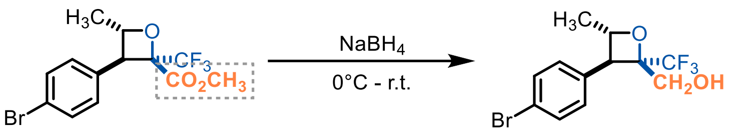

To an oven dried 8 mL crimp cap vial was added THF (0.5 mL), methanol (0.5 mL), methyl-3-(4-bromophenyl)-4-methyl-2-(trifluoromethyl)oxetane-2-carboxylate **14a** (52.0 mg, 1.0 equiv) and lithium chloride (7.50 mg, 1.2 equiv). This was cooled to a temperature of 5 °C prior to the addition of sodium borohydride (11.14 mg, 2 equiv), maintaining an internal temperature below 10 °C. The temperature was increased to 25 °C, and upon reaction completion, the mixture was quenched with saturated aqueous potassium sodium tartrate (6 mL). The aqueous phase was then further extracted with ethyl acetate (3 × 5 mL). The combined organic phases were dried with anhydrous sodium sulfate then concentrated and purified by flash column chromatography to afford 3-(4-Bromophenyl)-4-methyl-2-(trifluoromethyl)oxetan-2-yl)methanol as a colorless oil (29.0 mg, 61% yield).

^{1}H NMR (300 MHz, $CDCl_3$): δ 7.57 – 7.45 (m, 2H), 7.17 – 7.04 (m, 2H), 5.38 – 5.24 (m, 1H), 4.04 (d, *J* = 7.7 Hz, 1H), 3.81 – 3.57 (m, 2H), 1.77 (s, 1H), 1.59 (d, *J* = 6.1 Hz, 3H).

^{19}F NMR (282 MHz, $CDCl_3$): δ -80.67 (s, 3F).

^{13}C NMR (101 MHz, $CDCl_3$): δ 133.1, 132.1, 129.5, 124.2 (q, *J* = 283.2 Hz), 121.9, 82.8 (q, *J* = 29.8 Hz), 78.7, 60.1, 48.1 (d, *J* = 1.8 Hz), 22.4.

HRMS: (ESI) m/z, $[M]^+$ calcd for $C_{12}H_{12}O_2BrF_3Na$ $[M+Na]^+$: 346.9865; found: 346.9863.

Methyl-3-(3'-(9H-carbazol-9-yl)-[1,1'-biphenyl]-4-yl)-4-methyl-2-(trifluoromethyl)oxetane-2-carboxylate (29).

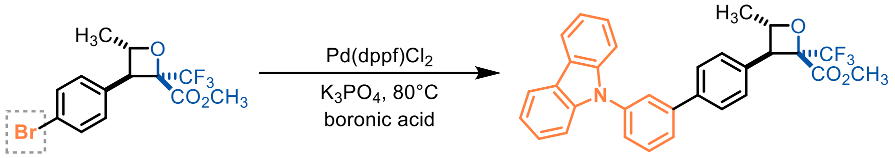

To an oven dried 8 mL crimp cap vial was added methyl-3-(4-bromophenyl)-4-methyl-2-(trifluoromethyl)oxetane-2-carboxylate **14a** (130 mg, 0.368 mmol, 1.0 equiv), (3-(9H-carbazol-9-yl)phenyl)boronic acid (116 mg, 0.405 mmol, 1.1 equiv), $Pd(dppf)Cl_2$ (10.8 mg, 0.015 mmol, 4 mol%), and K_3PO_4 (234 mg, 1.10 mmol, 3 equiv). The vial was capped and purged with N_2 before the addition of 1,4-dioxane (2 mL, 0.25 M), followed by H_2O (33.2 µL, 1.84 mmol, 5 equiv). The reaction mixture was heated for 16 h at 80 °C with stirring. The vial was then allowed to cool to room temperature, opened, diluted with EtOAc (10 mL) and filtered through a plug of celite, eluting with EtOAc. The resulting solution was washed with H_2O (3 × 10 mL) followed by brine (10 mL) and the organic phases collected. The organic phase was dried over Na_2SO_4, filtered, and concentrated under vacuum. The crude residue was purified by column chromatography to afford the desired product as a white solid (104 mg, 55%).

^{1}H NMR (300 MHz, $CDCl_3$): δ 8.23 – 8.13 (m, 2H), 7.80 (m, 1H), 7.72 – 7.63 (m, 4H), 7.62 – 7.56 (m, 1H), 7.51 – 7.40 (m, 4H), 7.39 – 7.27 (m, 4H), 5.53 – 5.37 (m, 1H), 4.17 (d, *J* = 7.5 Hz, 1H), 3.50 (s, 3H), 1.63 (d, *J* = 6.1 Hz, 3H).
^{19}F NMR (282 MHz, $CDCl_3$): δ -77.74 (s, 3F).
^{13}C NMR (75 MHz, $CDCl_3$): δ 166.0, 142.2, 141.0, 140.3, 138.5, 133.3, 130.6, 128.6, 127.6, 126.4, 126.2, 126.1, 125.7, 122.7 (d, *J* = 282.9 Hz), 120.5, 120.2, 109.8, 85.5, 85.0, 80.9, 52.9, 50.8, 22.0.
HRMS: (ESI) m/z, $[M]^+$ calcd for $C_{31}H_{25}O_3NF_3$ $[M+H]^+$: 516.1781; found: 516.1774.

10. NMR Spectra

10.1. 2D NMR Studies and Stereochemical Assignment

Typical isomers

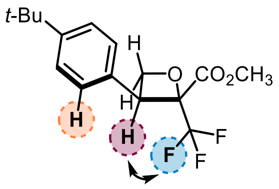

3a

^{1}H-^{19}F NOESY of **3a**

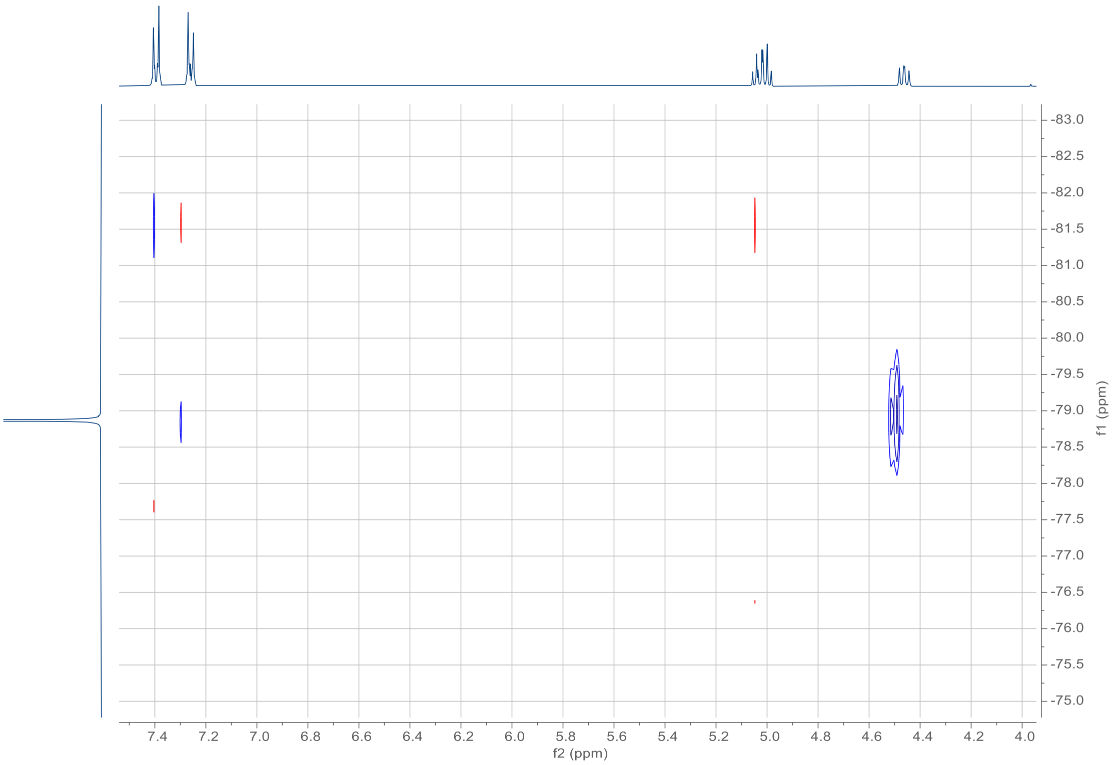

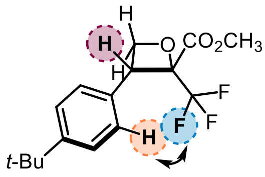

3a'

^{1}H-^{19}F NOESY of **3a'**

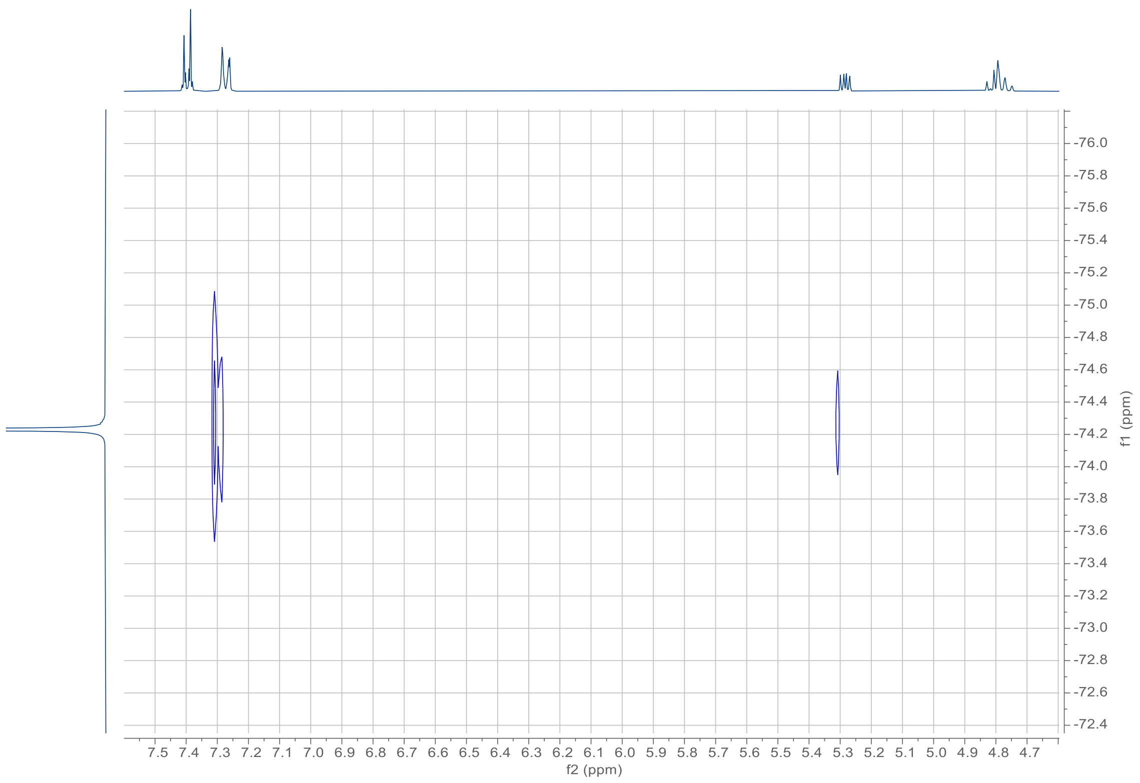

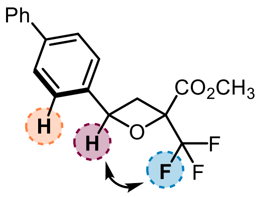

10b

^{1}H-^{19}F NOESY of **10b**

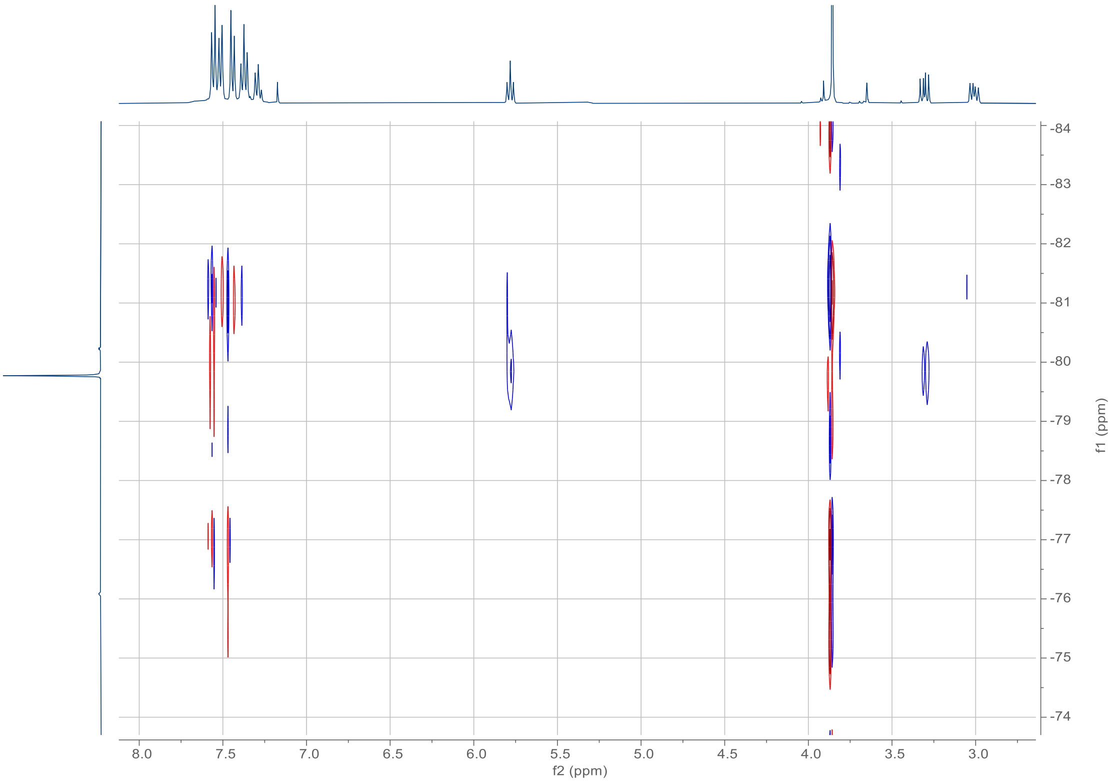

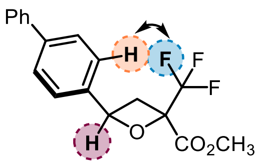

10b'

^{1}H-^{19}F NOESY of **10b'**

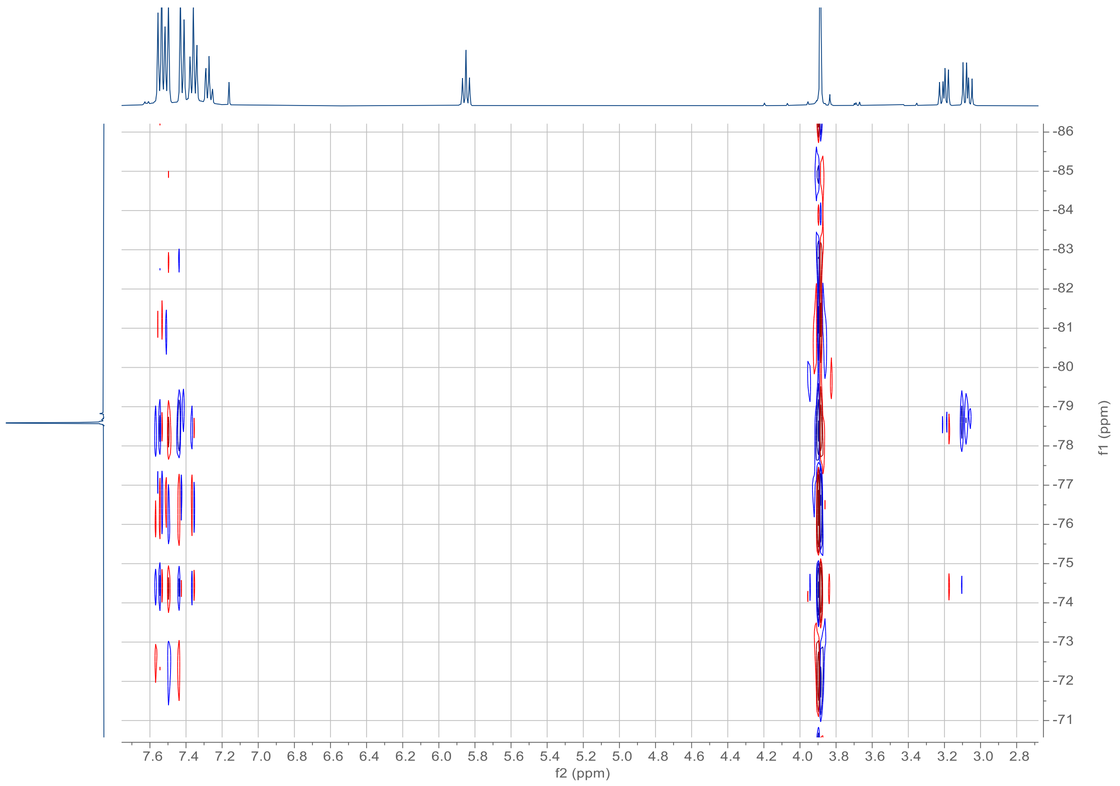

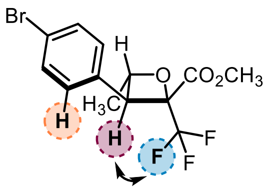

14a

^{1}H-^{19}F NOESY of **14a**

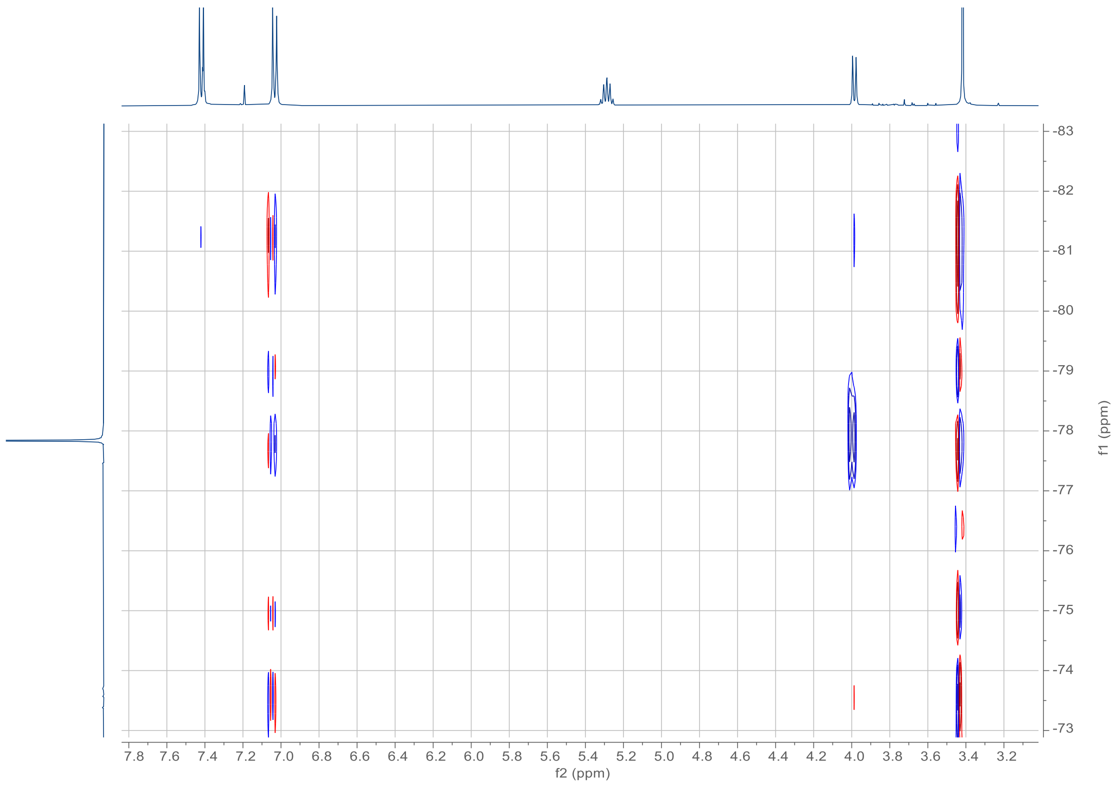

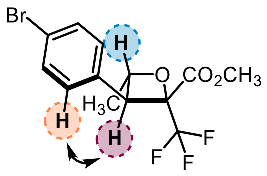

14a

^{1}H-^{1}H NOESY of **14a**

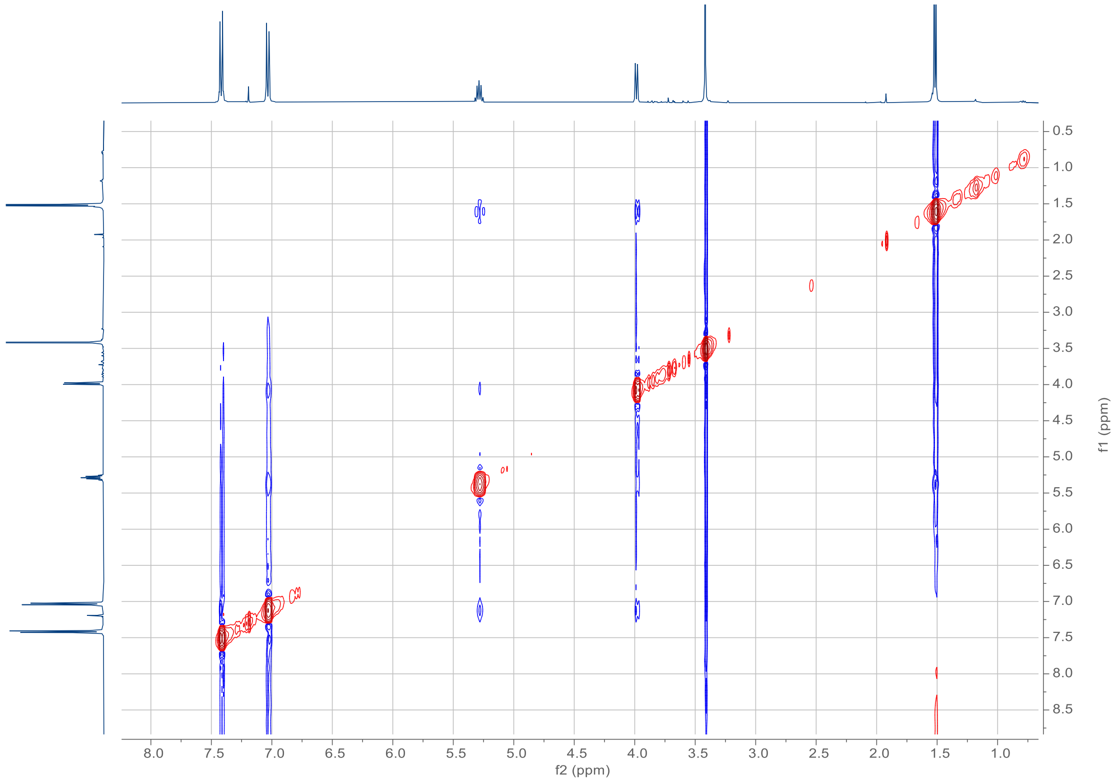

10.2. Copies of NMR spectra

^{1}H NMR (300 MHz, $CDCl_3$) of **S1**

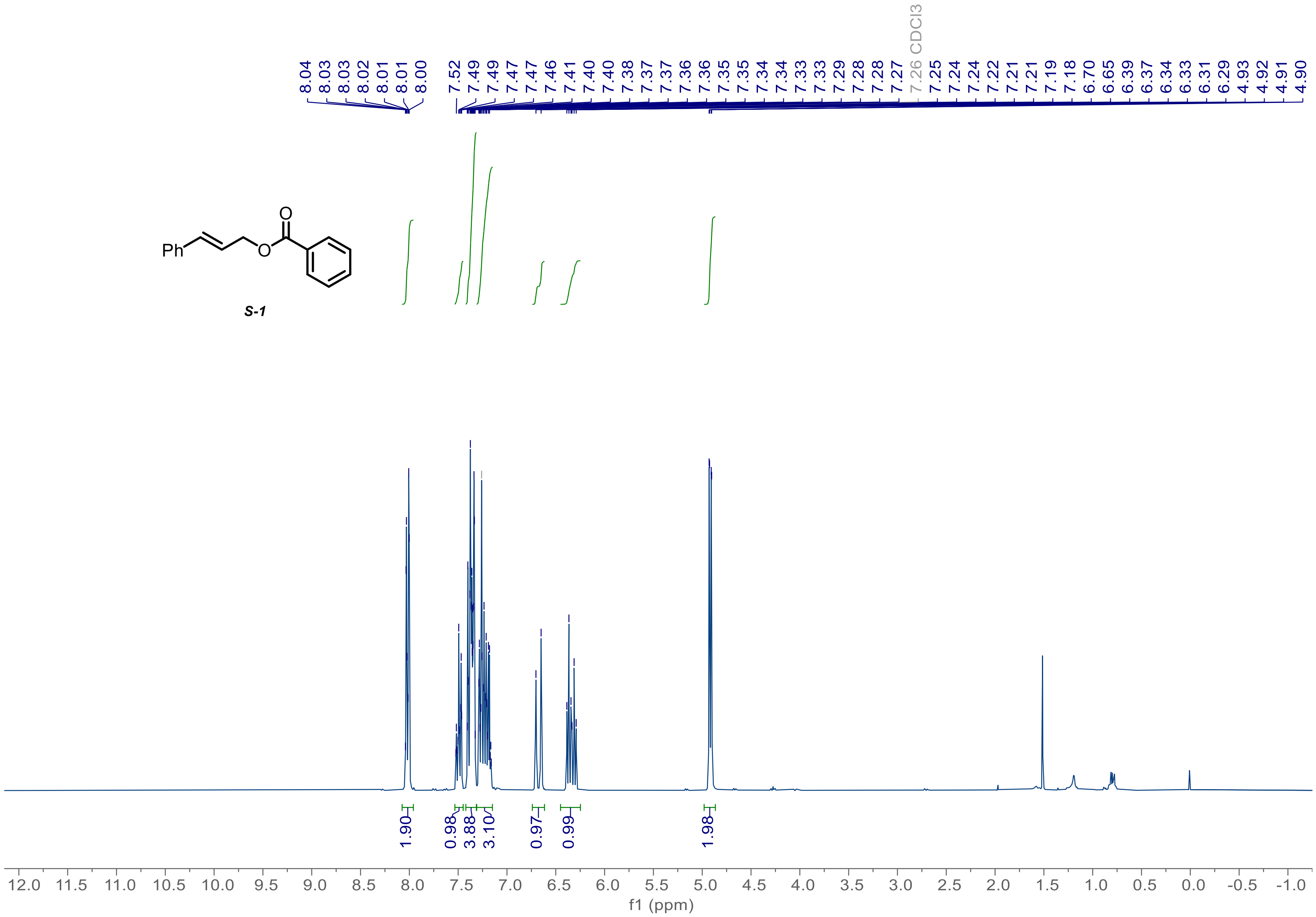

^{13}C NMR (75 MHz, $CDCl_3$) of **S1**

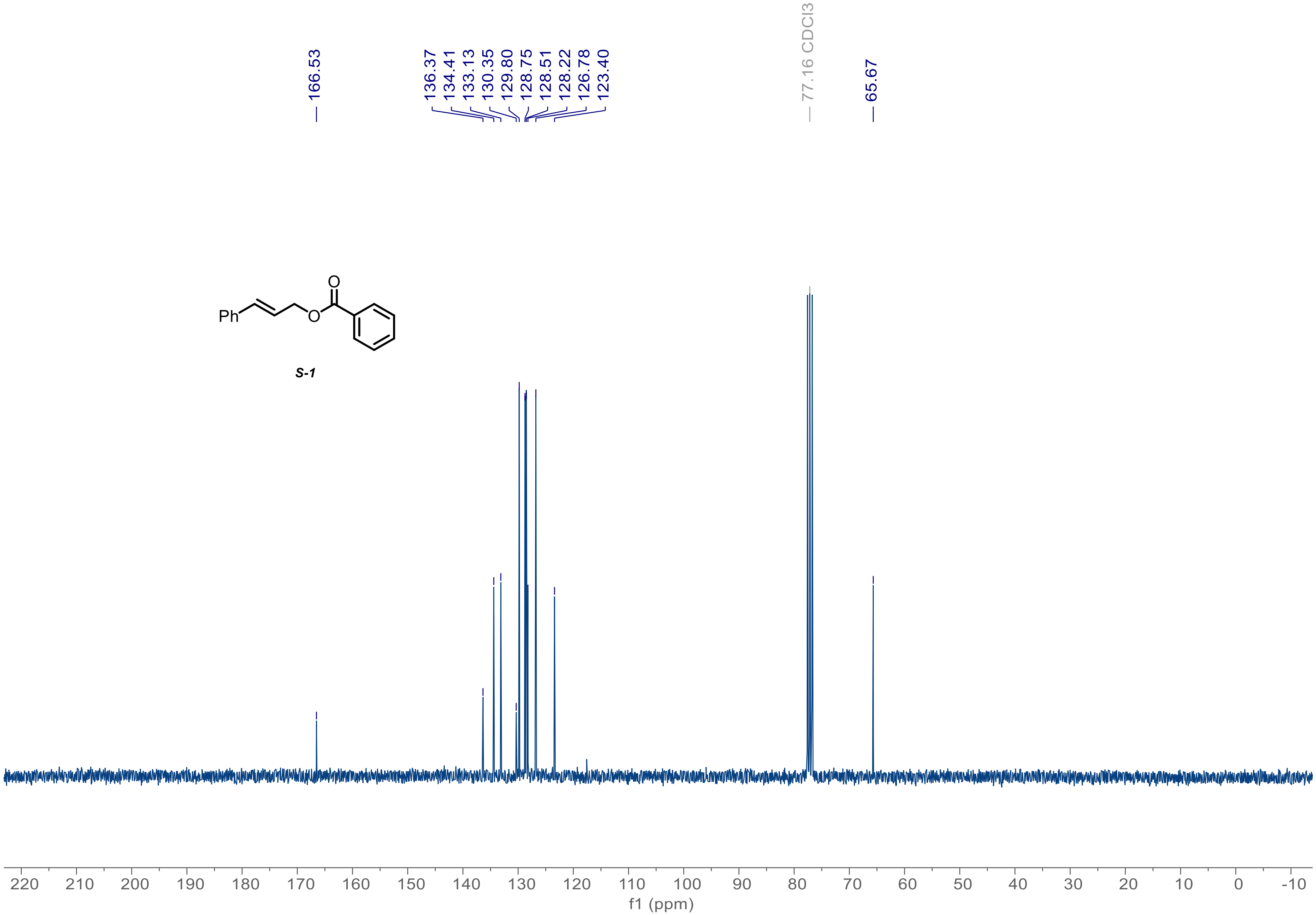

^{1}H NMR (300 MHz, $CDCl_3$) of **S2**

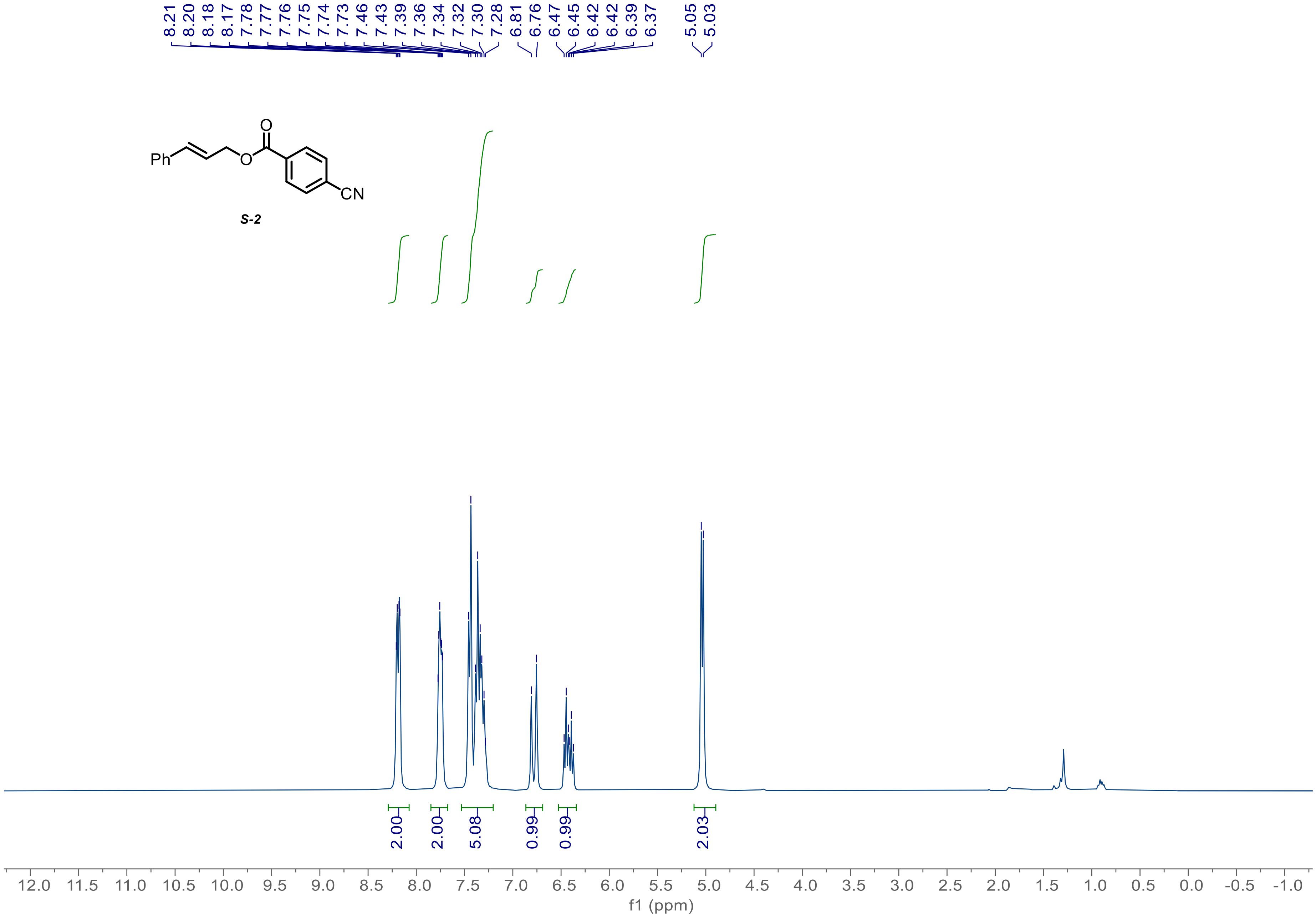

^{13}C NMR (75 MHz, $CDCl_3$) of **S2**

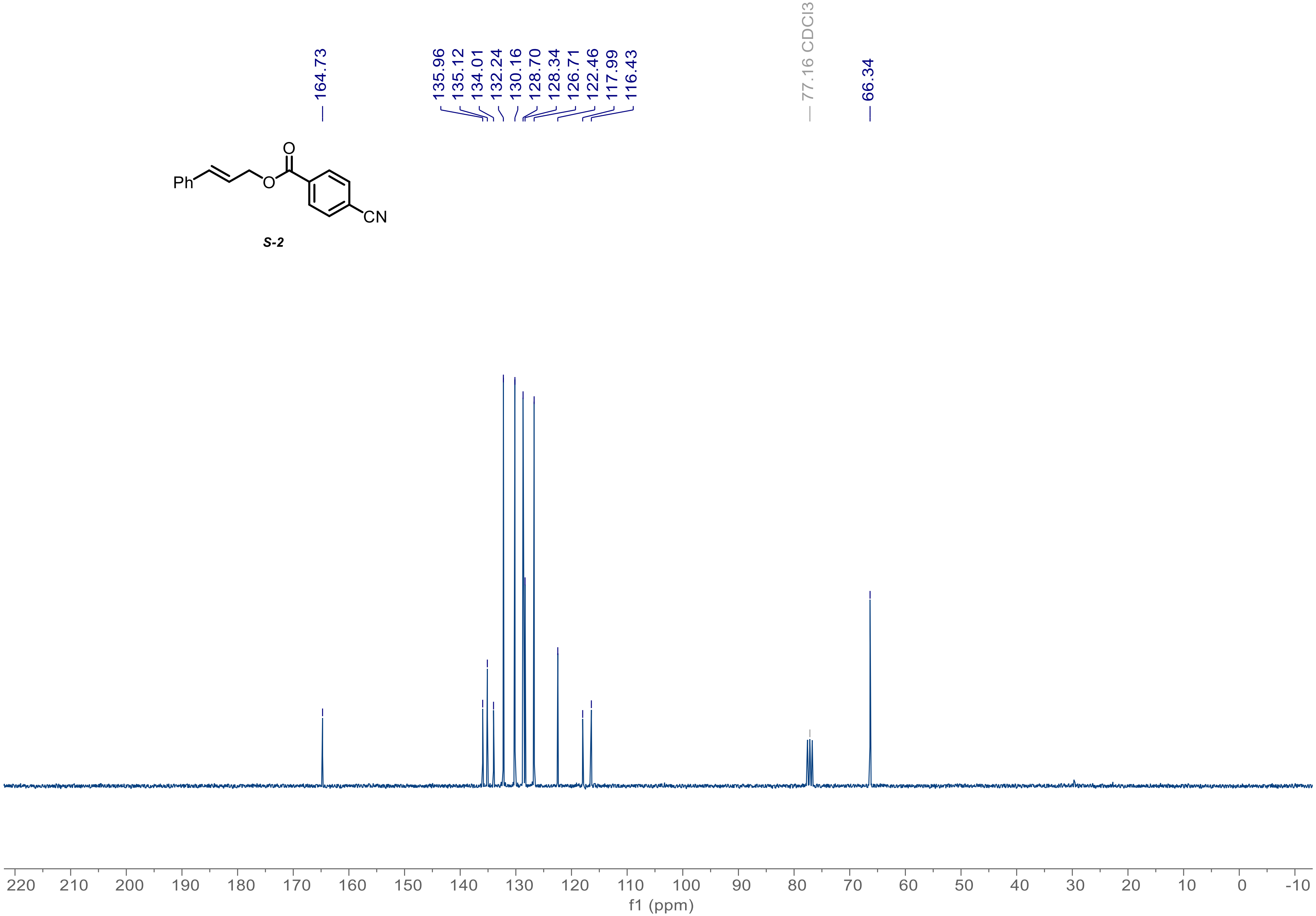

^{1}H NMR (300 MHz, $CDCl_3$) of **S3**

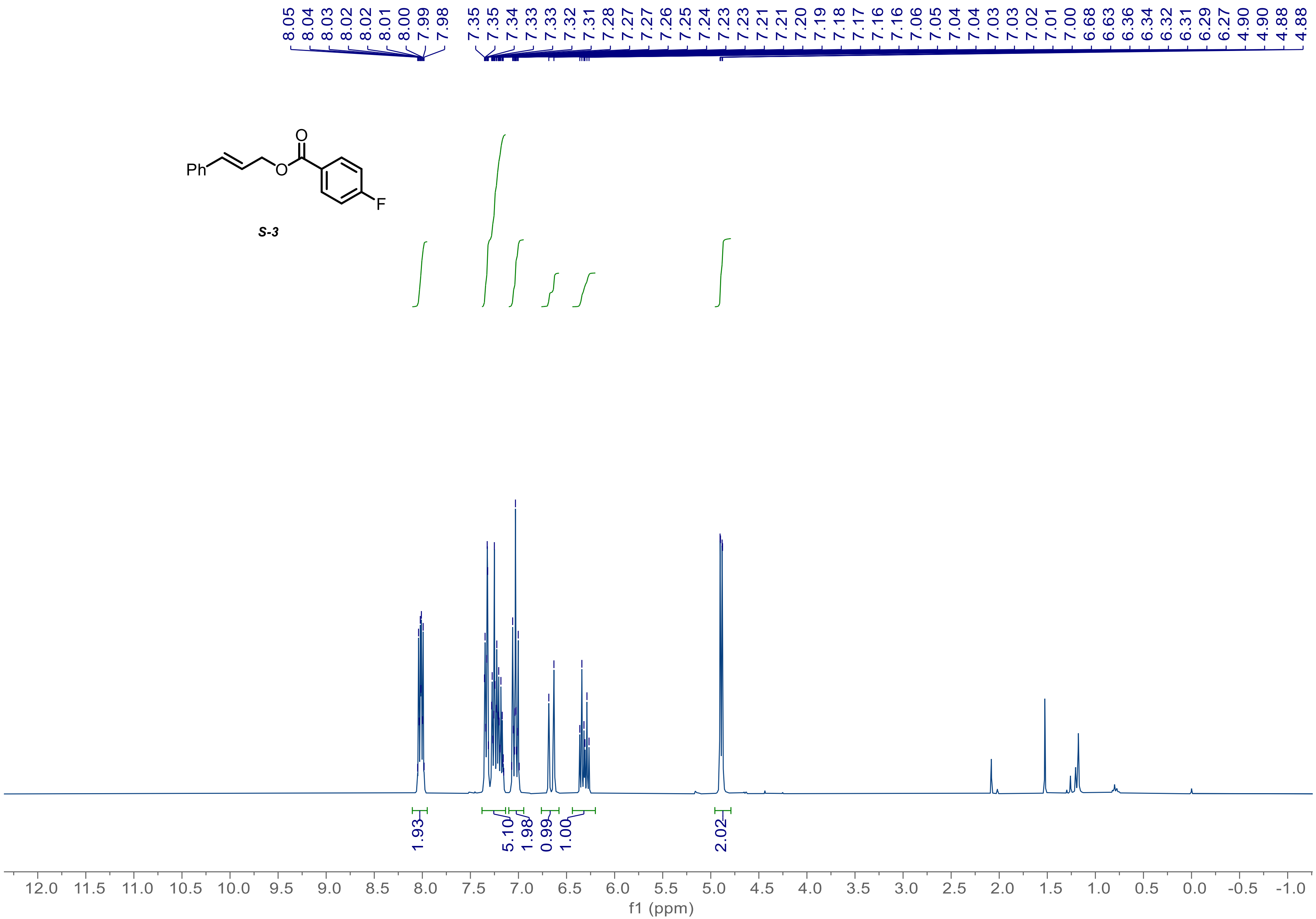

^{13}C NMR (75 MHz, $CDCl_3$) of **S3**

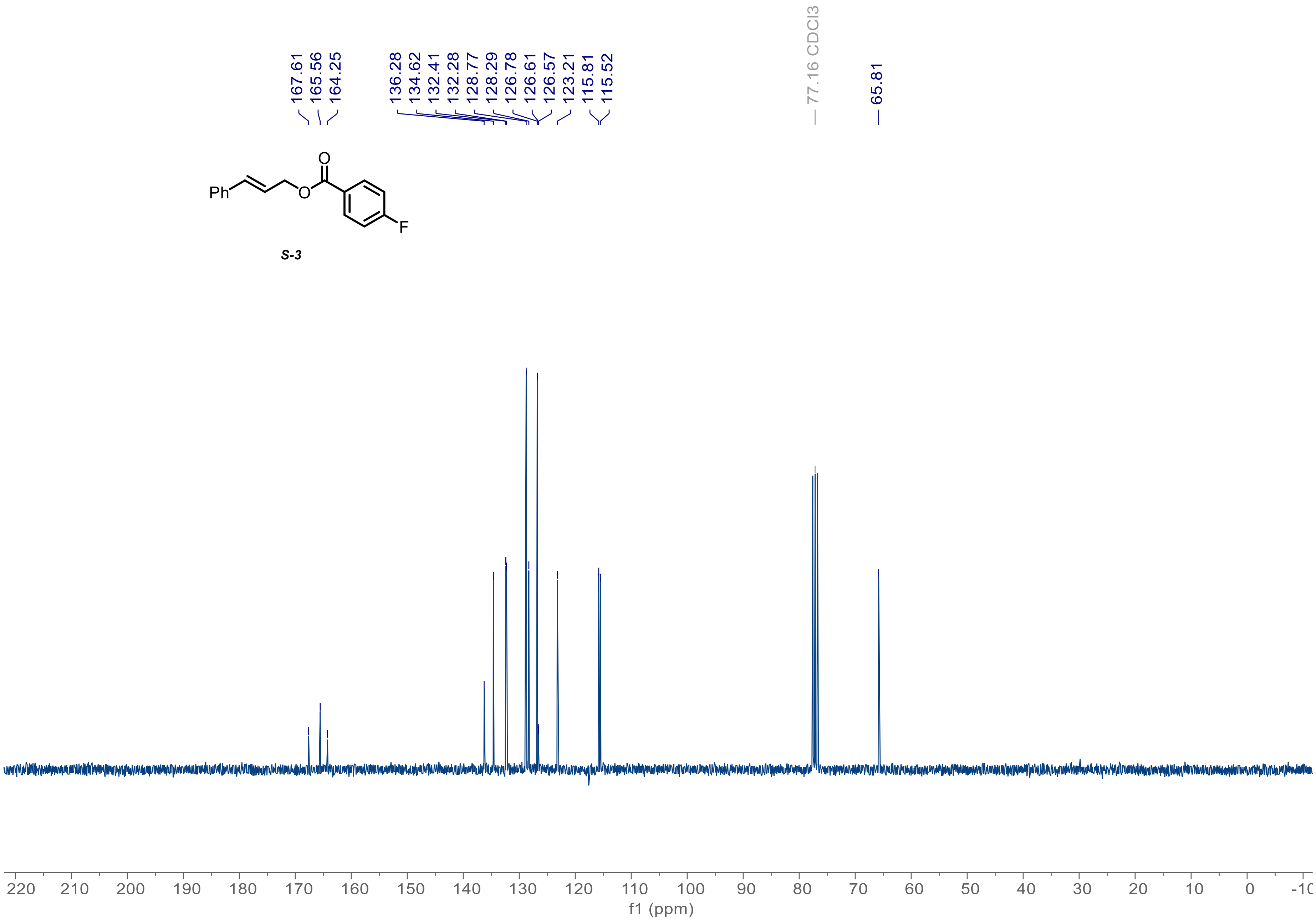

^{19}F NMR (282 MHz, $CDCl_3$) of **S3**

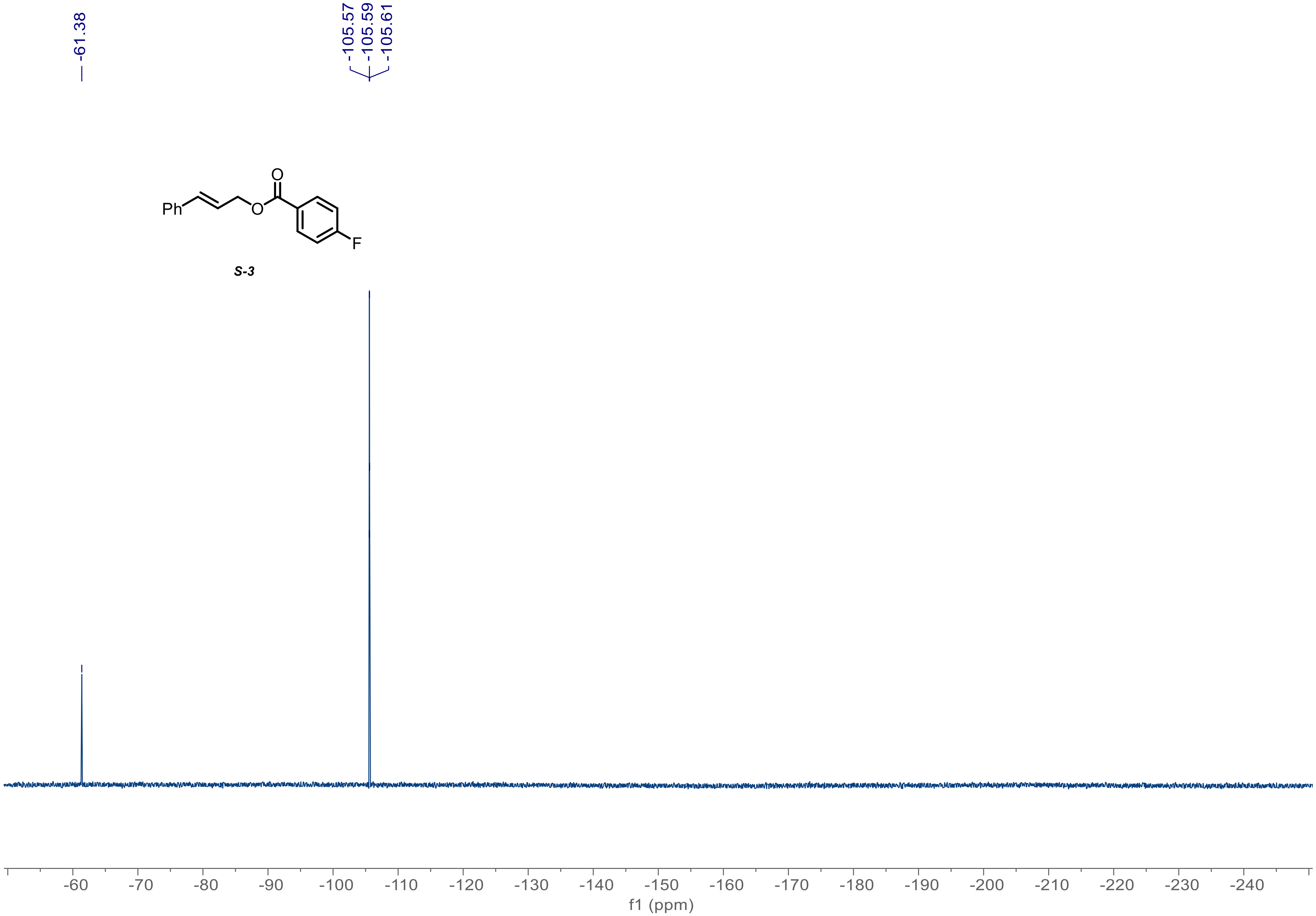

^{1}H NMR (300 MHz, $CDCl_3$) of **S4**

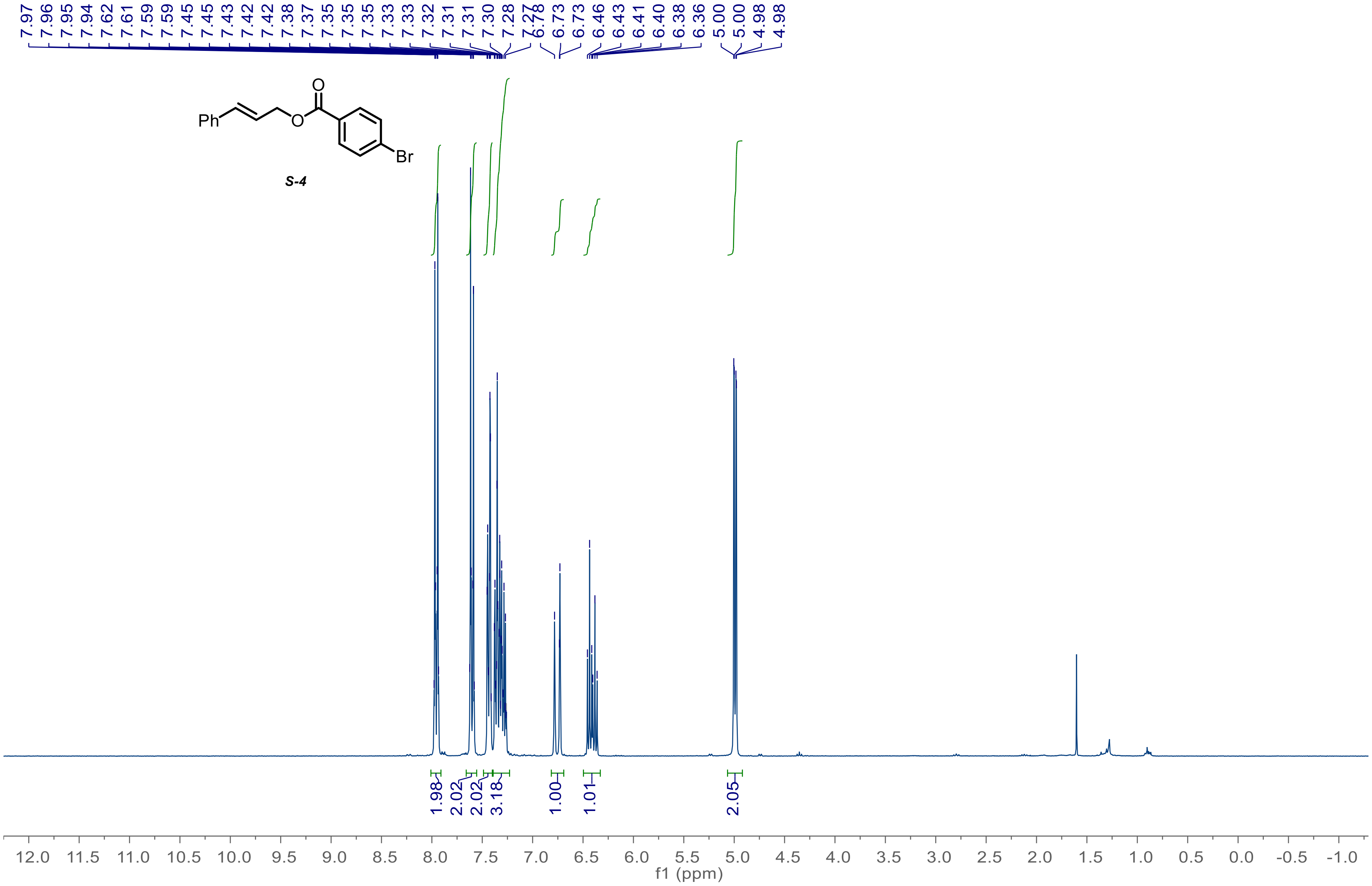

^{13}C NMR (75 MHz, $CDCl_3$) of **S4**

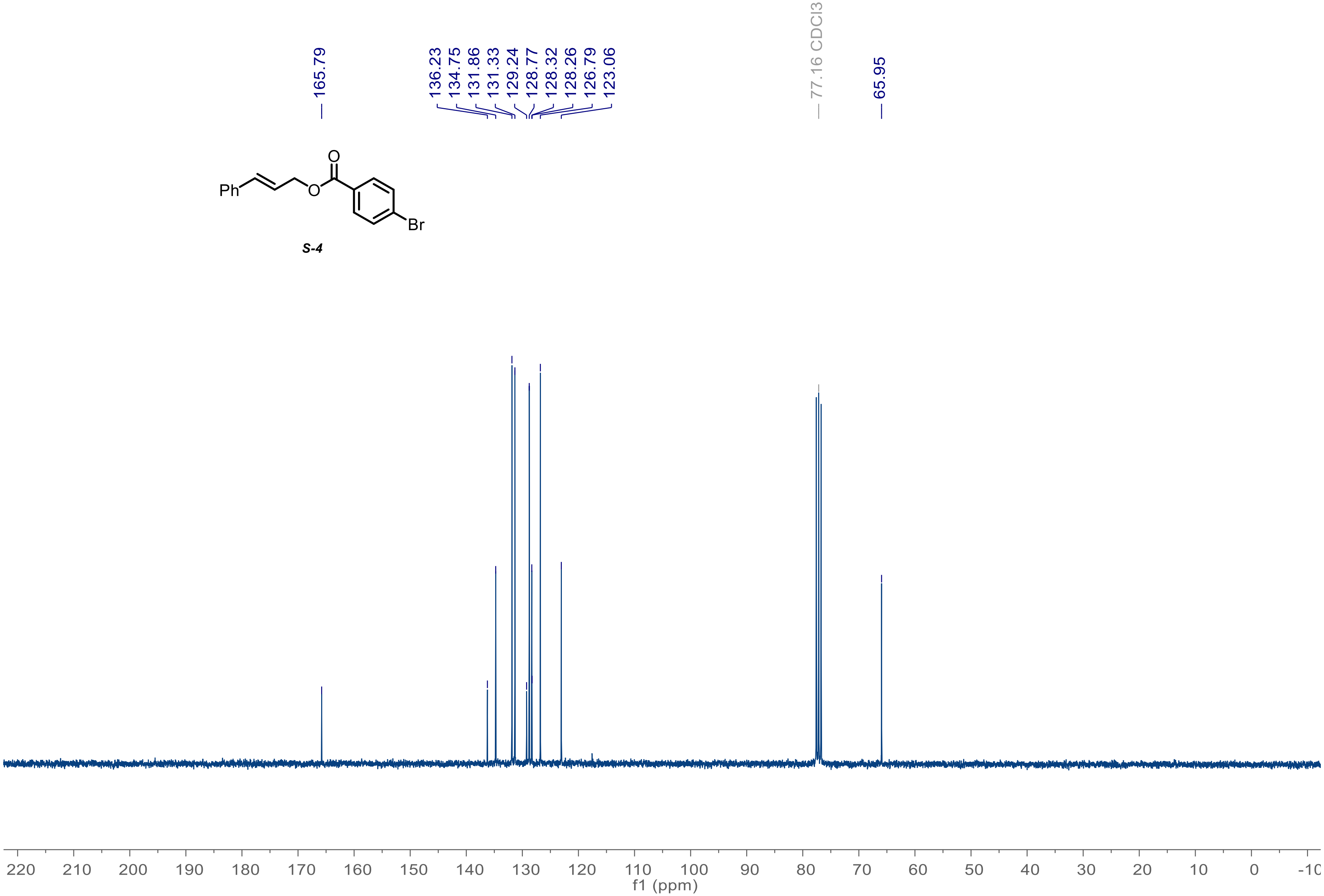

^{1}H NMR (300 MHz, $CDCl_3$) of **S5**

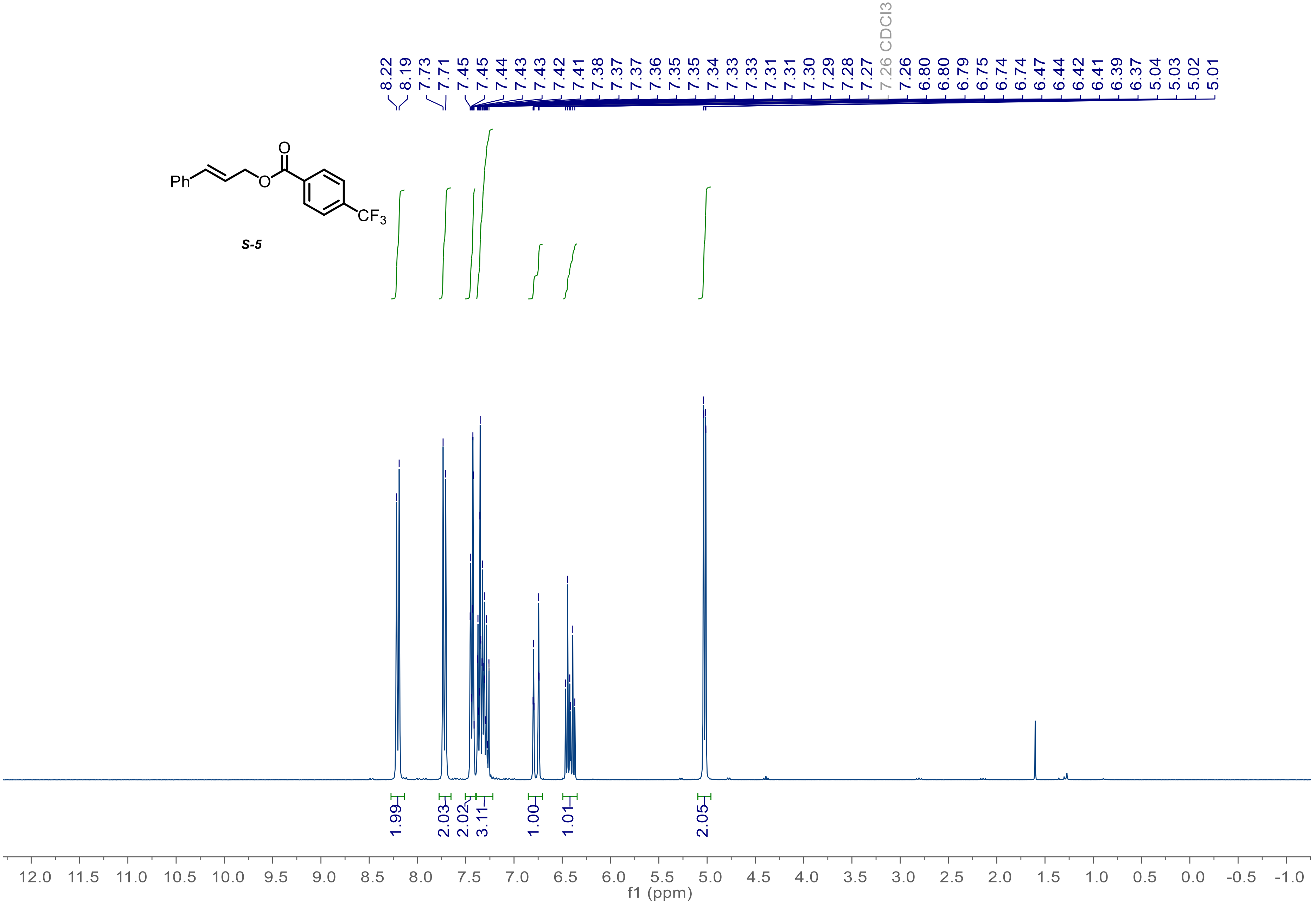

^{13}C NMR (75 MHz, $CDCl_3$) of **S5**

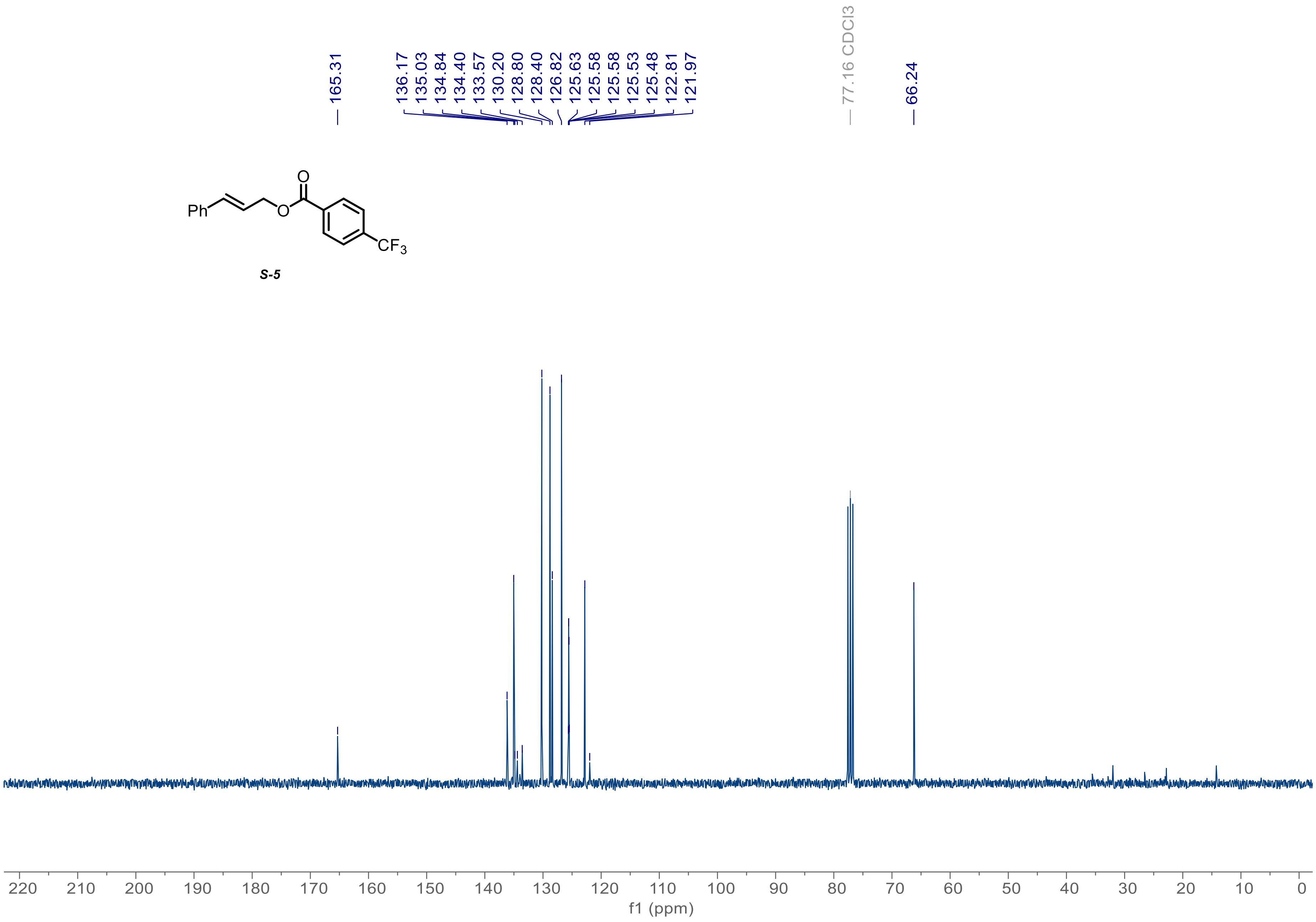

^{19}F NMR (282 MHz, $CDCl_3$) of **S5**

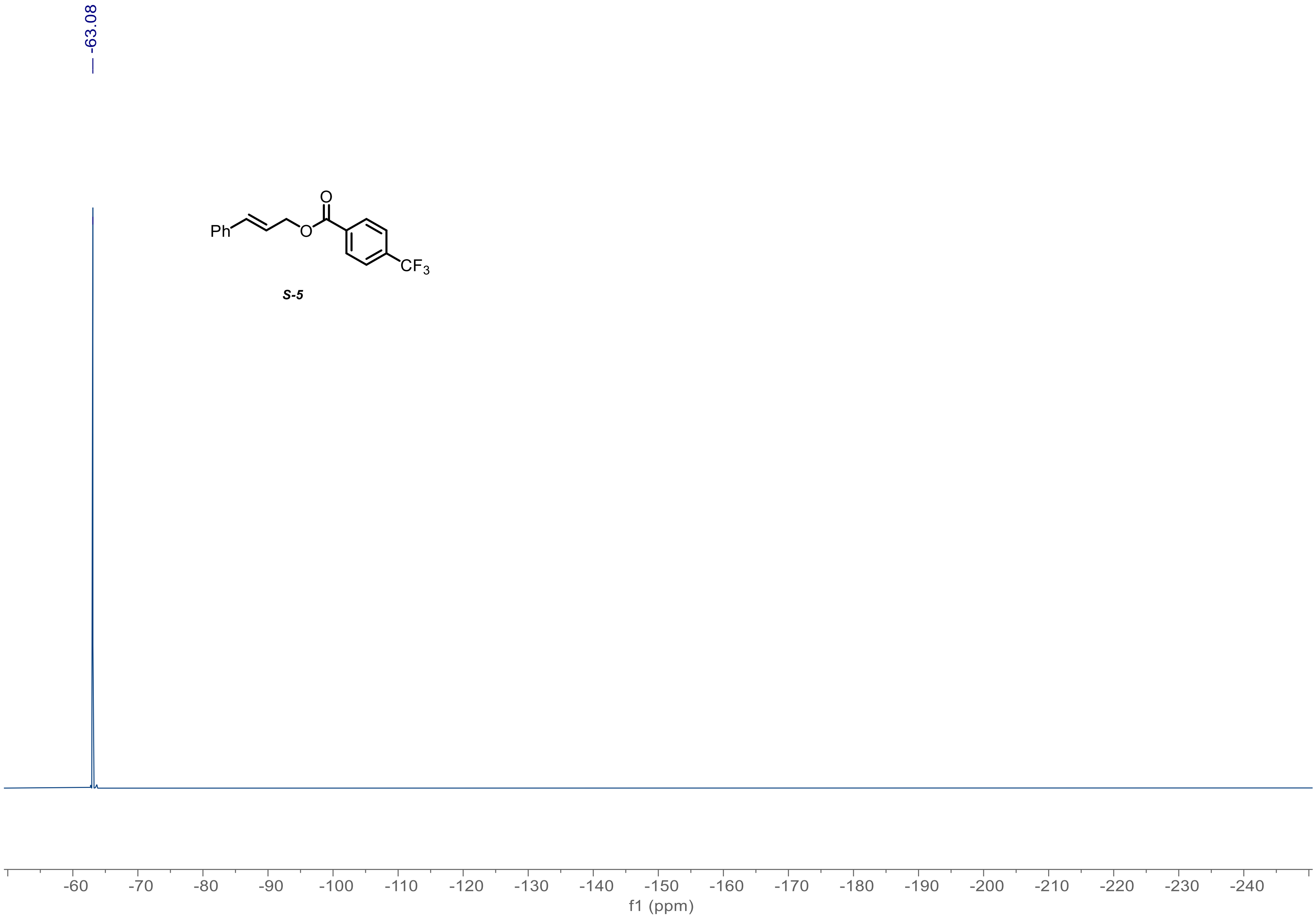

^{1}H NMR (300 MHz, $CDCl_3$) of **S6**

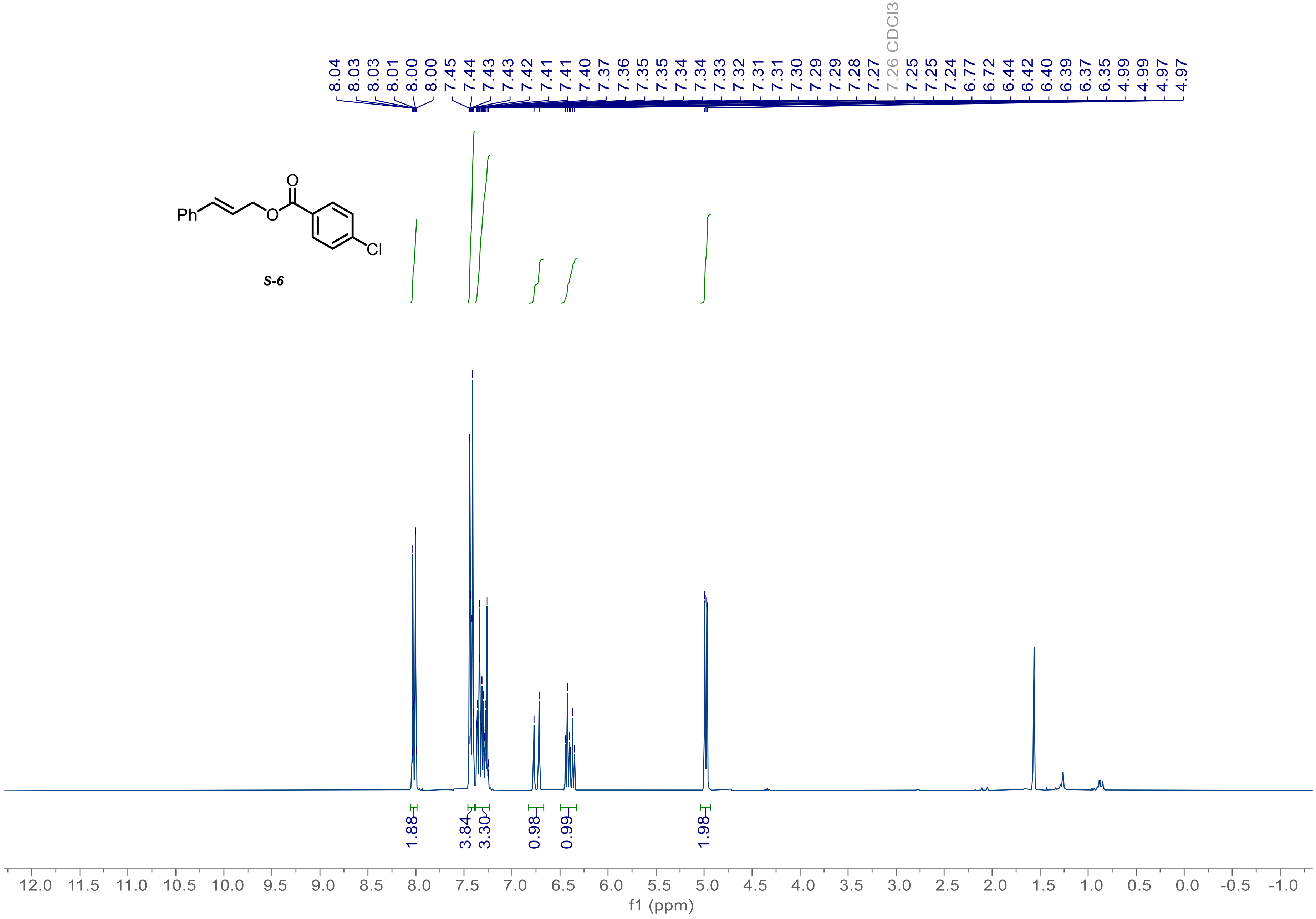

^{13}C NMR (75 MHz, $CDCl_3$) of **S6**

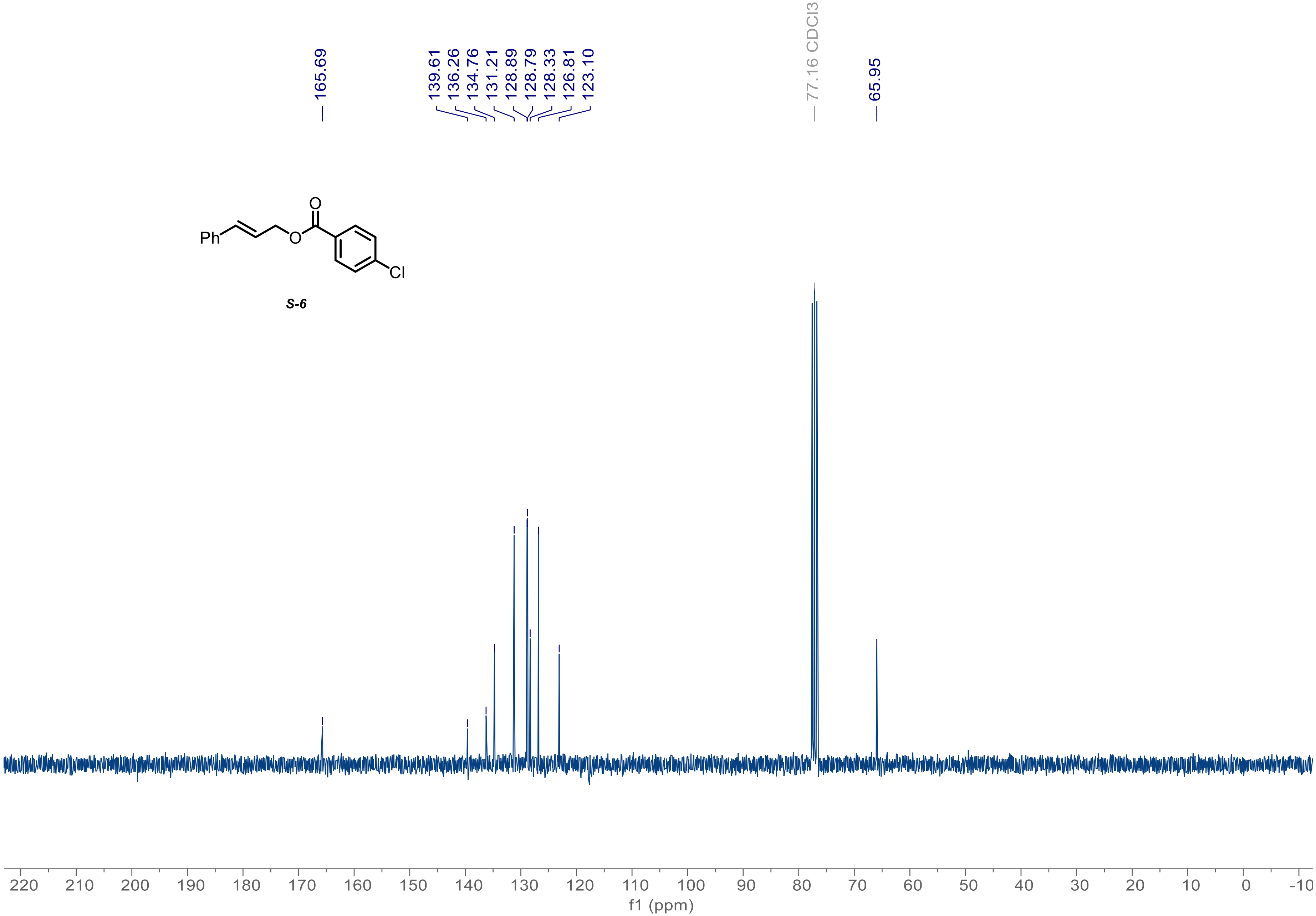

^{1}H NMR (300 MHz, $CDCl_3$) of **S7**

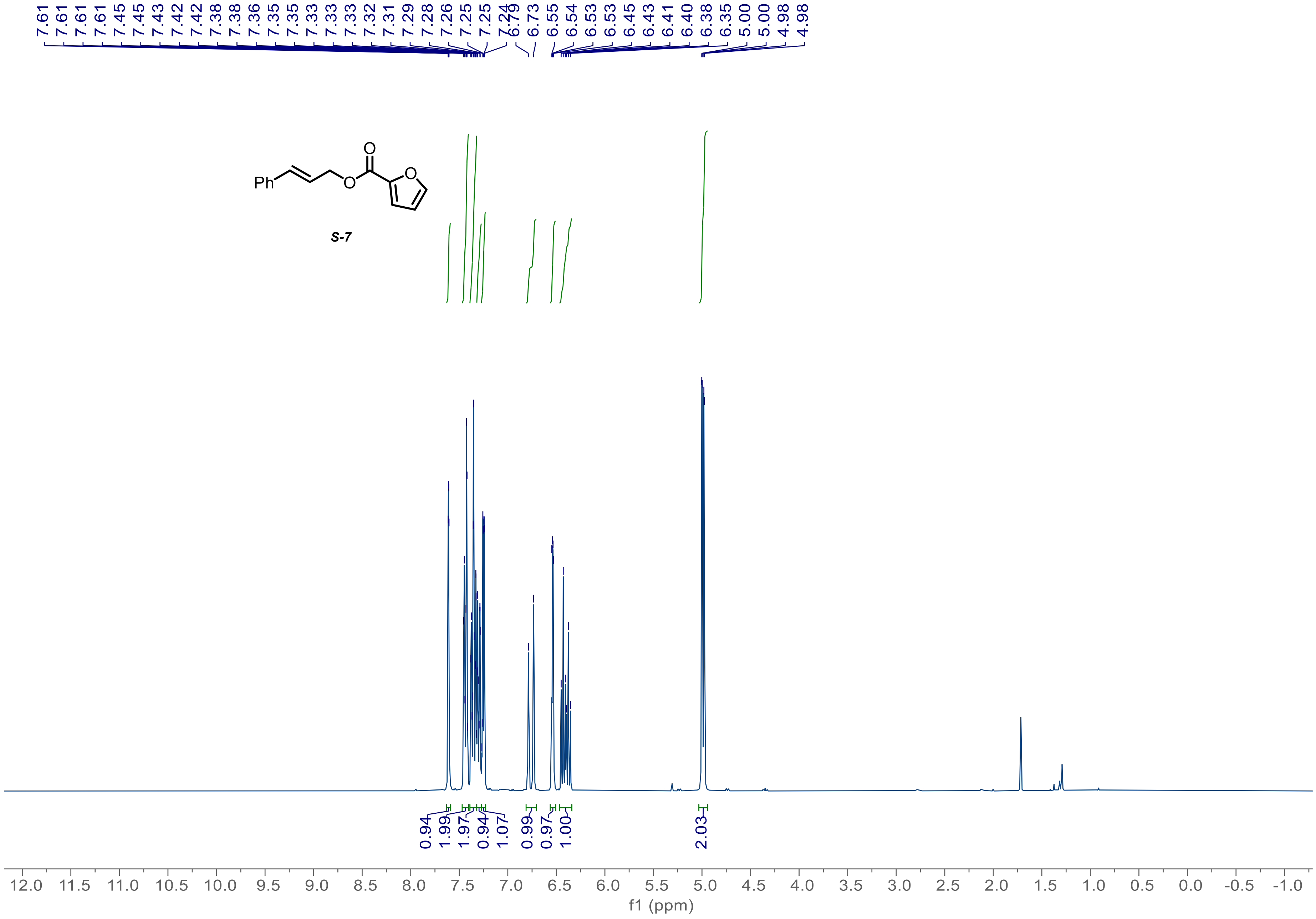

^{13}C NMR (75 MHz, $CDCl_3$) of **S7**

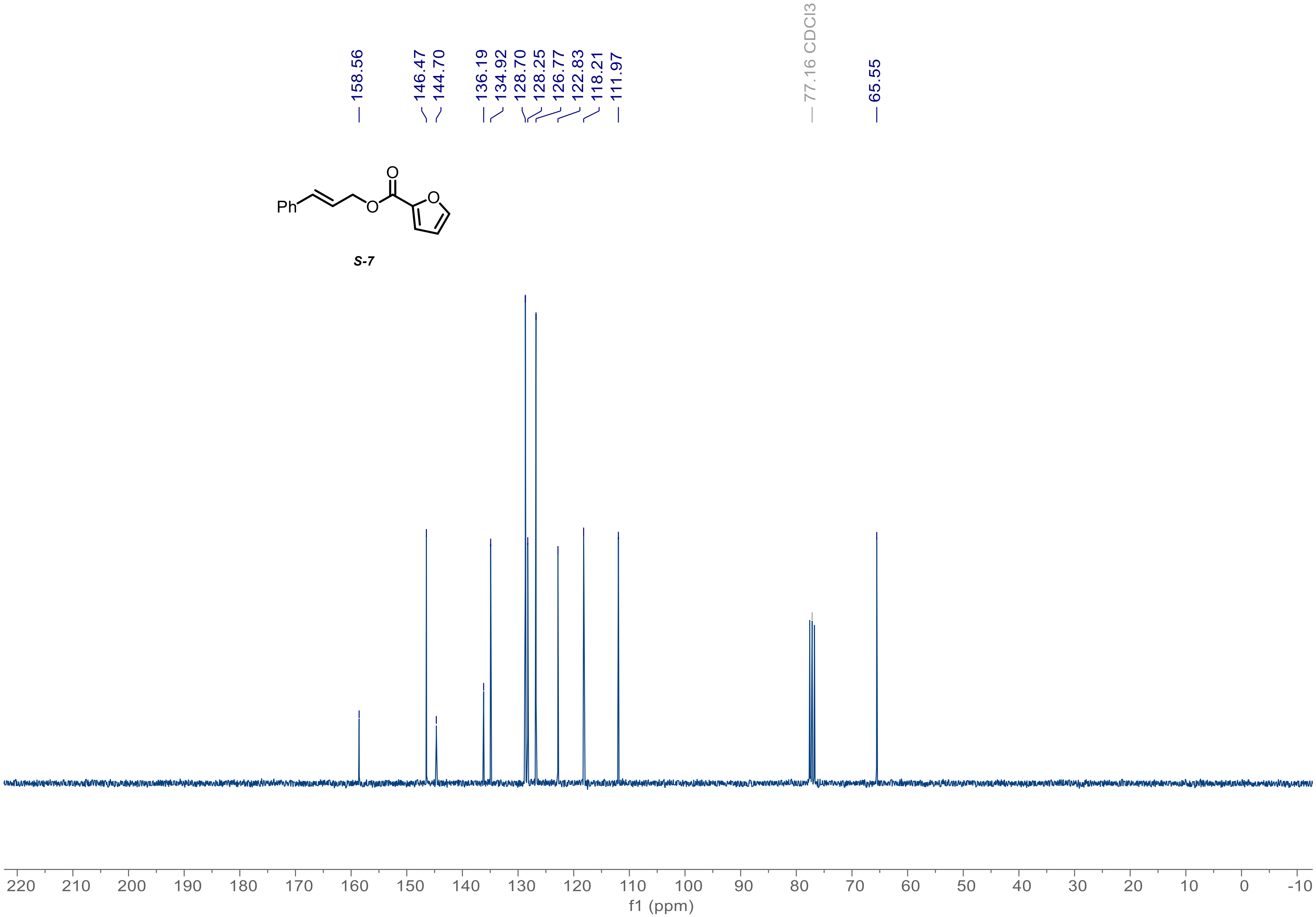

^{1}H NMR (300 MHz, $CDCl_3$) of **S8**

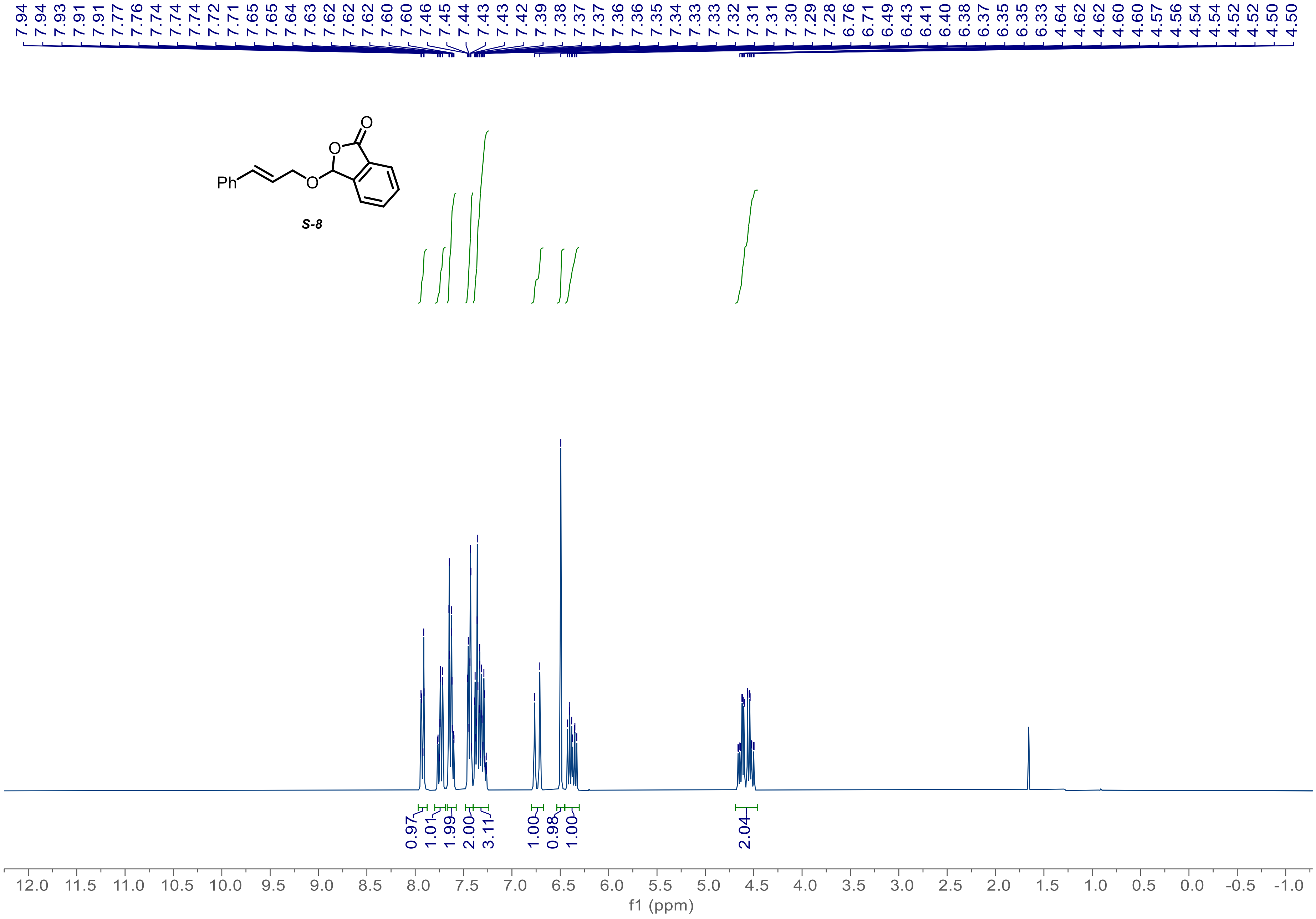

^{13}C NMR (75 MHz, $CDCl_3$) of **S8**

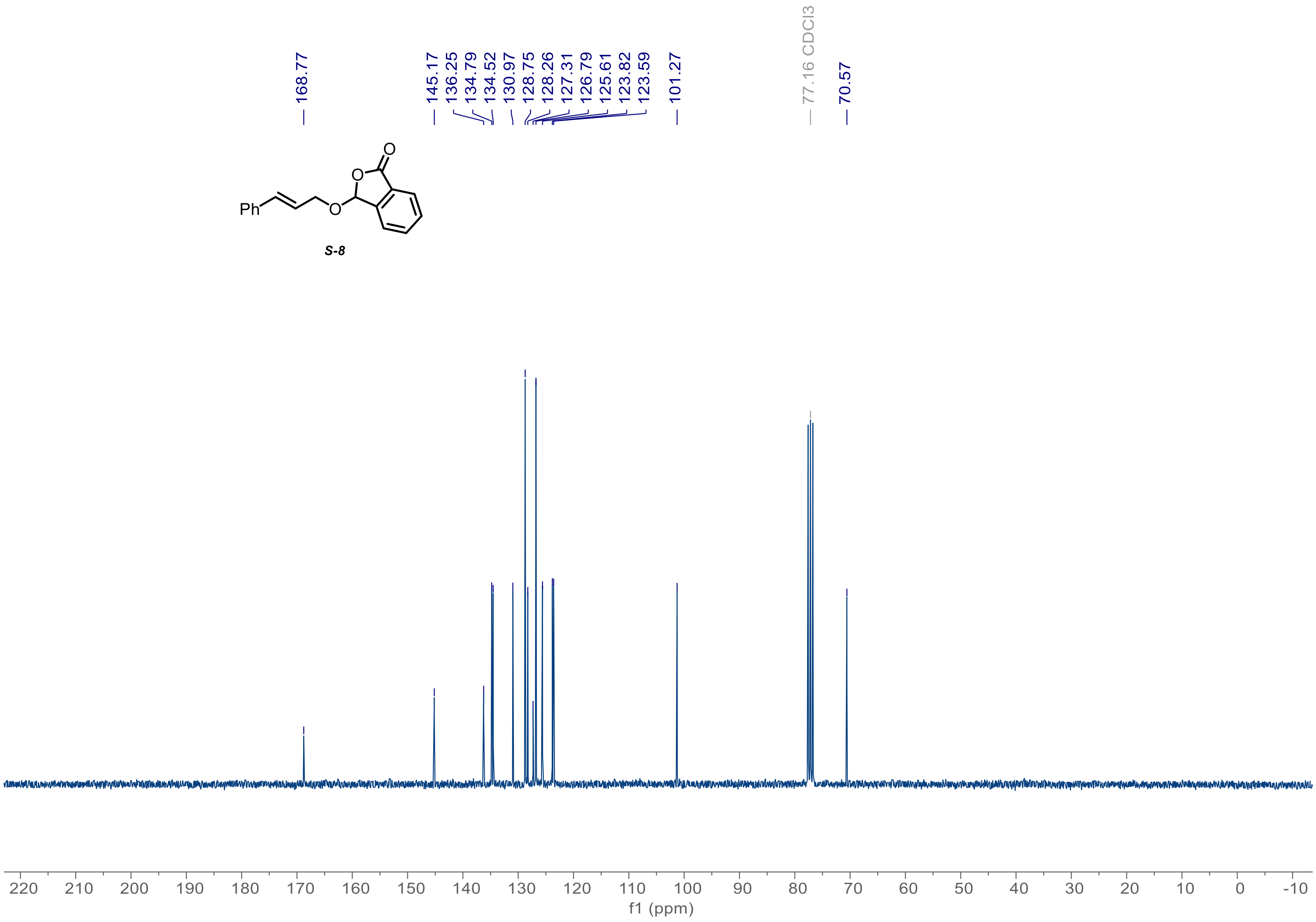

^{1}H NMR (300 MHz, $CDCl_3$) of **S9**

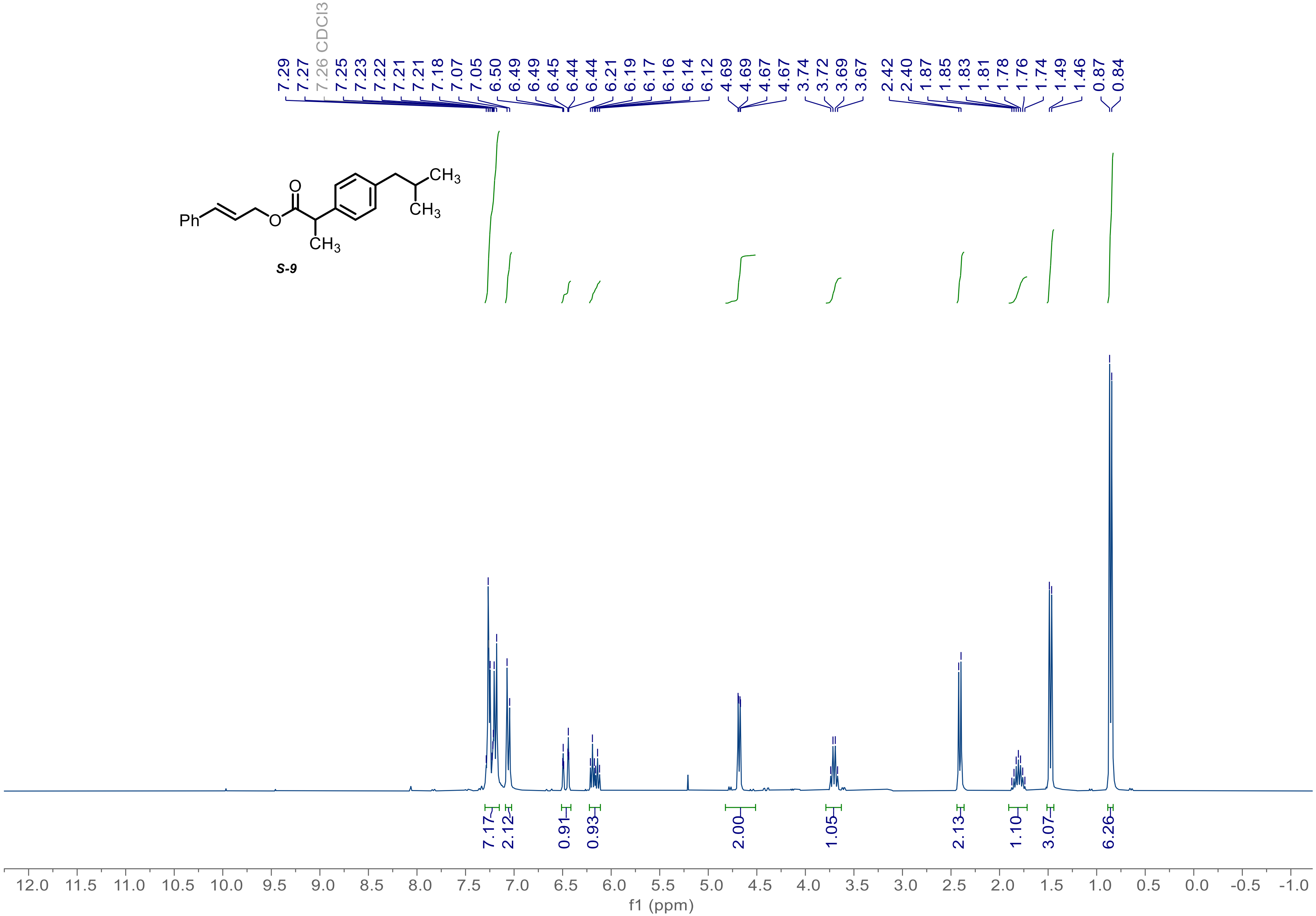

^{13}C NMR (75 MHz, $CDCl_3$) of **S9**

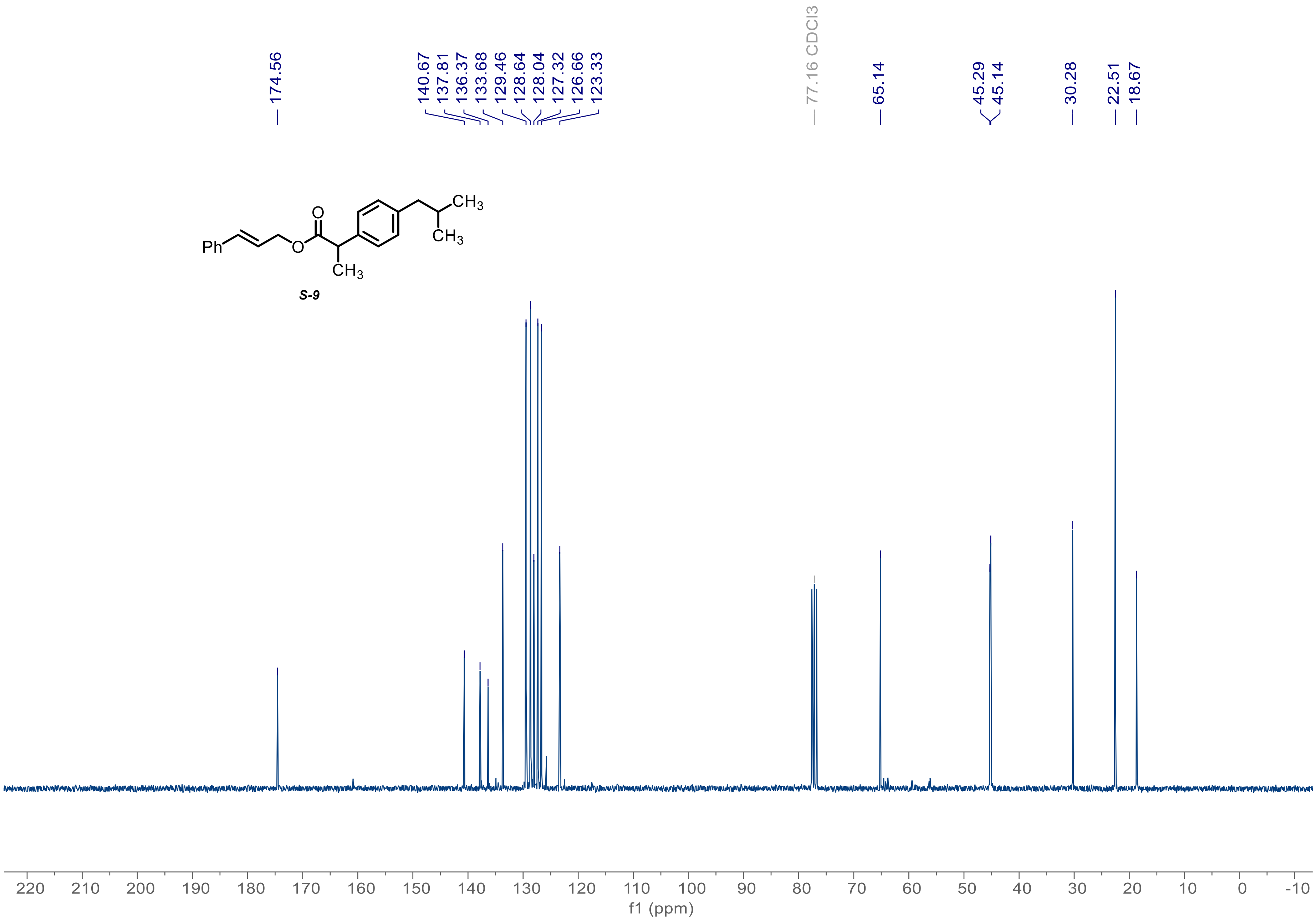

^{1}H NMR (300 MHz, $CDCl_3$) of **S10**

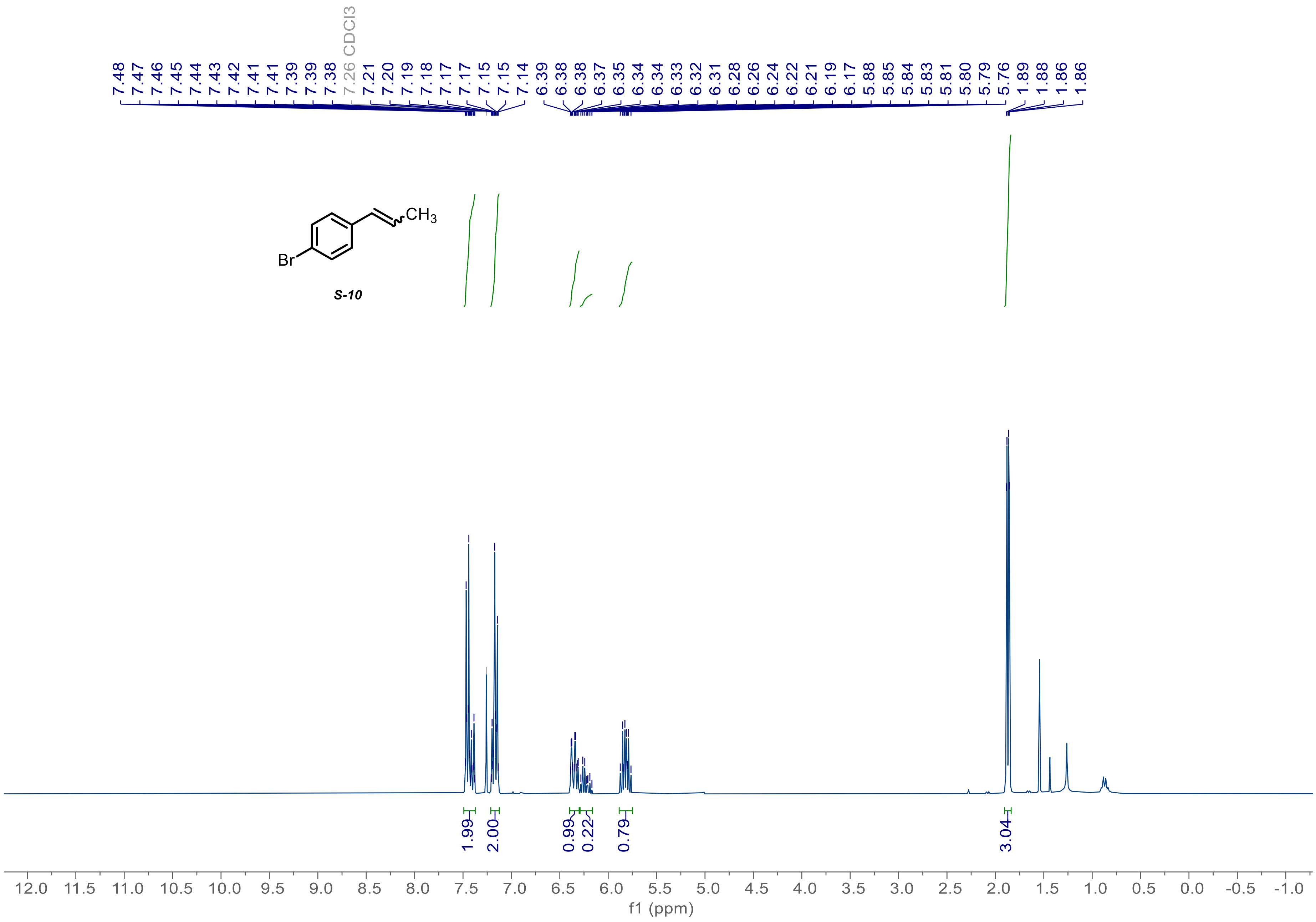

^{13}C NMR (75 MHz, $CDCl_3$) of **S10**

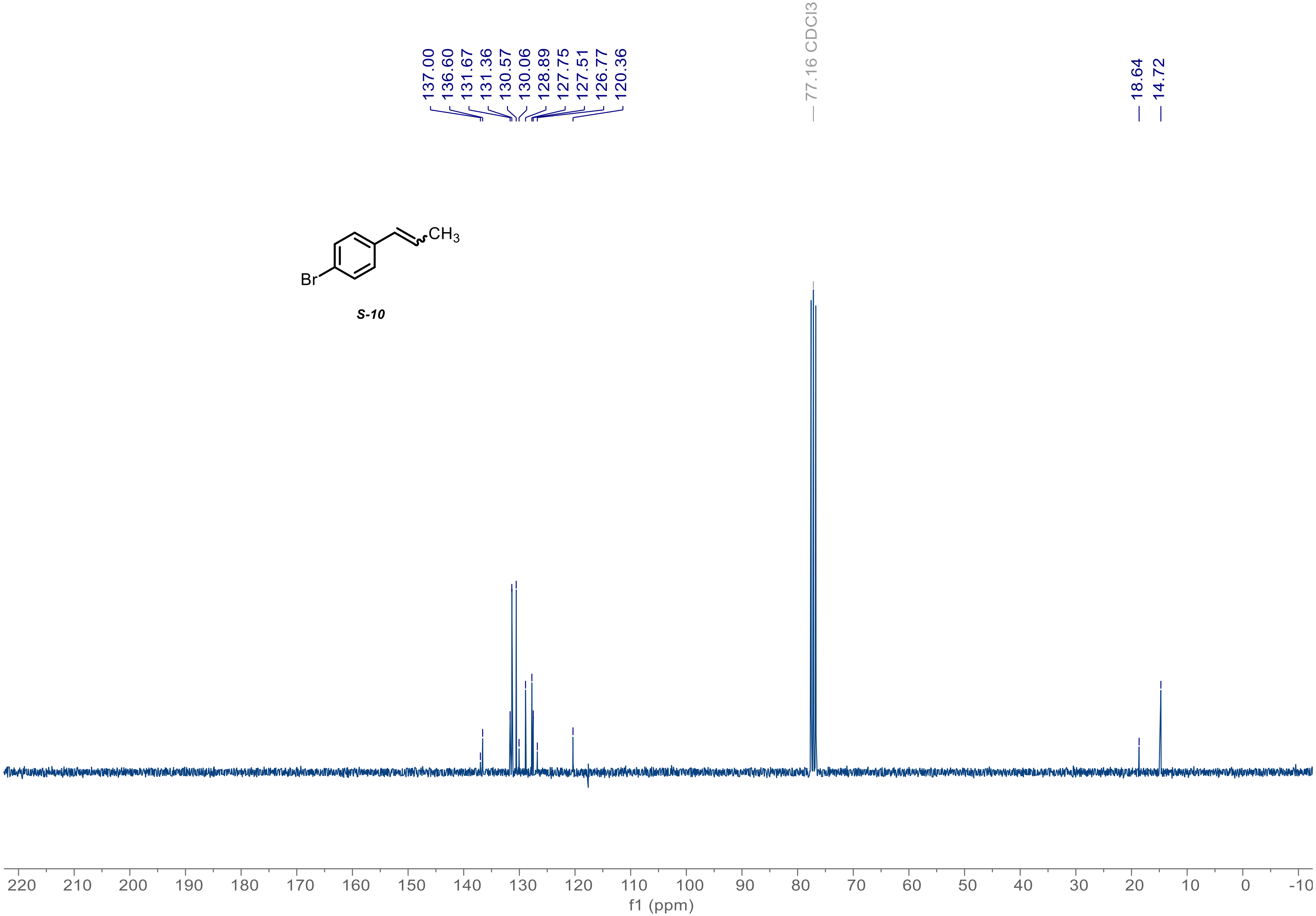

^{1}H NMR (300 MHz, $CDCl_3$) of **S11**

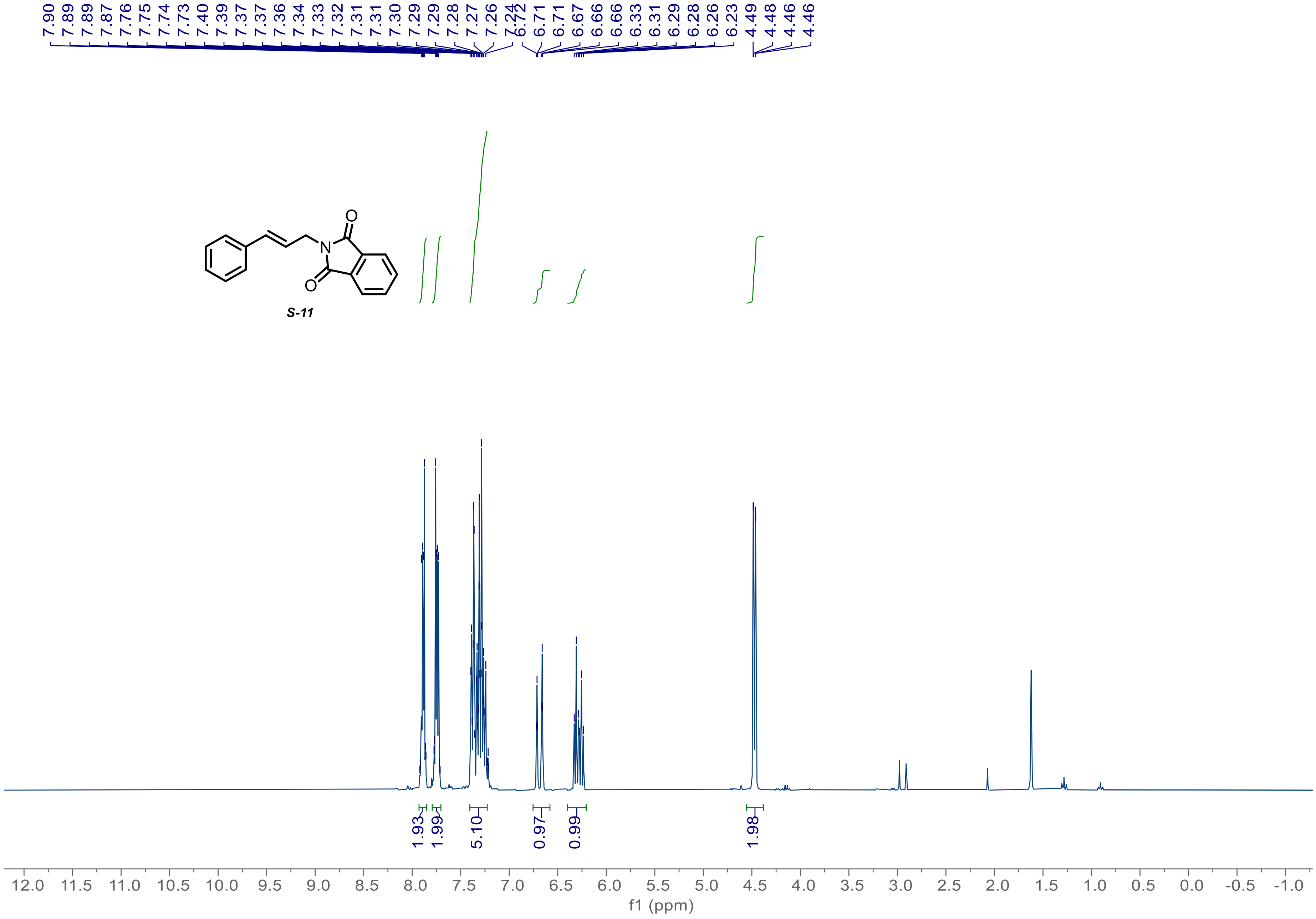

^{13}C NMR (75 MHz, $CDCl_3$) of **S11**

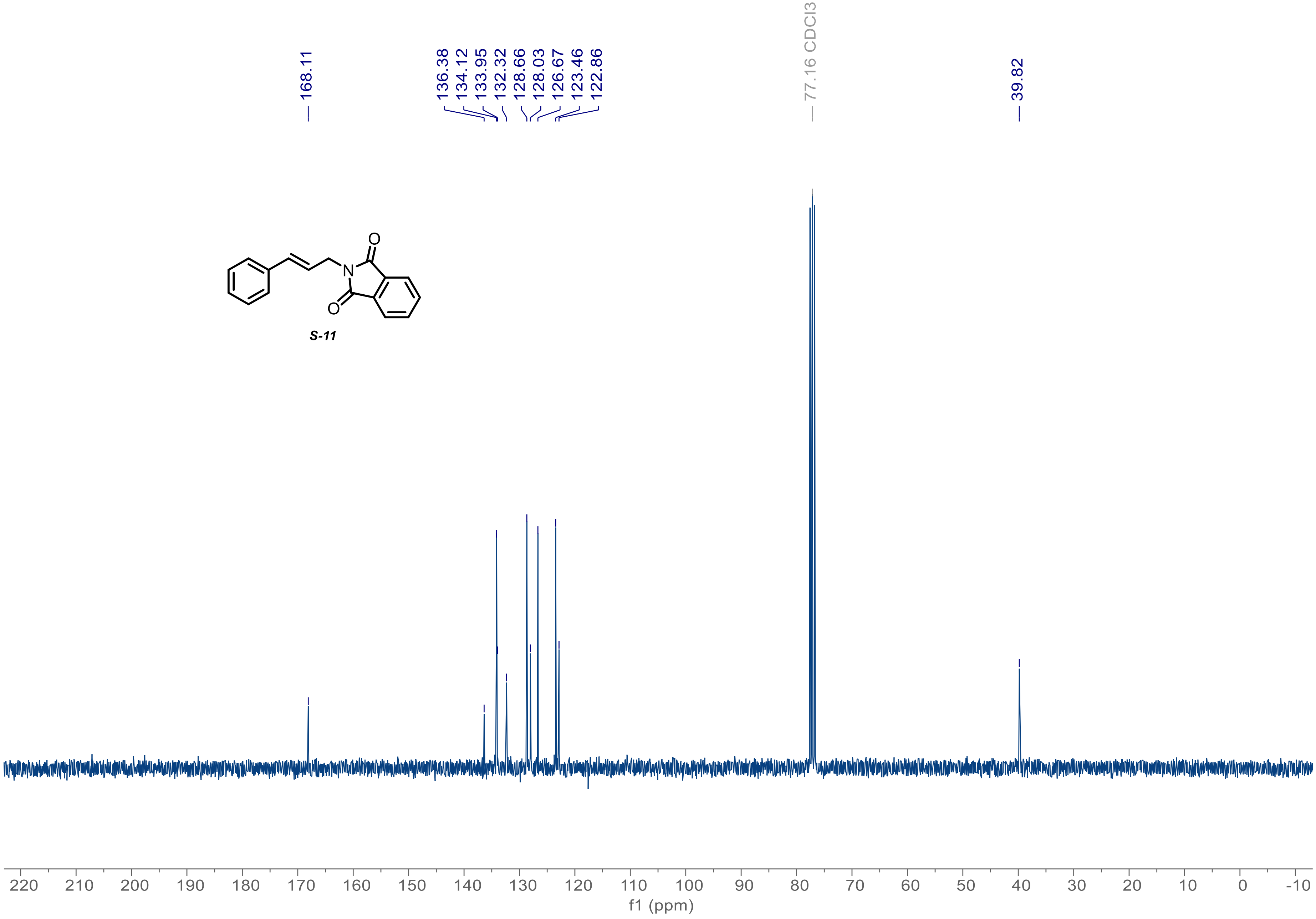

^{1}H NMR (300 MHz, $CDCl_3$) of **S12**

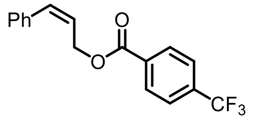

S-12

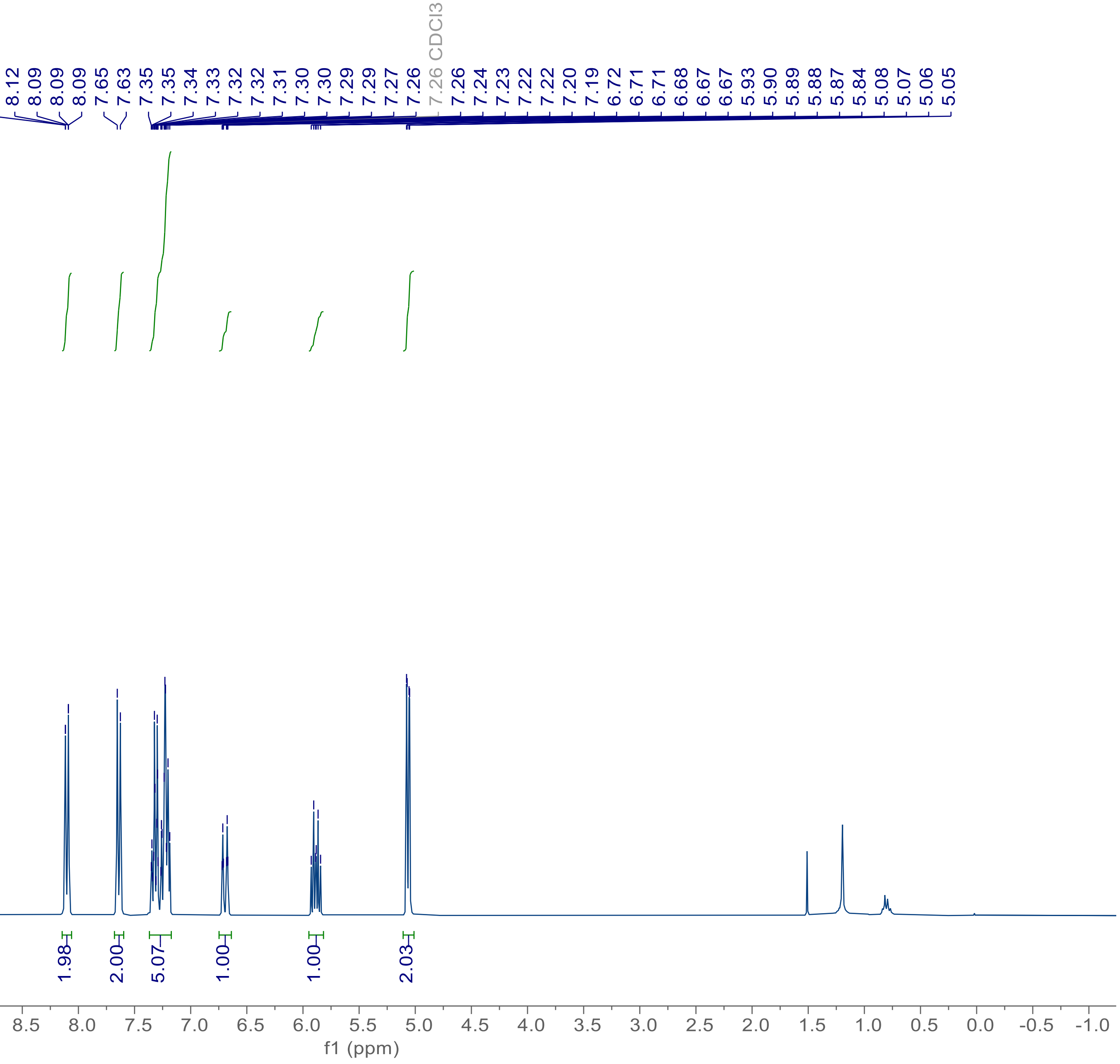

^{13}C NMR (75 MHz, $CDCl_3$) of **S12**

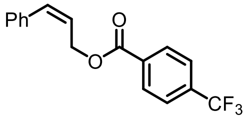

S-12

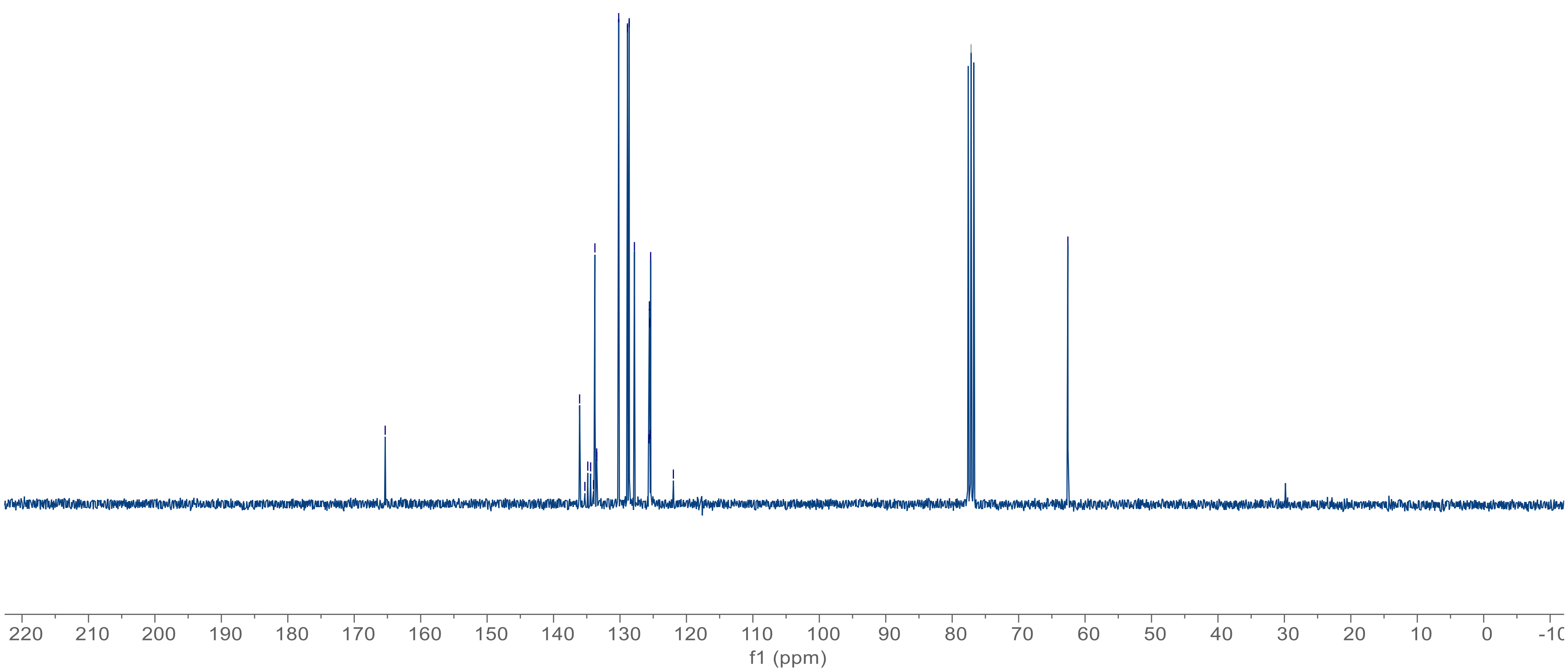

^{19}F NMR (282 MHz, $CDCl_3$) of **S12**

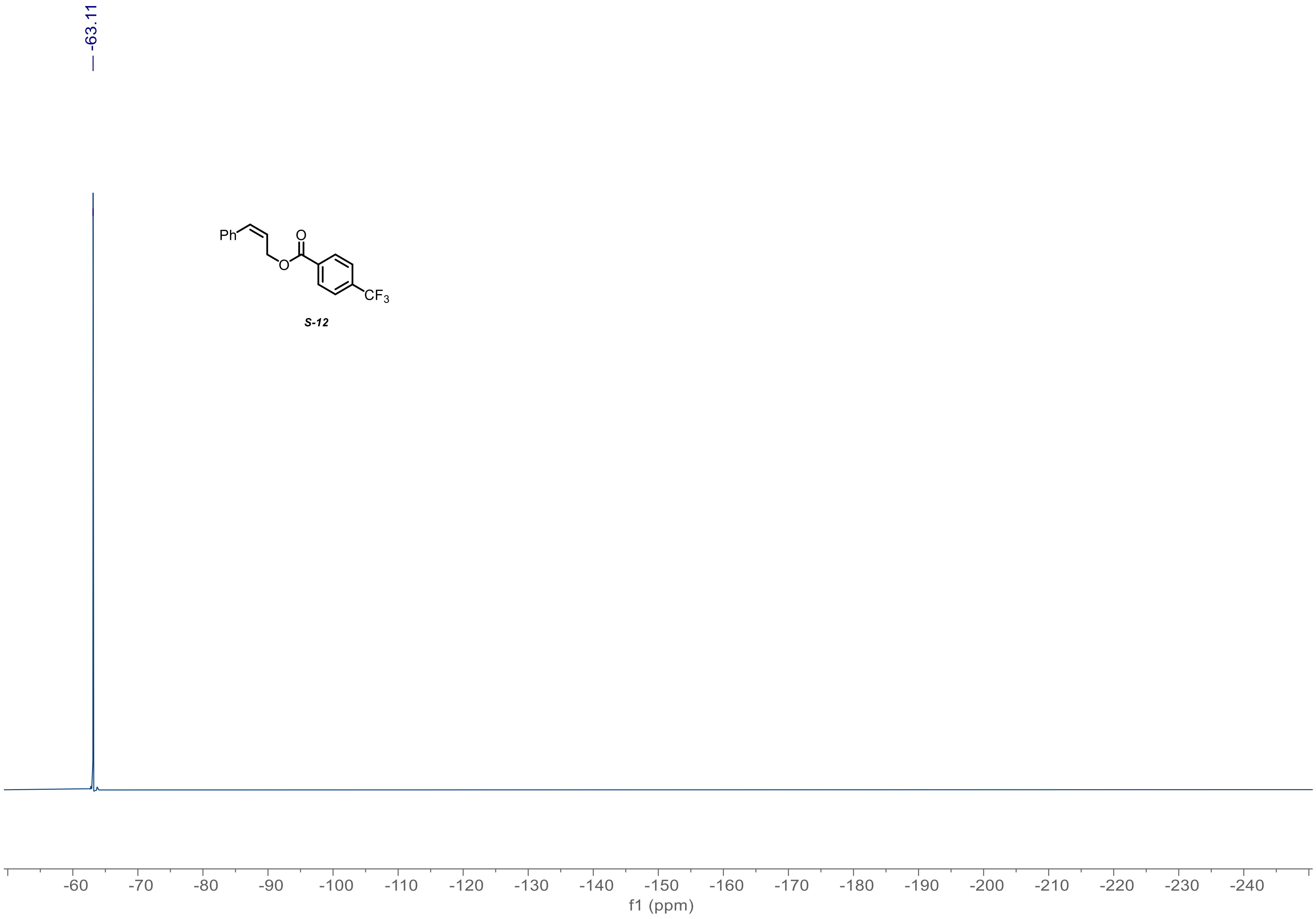

^{1}H NMR (300 MHz, $CDCl_3$) of **3a**

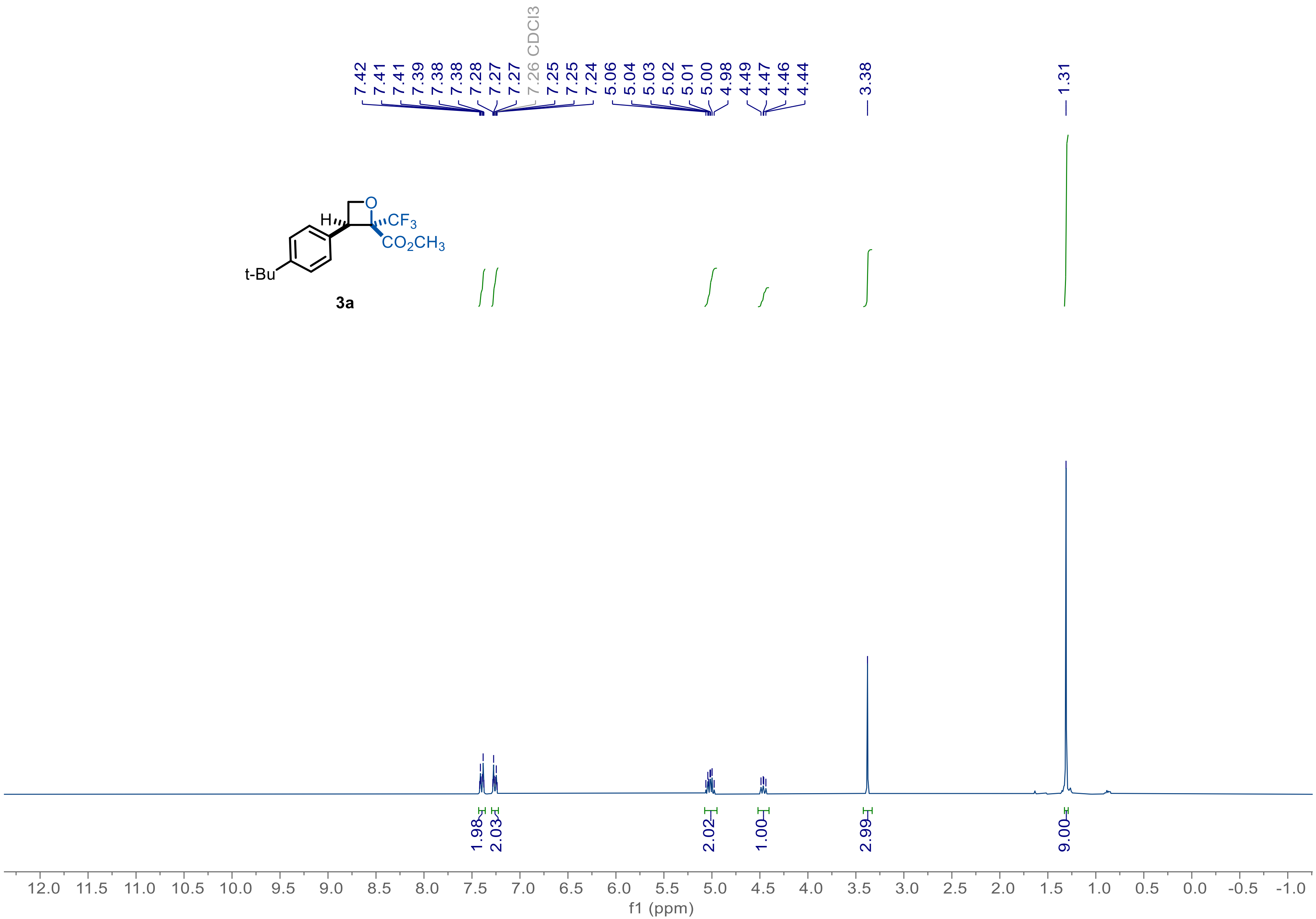

^{13}C NMR (75 MHz, $CDCl_3$) of **3a**

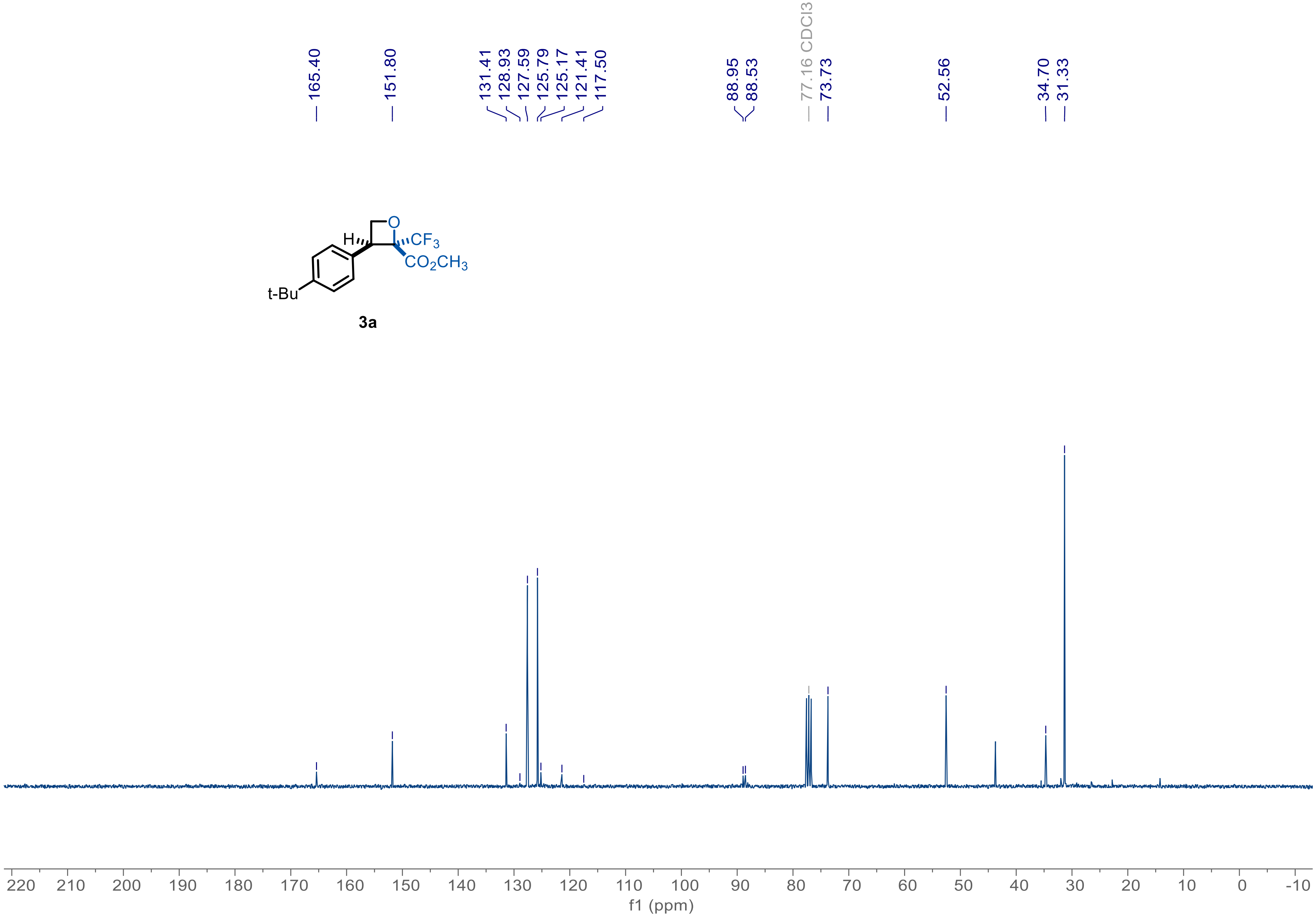

^{19}F NMR (282 MHz, $CDCl_3$) of **3a**

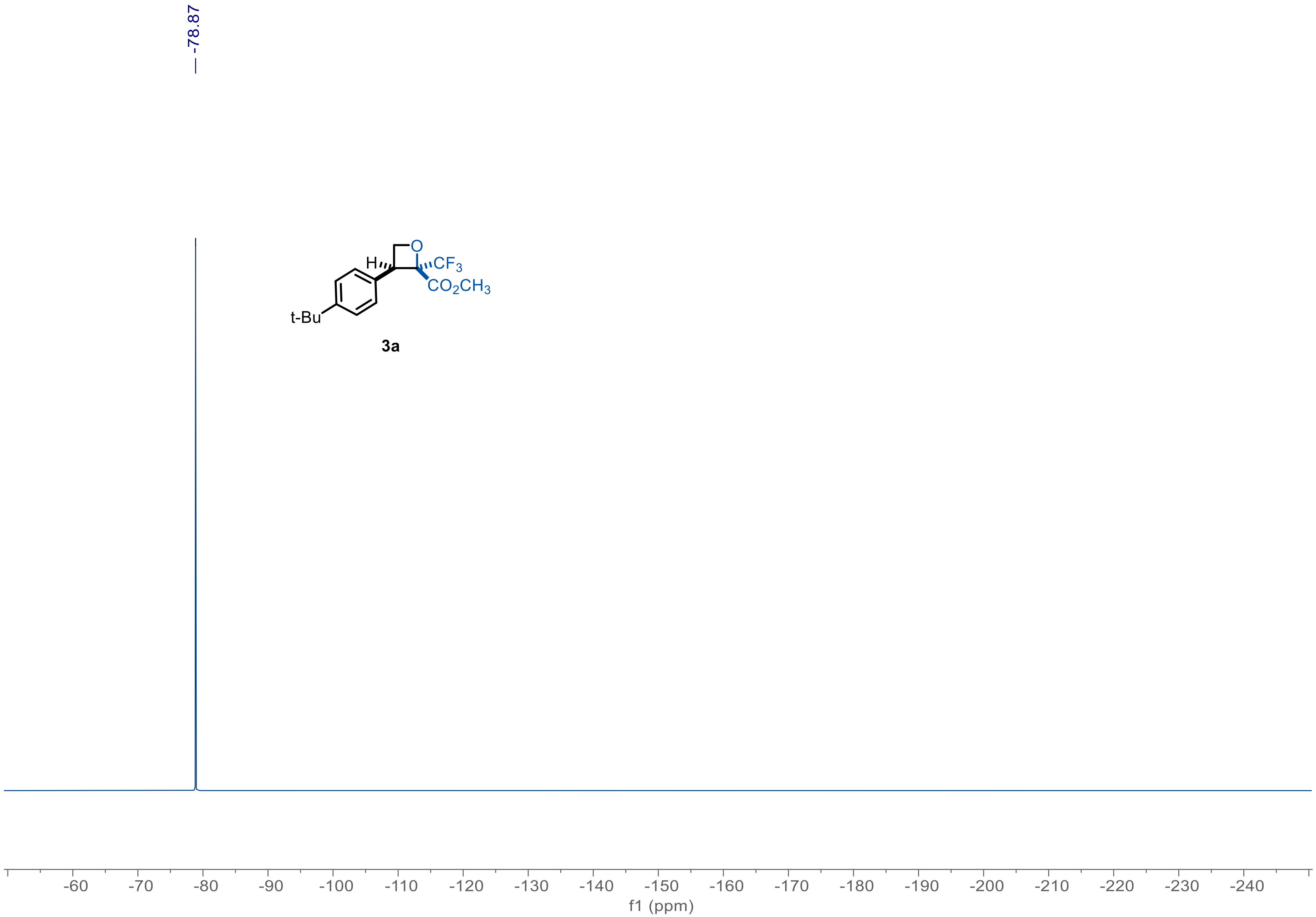

^{1}H NMR (300 MHz, $CDCl_3$) of **3a'**

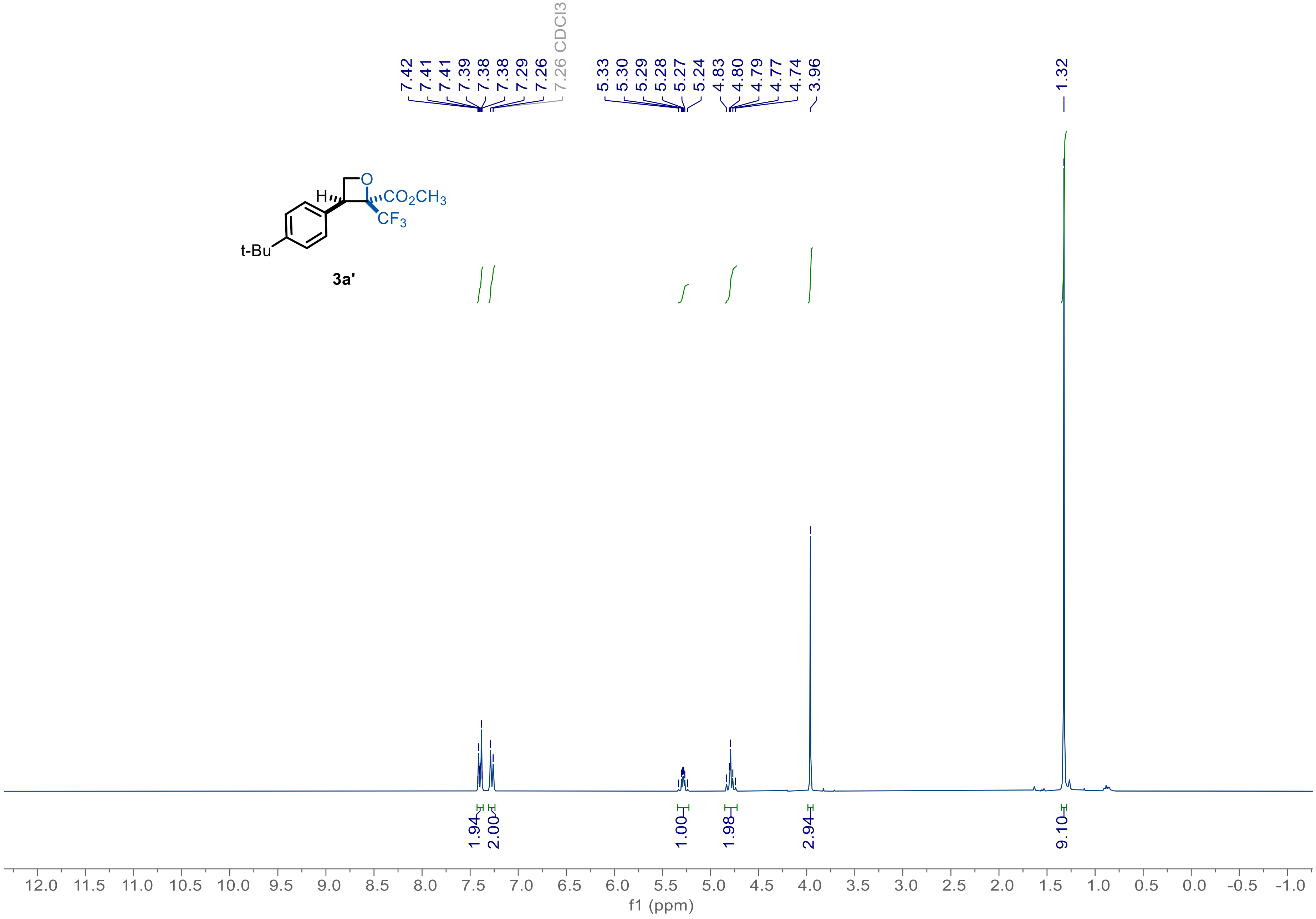

^{13}C NMR (75 MHz, $CDCl_3$) of **3a'**

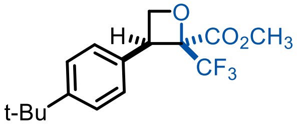

3a'

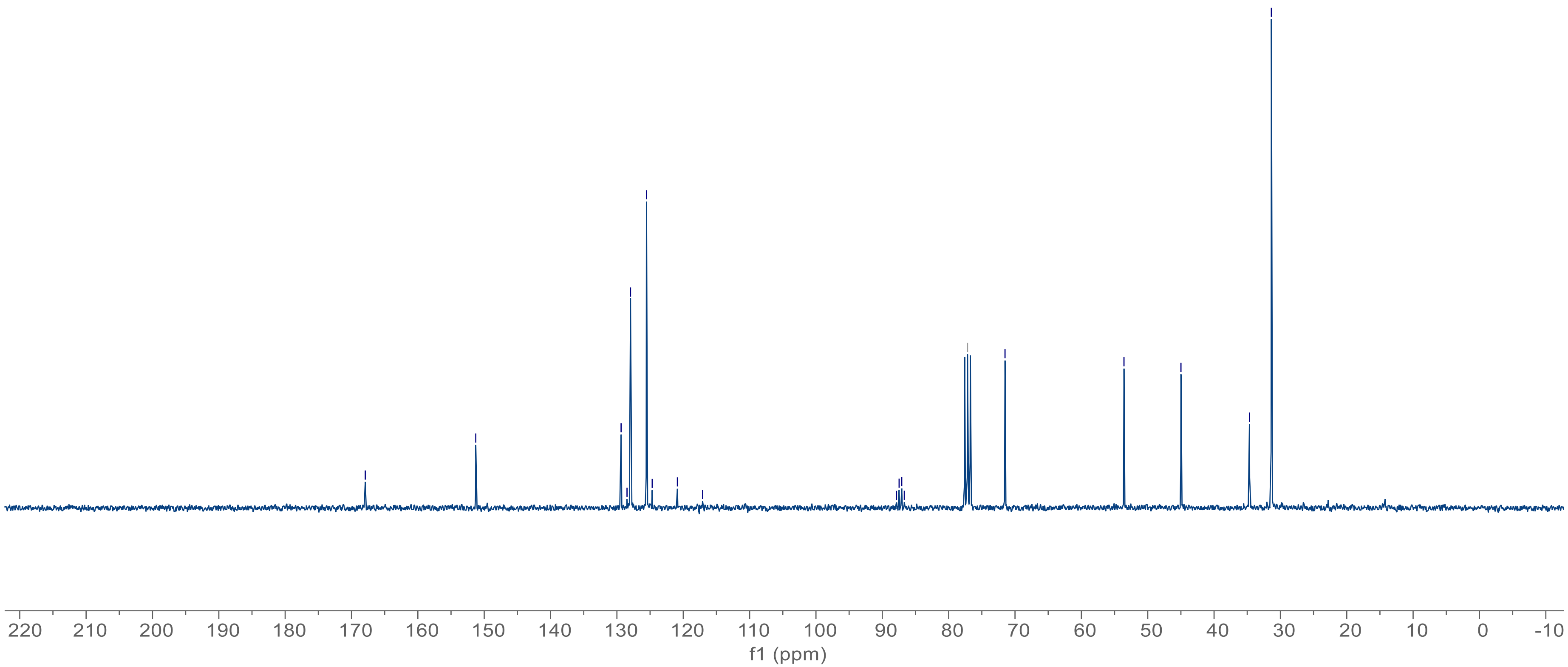

^{19}F NMR (282 MHz, $CDCl_3$) of **3a'**

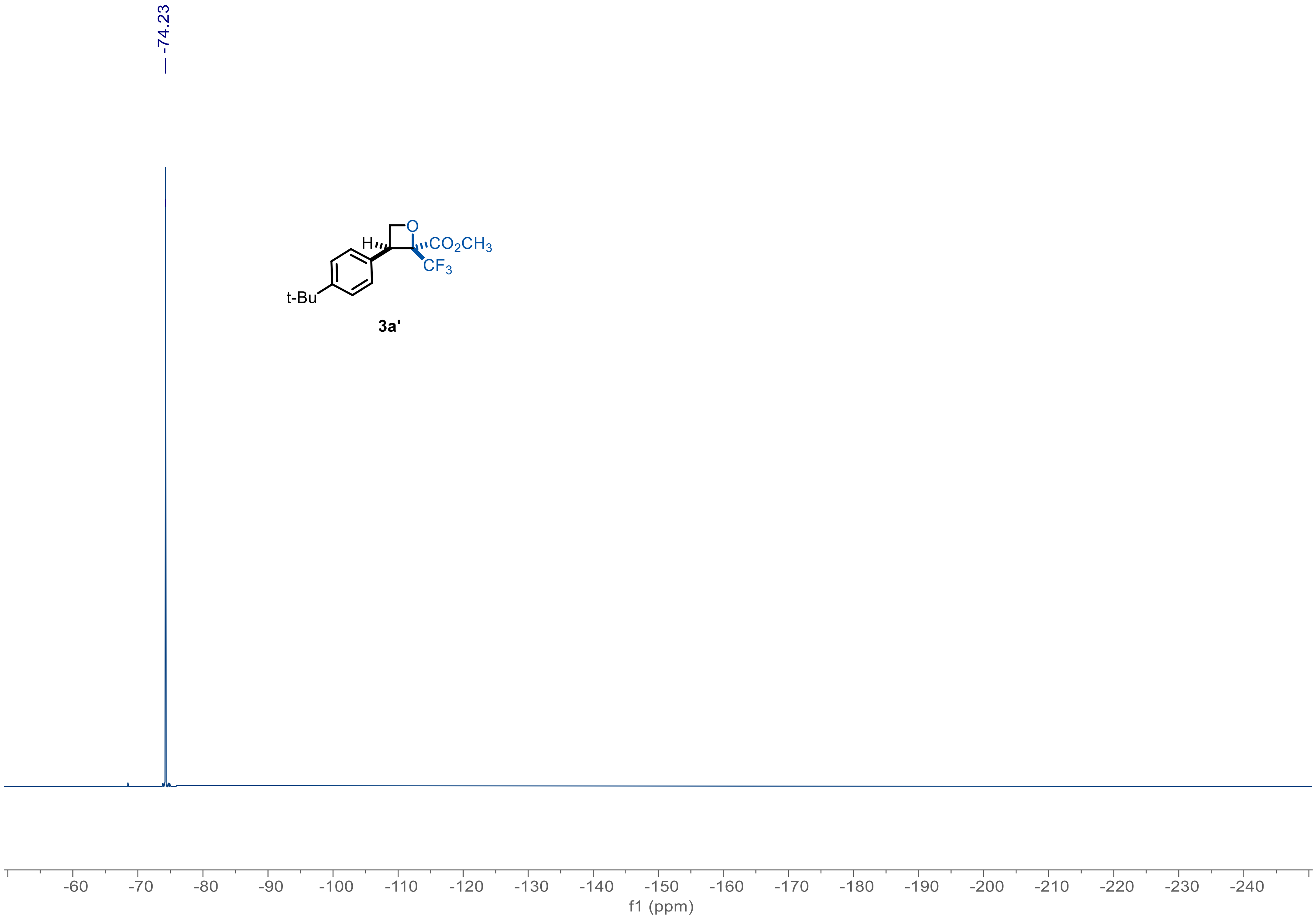

^{1}H NMR (300 MHz, $CDCl_3$) of **3b**

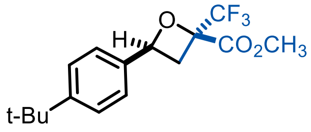

3b

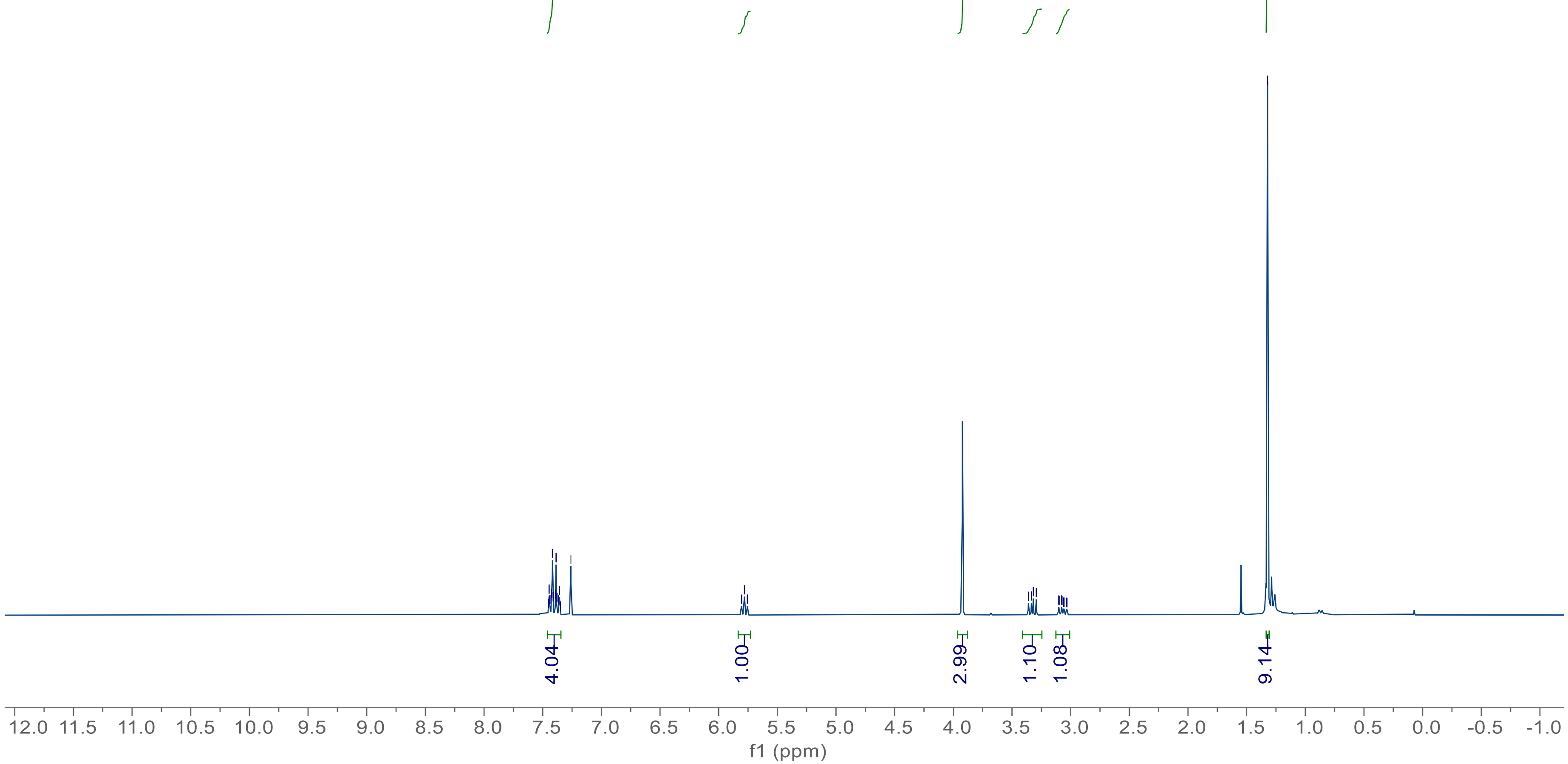

^{13}C NMR (101 MHz, $CDCl_3$) of **3b**

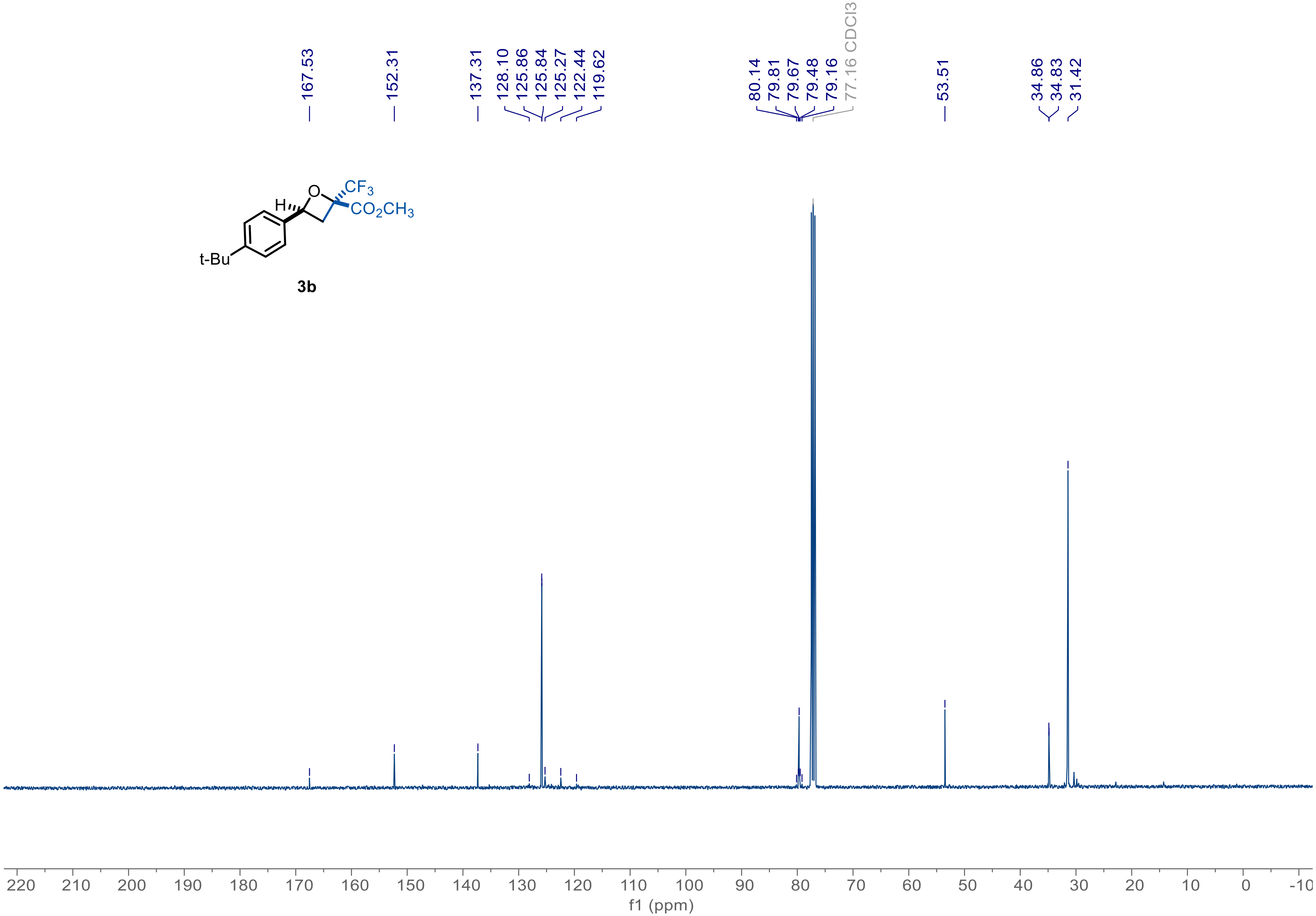

^{19}F NMR (282 MHz, $CDCl_3$) of **3b**

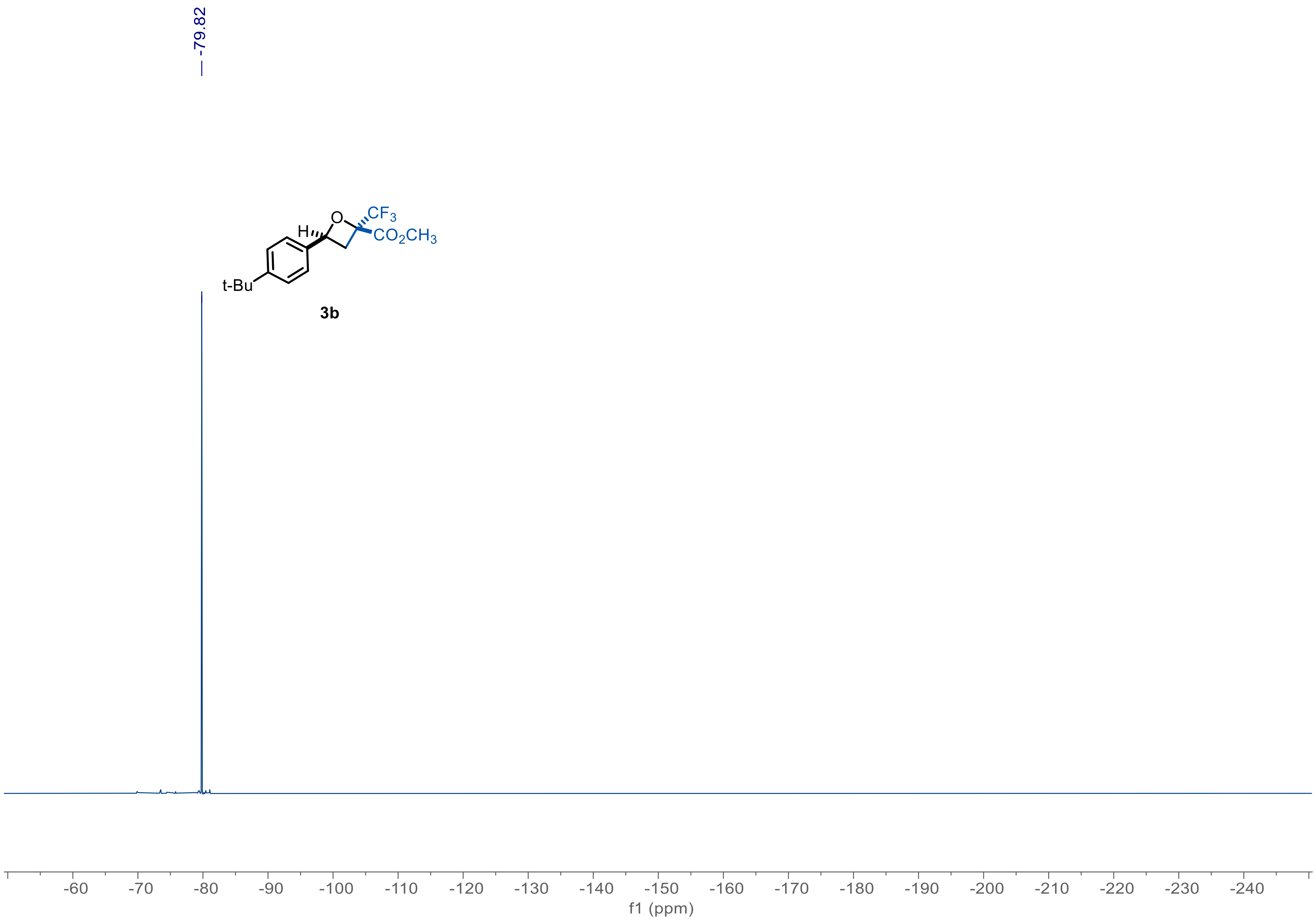

^{1}H NMR (300 MHz, $CDCl_3$) of **3b'**

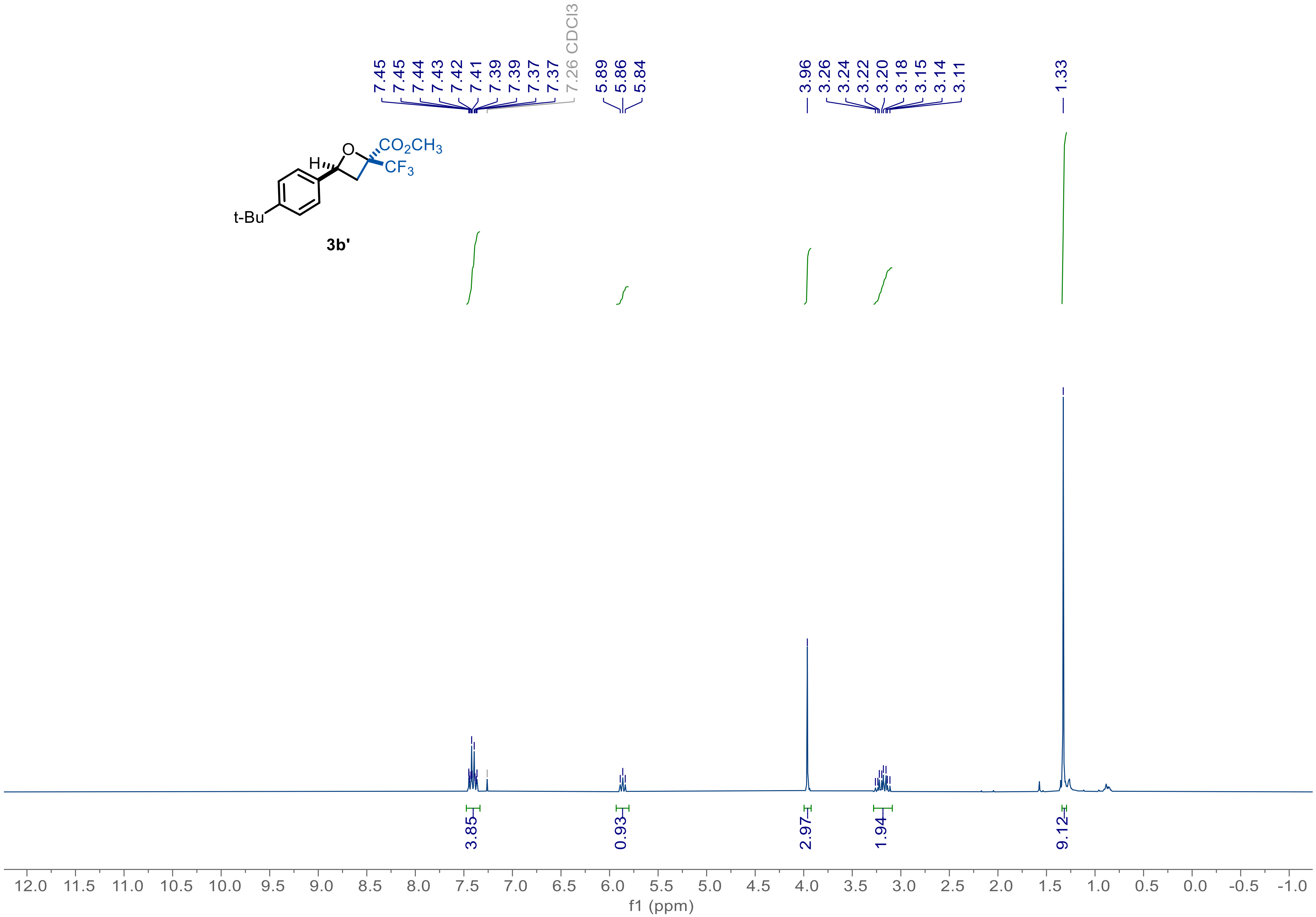

^{13}C NMR (75 MHz, $CDCl_3$) of **3b'**

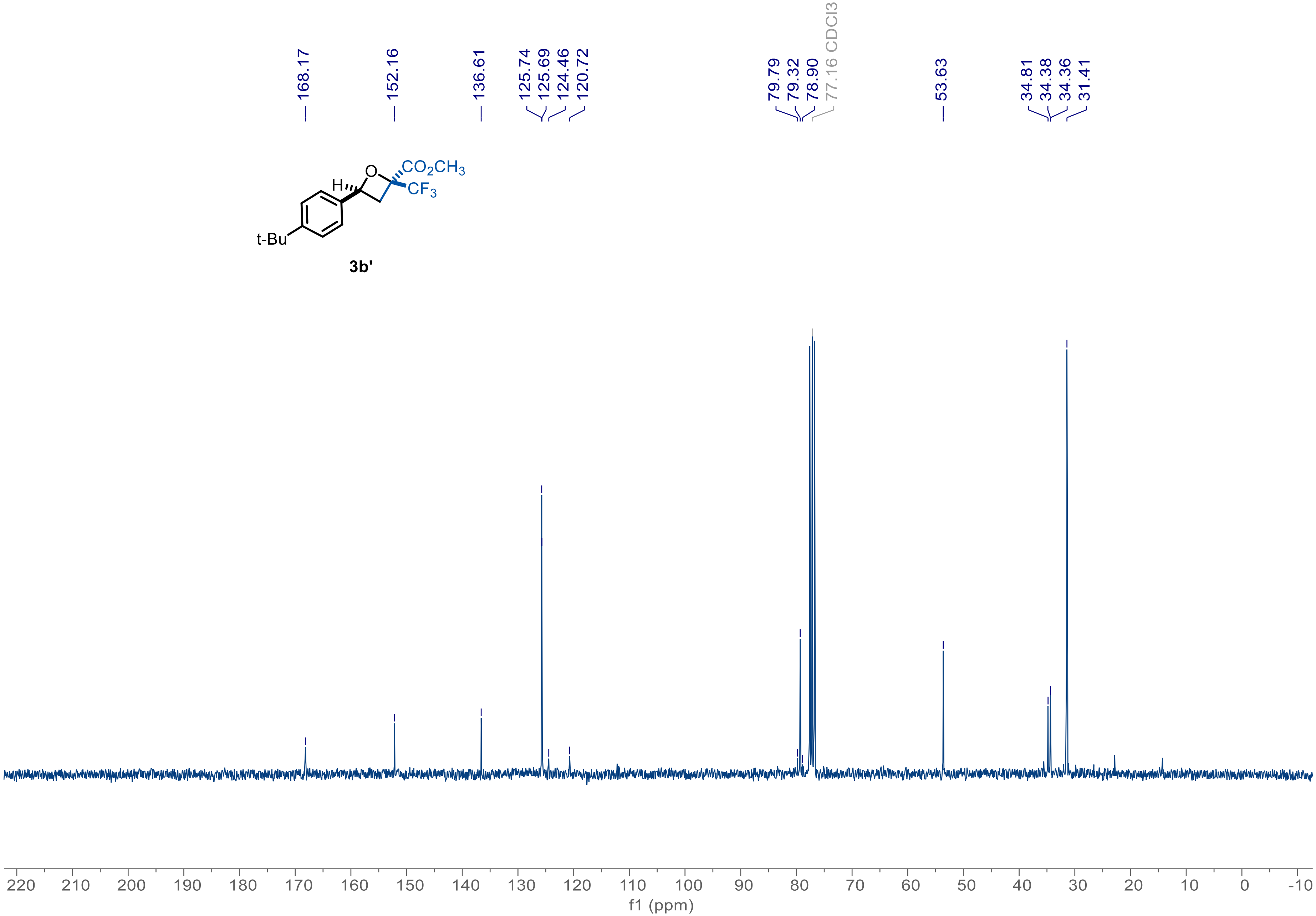

^{19}F NMR (282 MHz, $CDCl_3$) of **3b’**

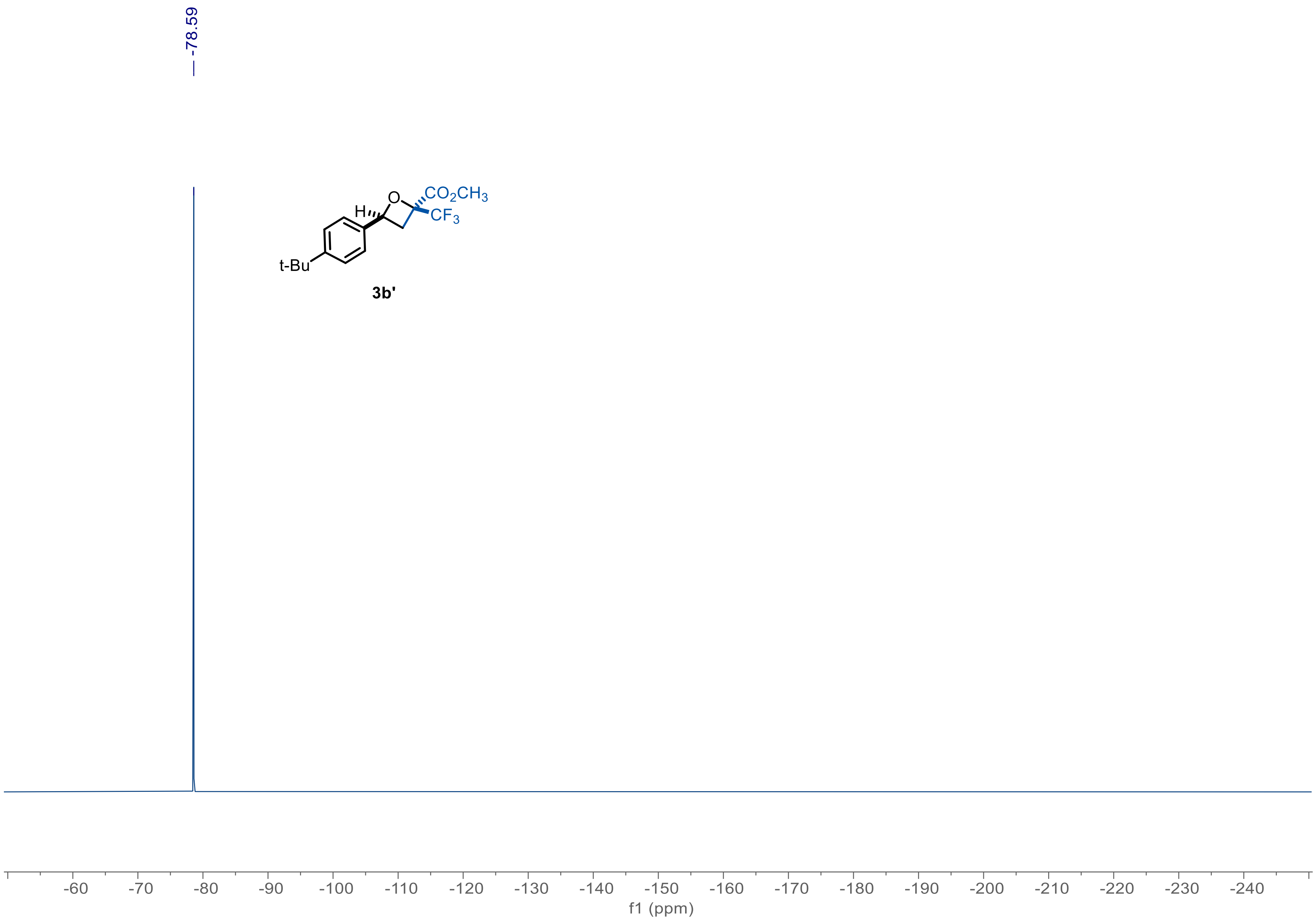

^{1}H NMR (300 MHz, $CDCl_3$) of **4b**

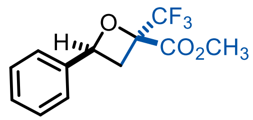

4b

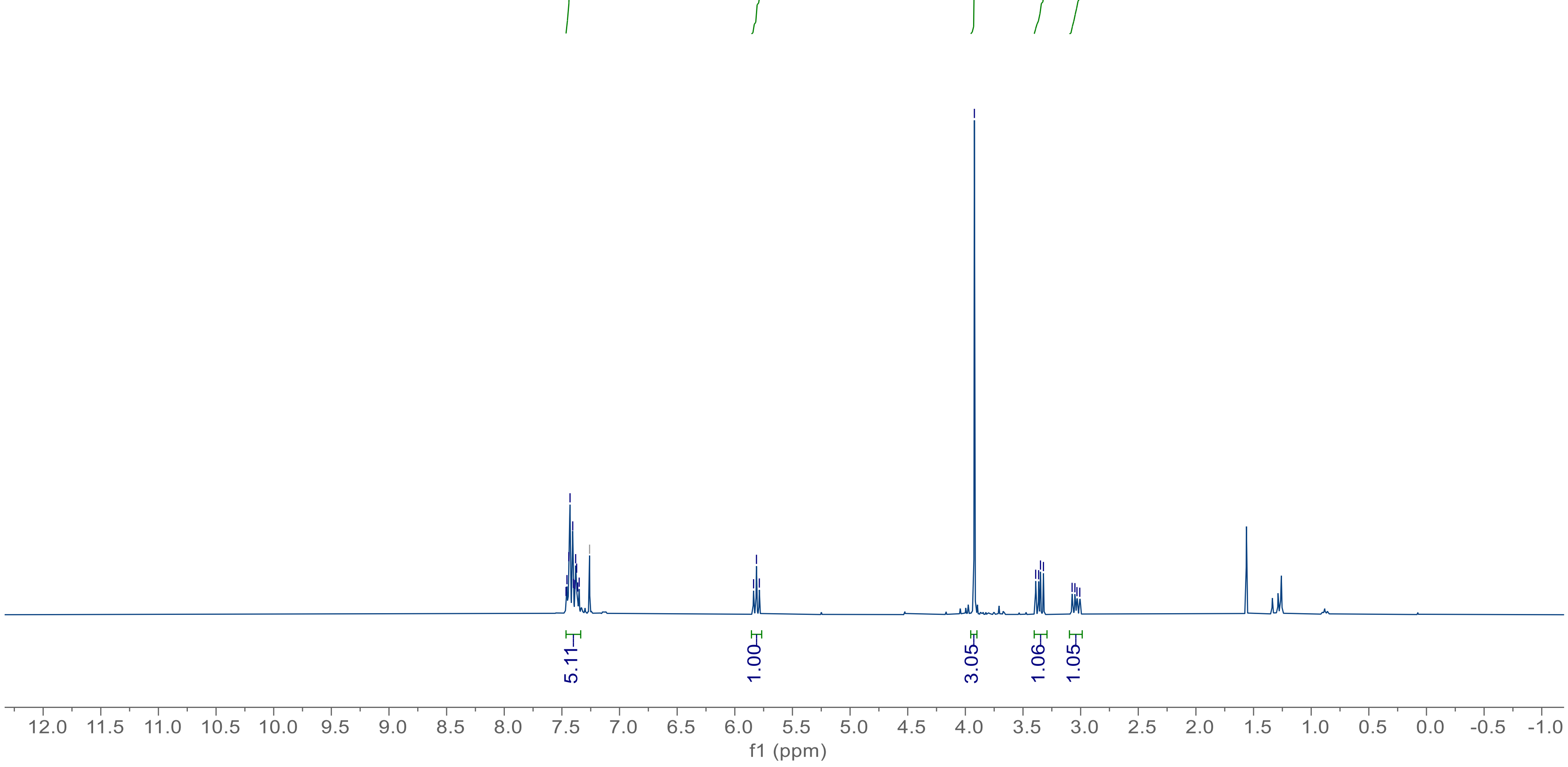

^{13}C NMR (75 MHz, $CDCl_3$) of mixture of **4b**

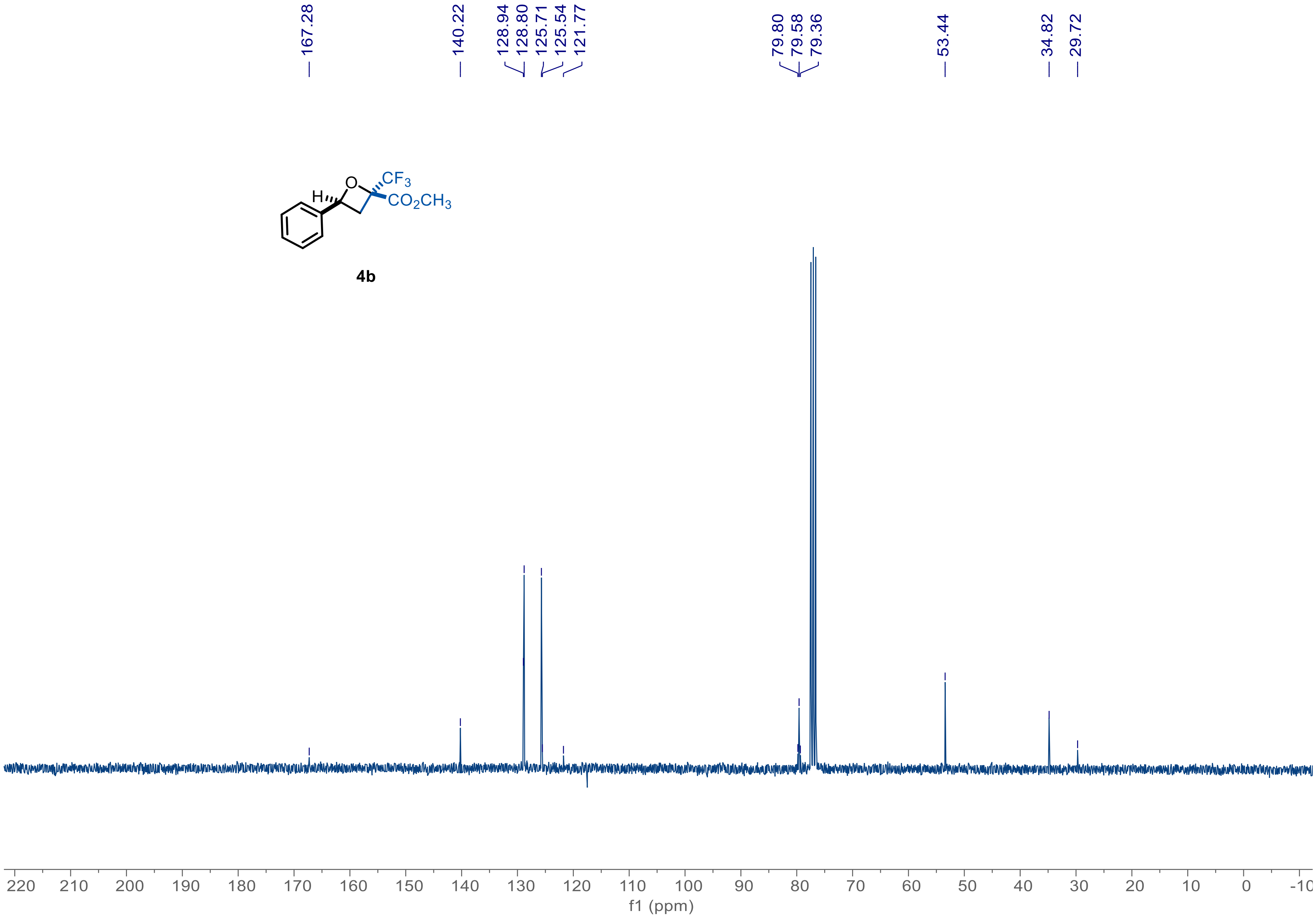

^{19}F NMR (282 MHz, $CDCl_3$) of **4b**

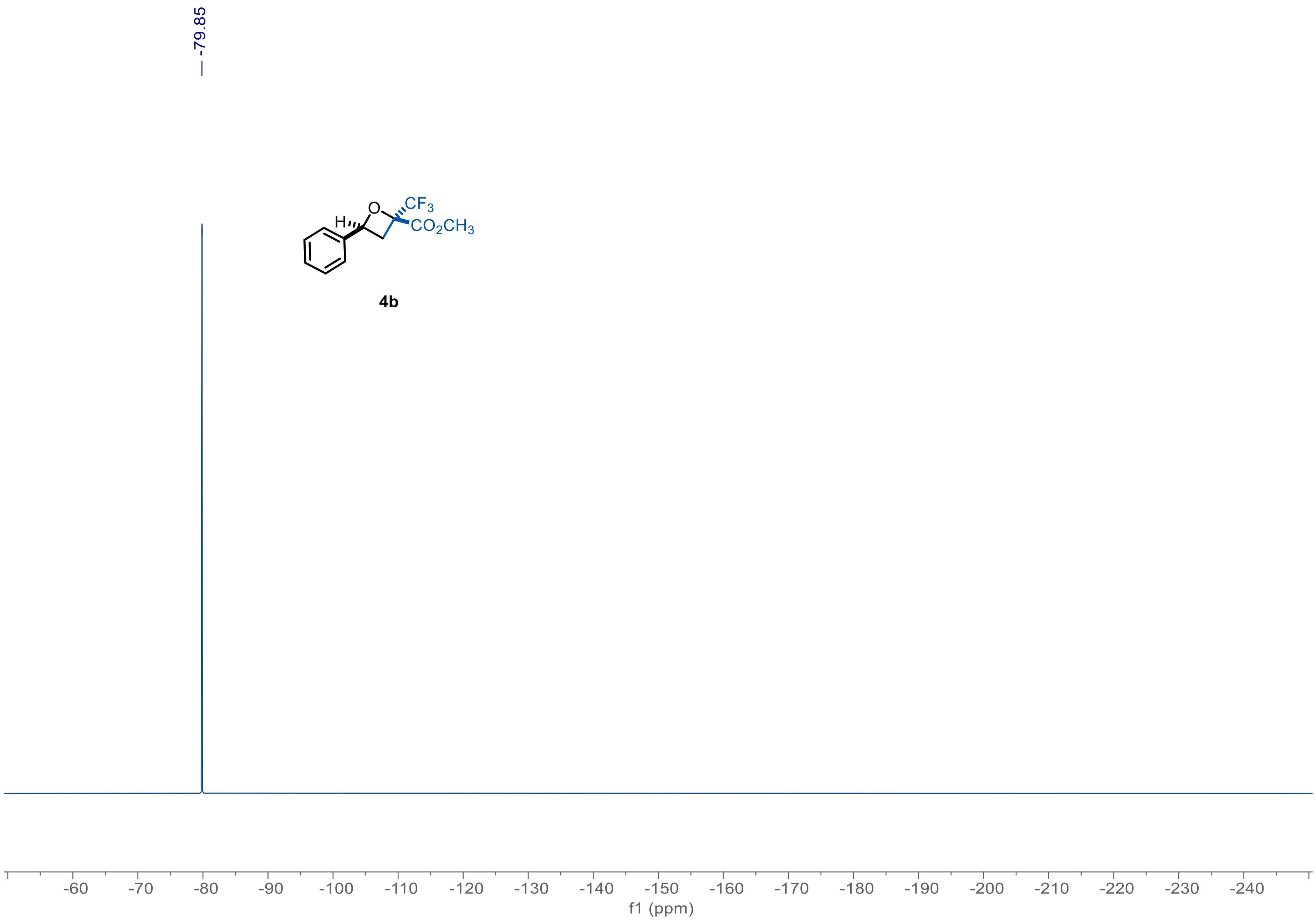

^{1}H NMR (300 MHz, $CDCl_3$) of **4a**, **4a'**, **4b'**

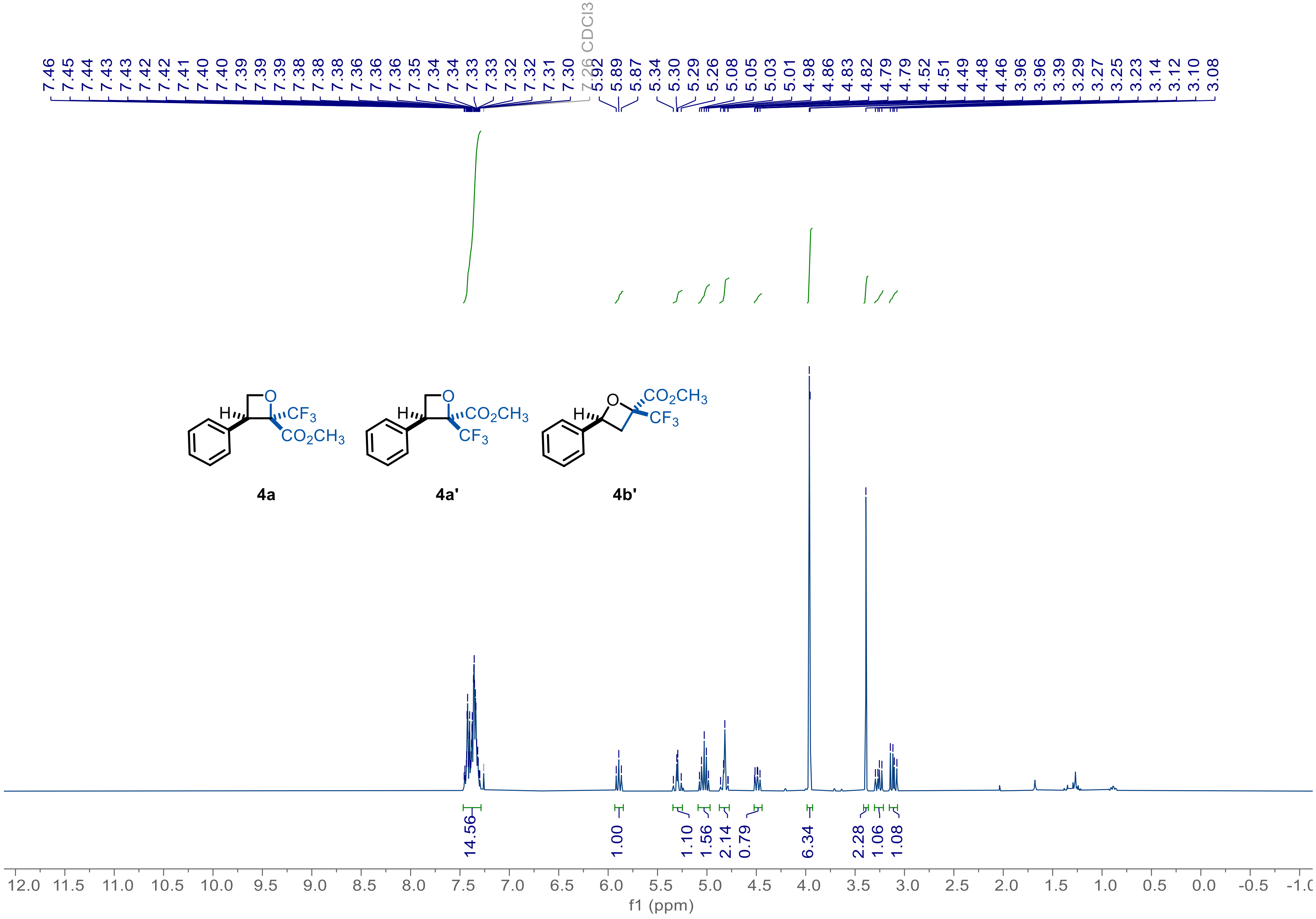

^{13}C NMR (75 MHz, $CDCl_3$) of **4a**, **4a’**, **4b’**

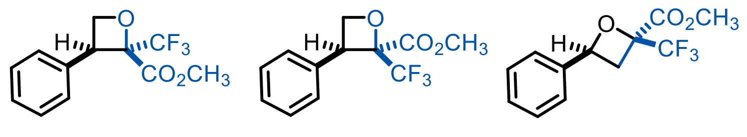

4a **4a'** **4b'**

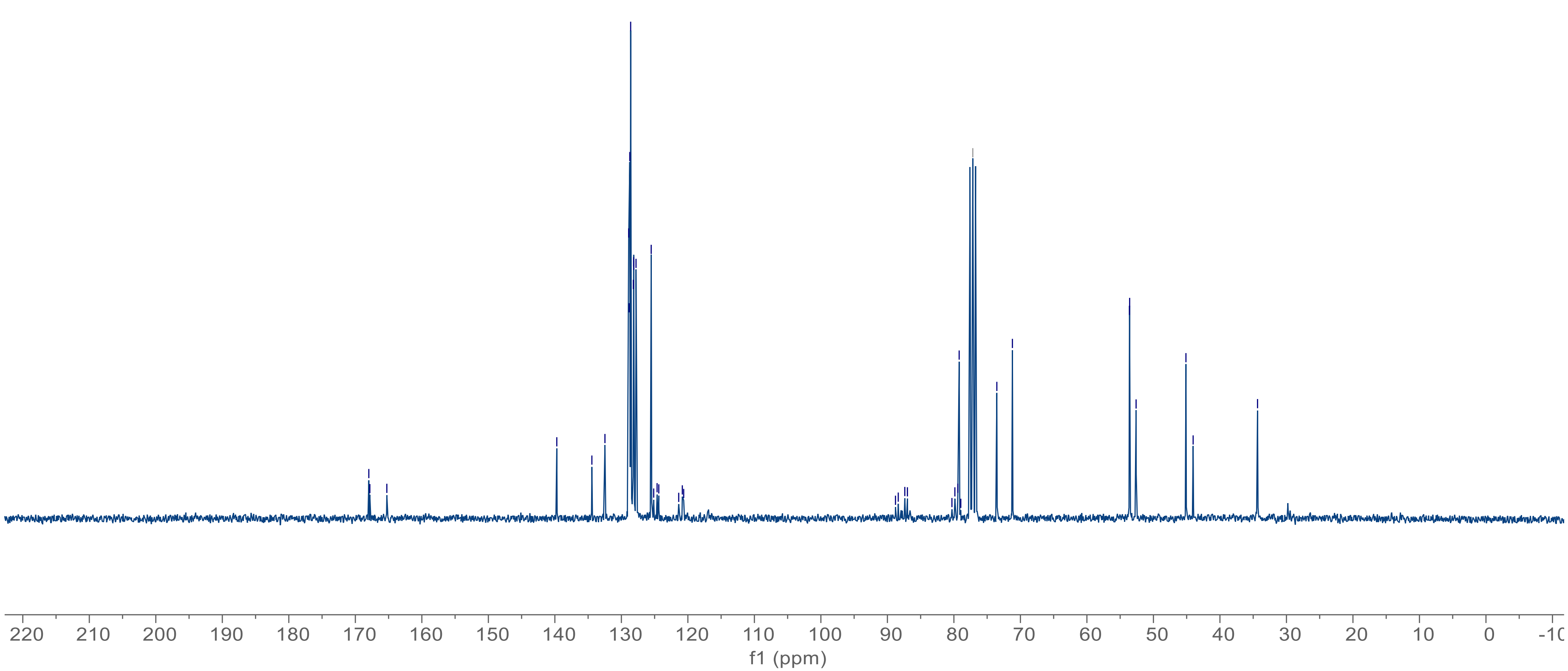

^{19}F NMR (282 MHz, $CDCl_3$) of **4a**, **4a'**, **4b'**

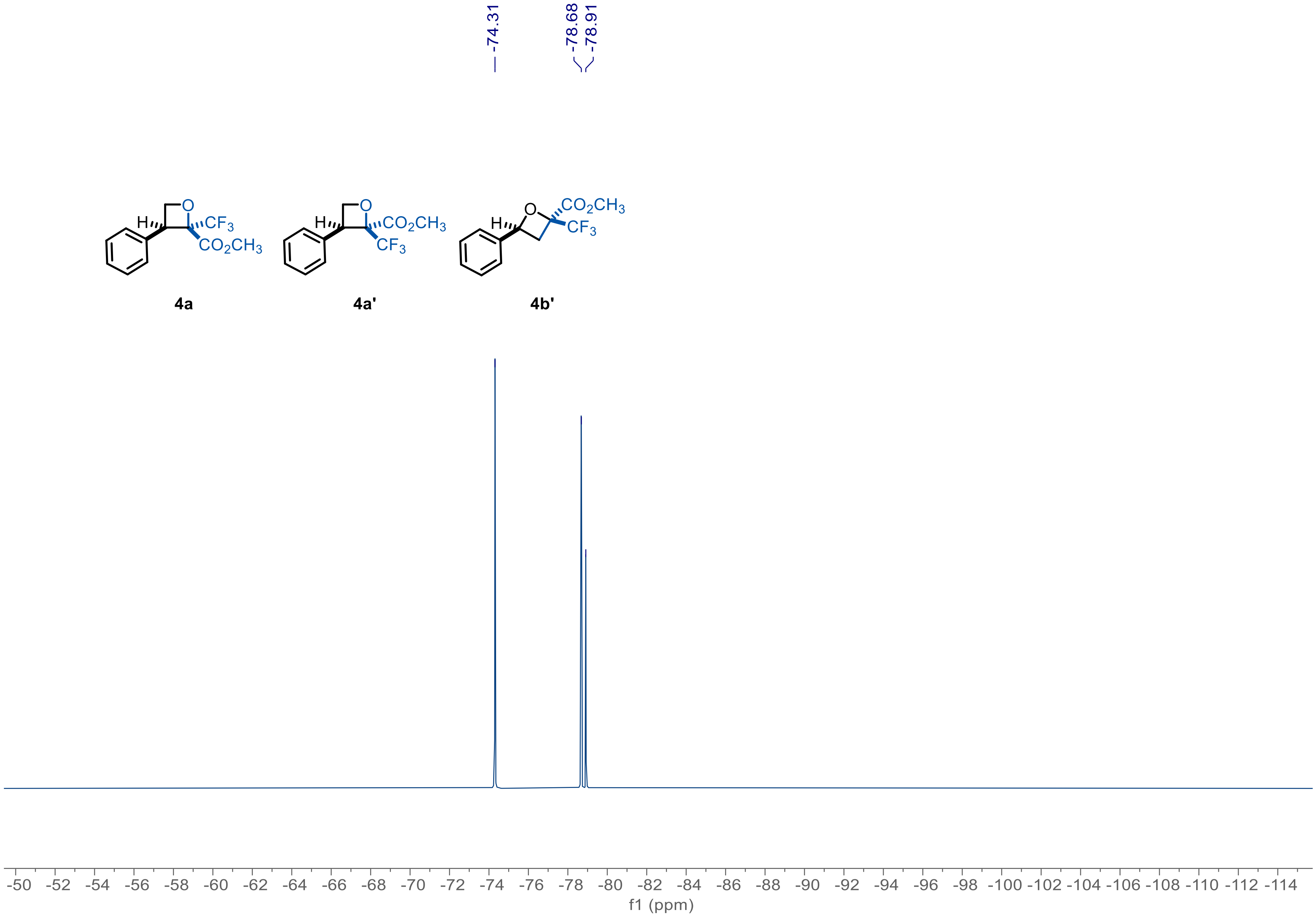

^{1}H NMR (300 MHz, $CDCl_3$) of **5a**

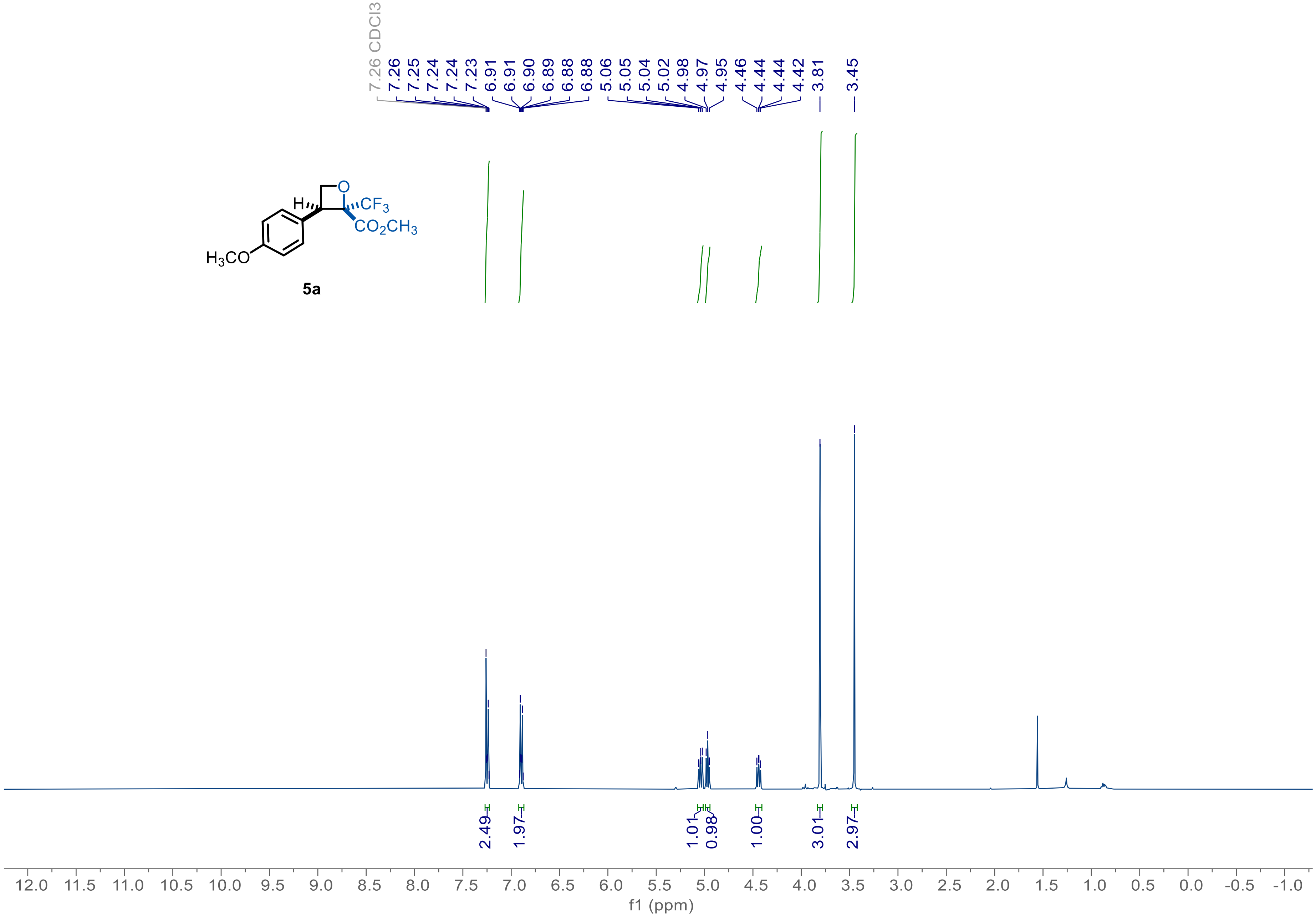

^{13}C NMR (101 MHz, $CDCl_3$) of **5a**

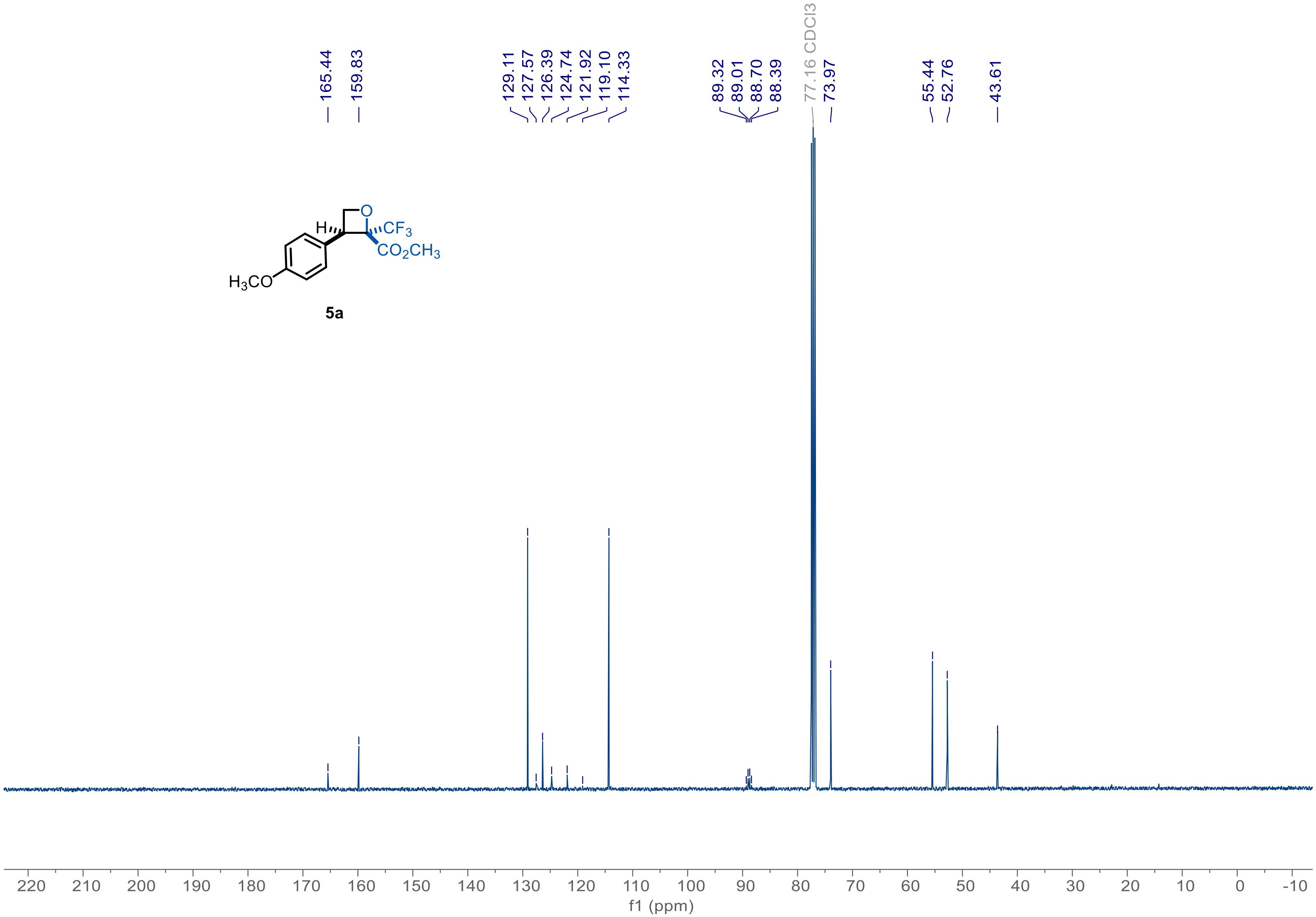

^{19}F NMR (282 MHz, $CDCl_3$) of **5a**

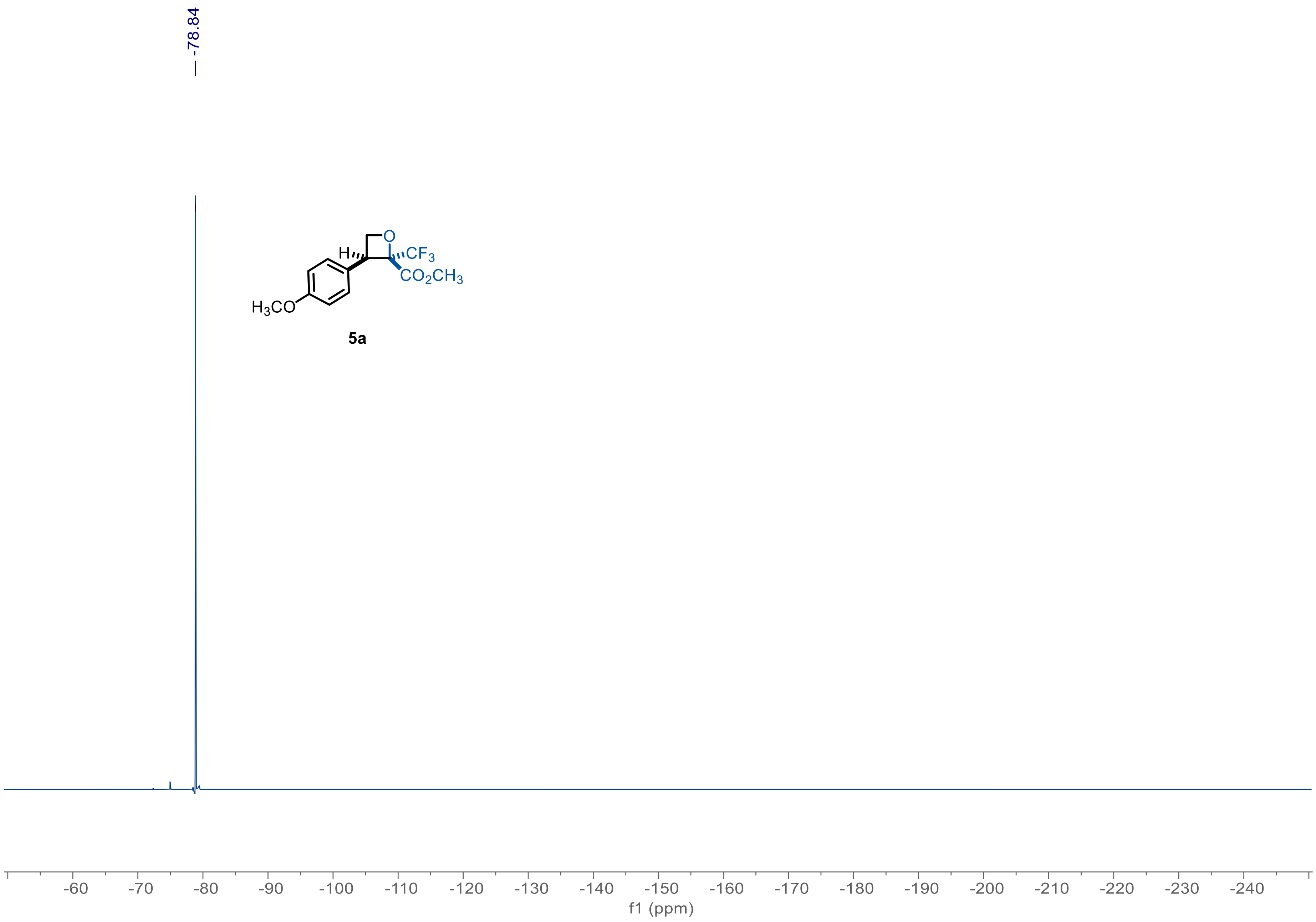

^{1}H NMR (300 MHz, $CDCl_3$) of **5a'**

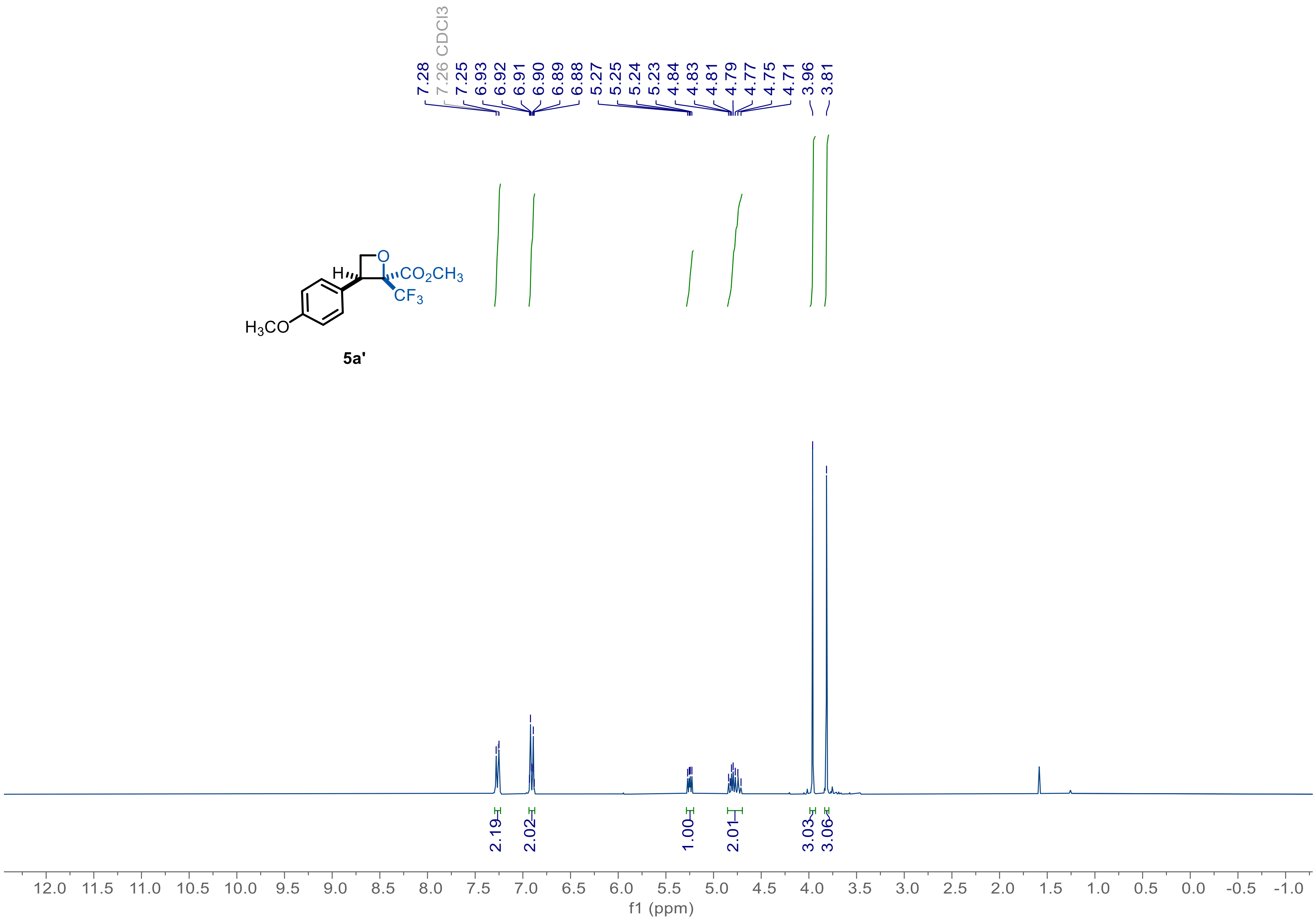

^{13}C NMR (75 MHz, $CDCl_3$) of **5a’**

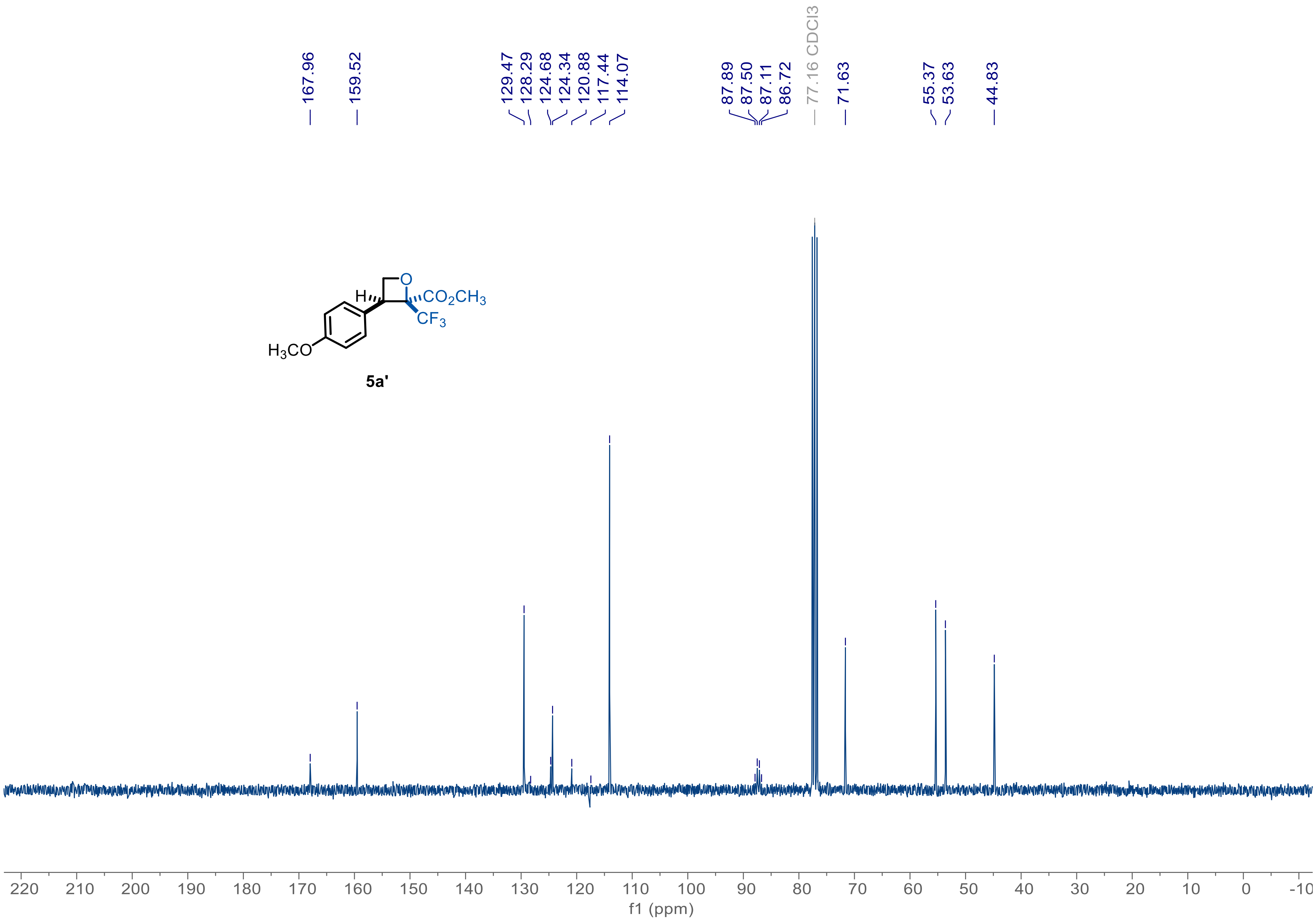

^{19}F NMR (282 MHz, $CDCl_3$) of **5a'**

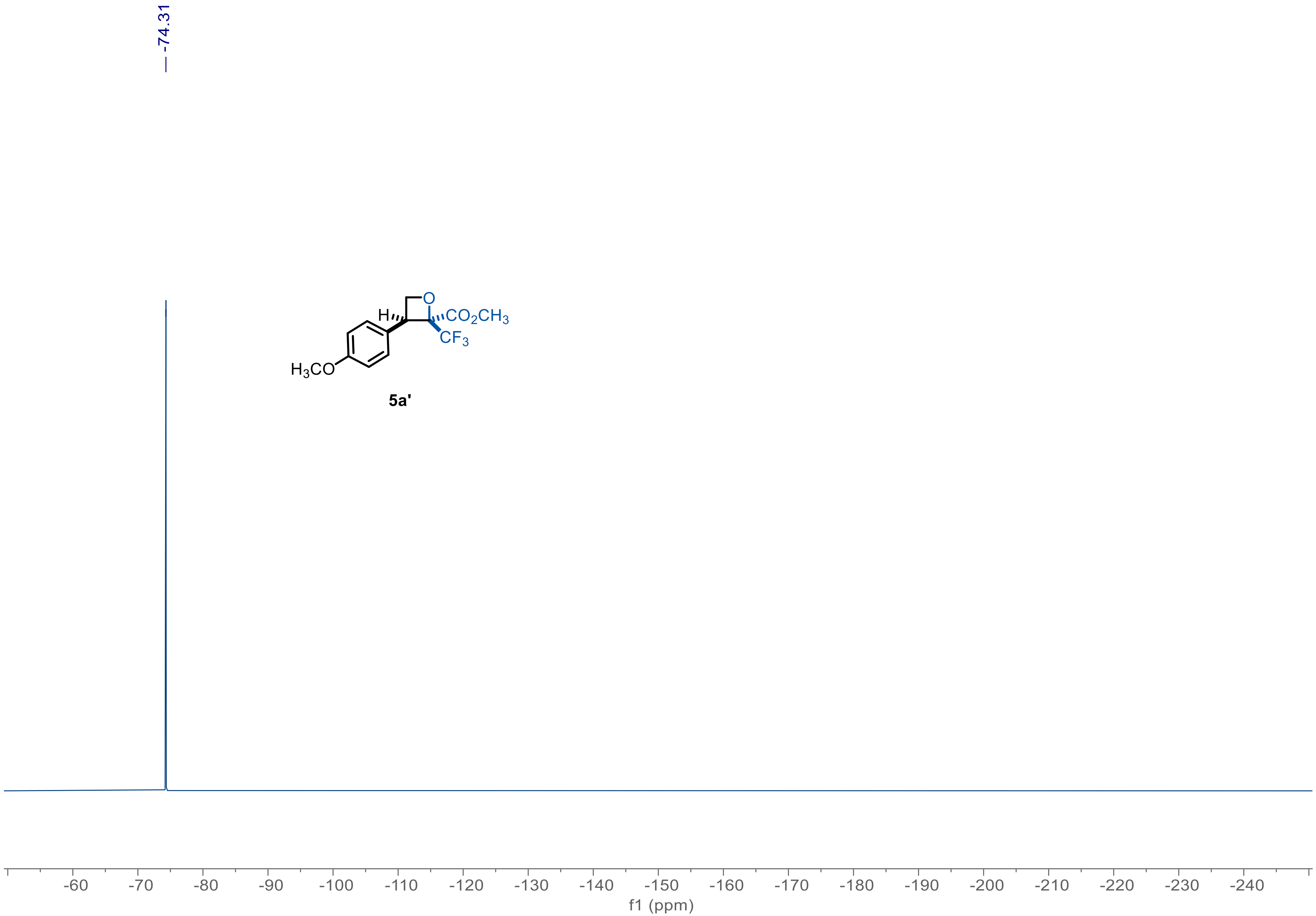

^{1}H NMR (300 MHz, $CDCl_3$) of **6a**

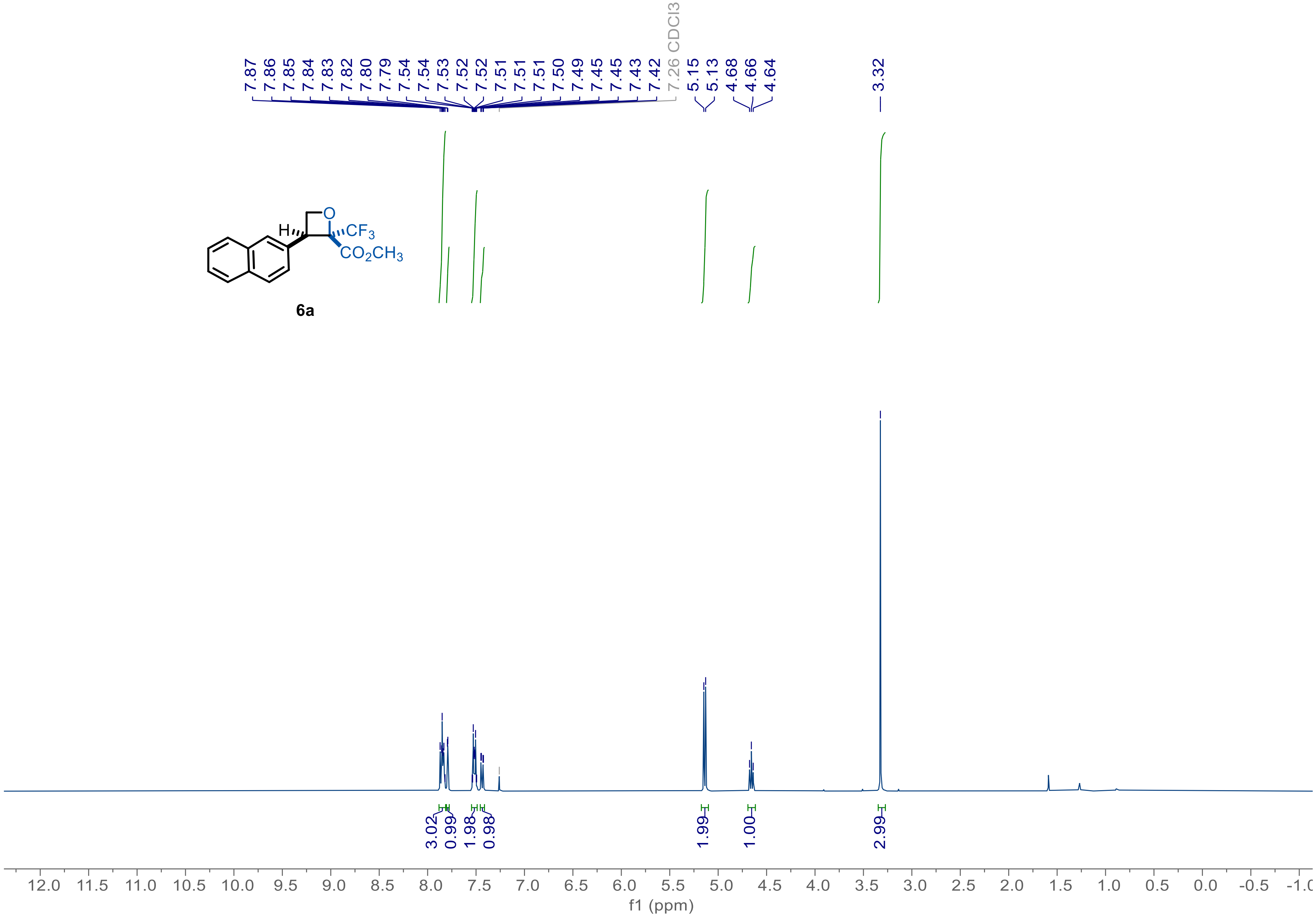

^{13}C NMR (101 MHz, $CDCl_3$) of **6a**

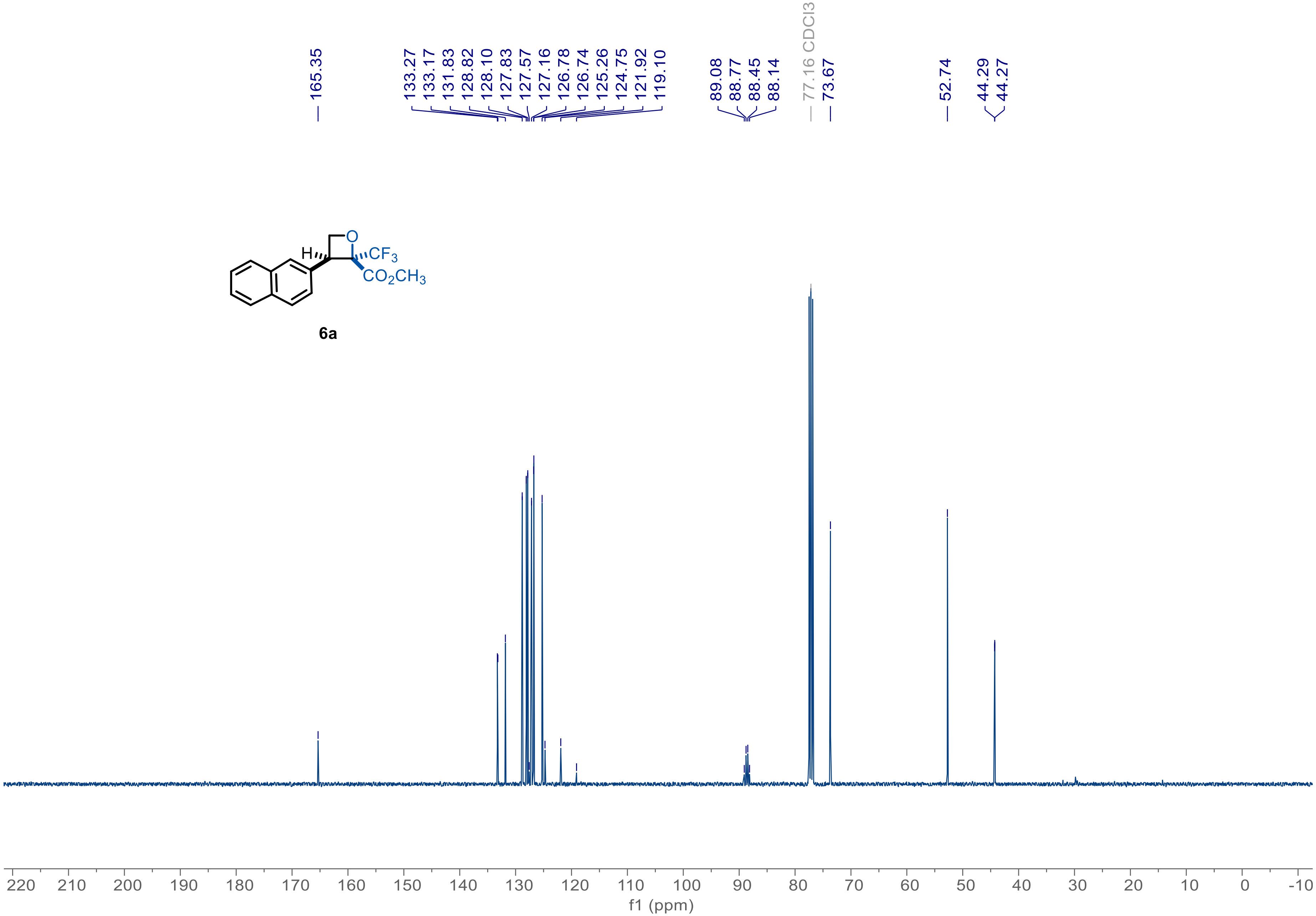

^{19}F NMR (282 MHz, $CDCl_3$) of **6a**

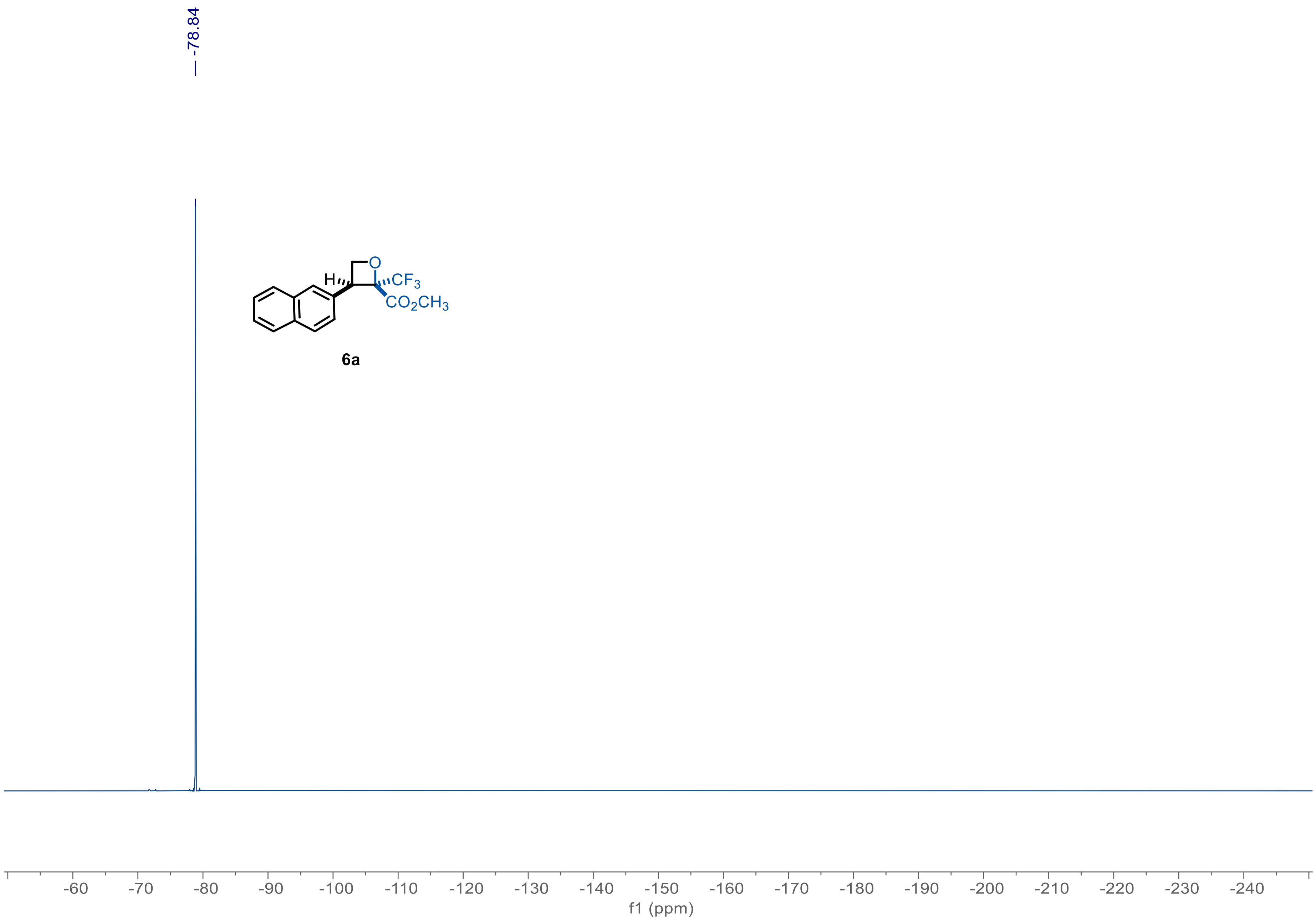

^{1}H NMR (300 MHz, $CDCl_3$) of **6a’**

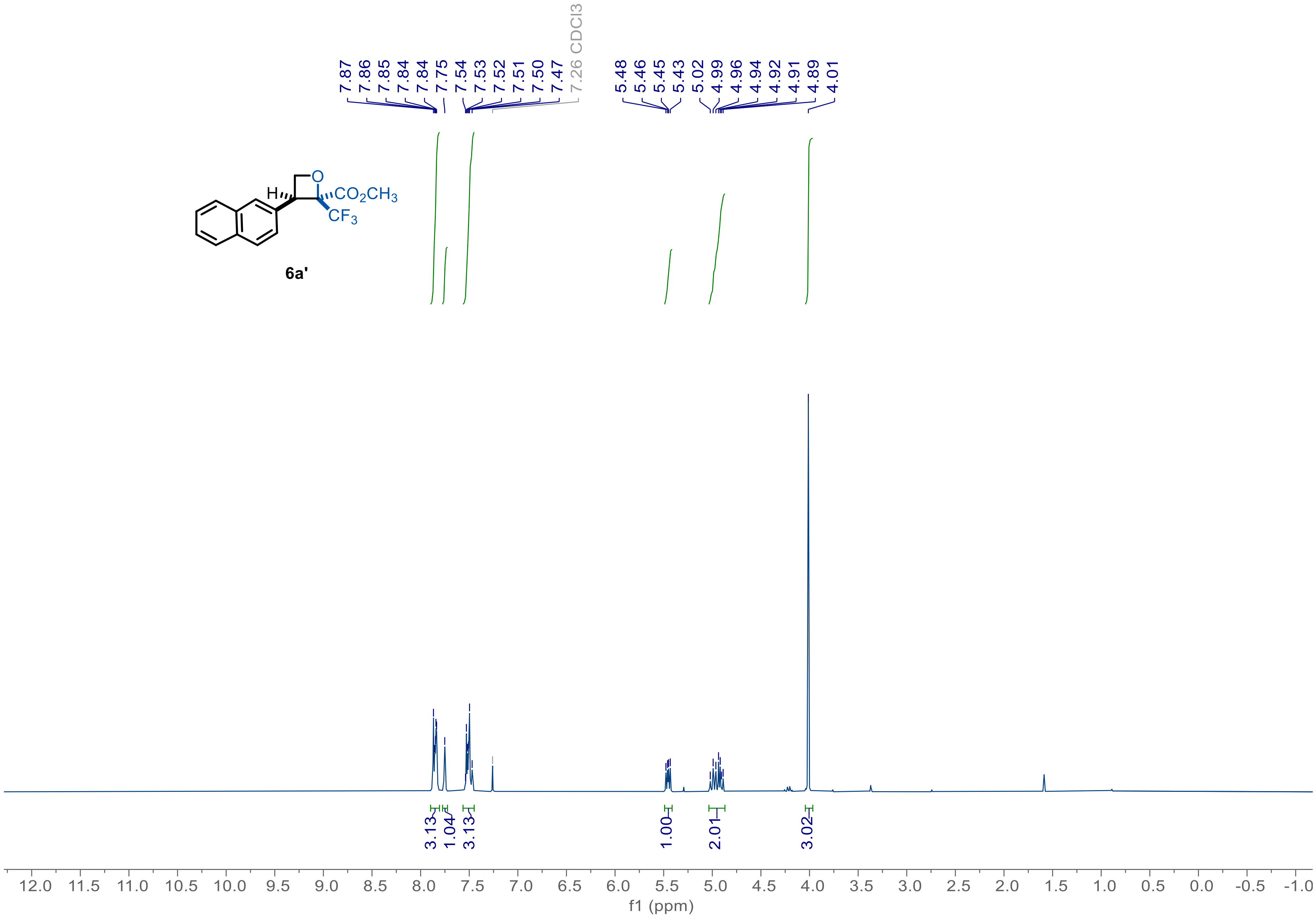

^{13}C NMR (101 MHz, $CDCl_3$) of **6a'**

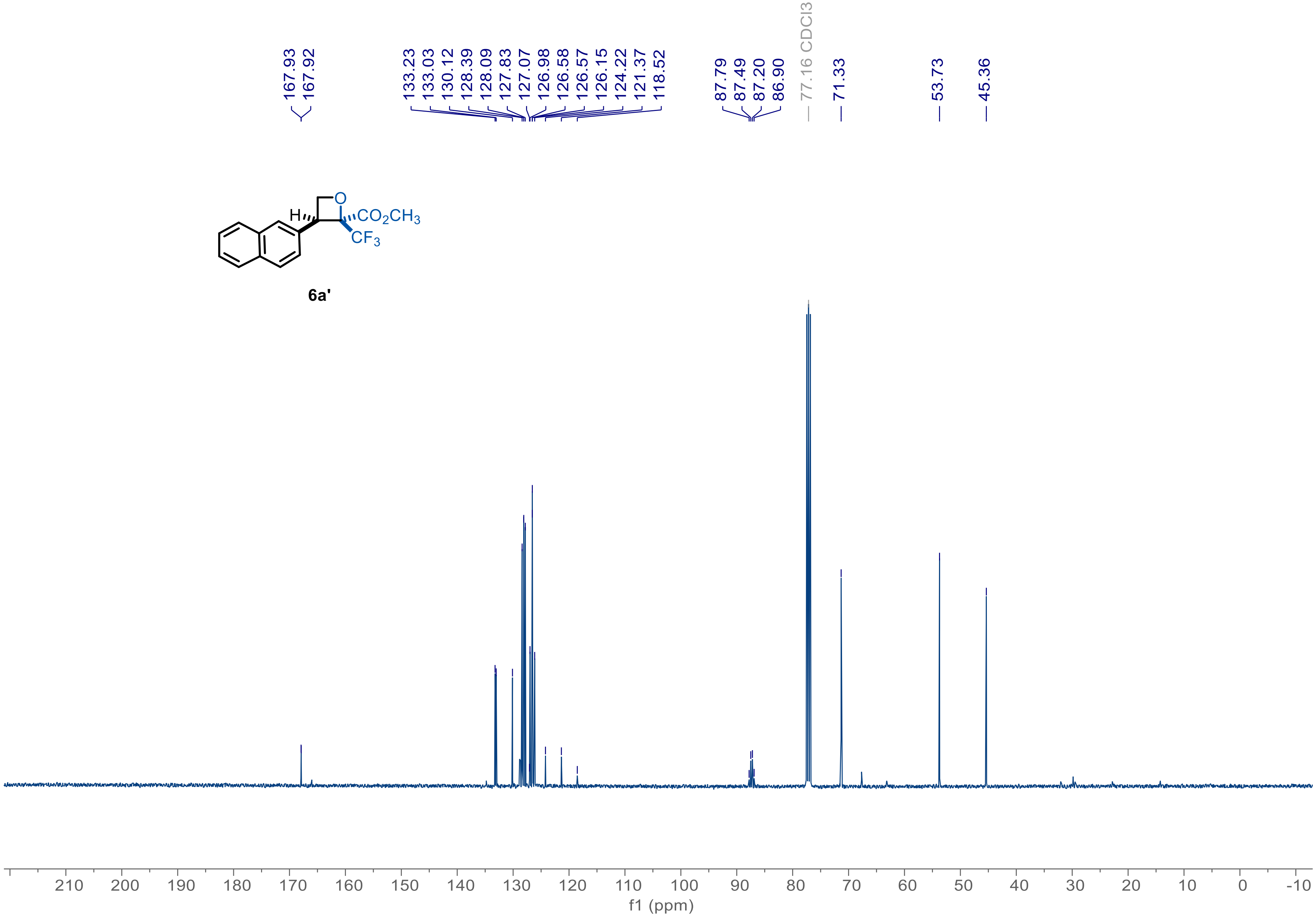

^{19}F NMR (282 MHz, $CDCl_3$) of **6a’**

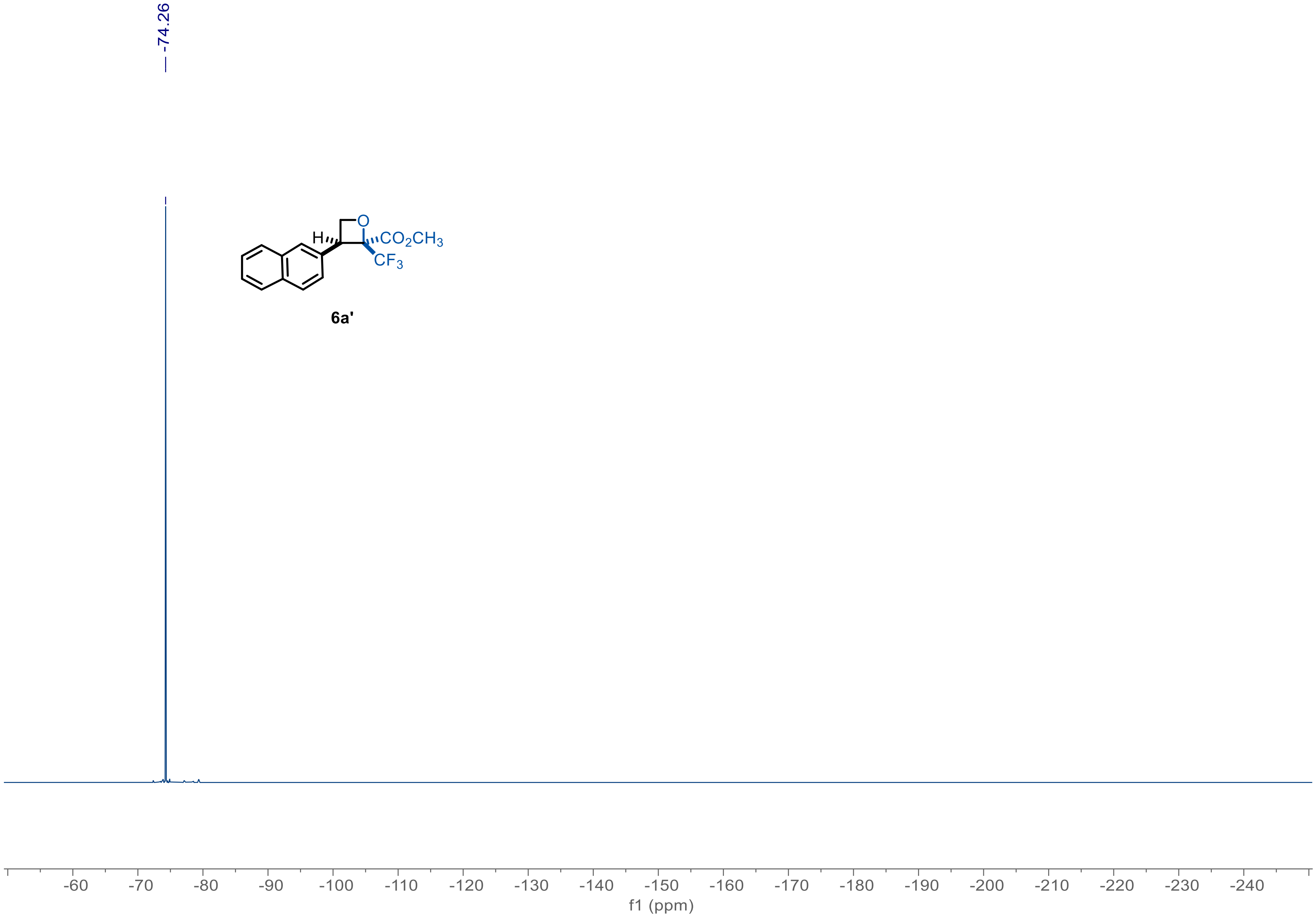

^{1}H NMR (300 MHz, $CDCl_3$) of **6b**

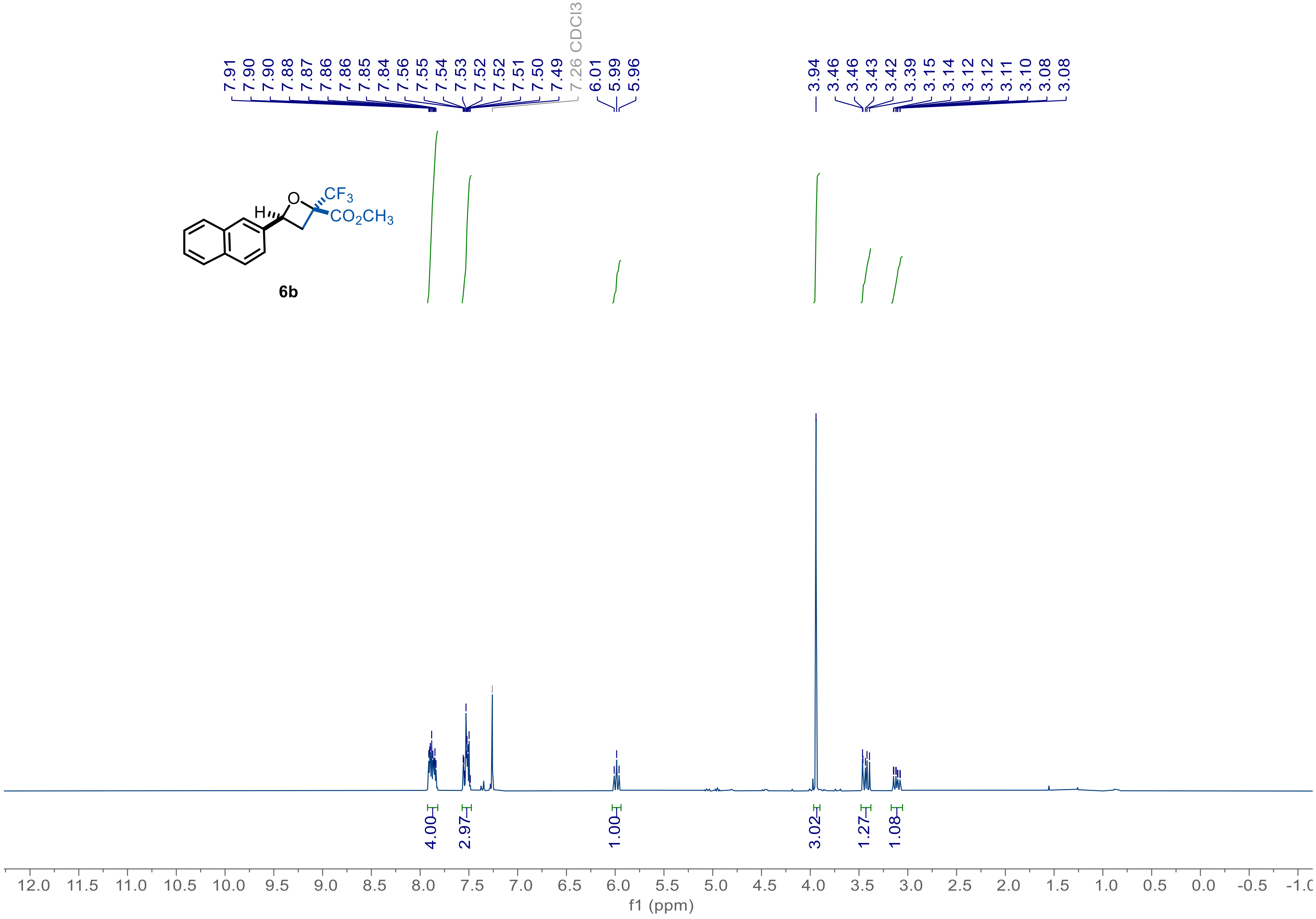

^{13}C NMR (101 MHz, $CDCl_3$) of **6b**

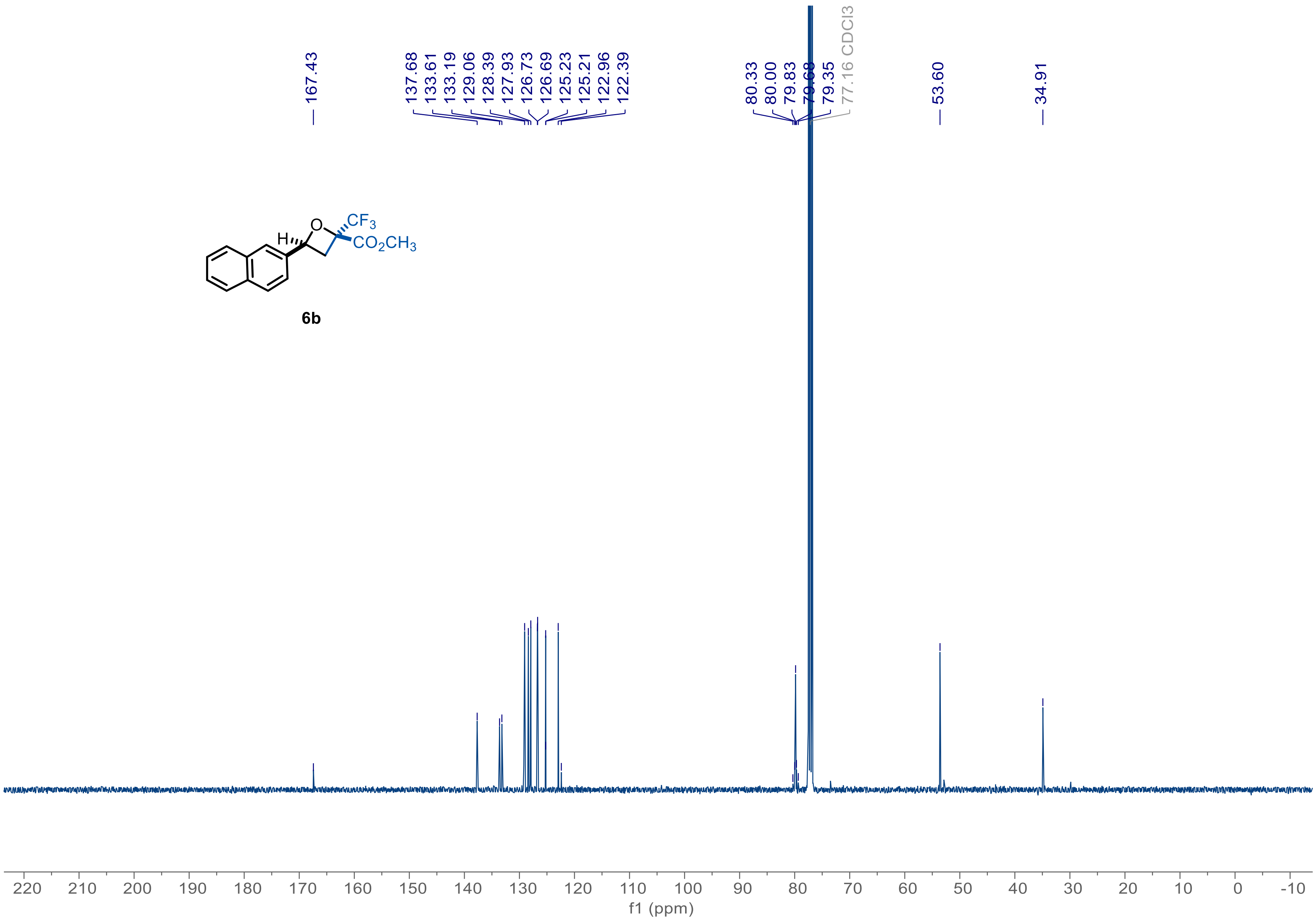

^{19}F NMR (282 MHz, $CDCl_3$) of **6b**

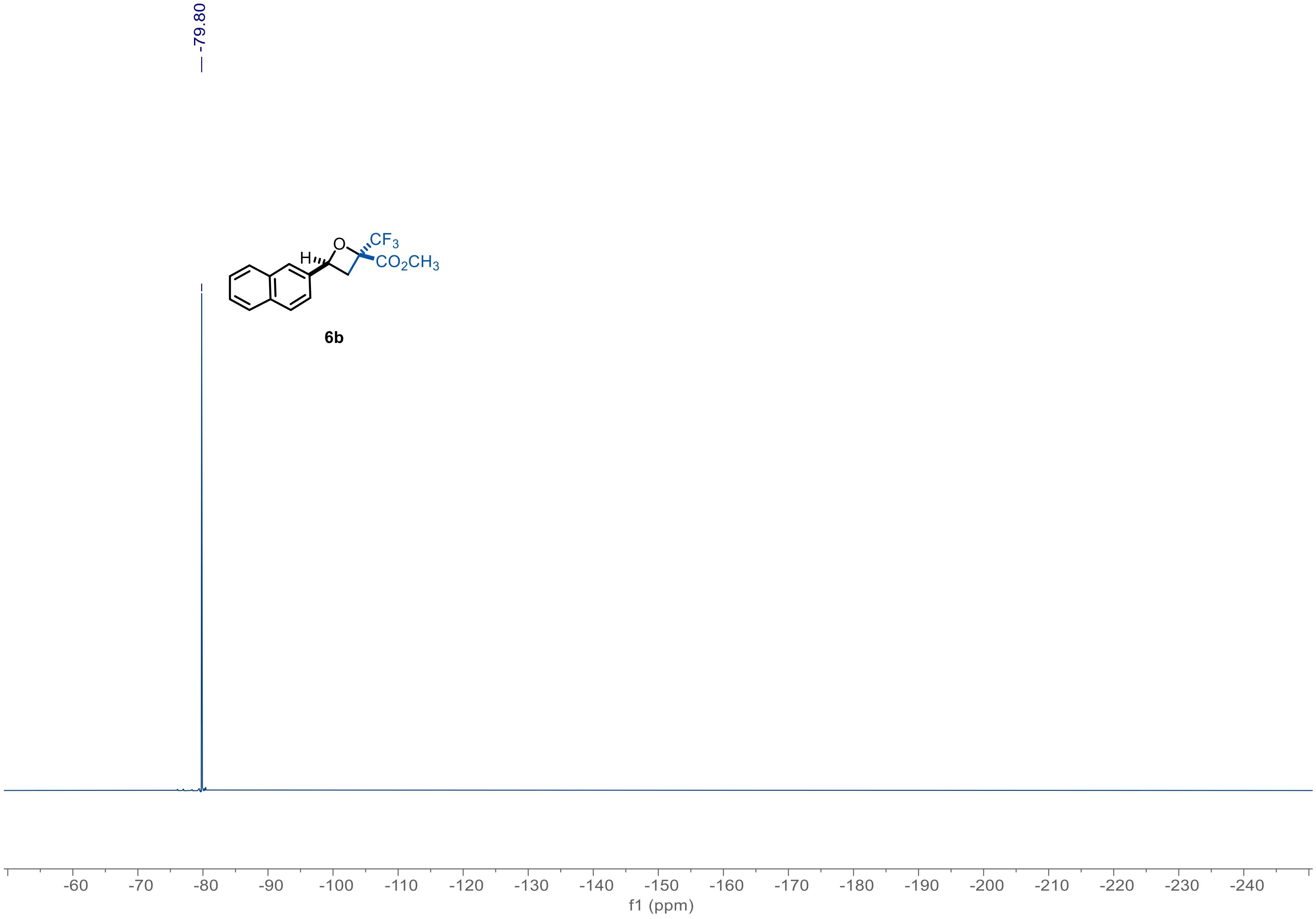

^{1}H NMR (300 MHz, $CDCl_3$) of **6b'**

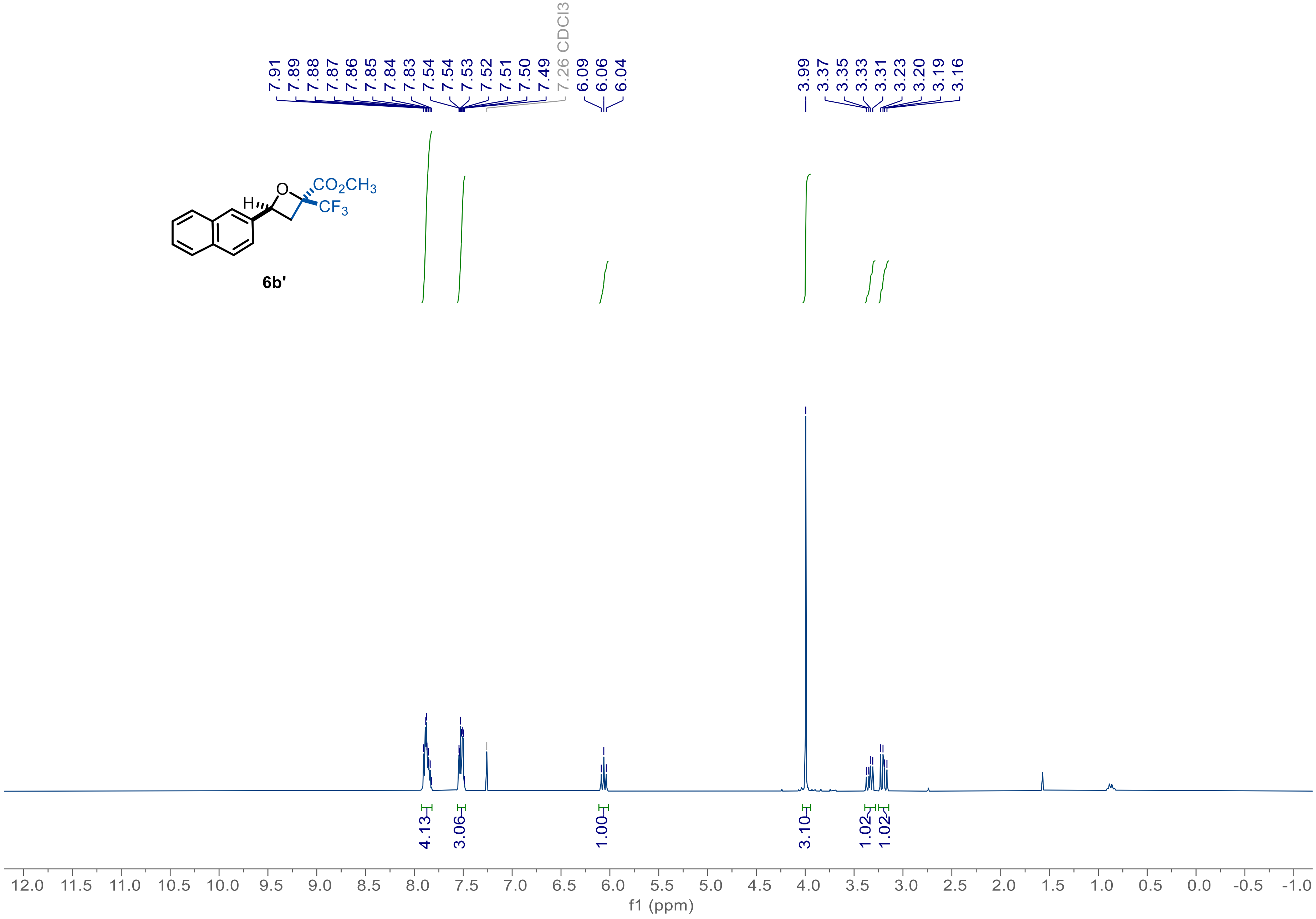

^{13}C NMR (75 MHz, $CDCl_3$) of **6b’**

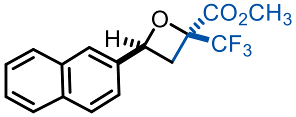

6b'

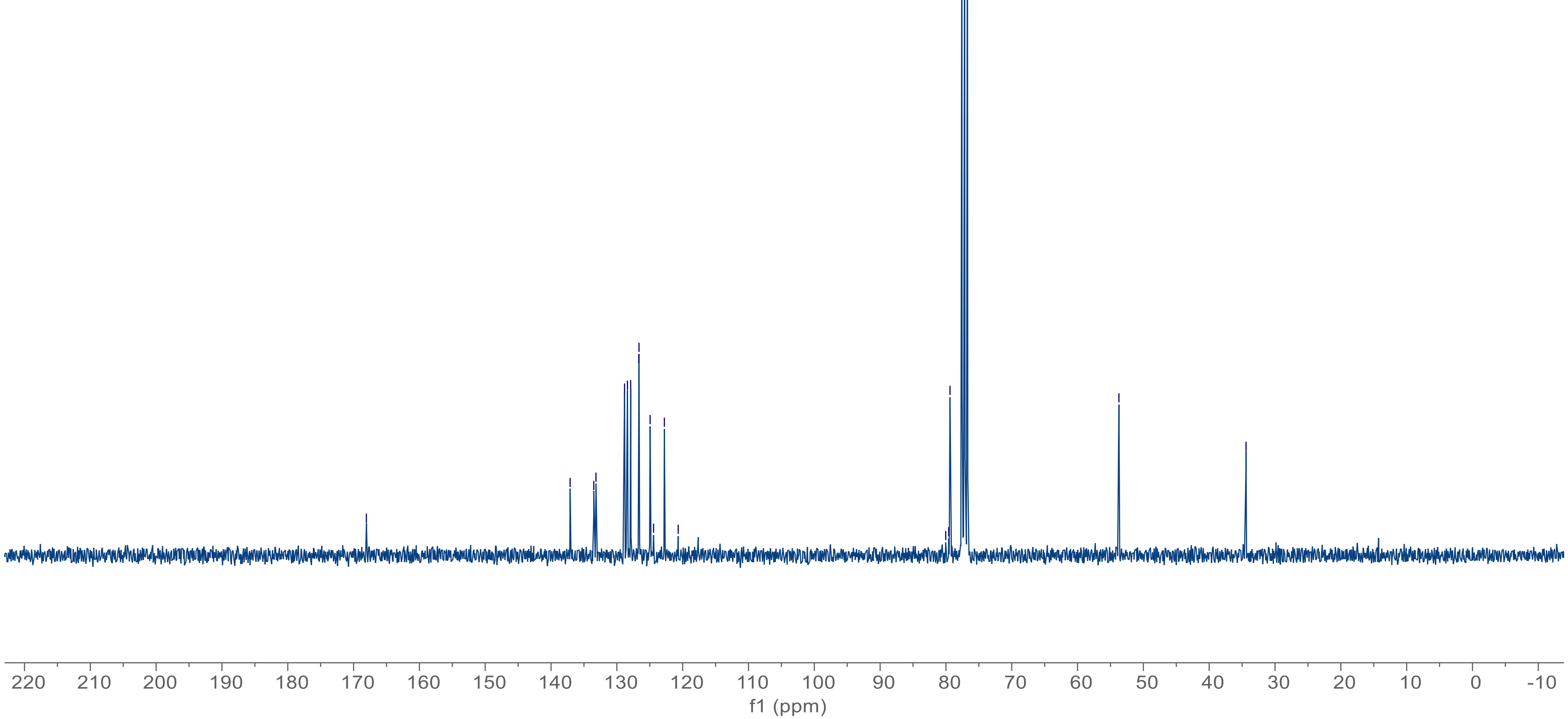

^{19}F NMR (282 MHz, $CDCl_3$) of **6b’**

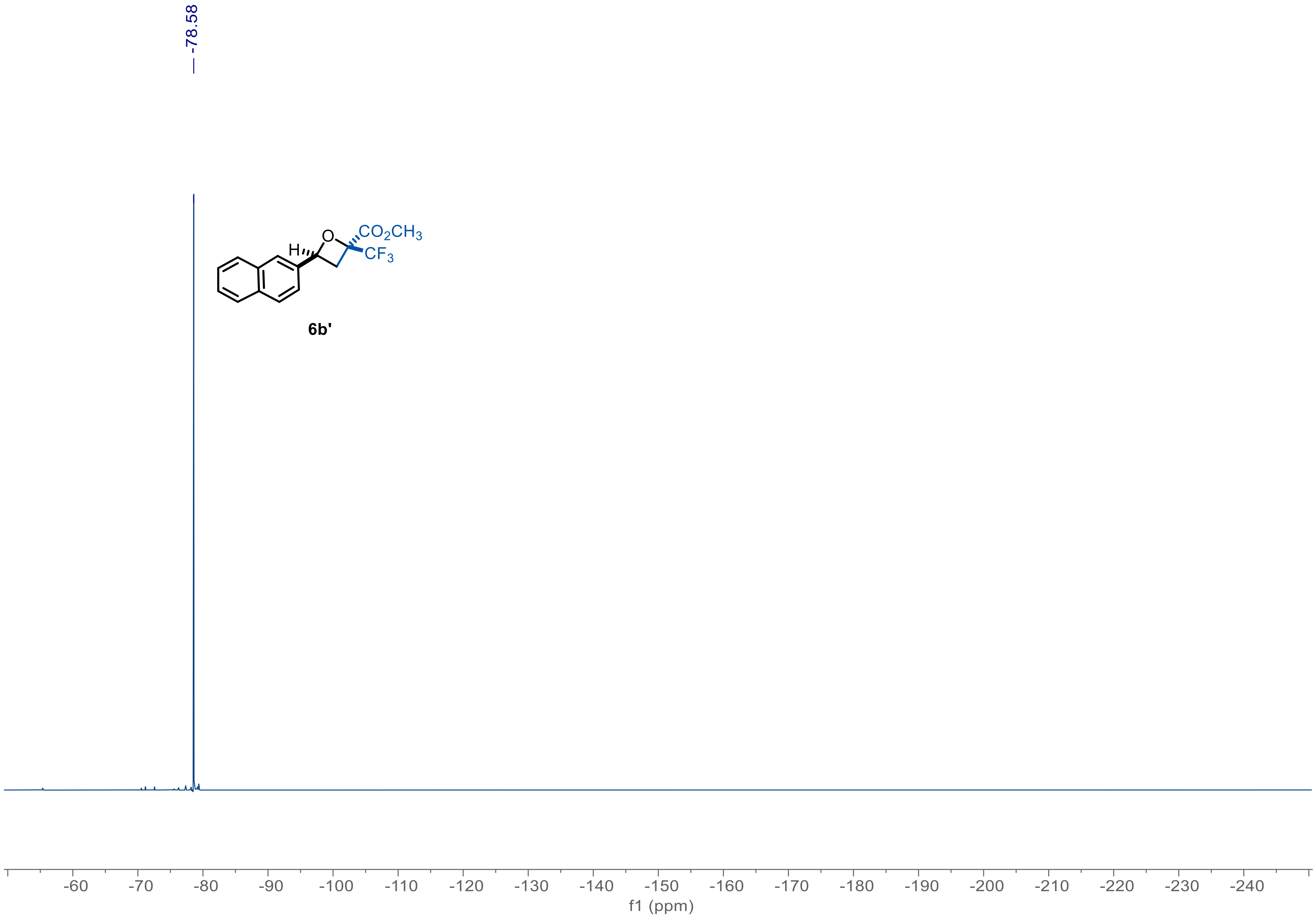

^{1}H NMR (300 MHz, $CDCl_3$) of **7a**

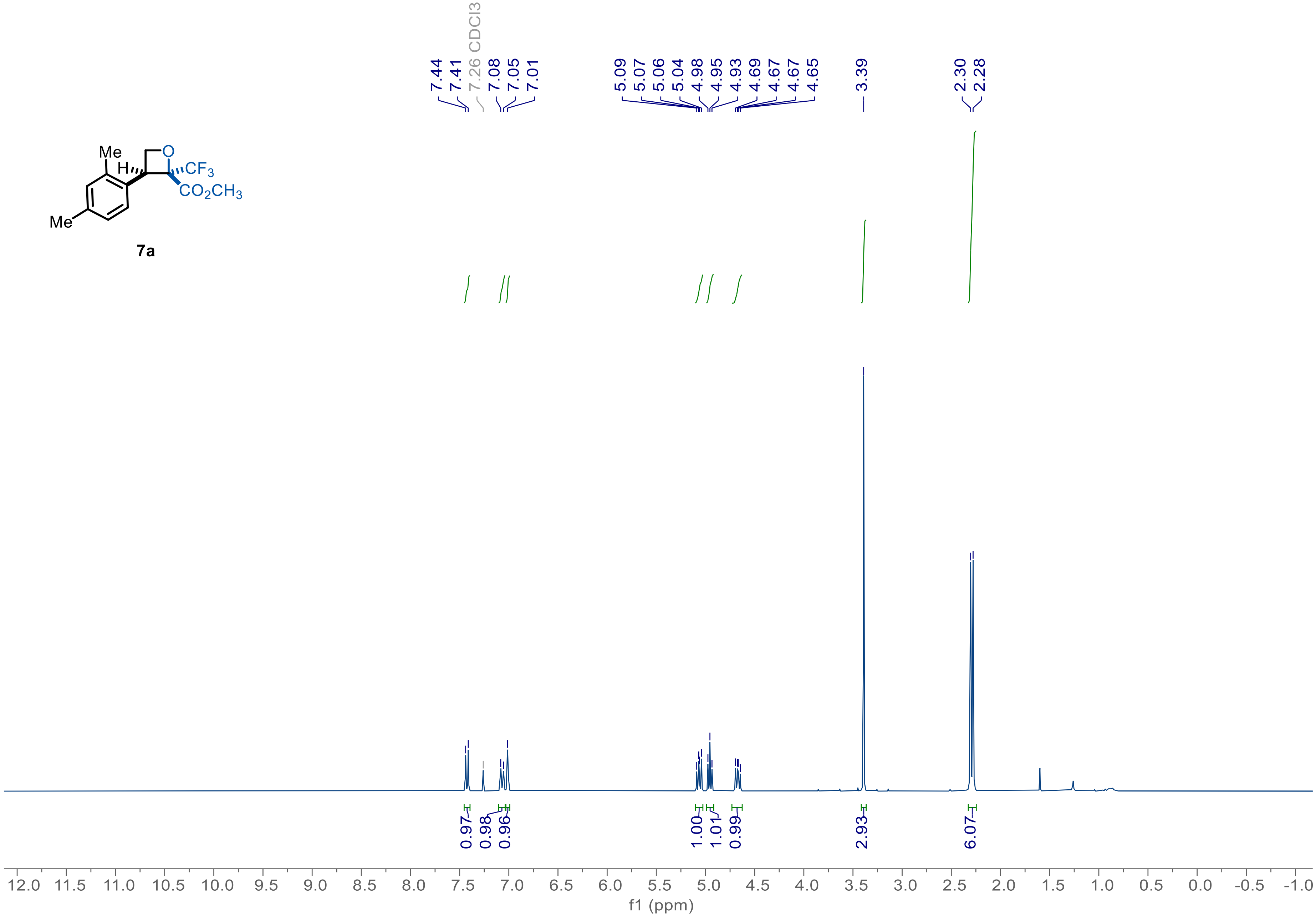

^{13}C NMR (75 MHz, $CDCl_3$) of **7a**

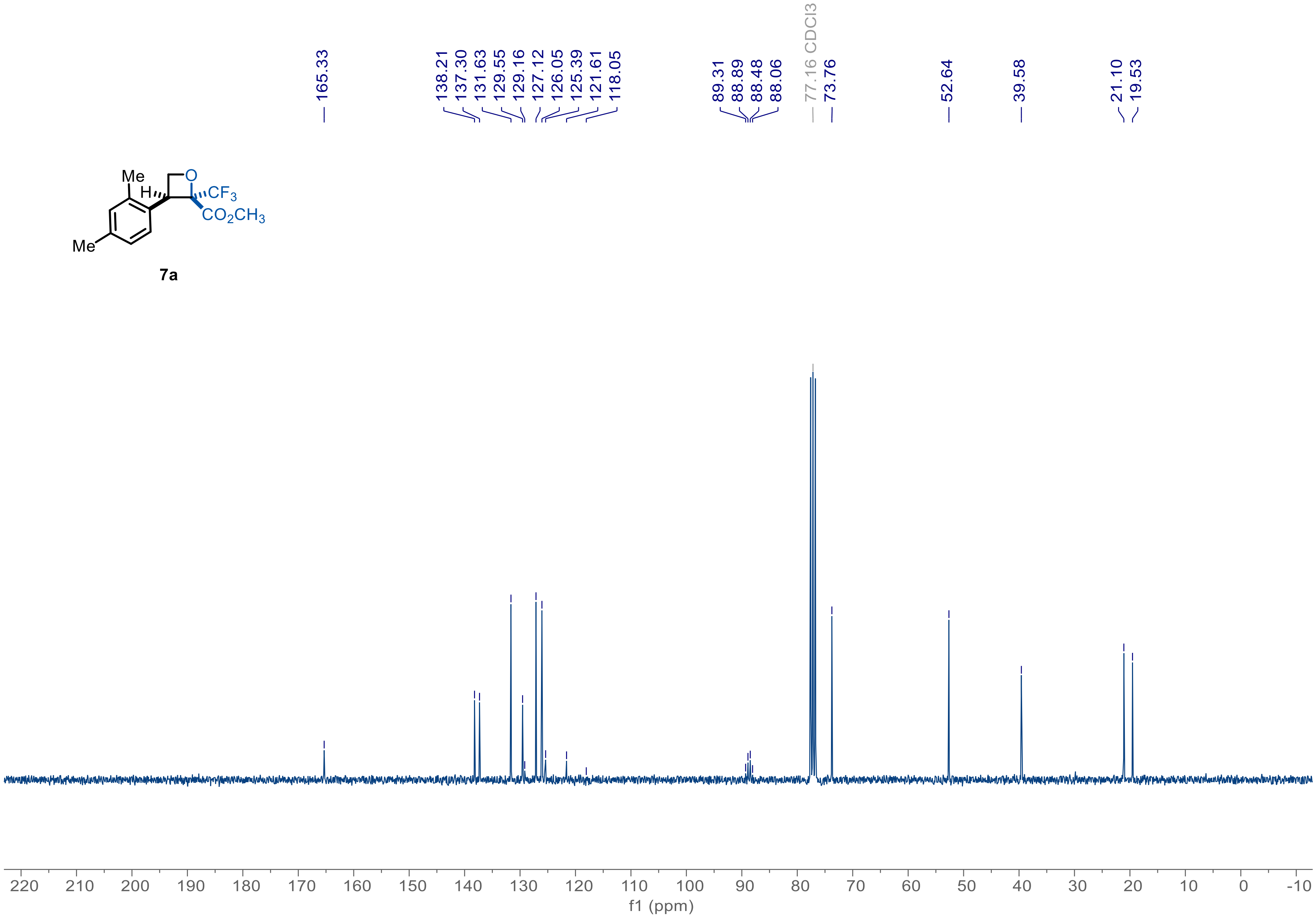

7a

^{19}F NMR (282 MHz, $CDCl_3$) of **7a**

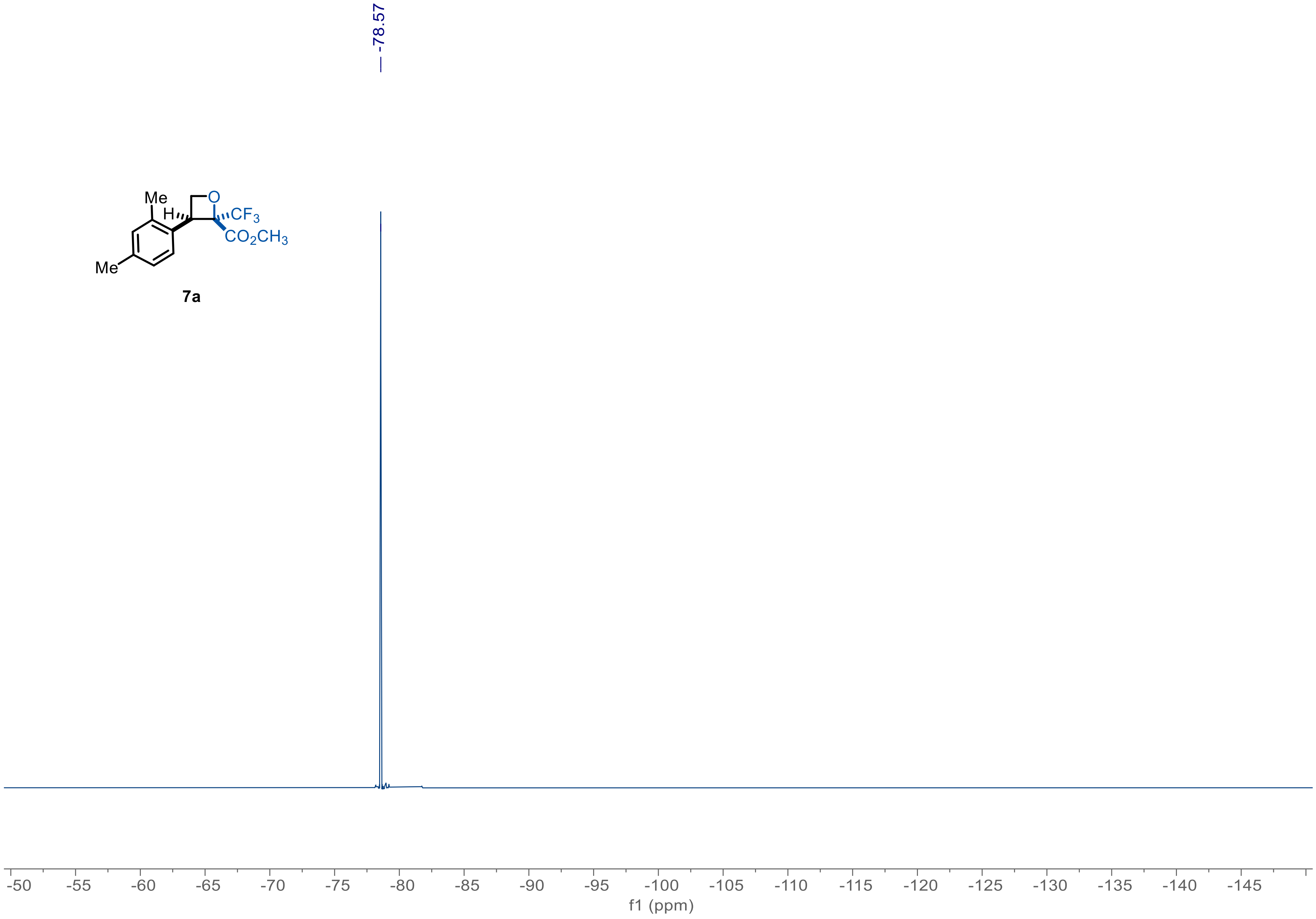

^1H NMR (300 MHz, $CDCl_3$) of **7a'**

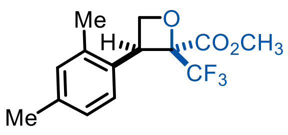

7a'

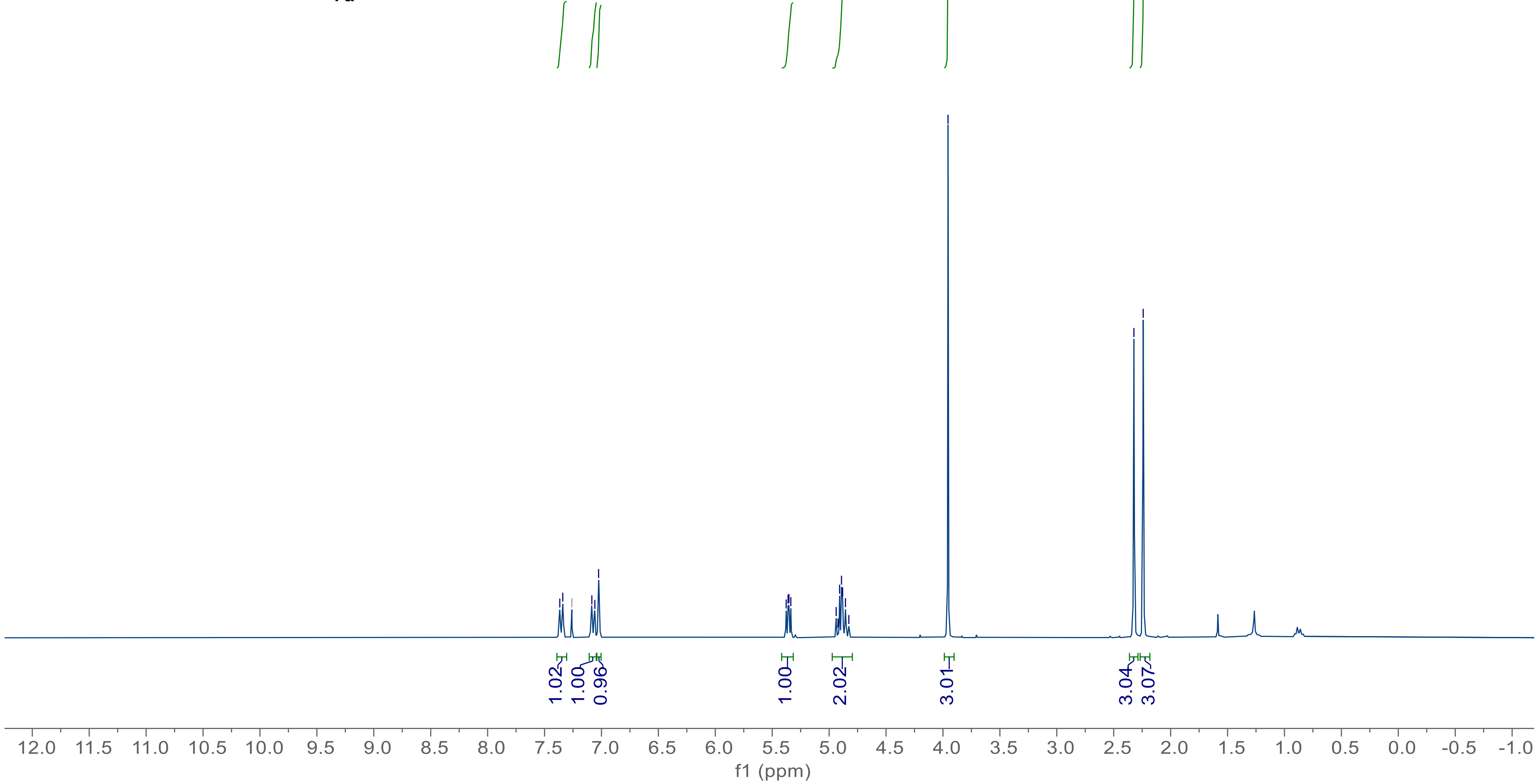

^{13}C NMR (75 MHz, $CDCl_3$) of **7a'**

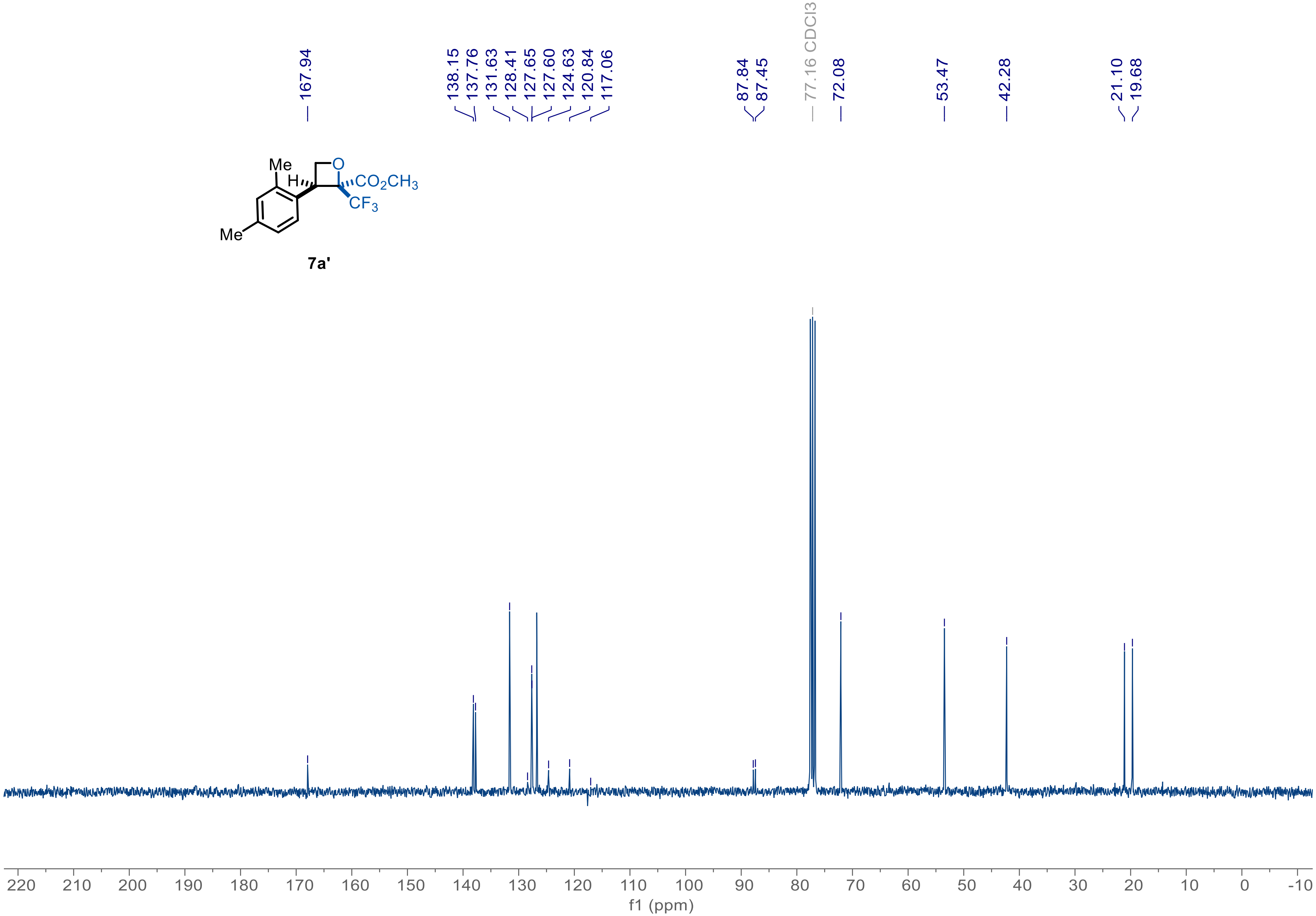

^{19}F NMR (282 MHz, CDCl3) of **7a’**

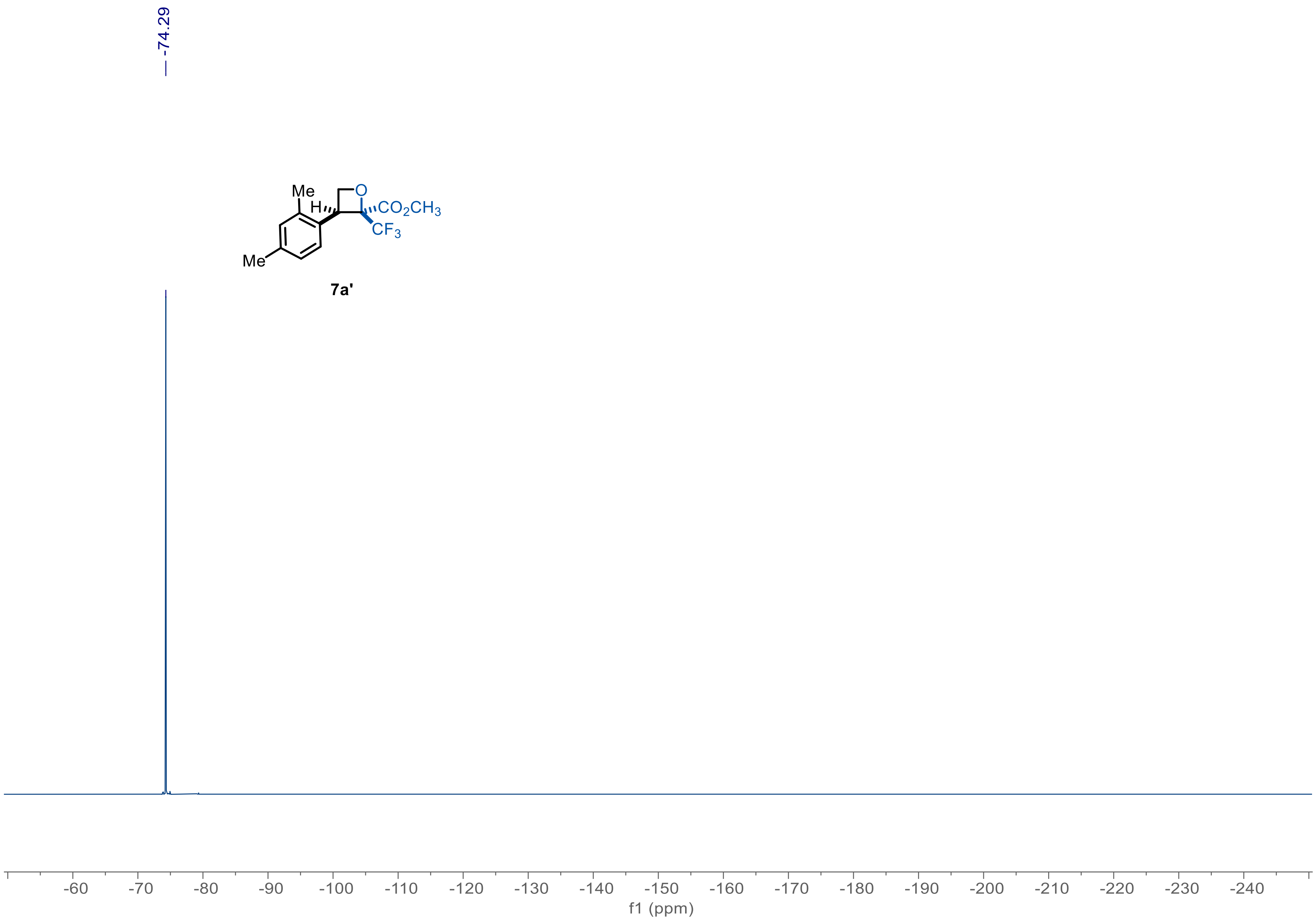

^{1}H NMR (300 MHz, $CDCl_3$) of **7b'**

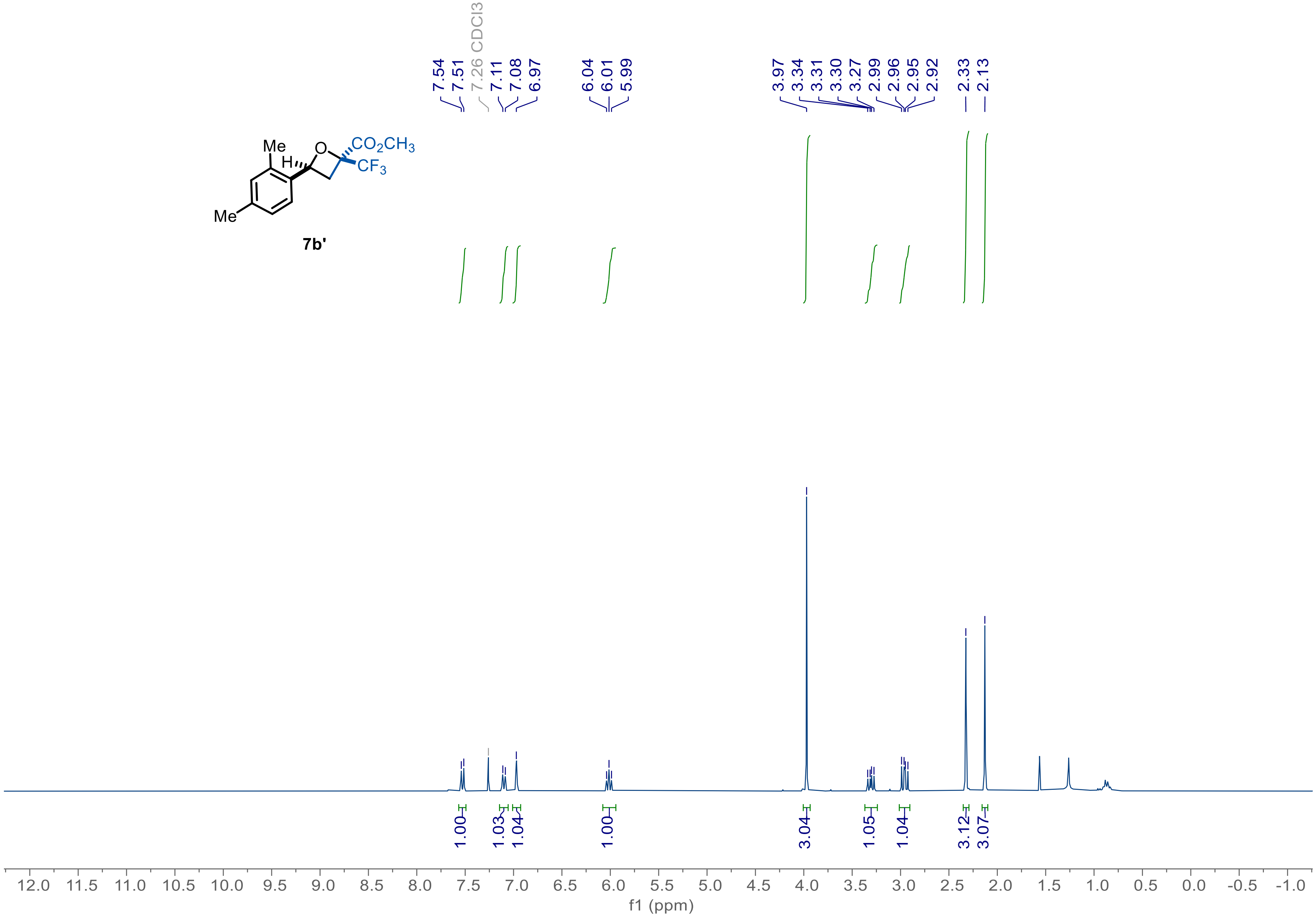

^{13}C NMR (101 MHz, $CDCl_3$) of **7b'**

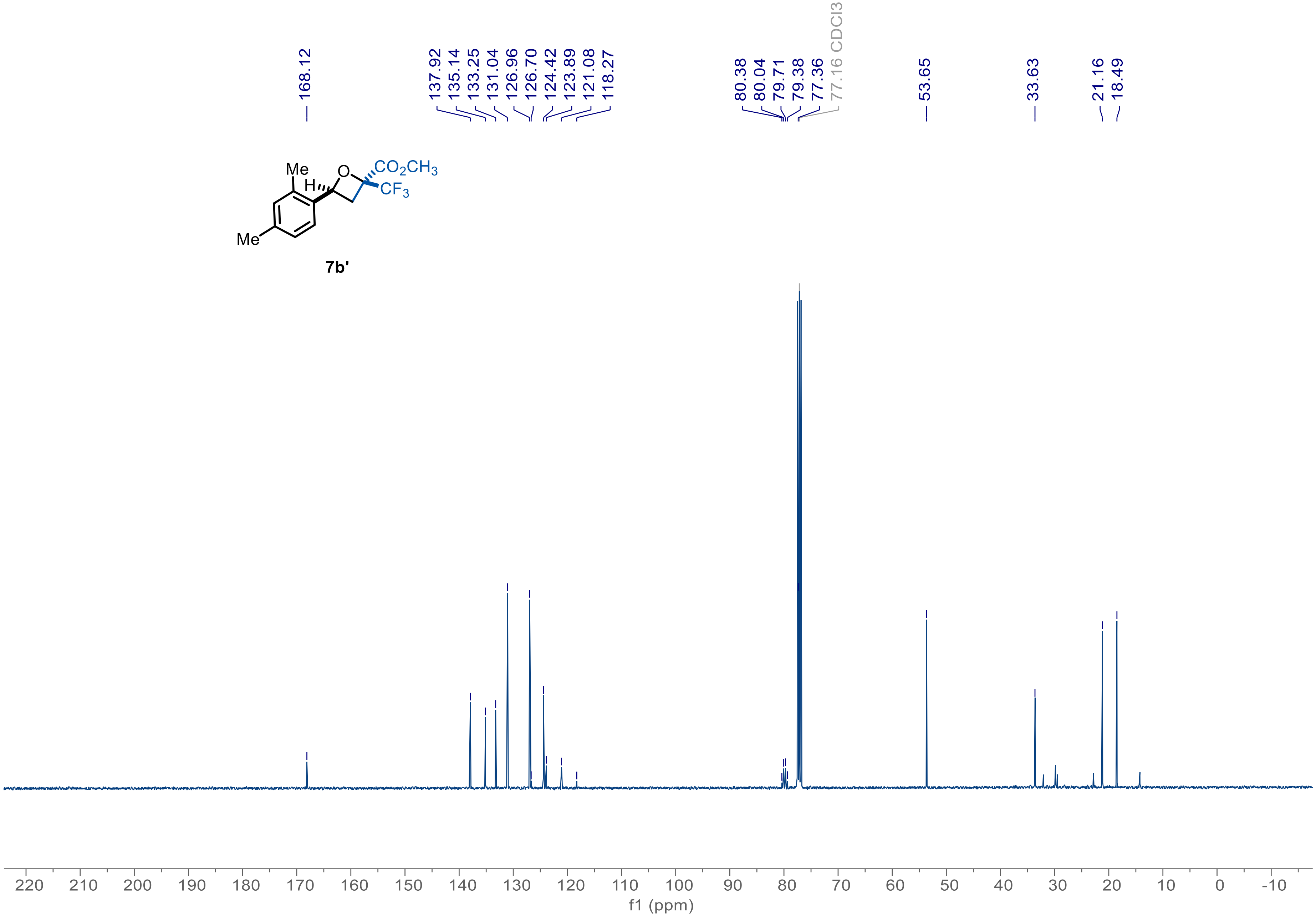

^{19}F NMR (282 MHz, $CDCl_3$) of **7b’**

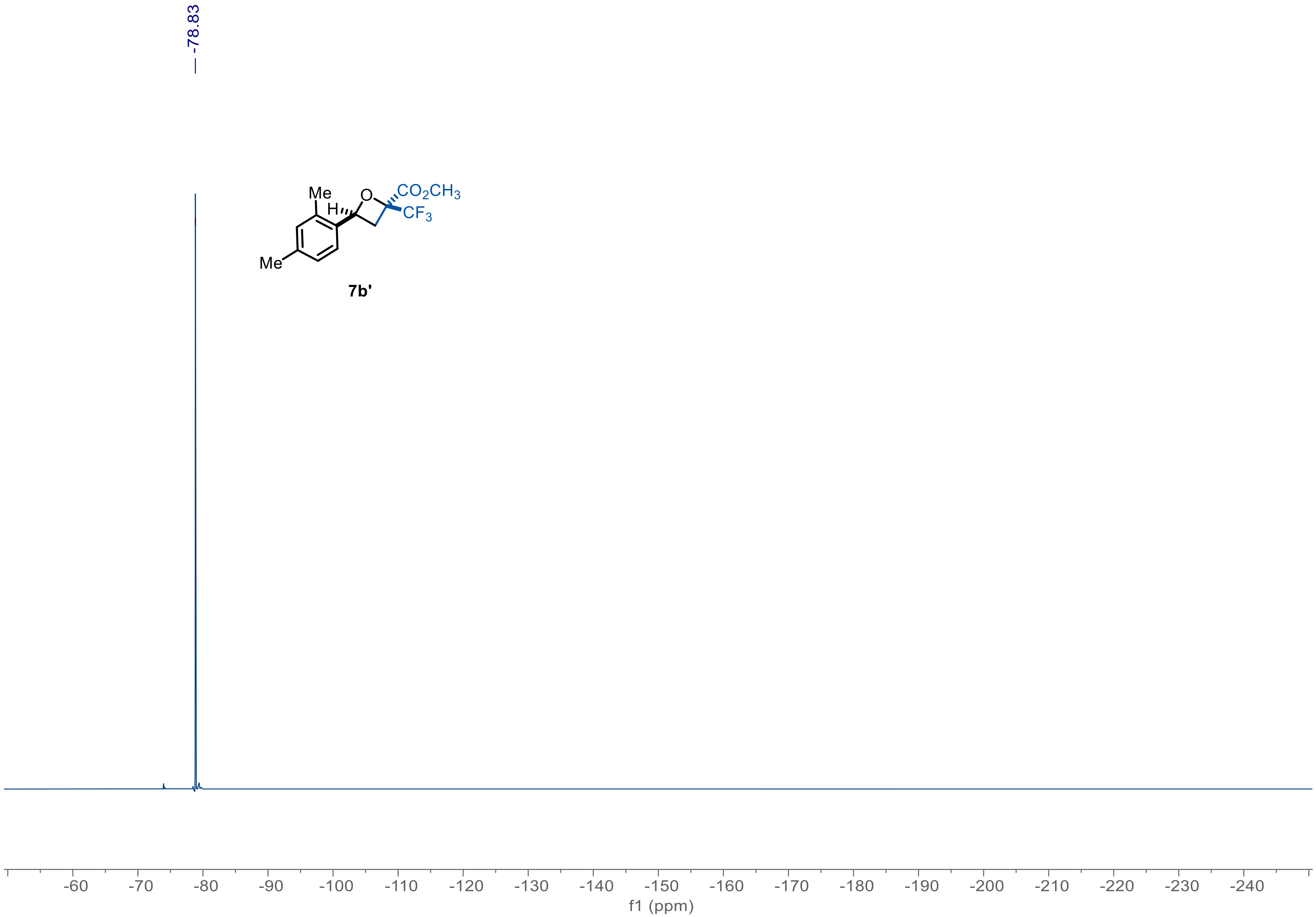

^{1}H NMR (300 MHz, $CDCl_3$) of **8a**

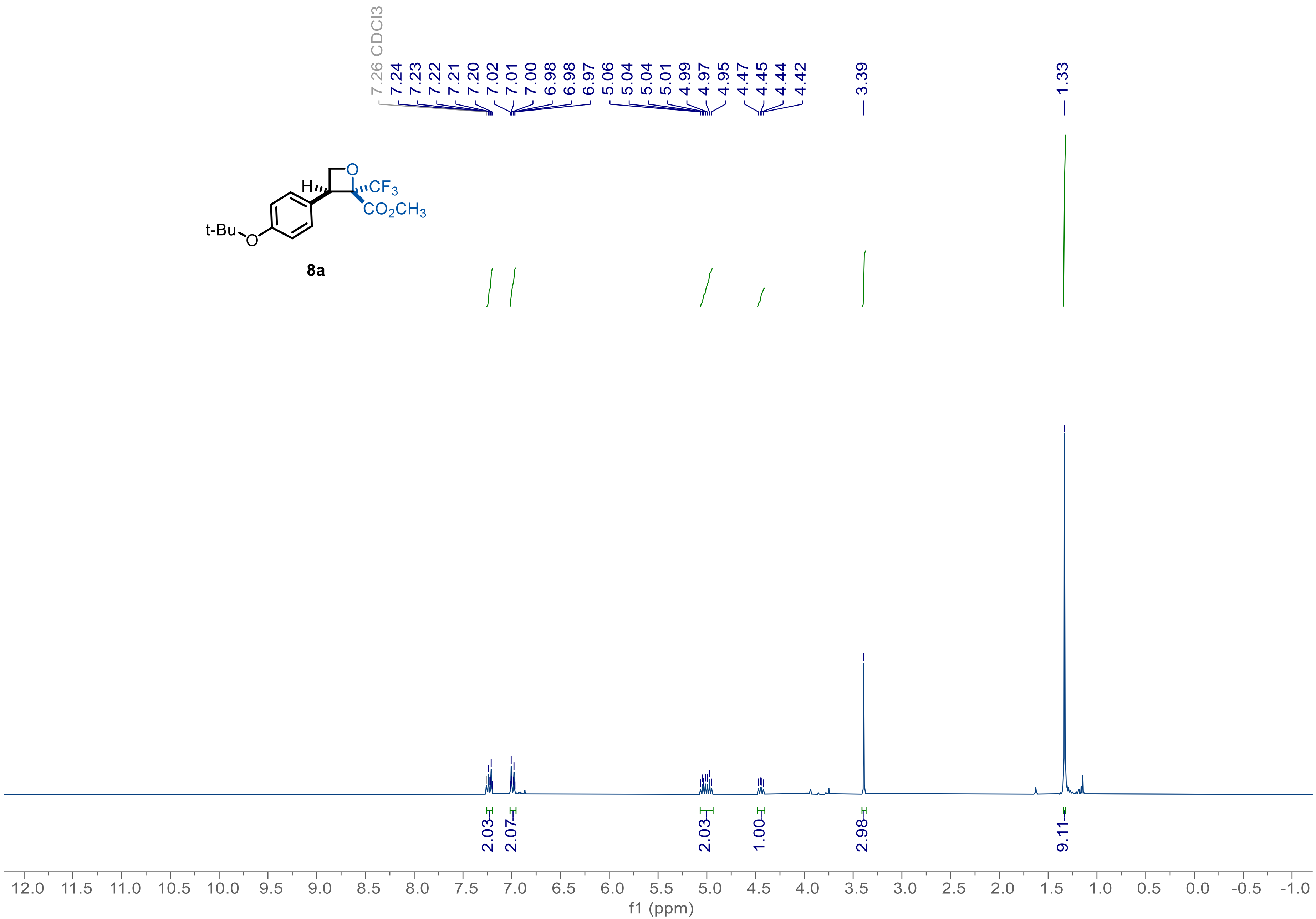

^{13}C NMR (75 MHz, $CDCl_3$) of **8a**

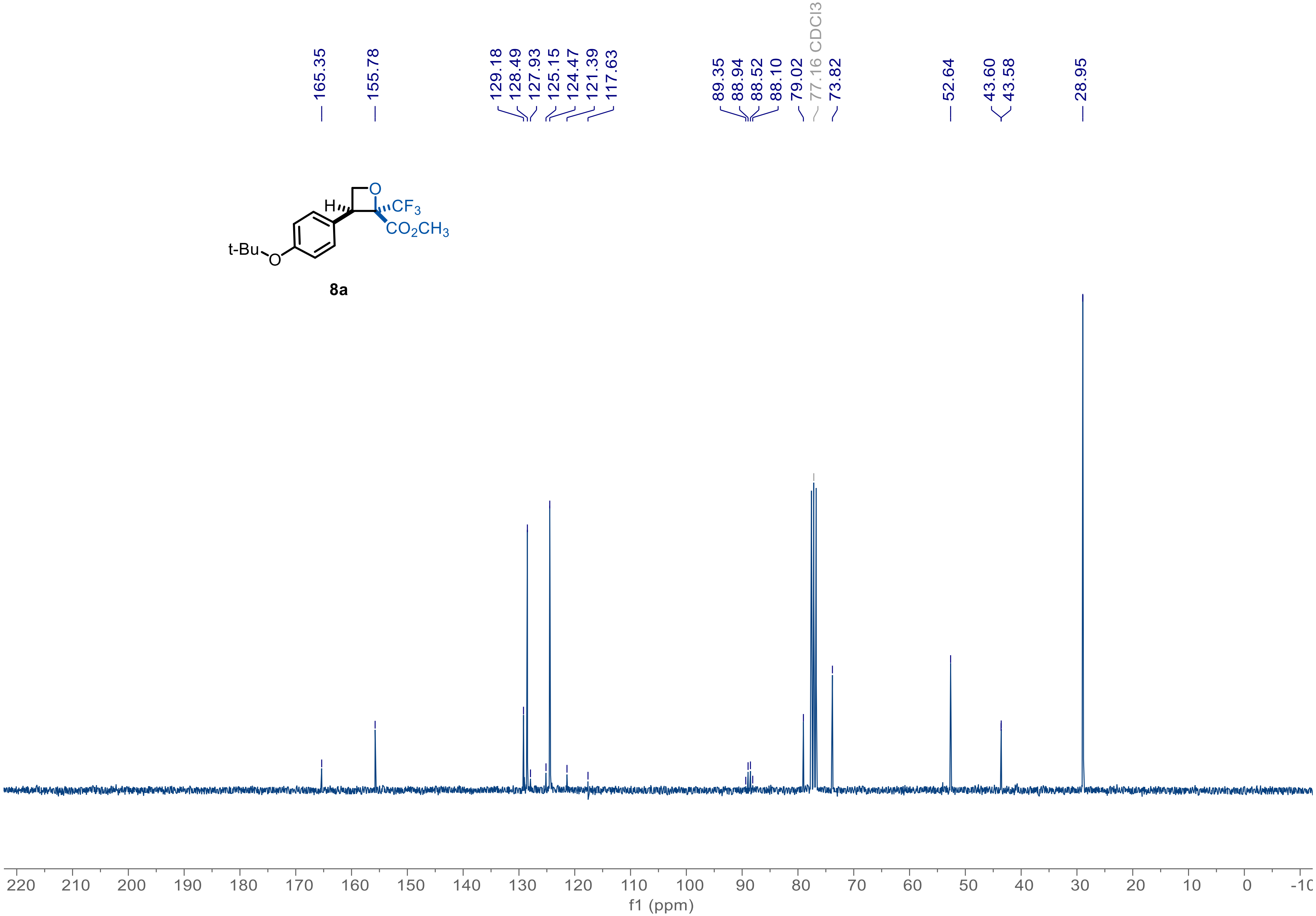

^{19}F NMR (282 MHz, $CDCl_3$) of **8a**

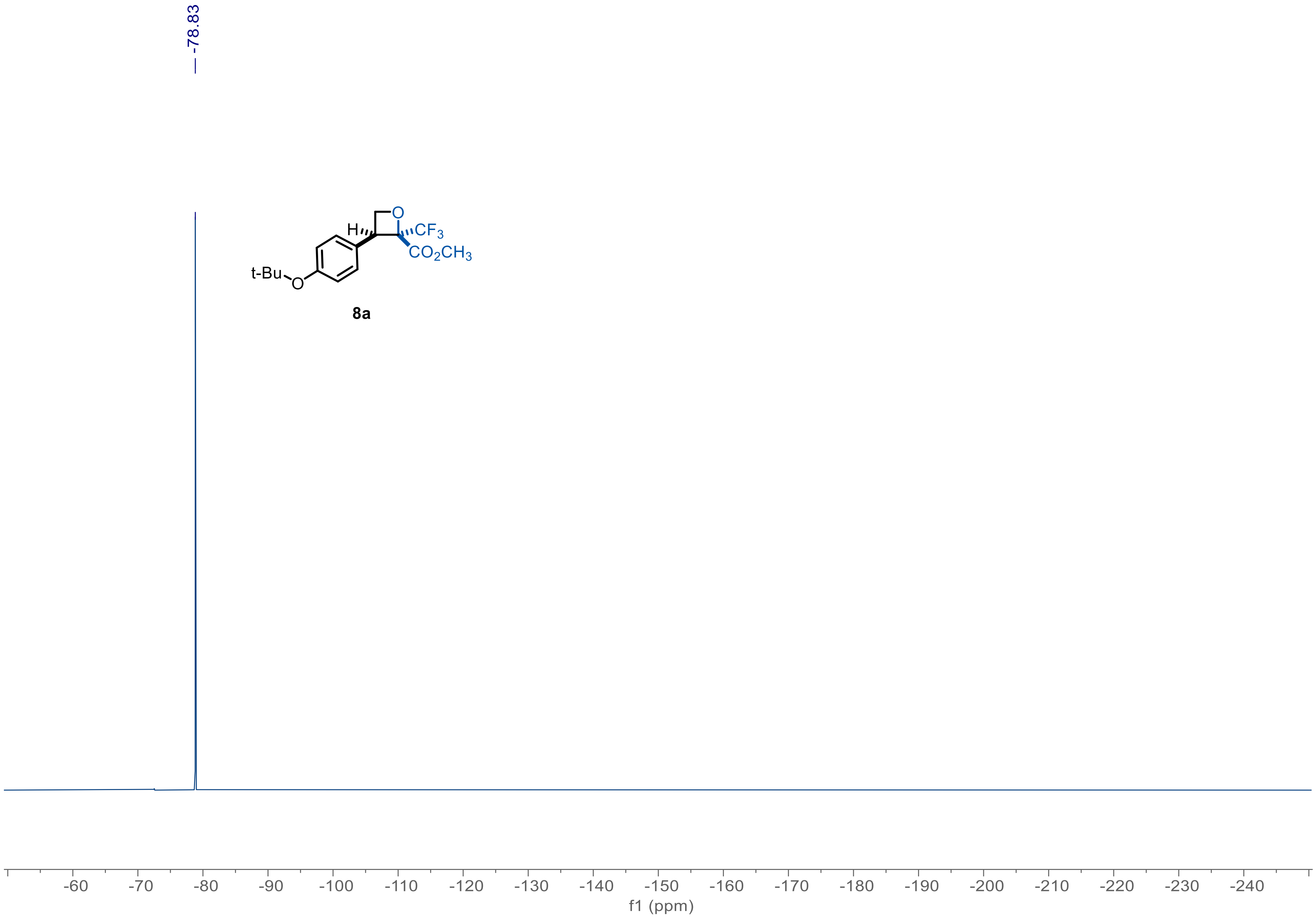

^{1}H NMR (300 MHz, $CDCl_3$) of **8a’**

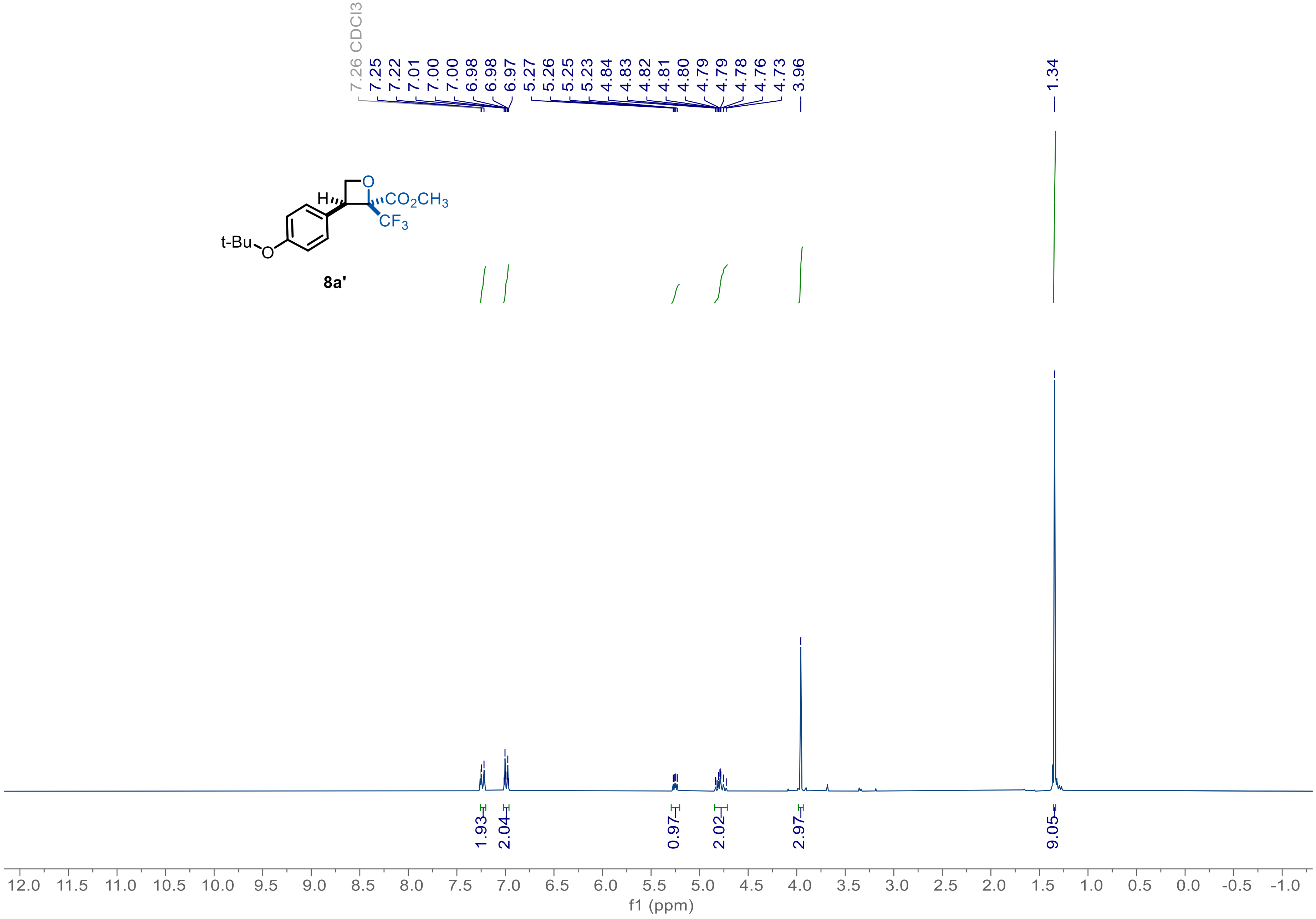

^{13}C NMR (75 MHz, $CDCl_3$) of **8a'**

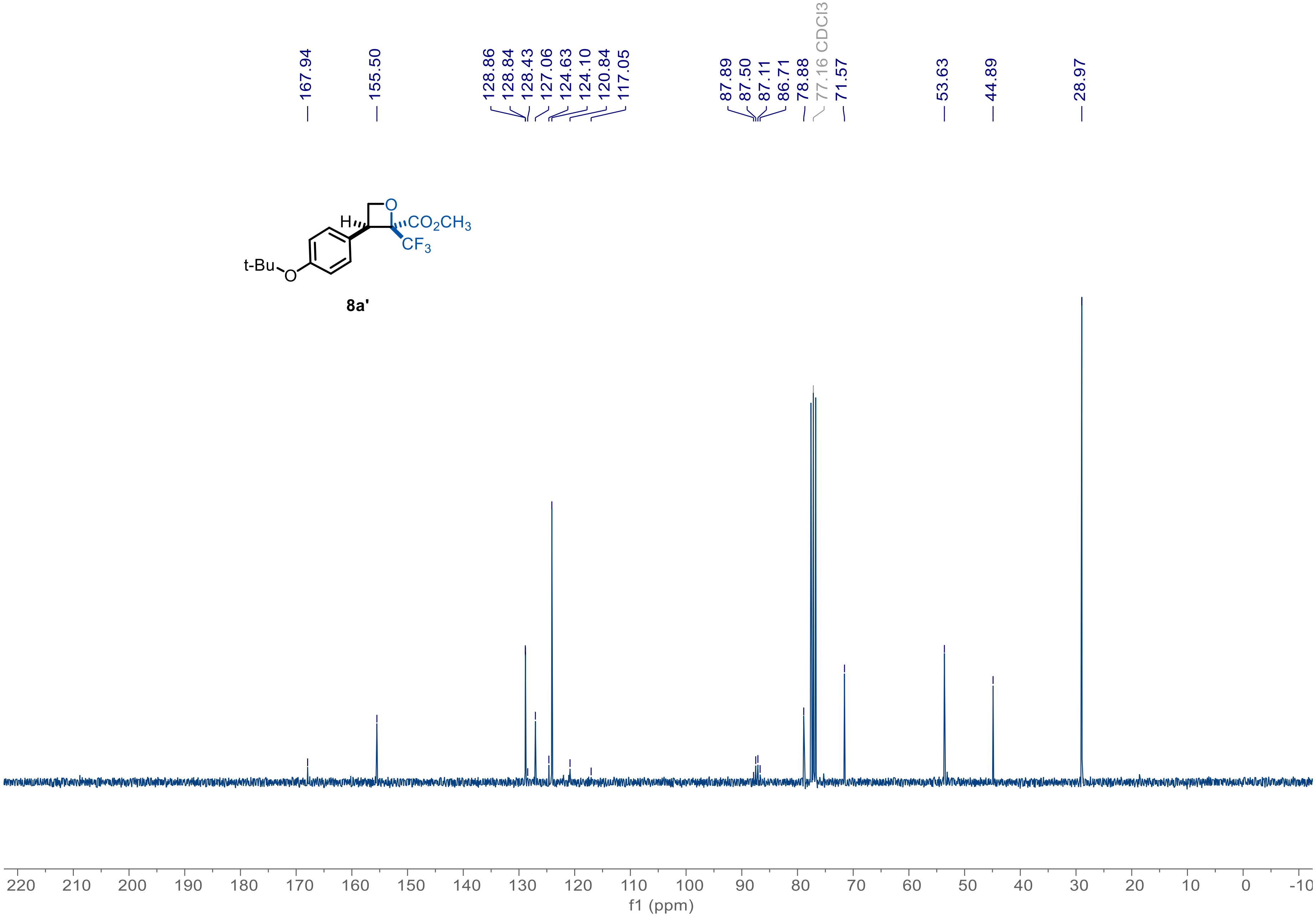

^{19}F NMR (282 MHz, $CDCl_3$) of **8a’**

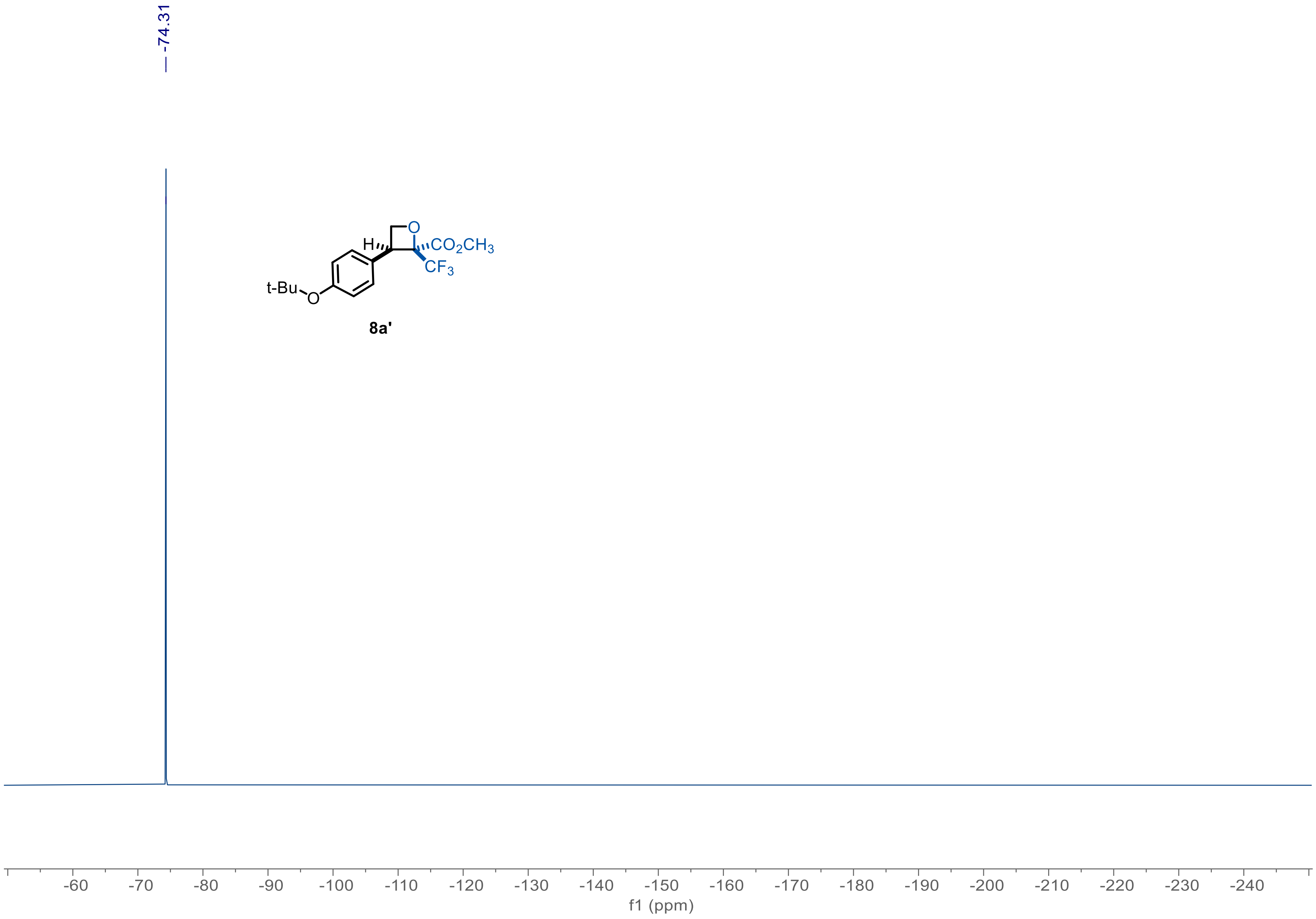

^{1}H NMR (300 MHz, $CDCl_3$) of **9a**

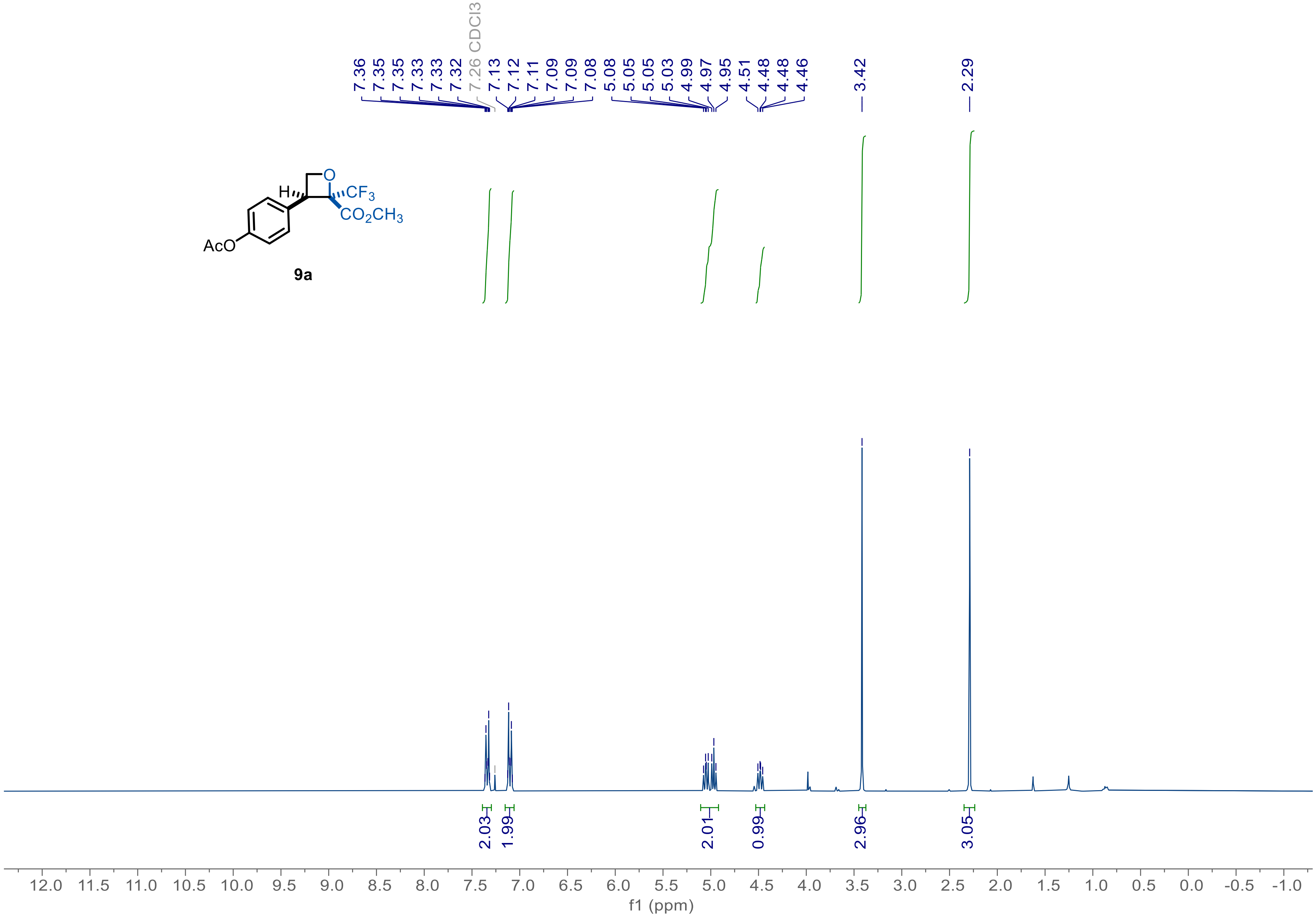

^{13}C NMR (75 MHz, $CDCl_3$) of **9a**

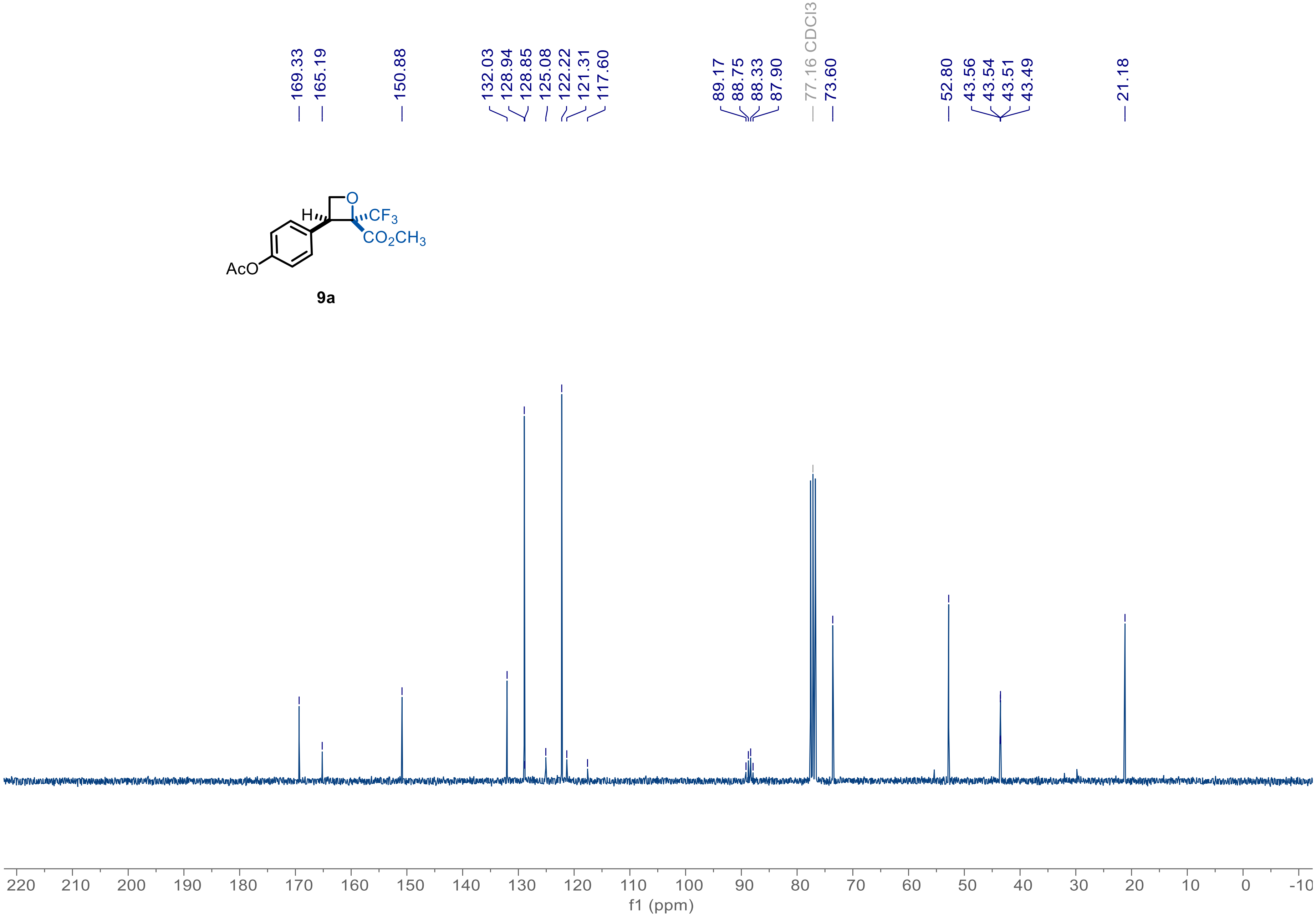

^{19}F NMR (282 MHz, $CDCl_3$) of **9a**

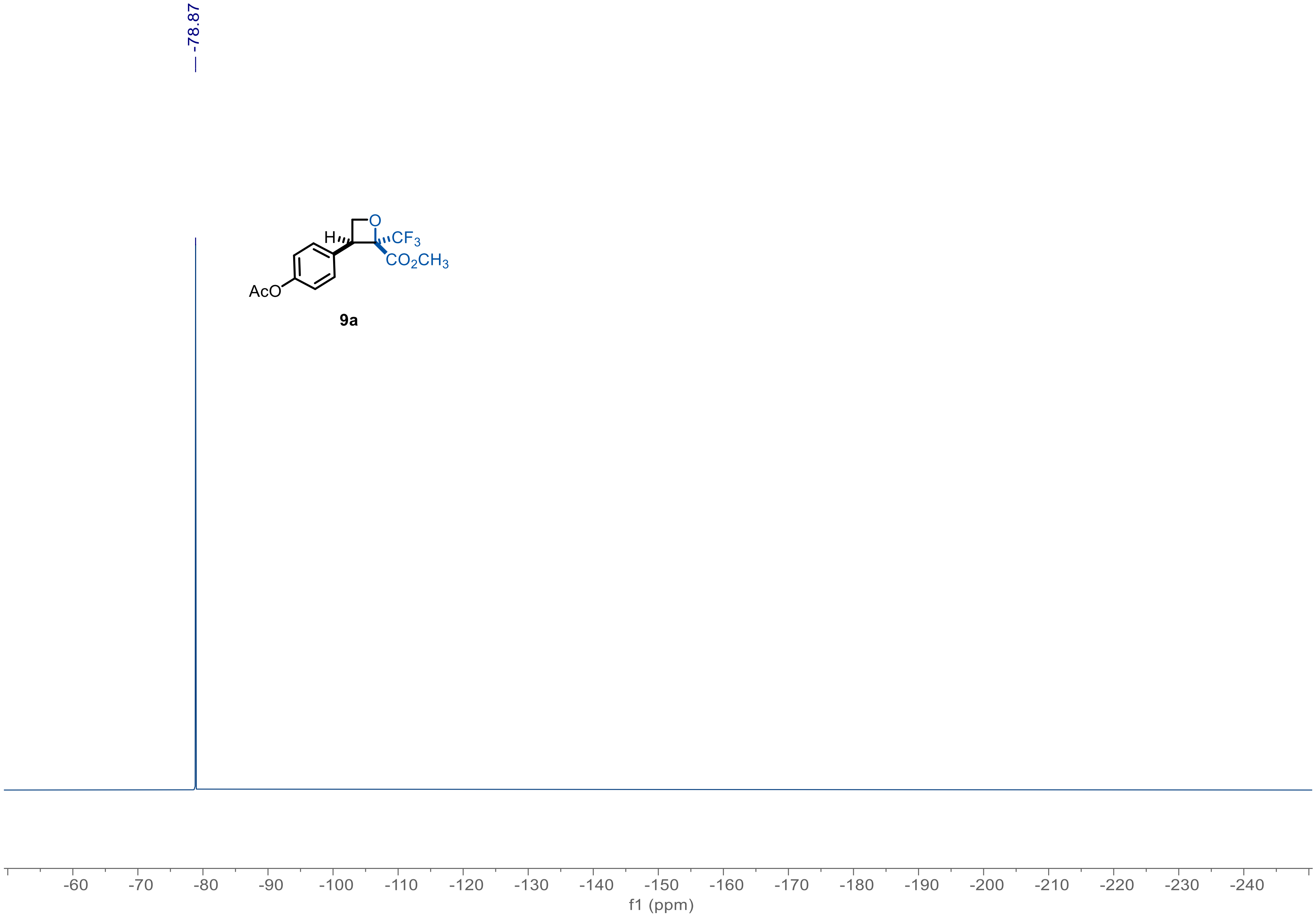

^1H NMR (300 MHz, $CDCl_3$) of **9a'**

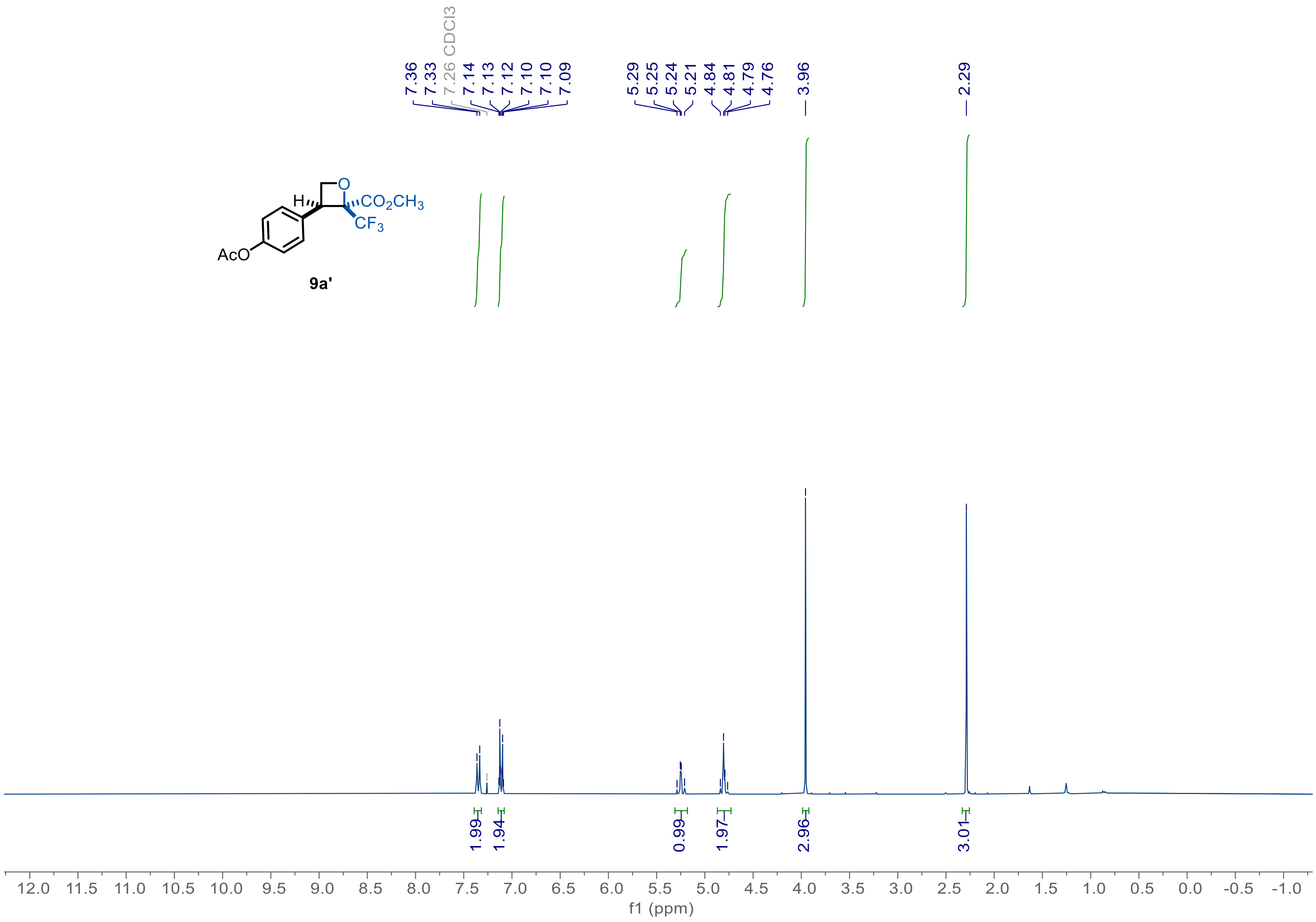

^{13}C NMR (75 MHz, $CDCl_3$) of **9a’**

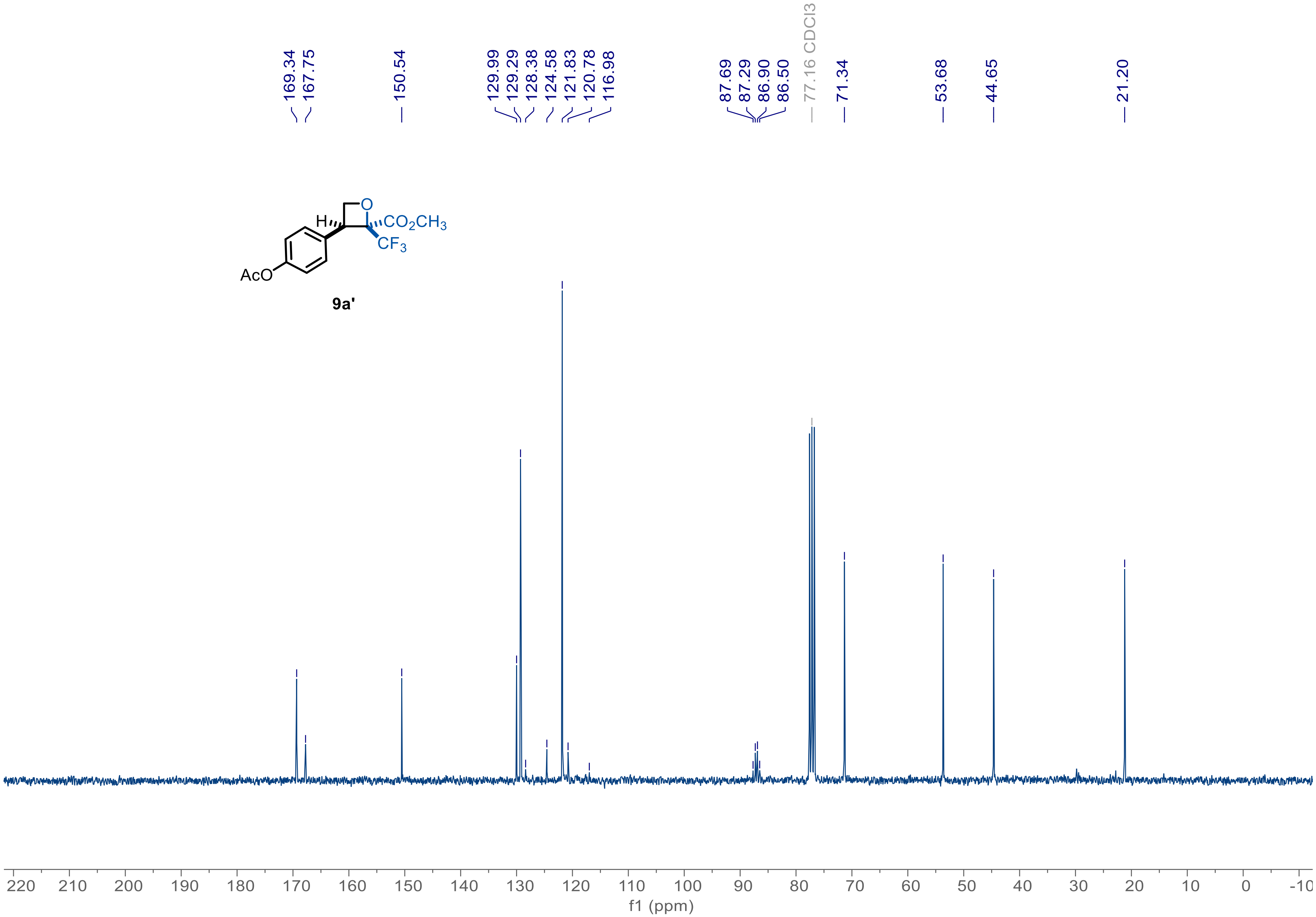

^{19}F NMR (282 MHz, $CDCl_3$) of **9a'**

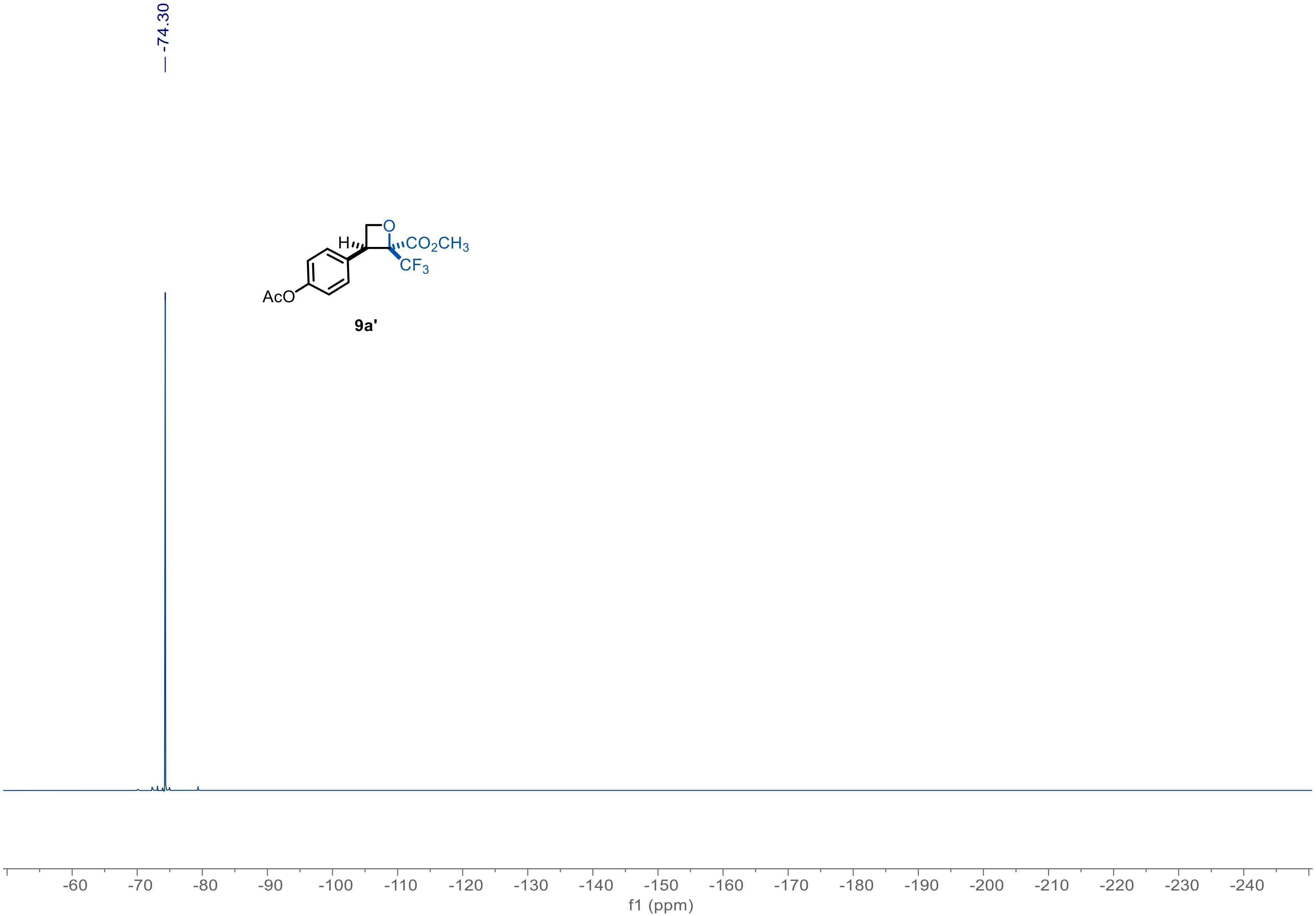

^{1}H NMR (300 MHz, $CDCl_3$) of **9b**

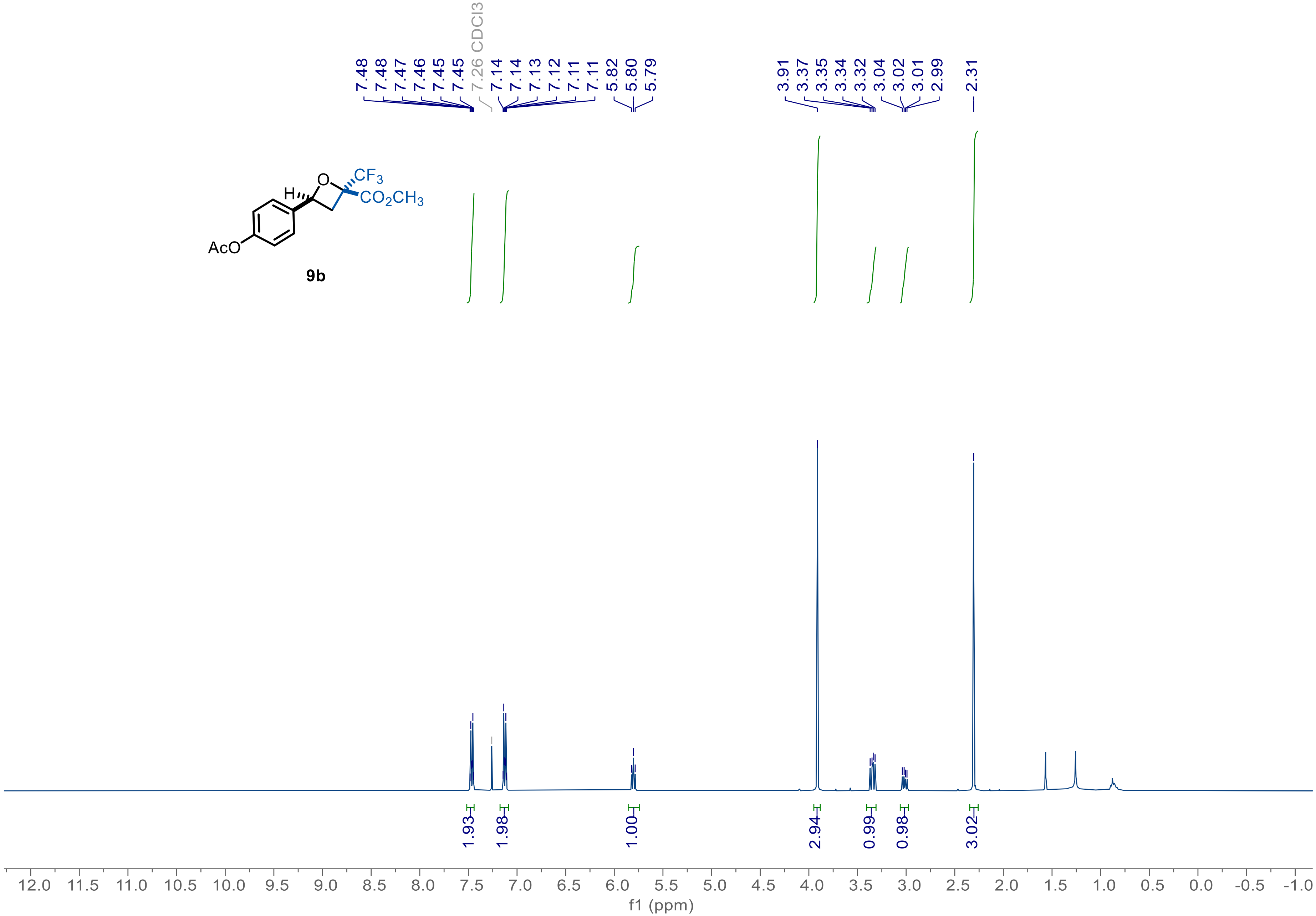

^{13}C NMR (101 MHz, $CDCl_3$) of **9b**

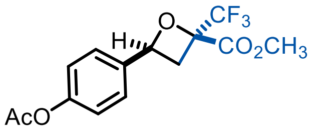

9b

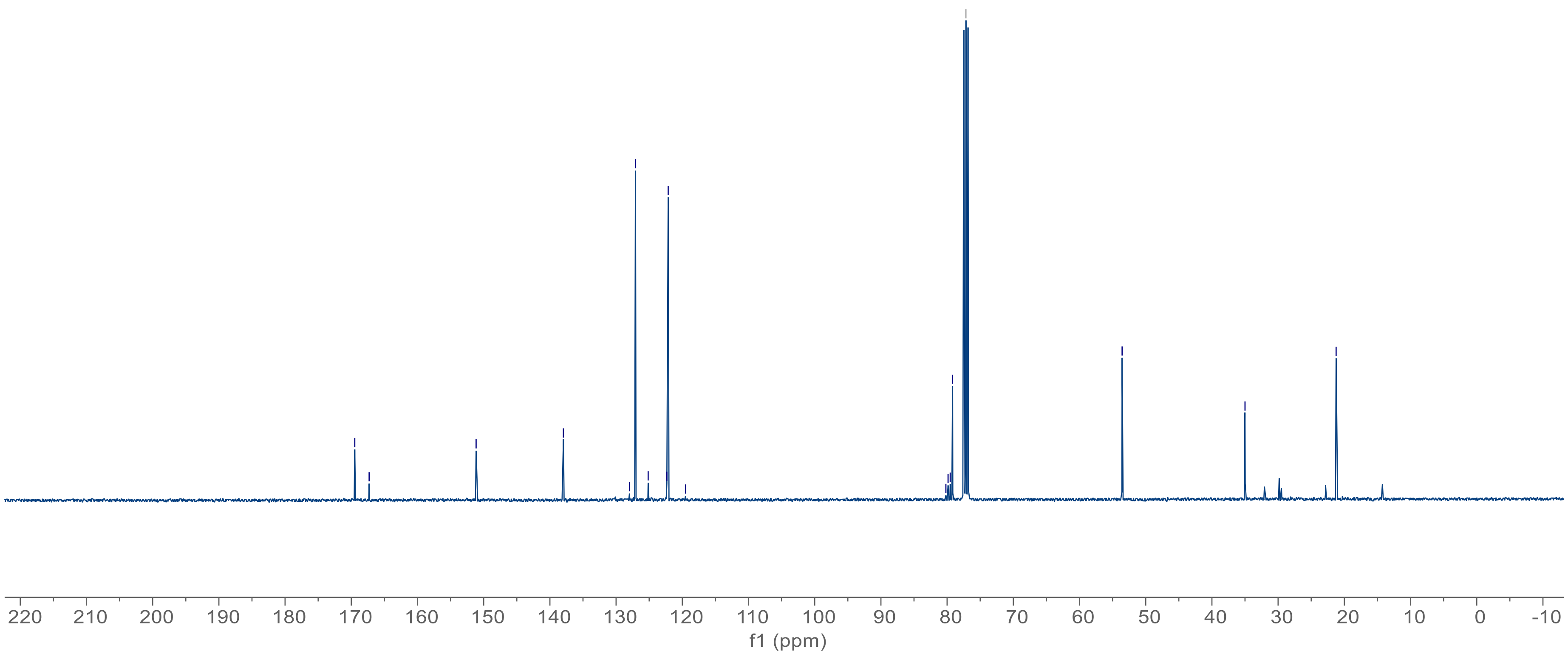

^{19}F NMR (282 MHz, $CDCl_3$) of **9b**

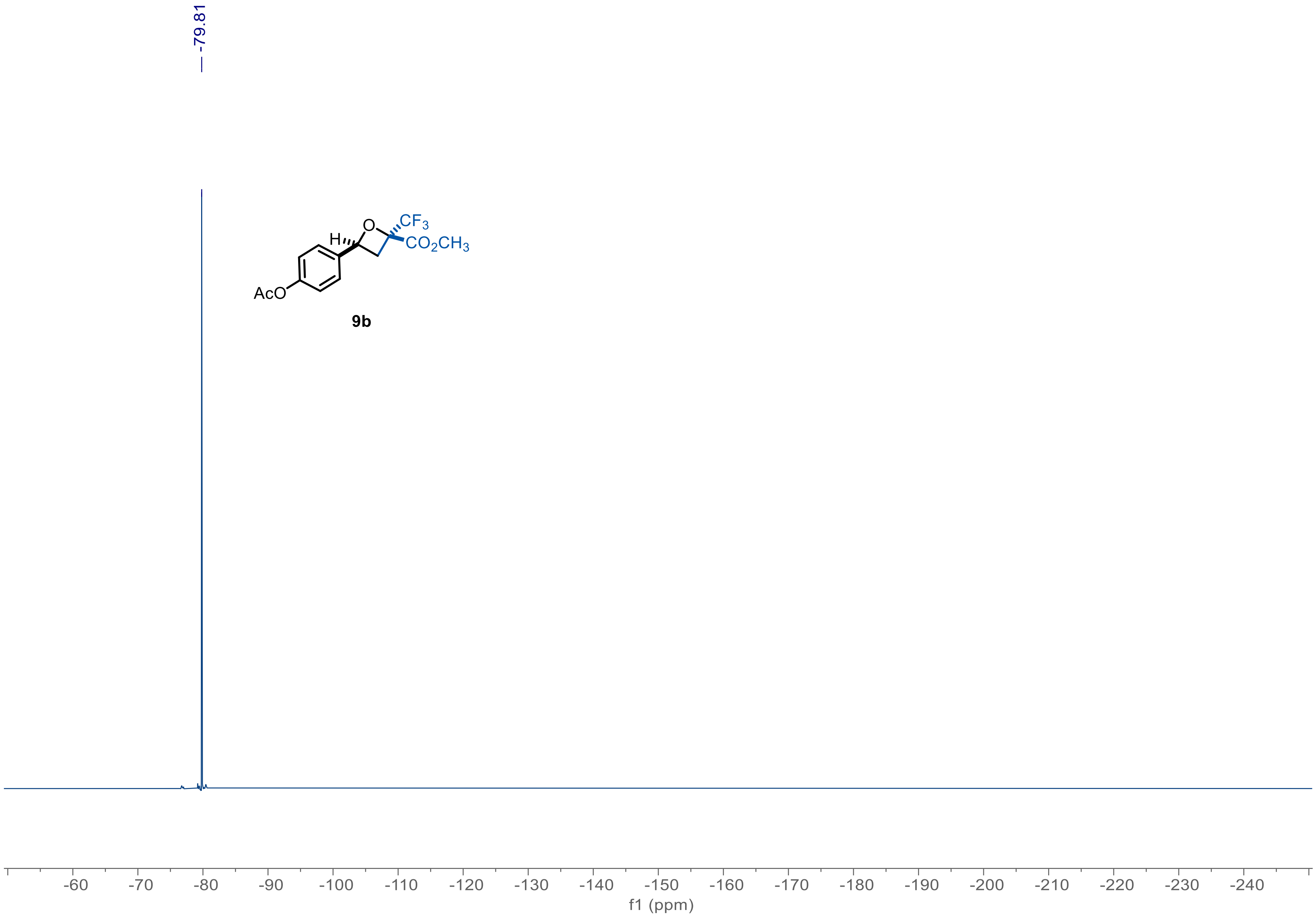

^{1}H NMR (300 MHz, $CDCl_3$) of **9b’**

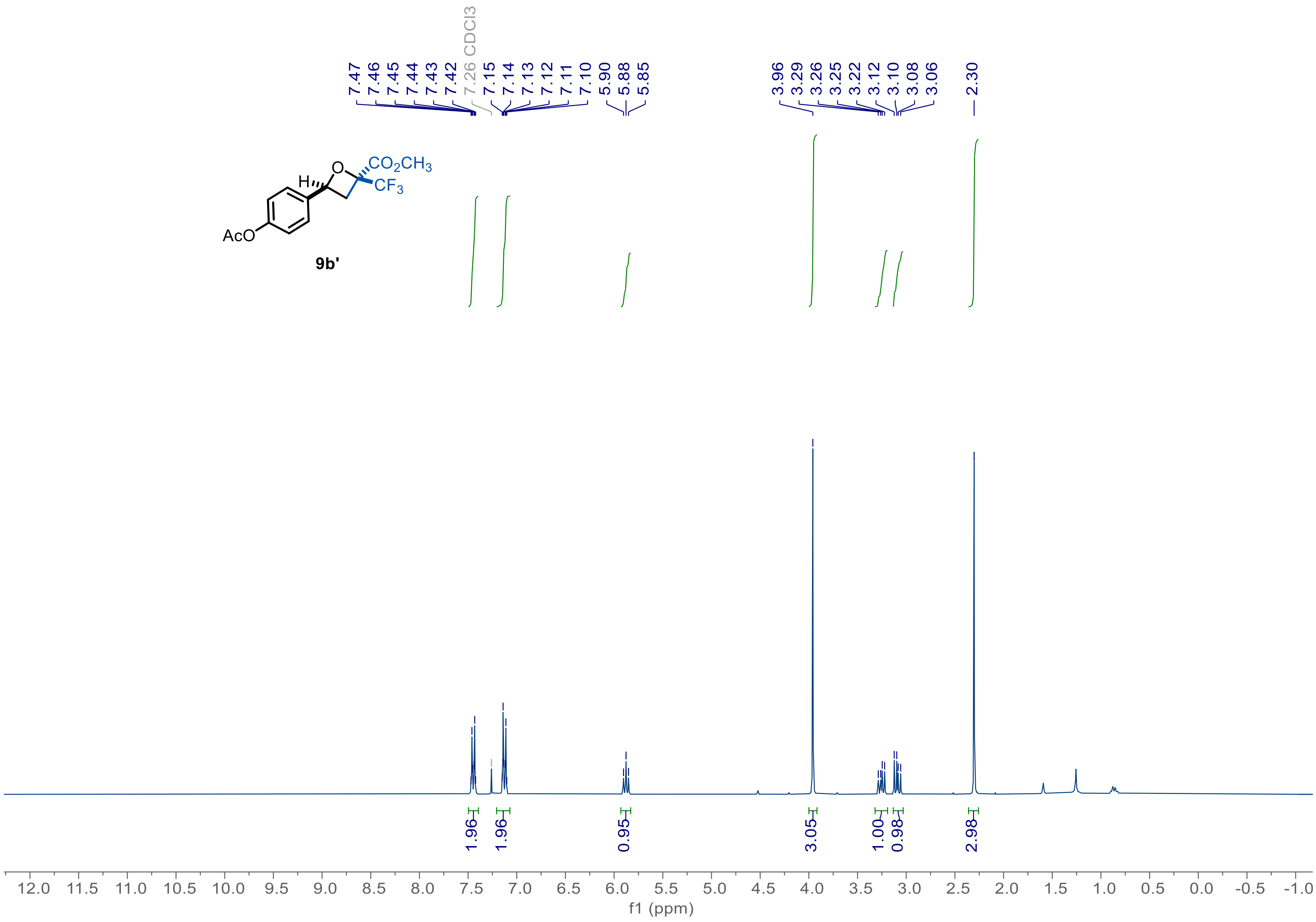

^{13}C NMR (75 MHz, $CDCl_3$) of **9b’**

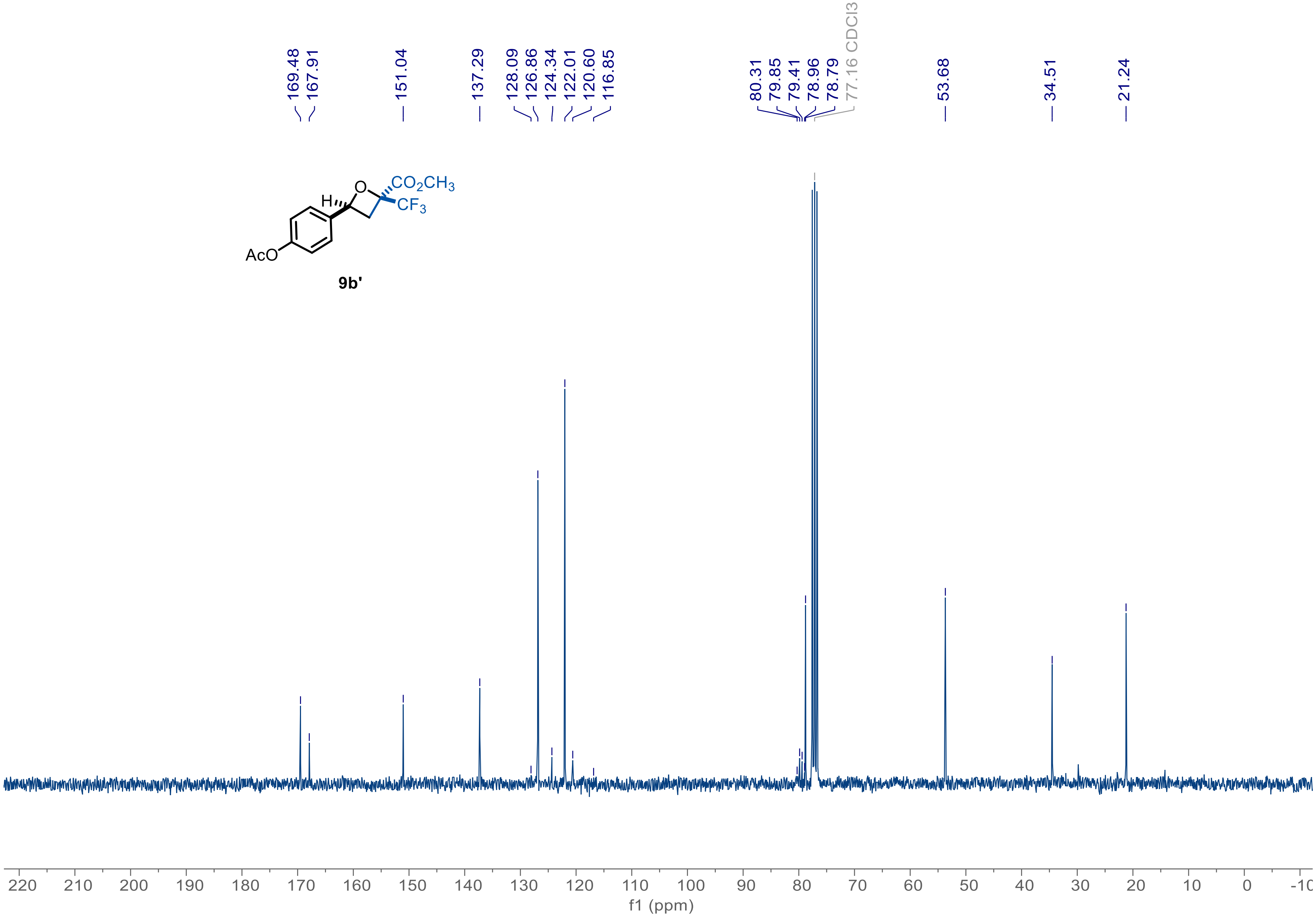

^{19}F NMR (282 MHz, $CDCl_3$) of **9b’**

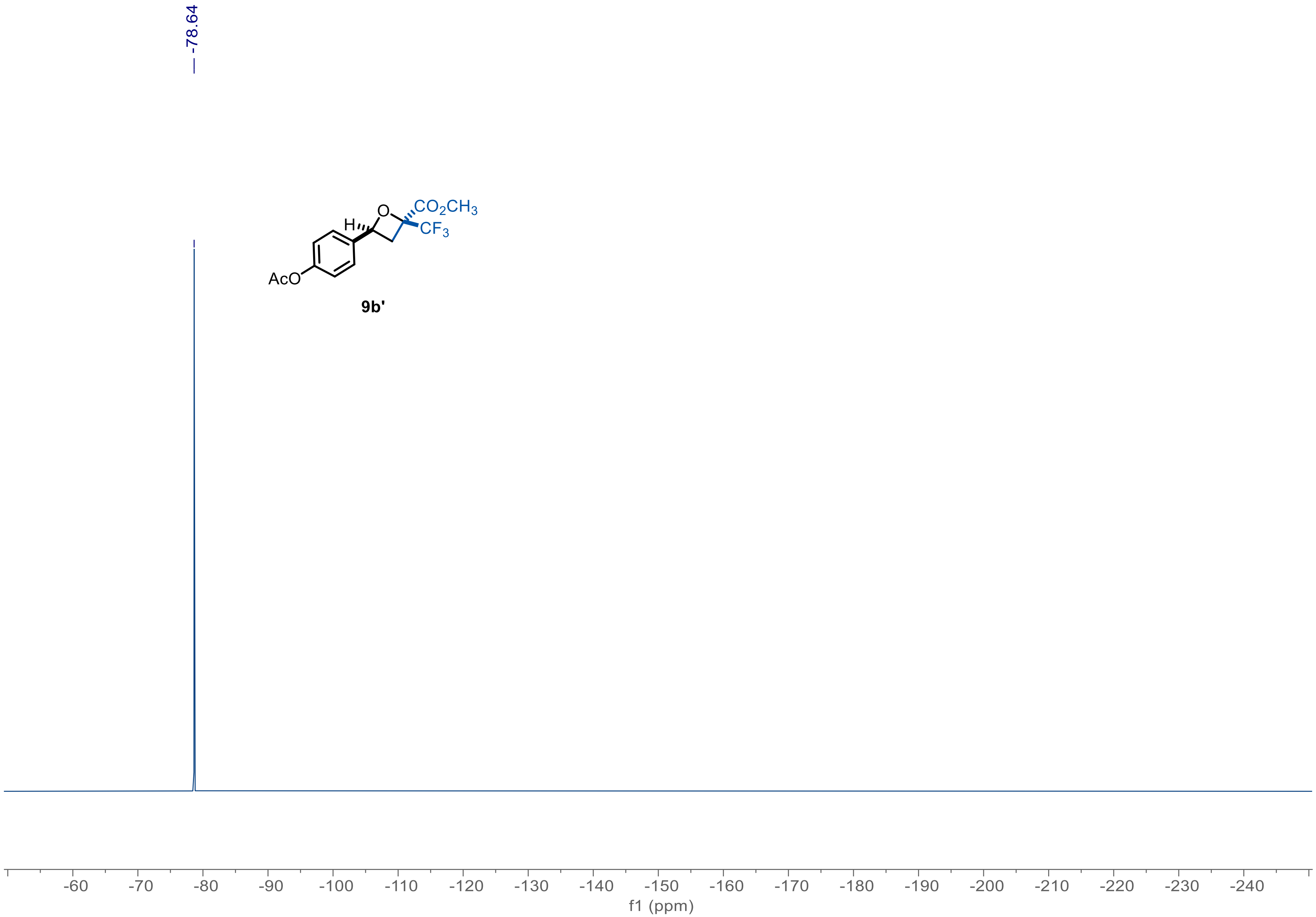

^{1}H NMR (300 MHz, $CDCl_3$) of **10a**

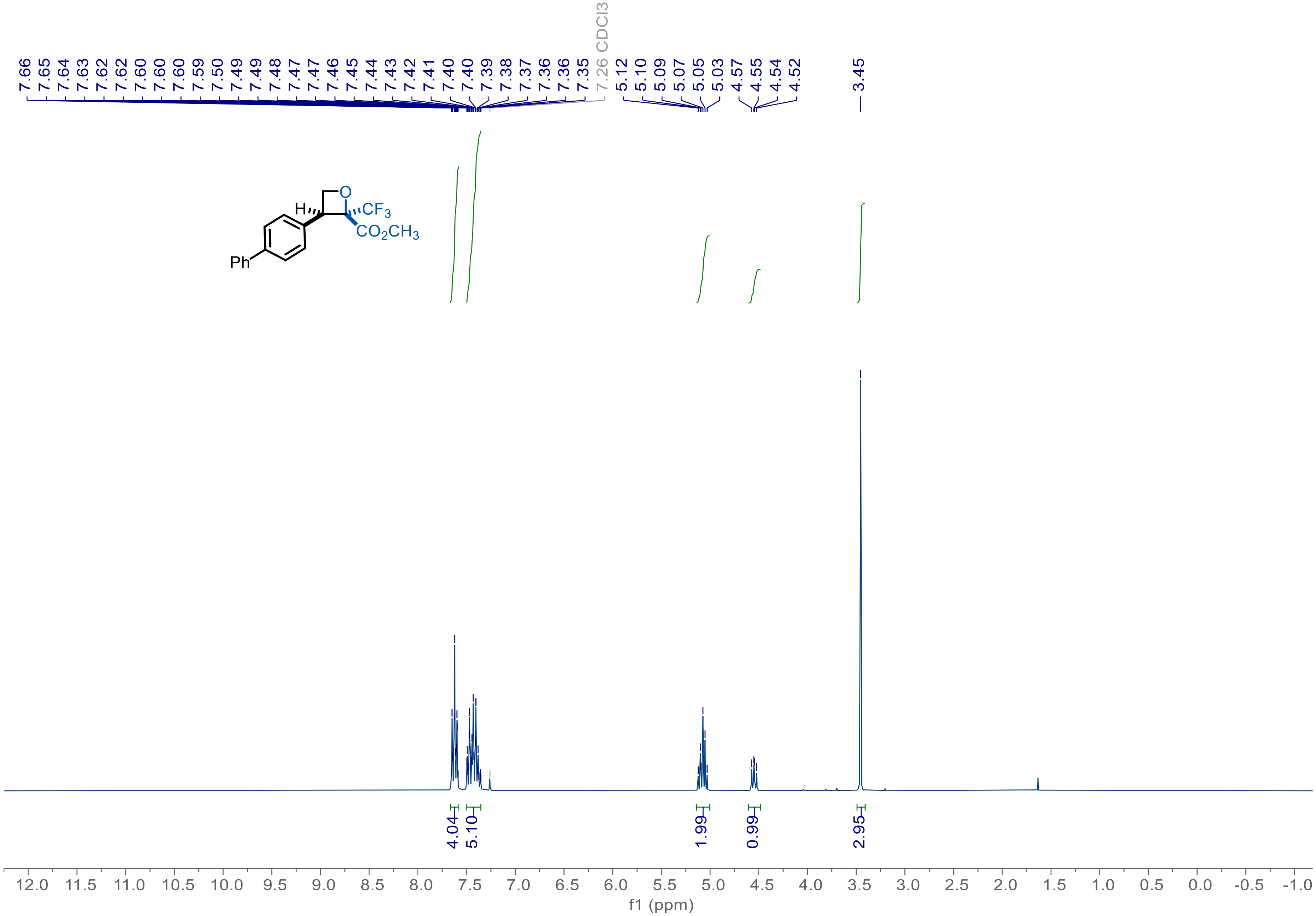

^{13}C NMR (75 MHz, $CDCl_3$) of **10a**

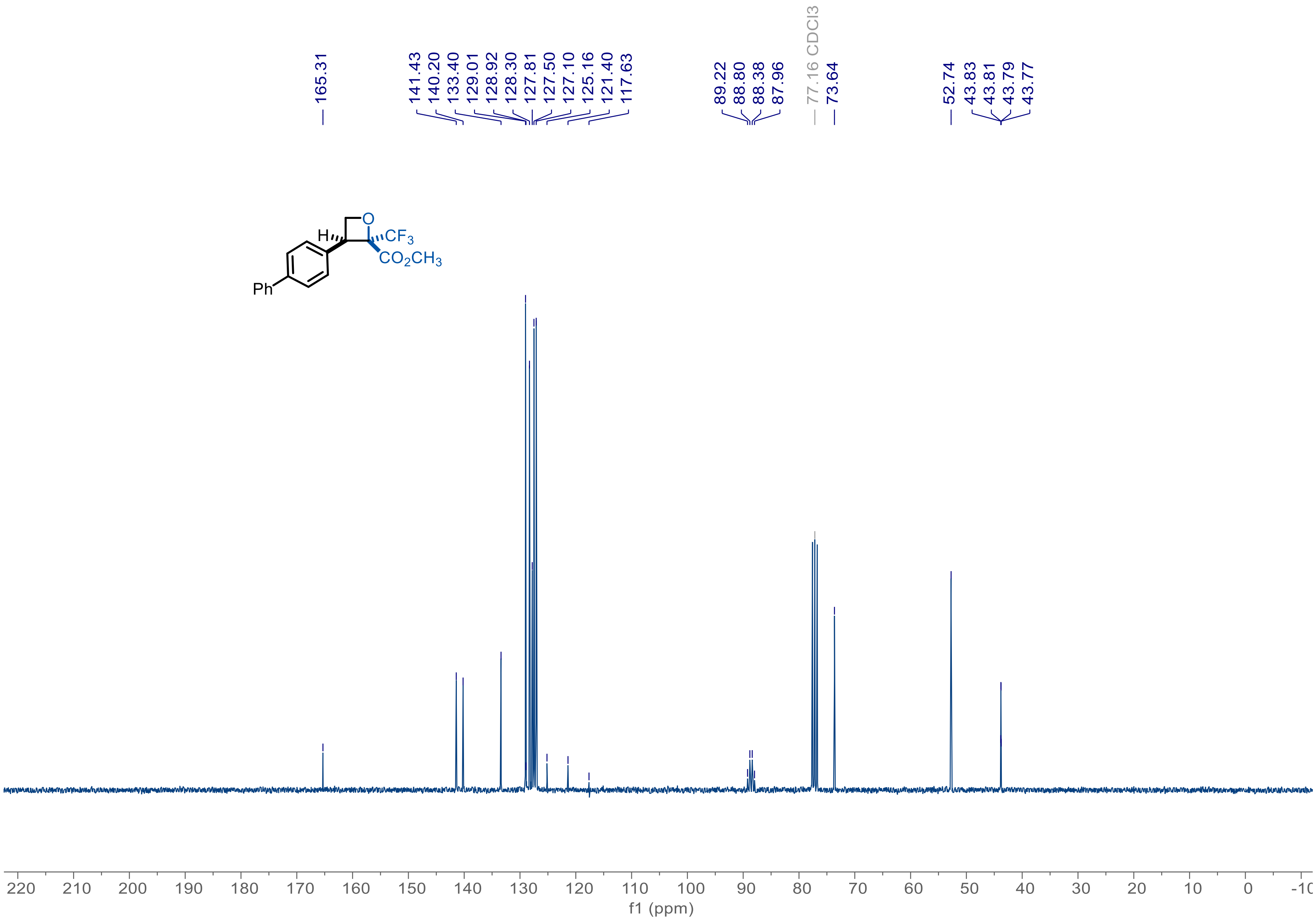

^{19}F NMR (282 MHz, $CDCl_3$) of **10a**

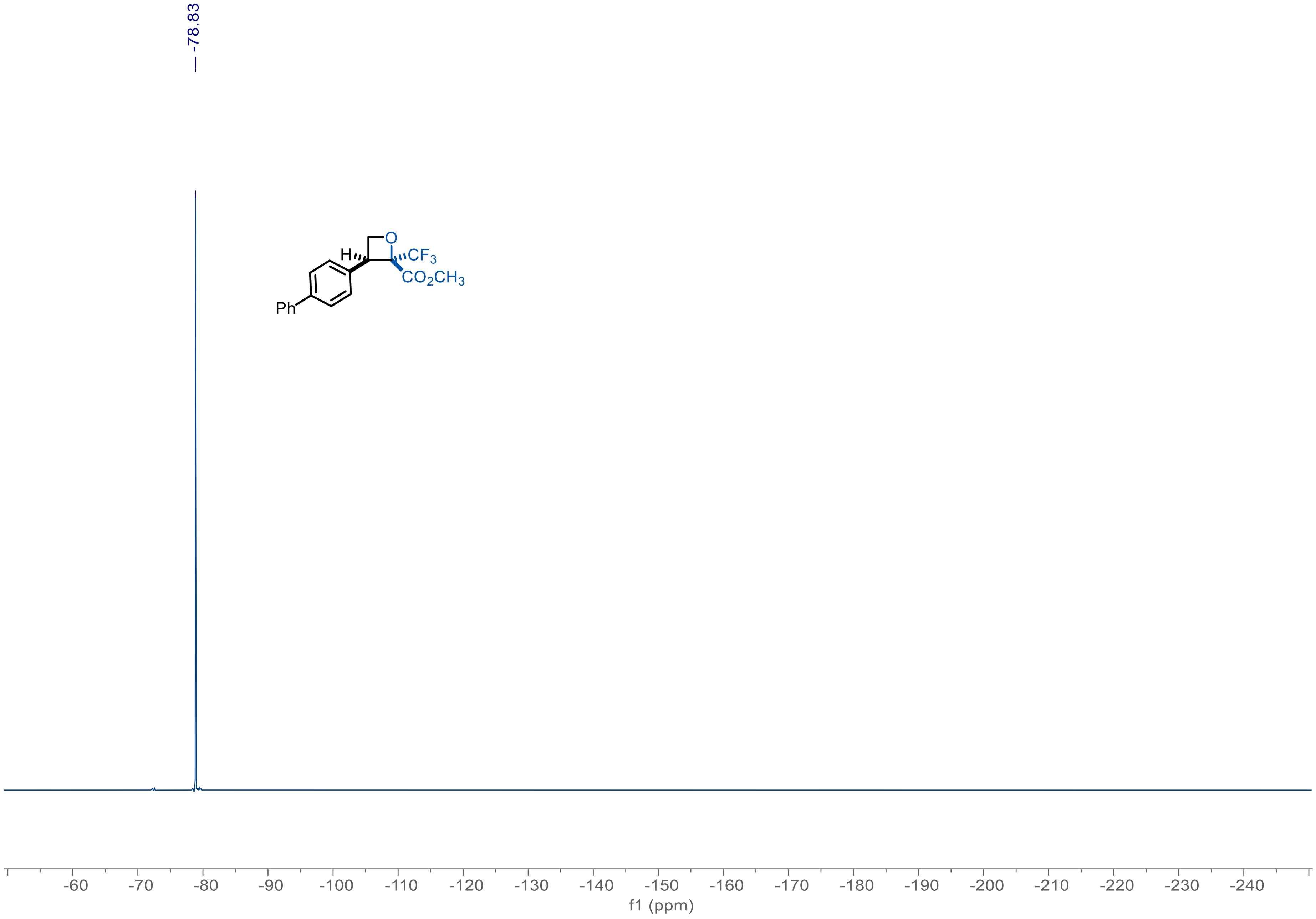

^{1}H NMR (300 MHz, $CDCl_3$) of **10a’**

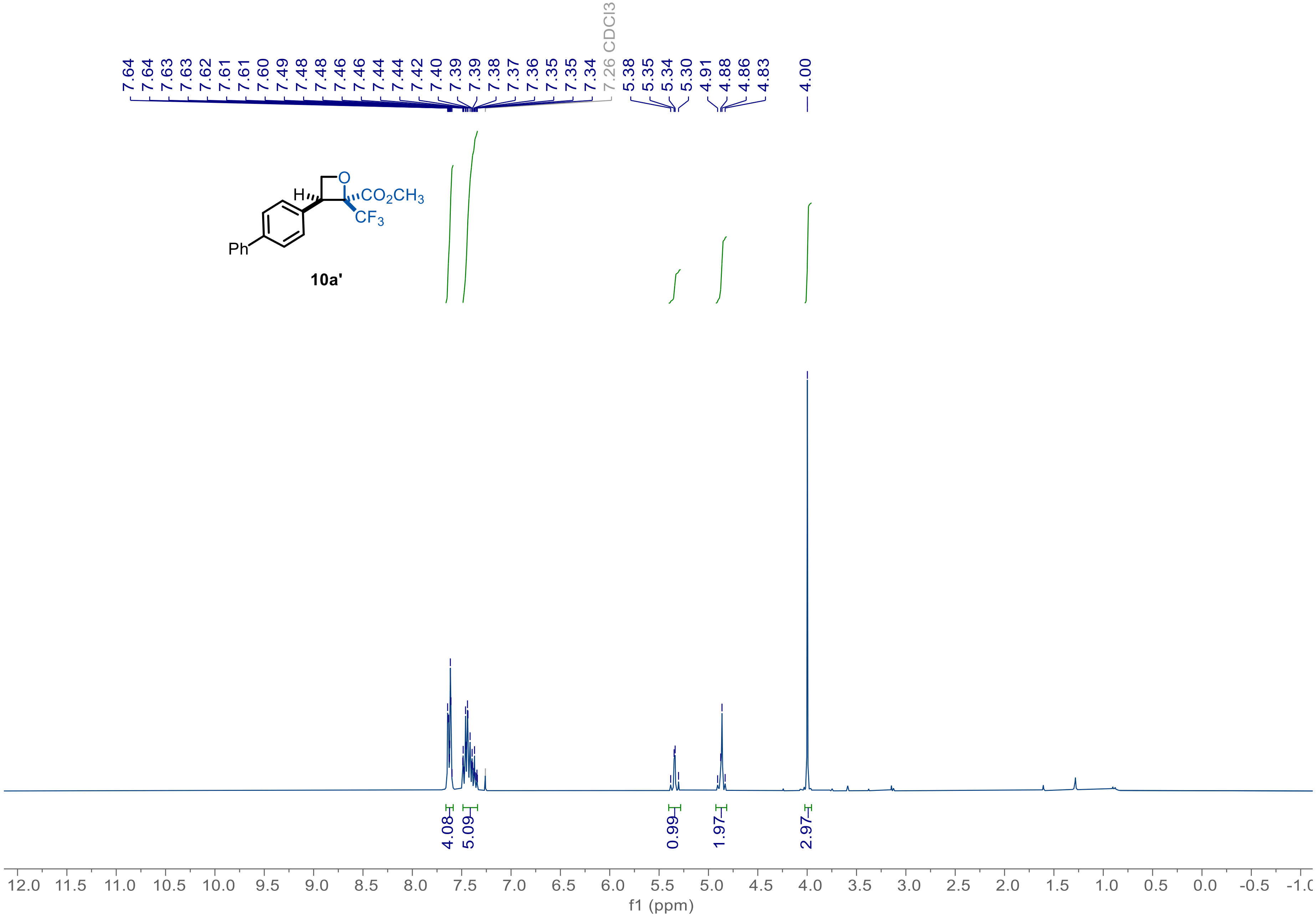

^{13}C NMR (75 MHz, $CDCl_3$) of **10a’**

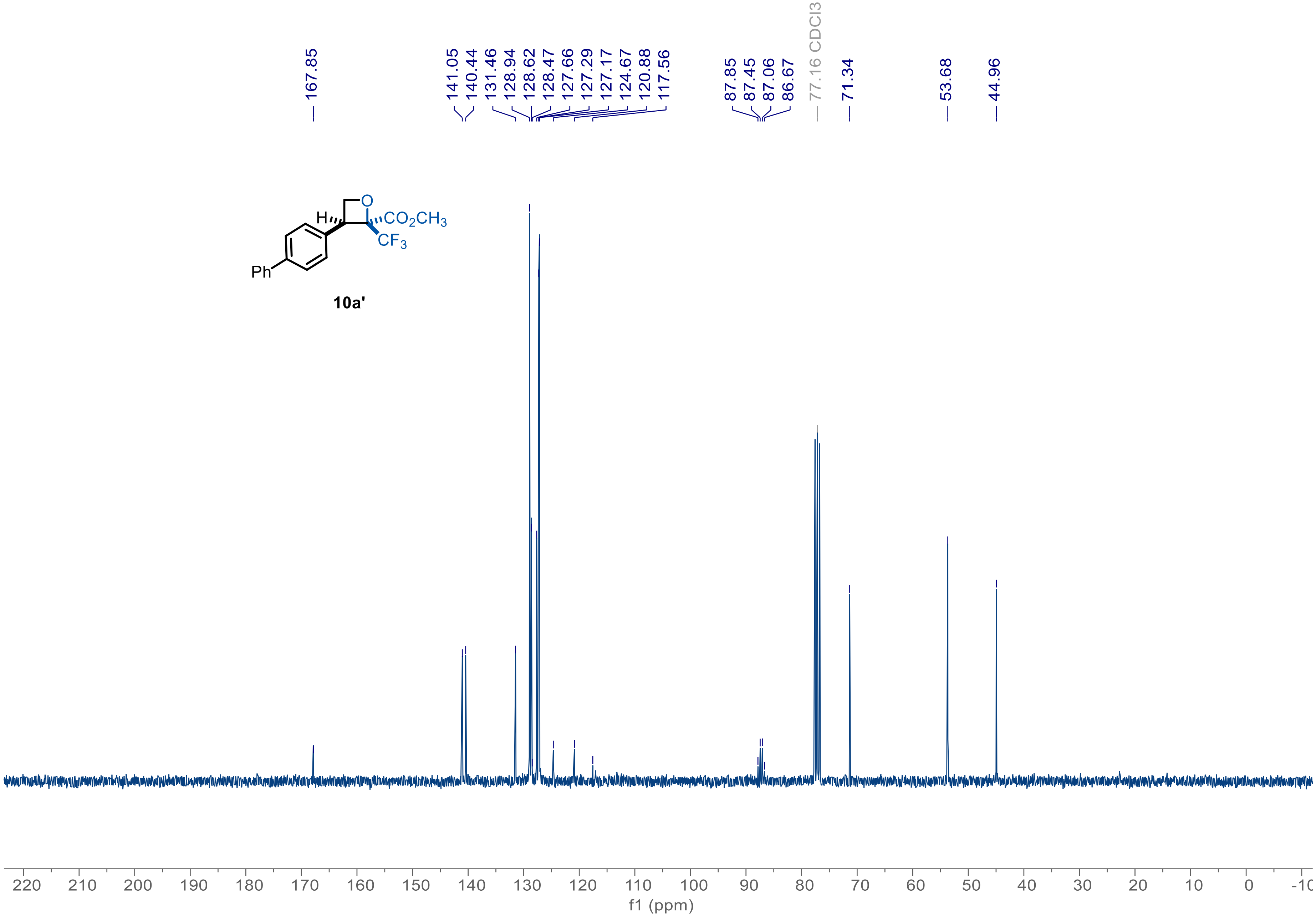

^{19}F NMR (282 MHz, $CDCl_3$) of **10a’**

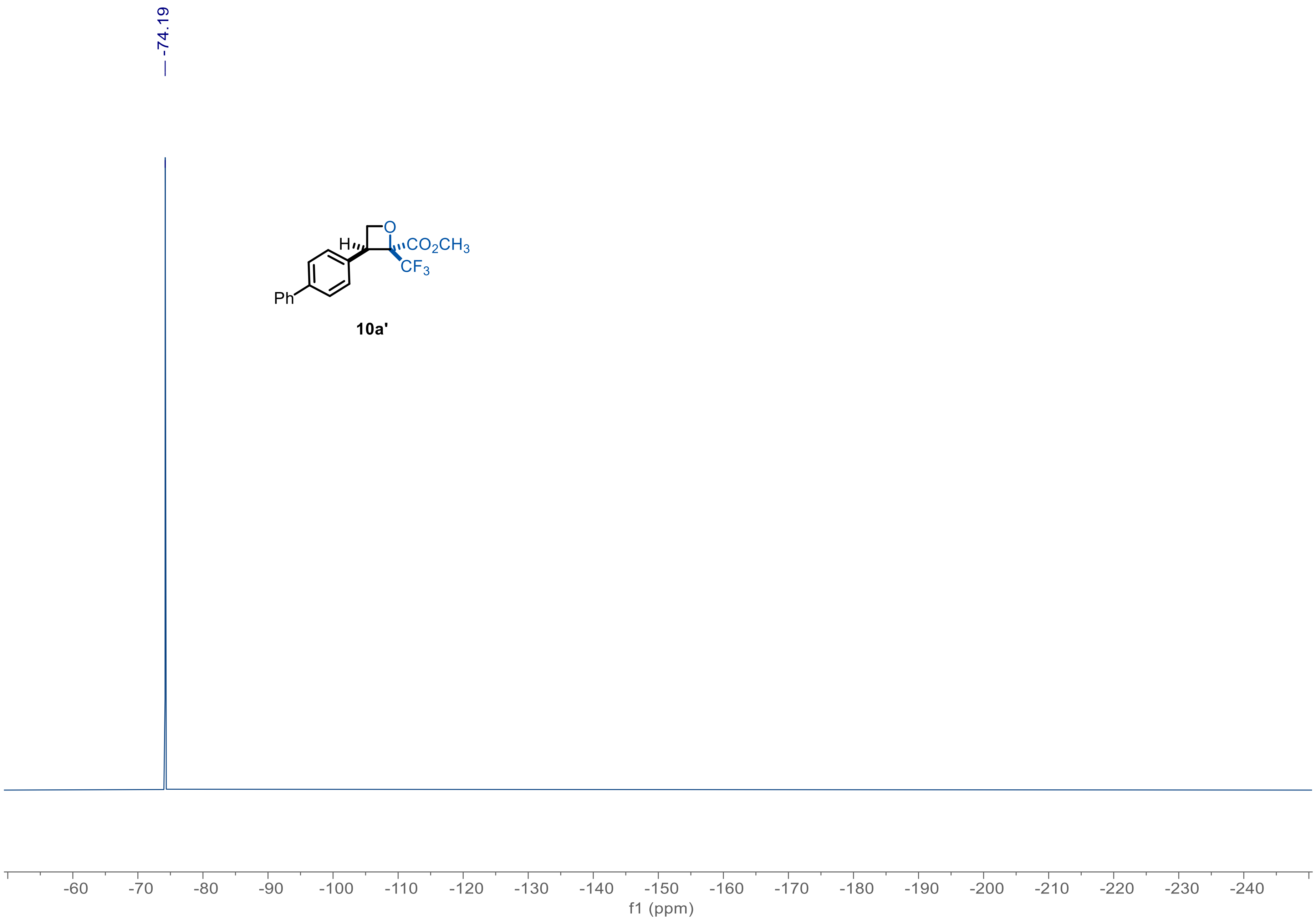

^{1}H NMR (300 MHz, $CDCl_3$) of **10b**

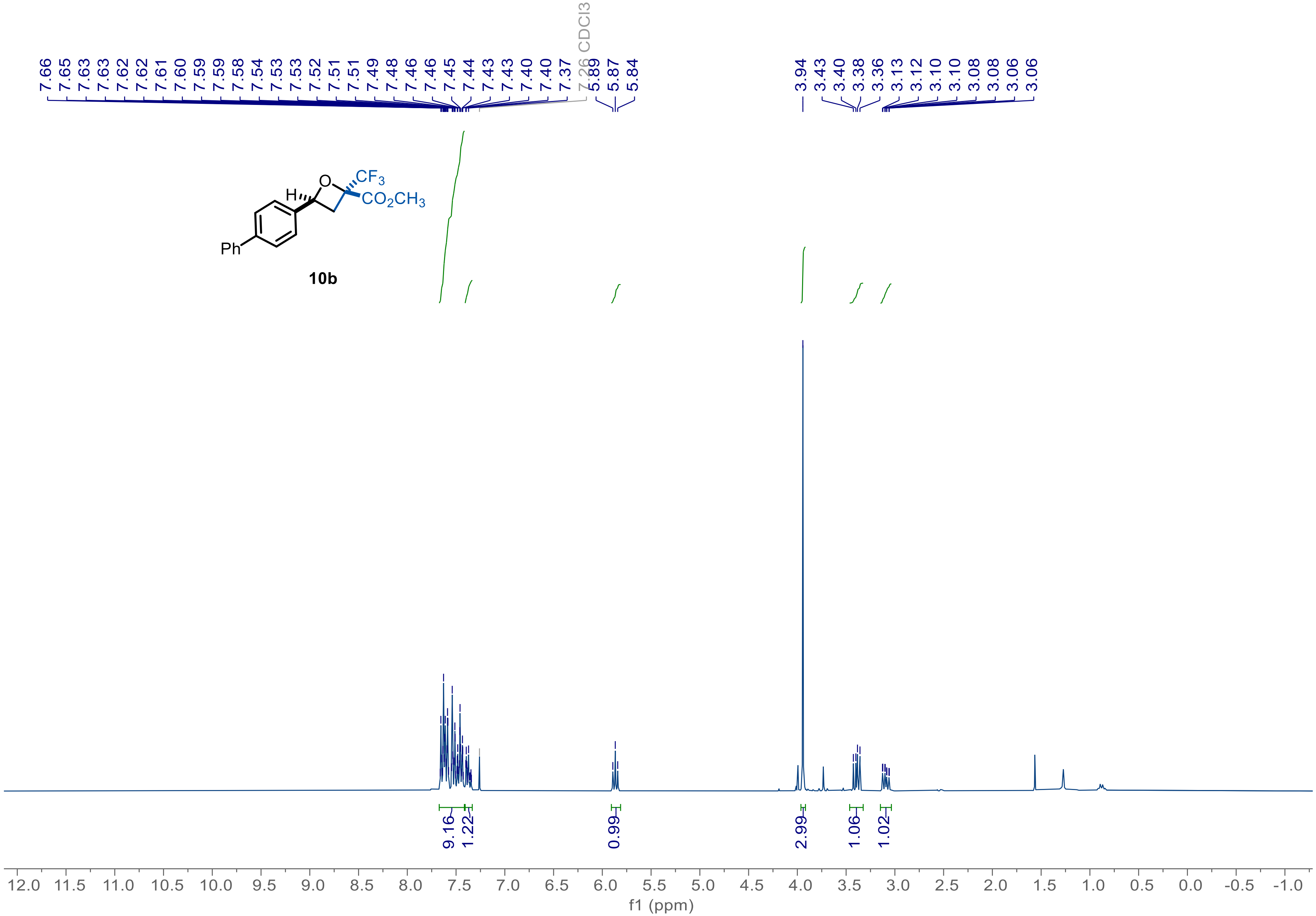

^{13}C NMR (101 MHz, $CDCl_3$) of **10b**

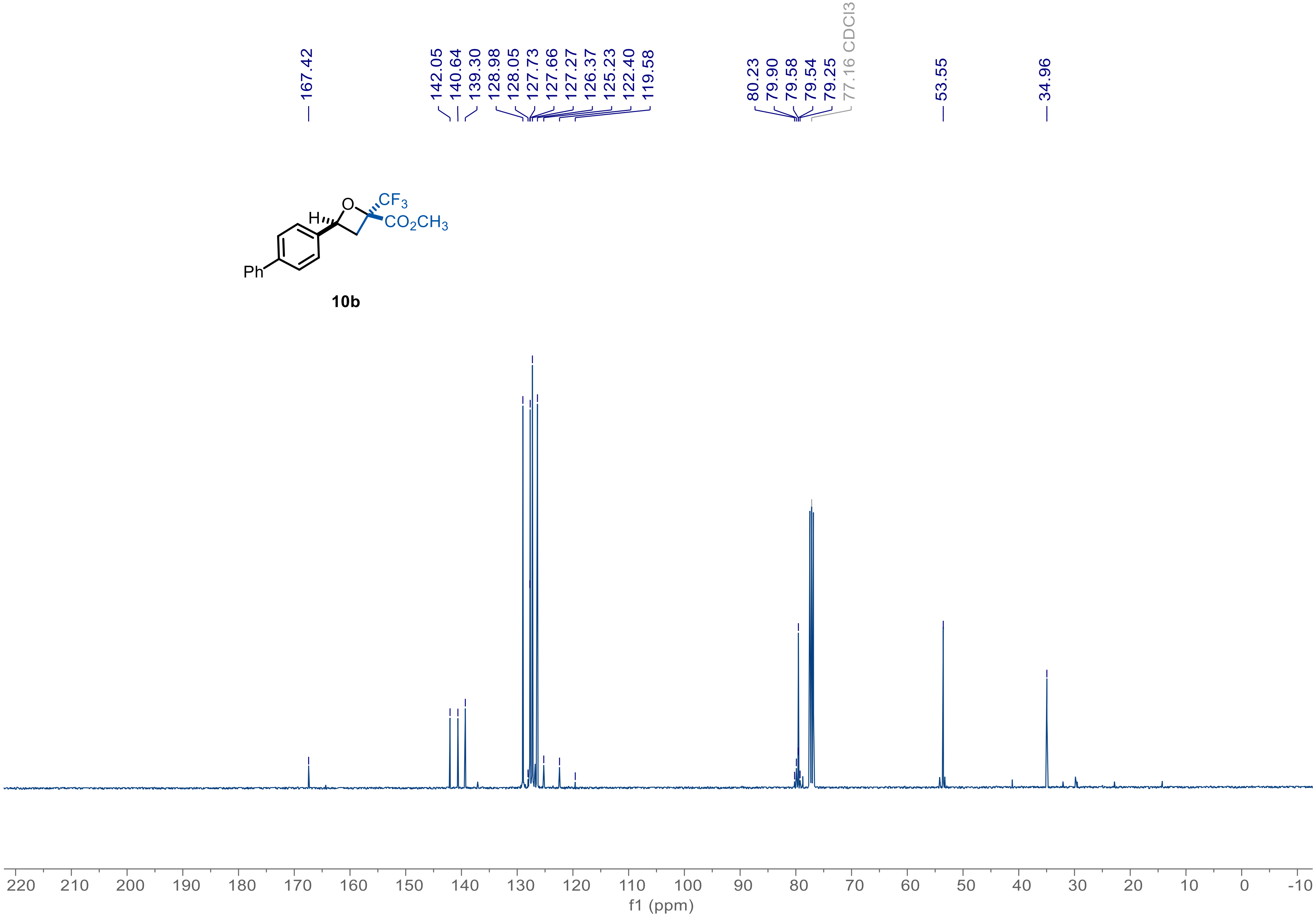

^{19}F NMR (282 MHz, $CDCl_3$) of **10b**

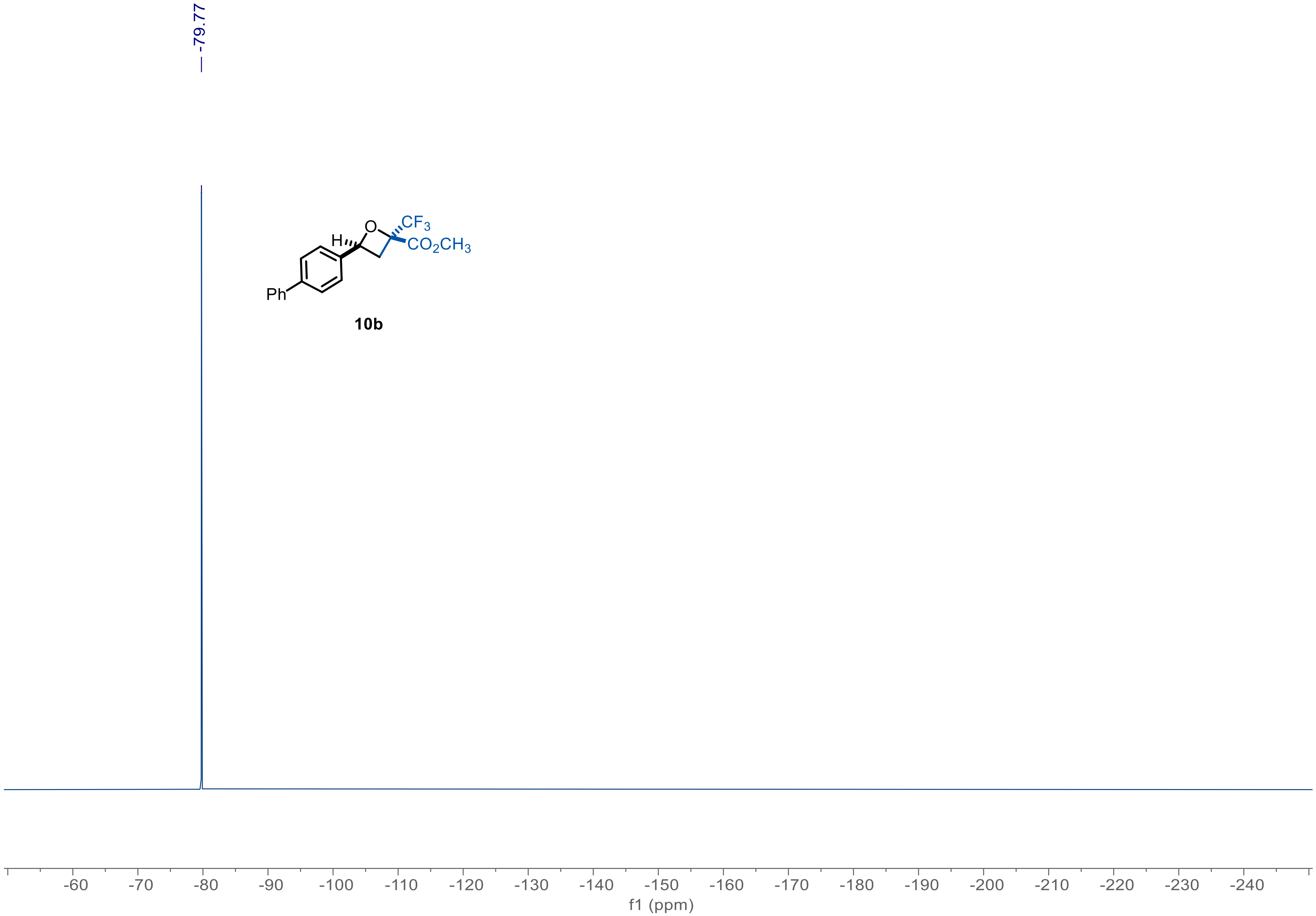

^{1}H NMR (300 MHz, $CDCl_3$) of **10b'**

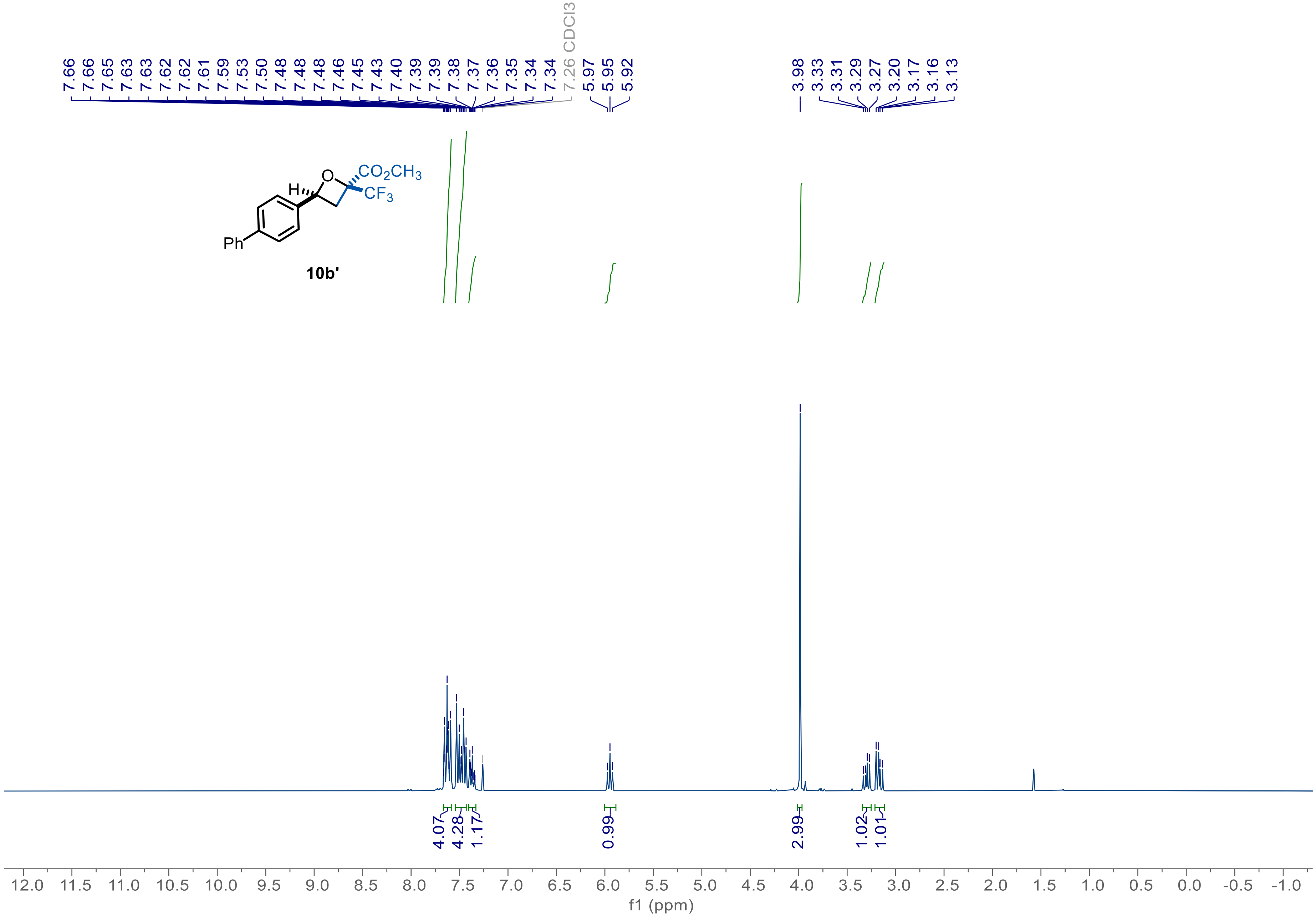

^{13}C NMR (75 MHz, $CDCl_3$) of **10b'**

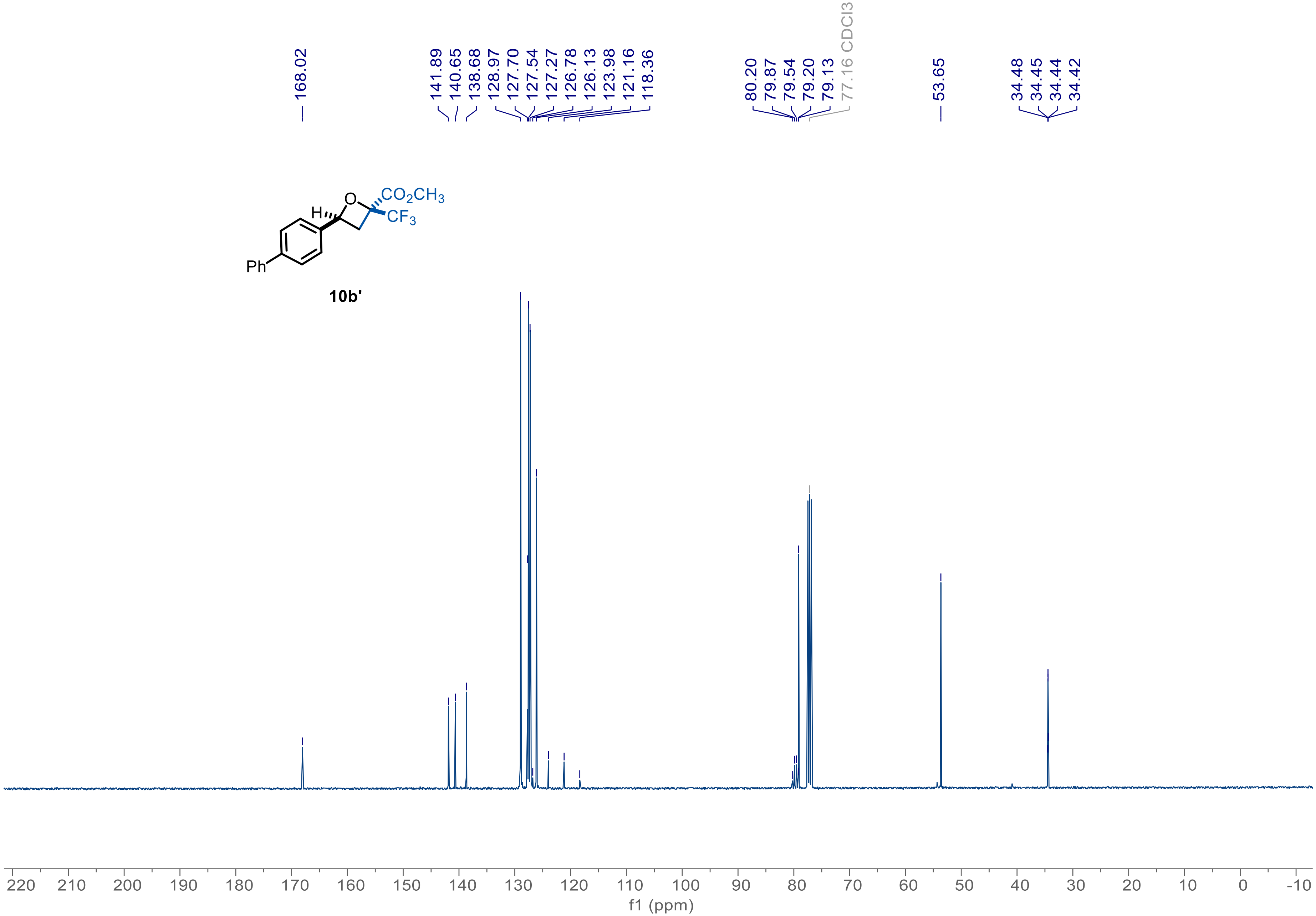

^{19}F NMR (282 MHz, $CDCl_3$) of **10b'**

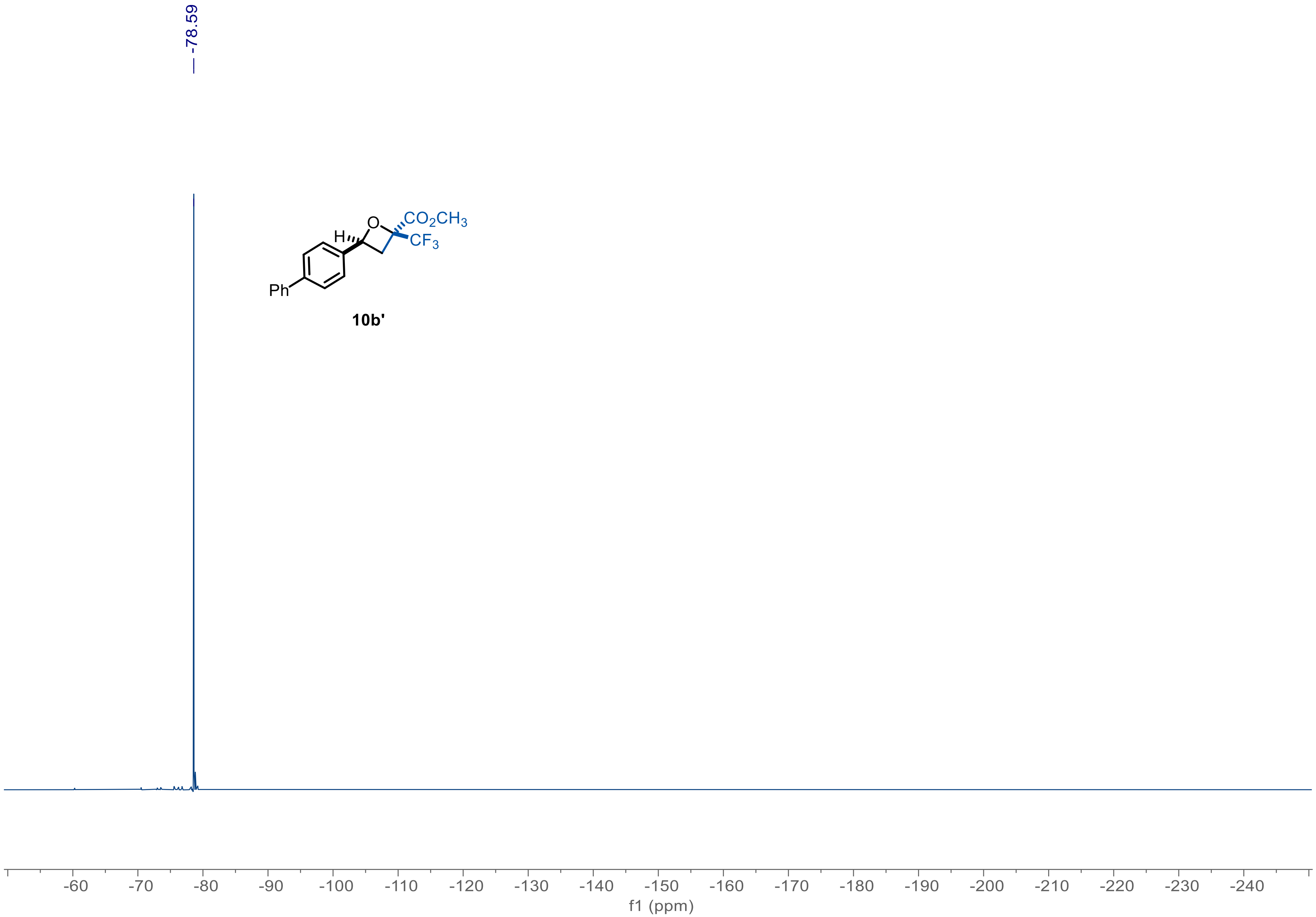

^{1}H NMR (300 MHz, CDCl3) of **11a**

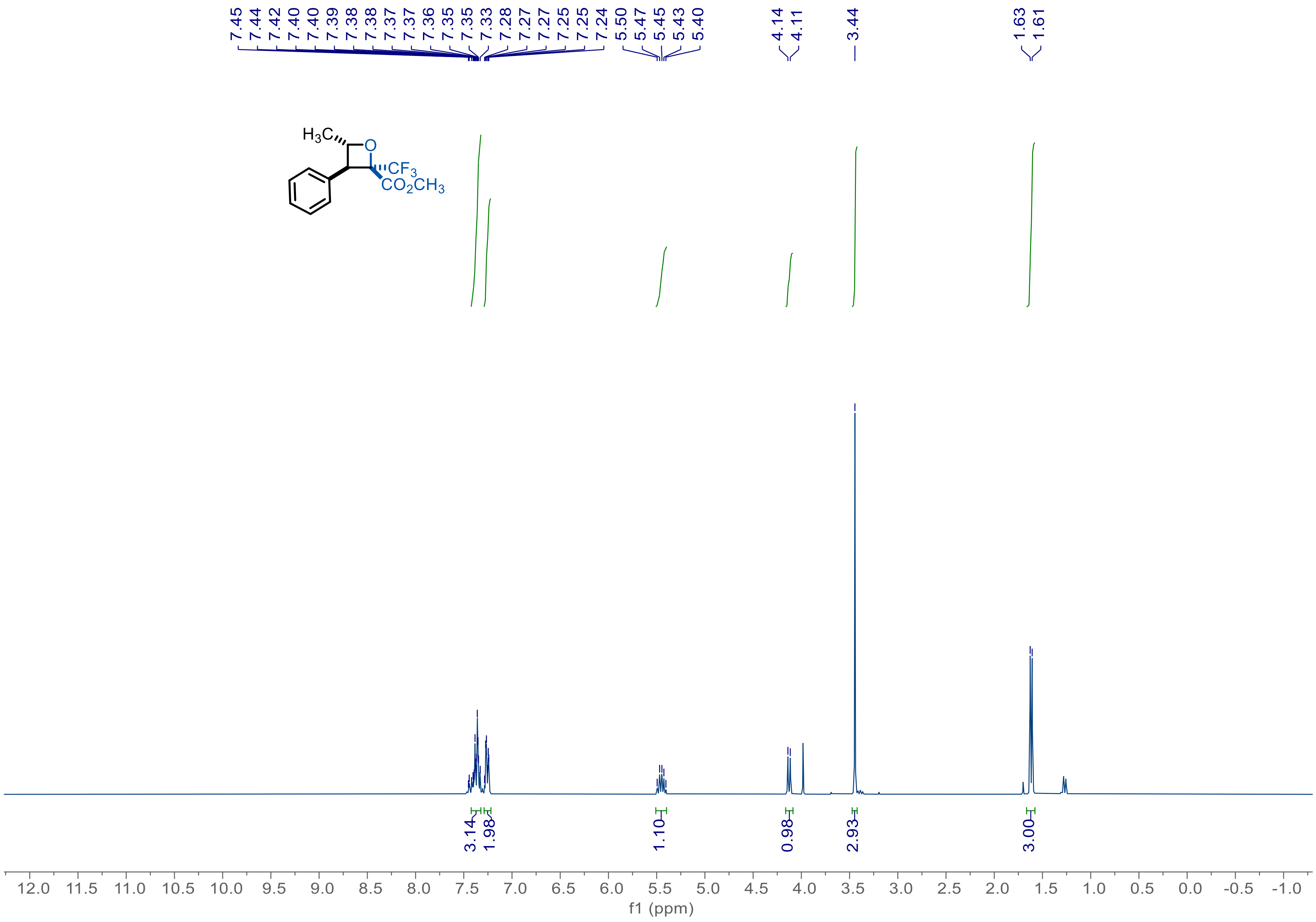

^{13}C NMR (75 MHz, CDCl$_3$) of **11a**

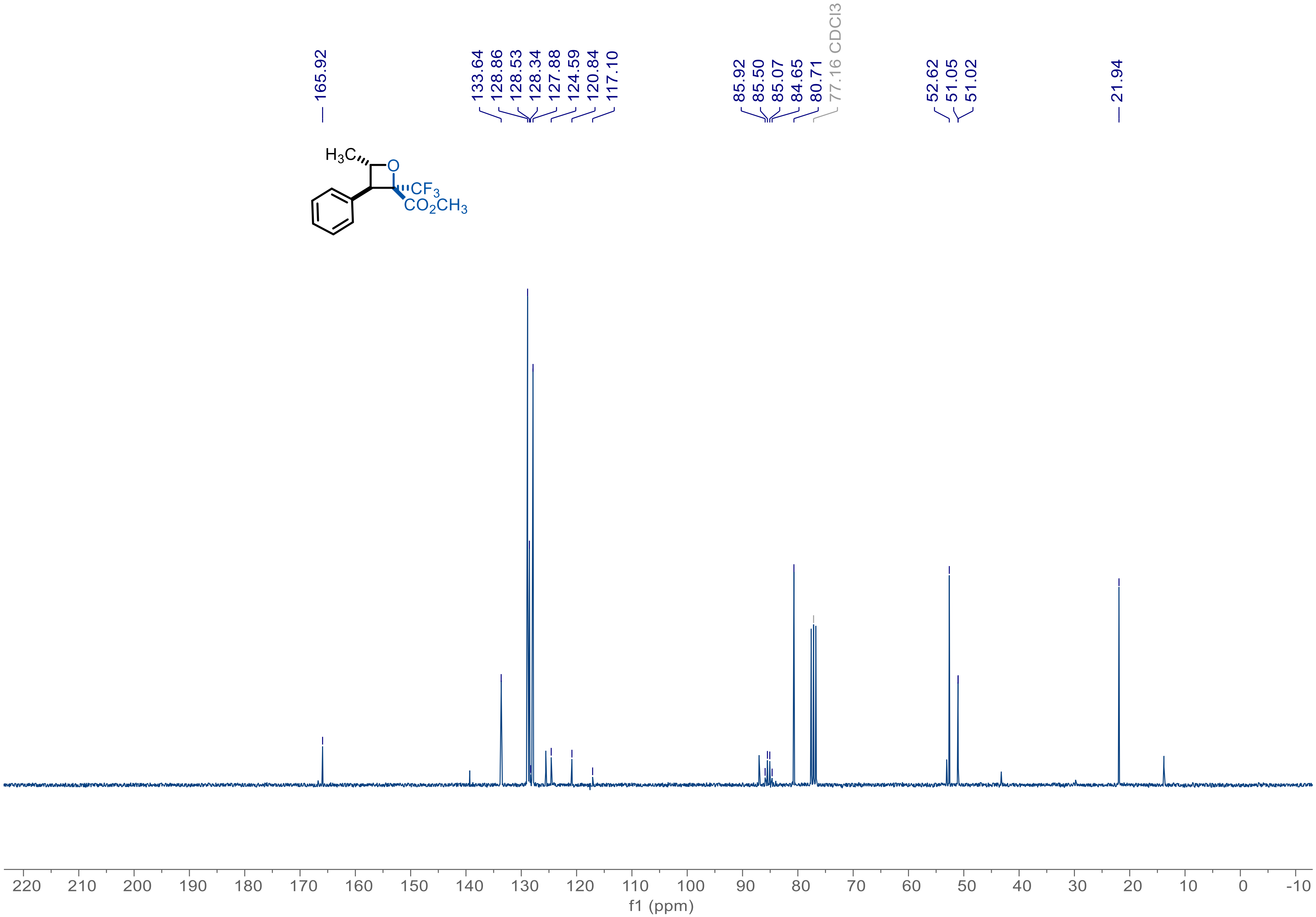

^{19}F NMR (282 MHz, $CDCl_3$) of **11a**

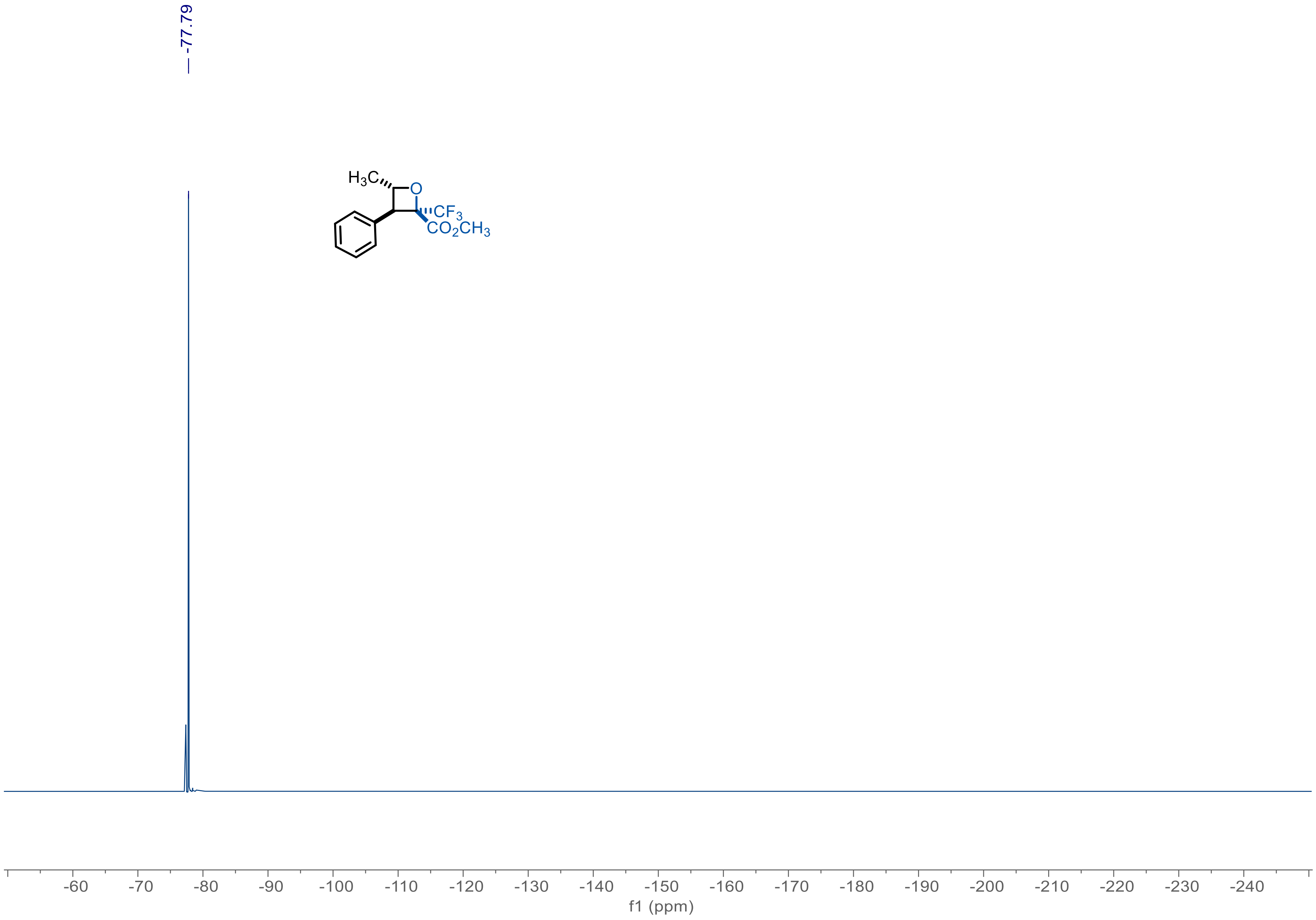

^{1}H NMR (300 MHz, $CDCl_3$) of **12a**

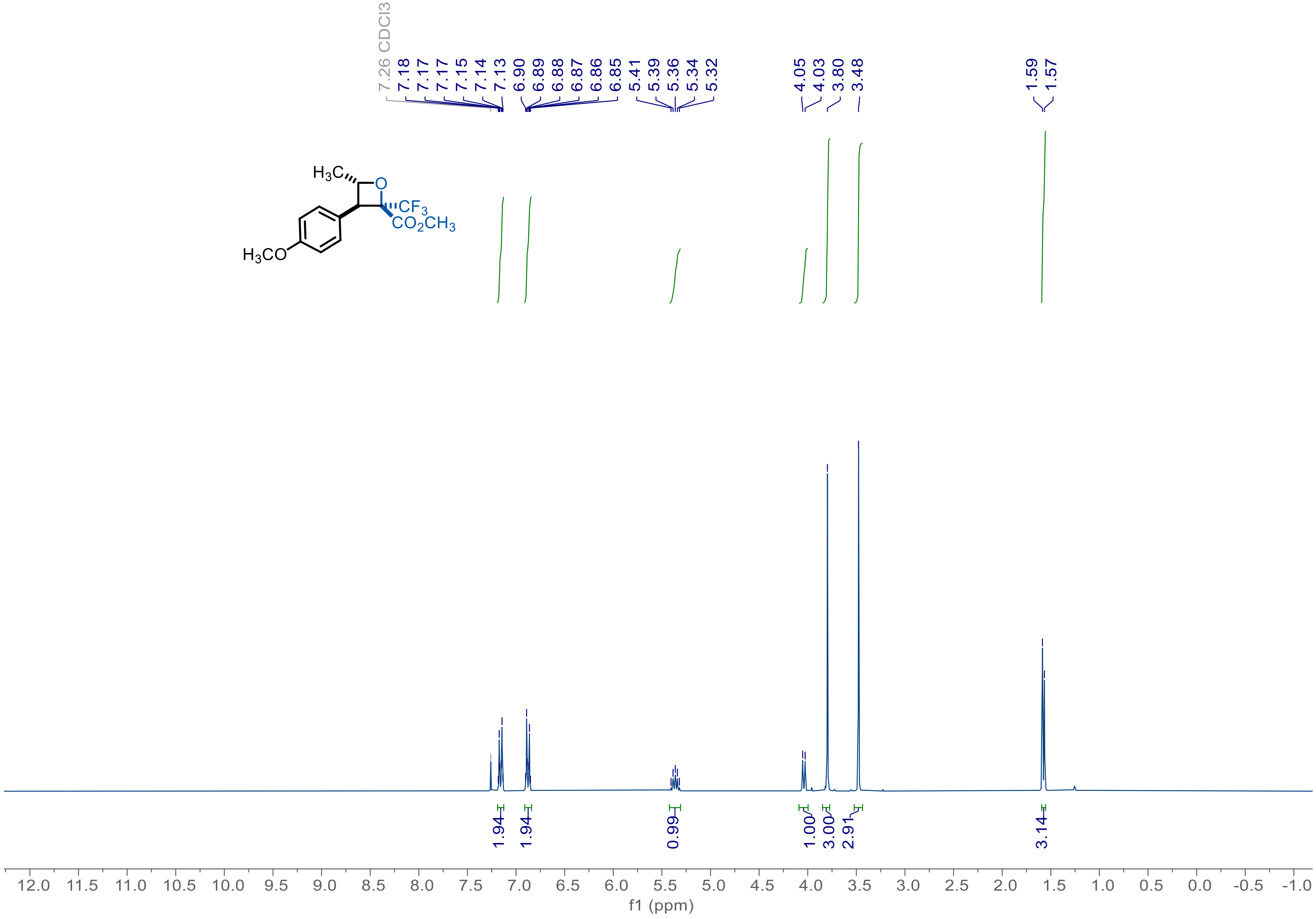

^{13}C NMR (75 MHz, $CDCl_3$) of **12a**

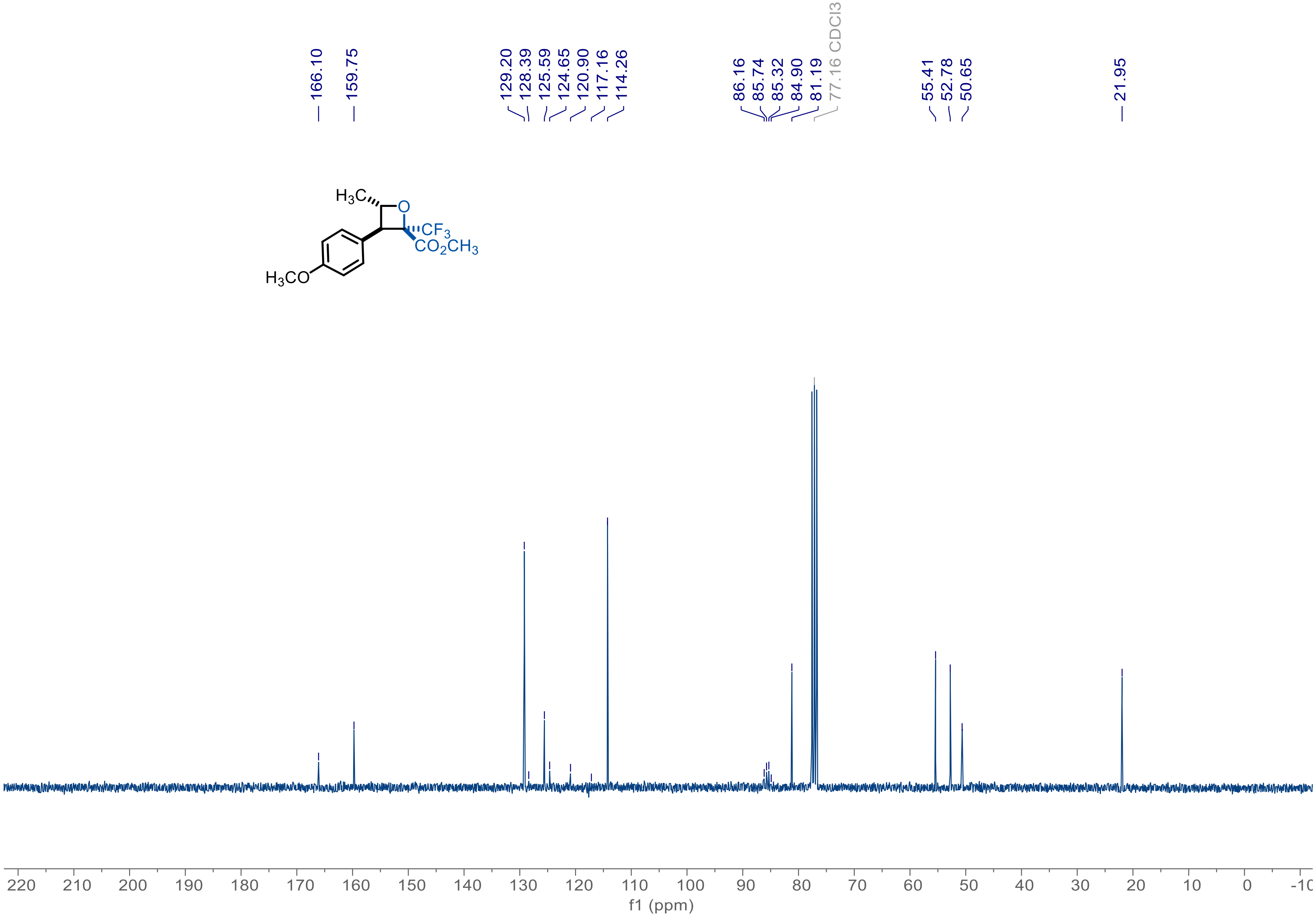

^{19}F NMR (282 MHz, $CDCl_3$) of **12a**

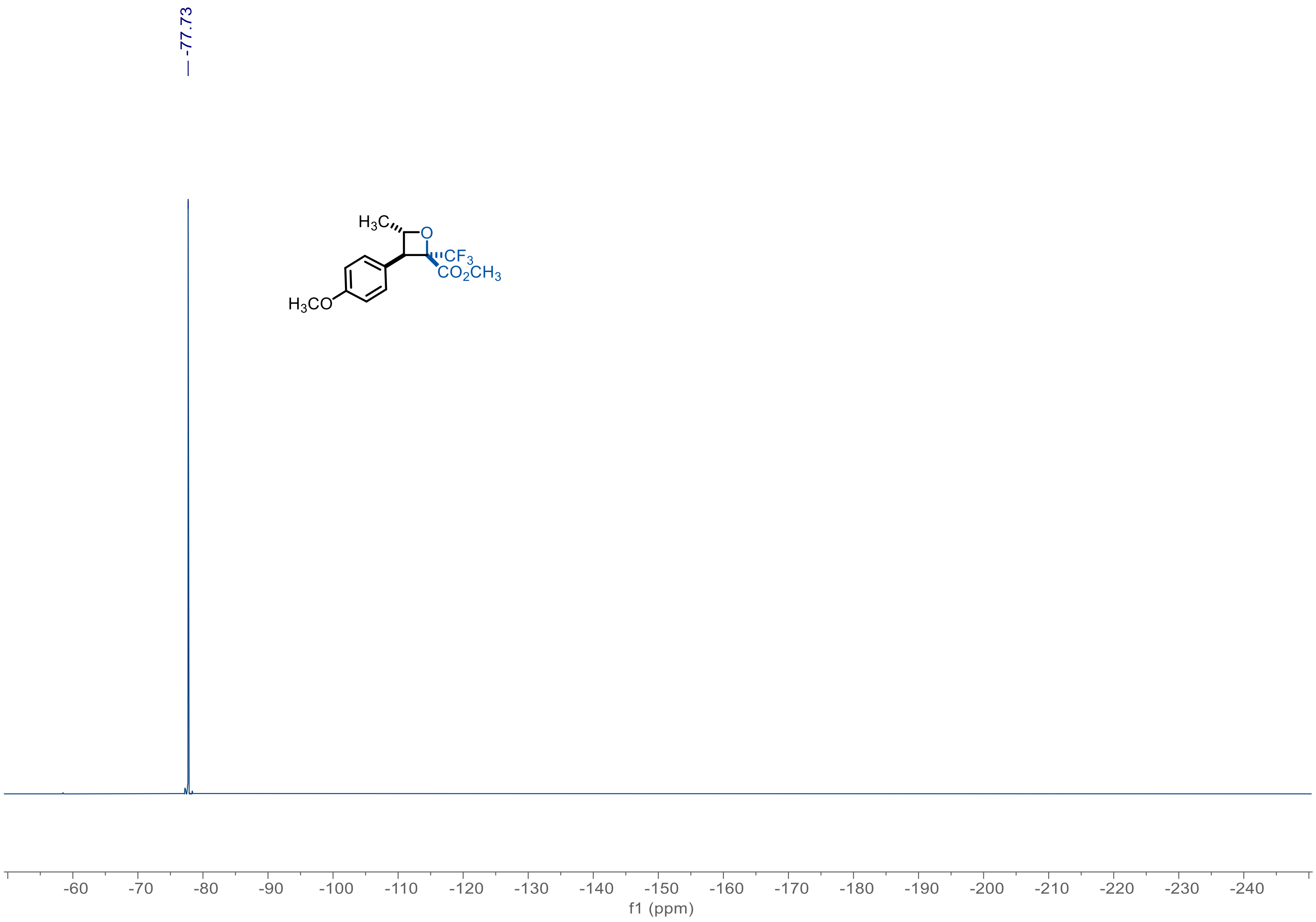

^{1}H NMR (300 MHz, $CDCl_3$) of **13a**

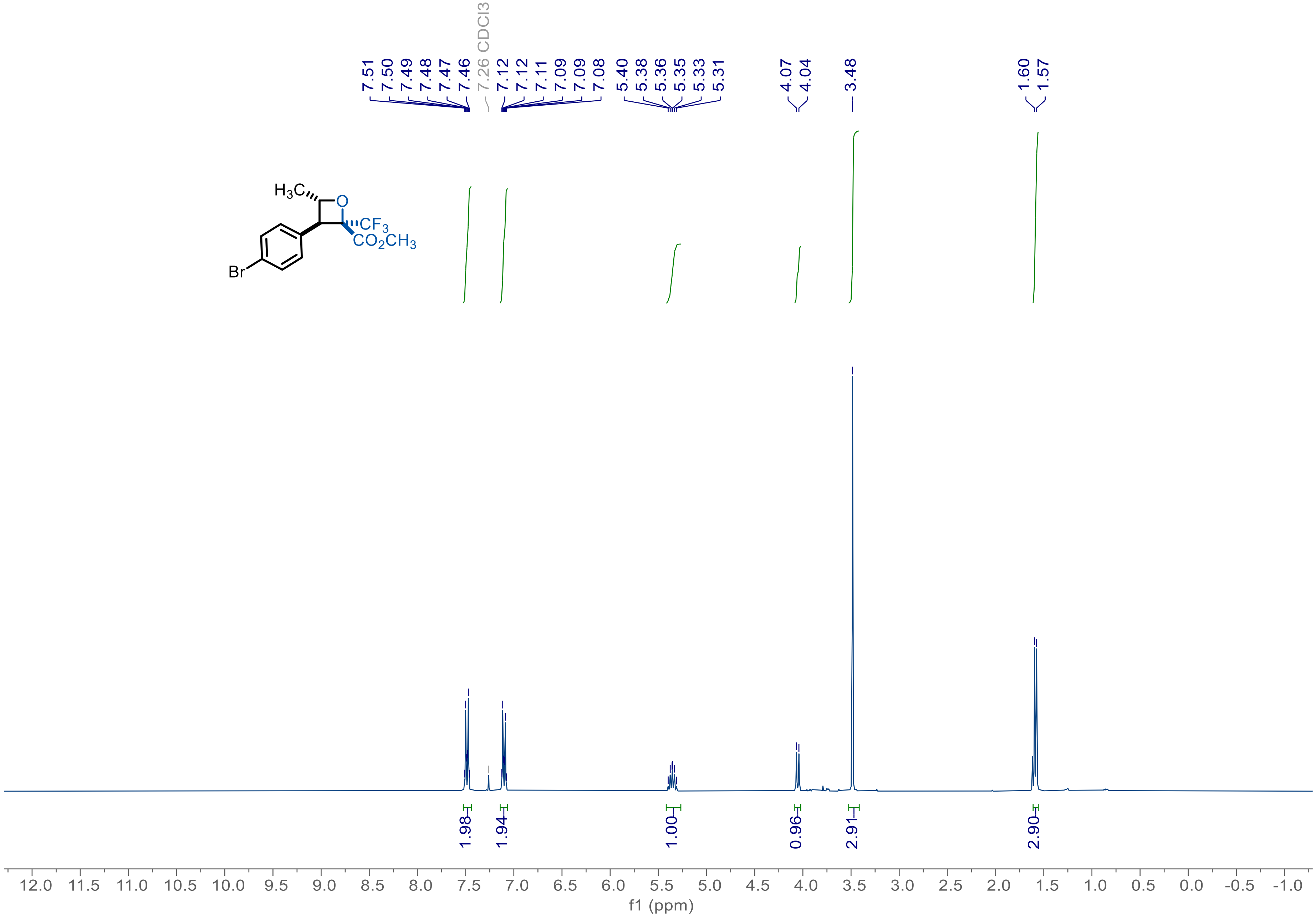

^{13}C NMR (75 MHz, $CDCl_3$) of **13a**

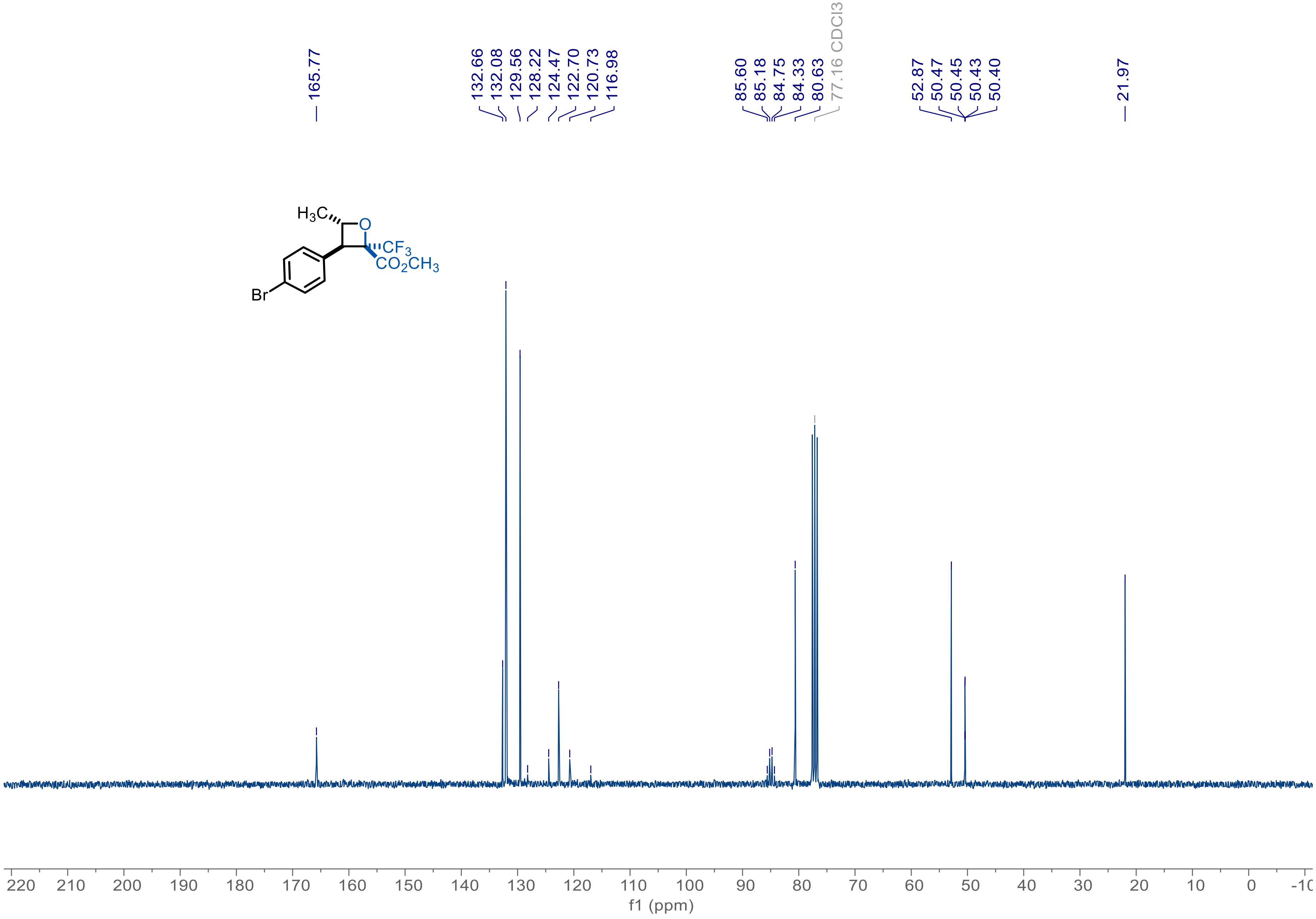

^{19}F NMR (282 MHz, $CDCl_3$) of **13a**

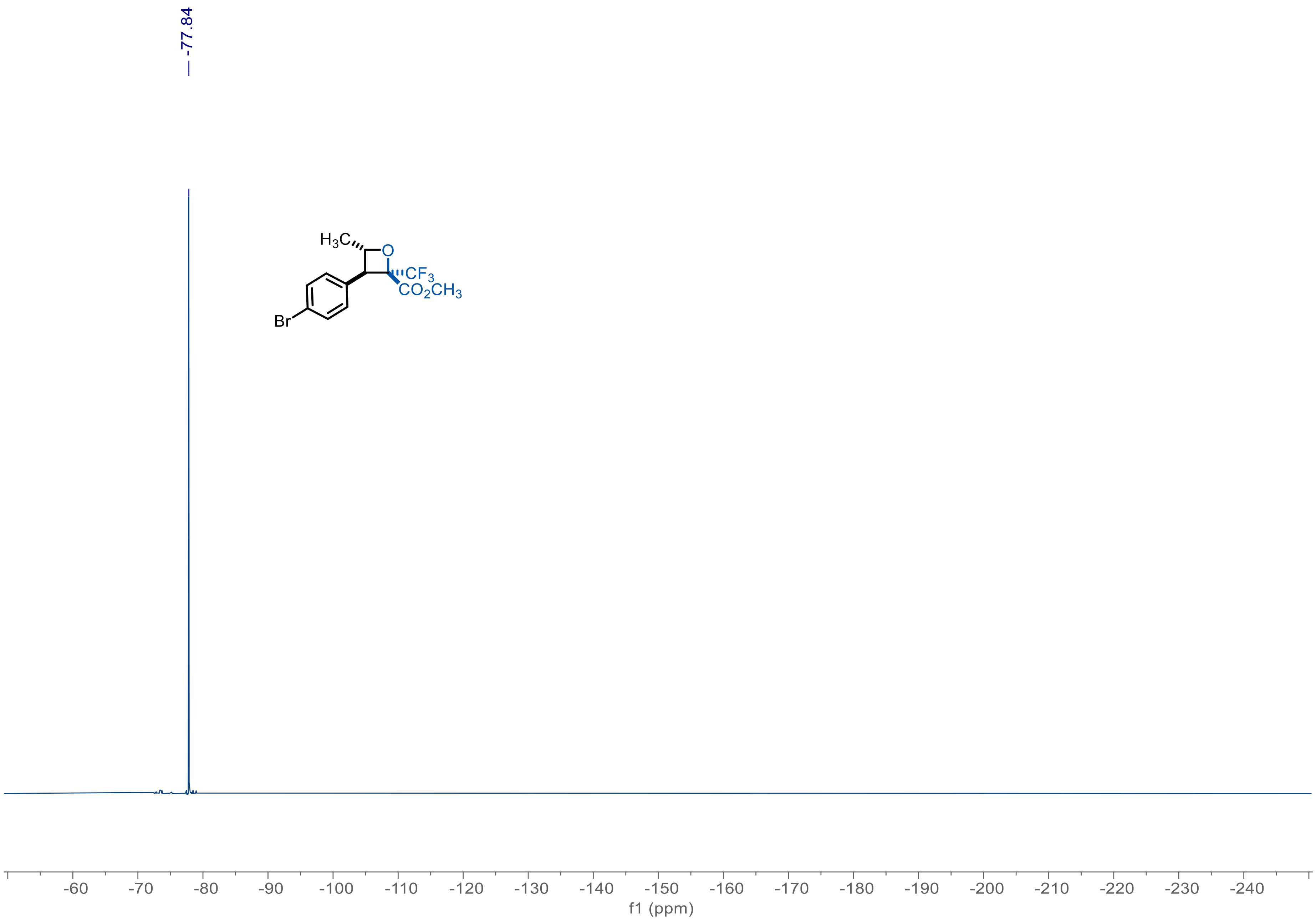

^{1}H NMR (300 MHz, $CDCl_3$) of **14a**

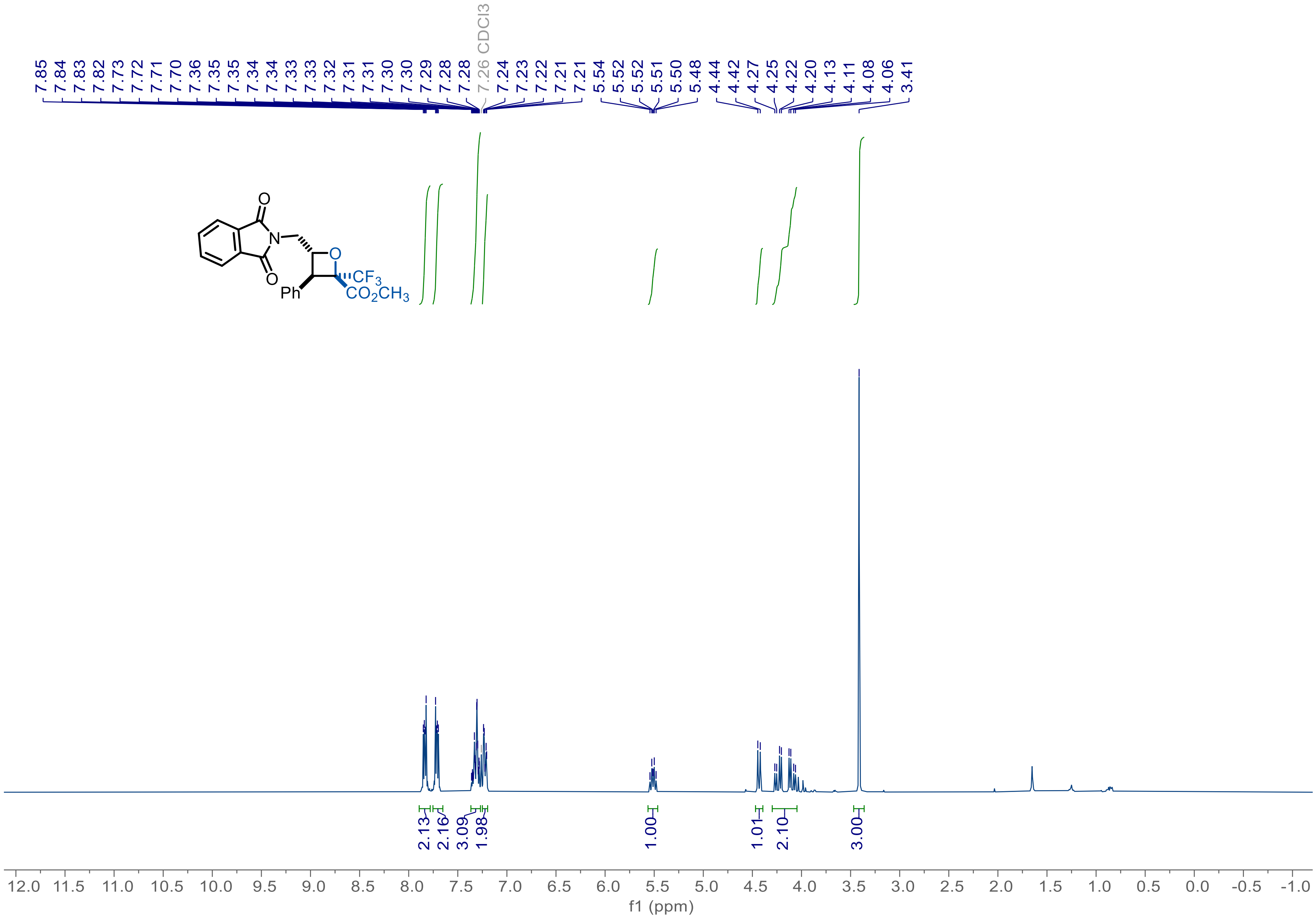

^{13}C NMR (75 MHz, $CDCl_3$) of **14a**

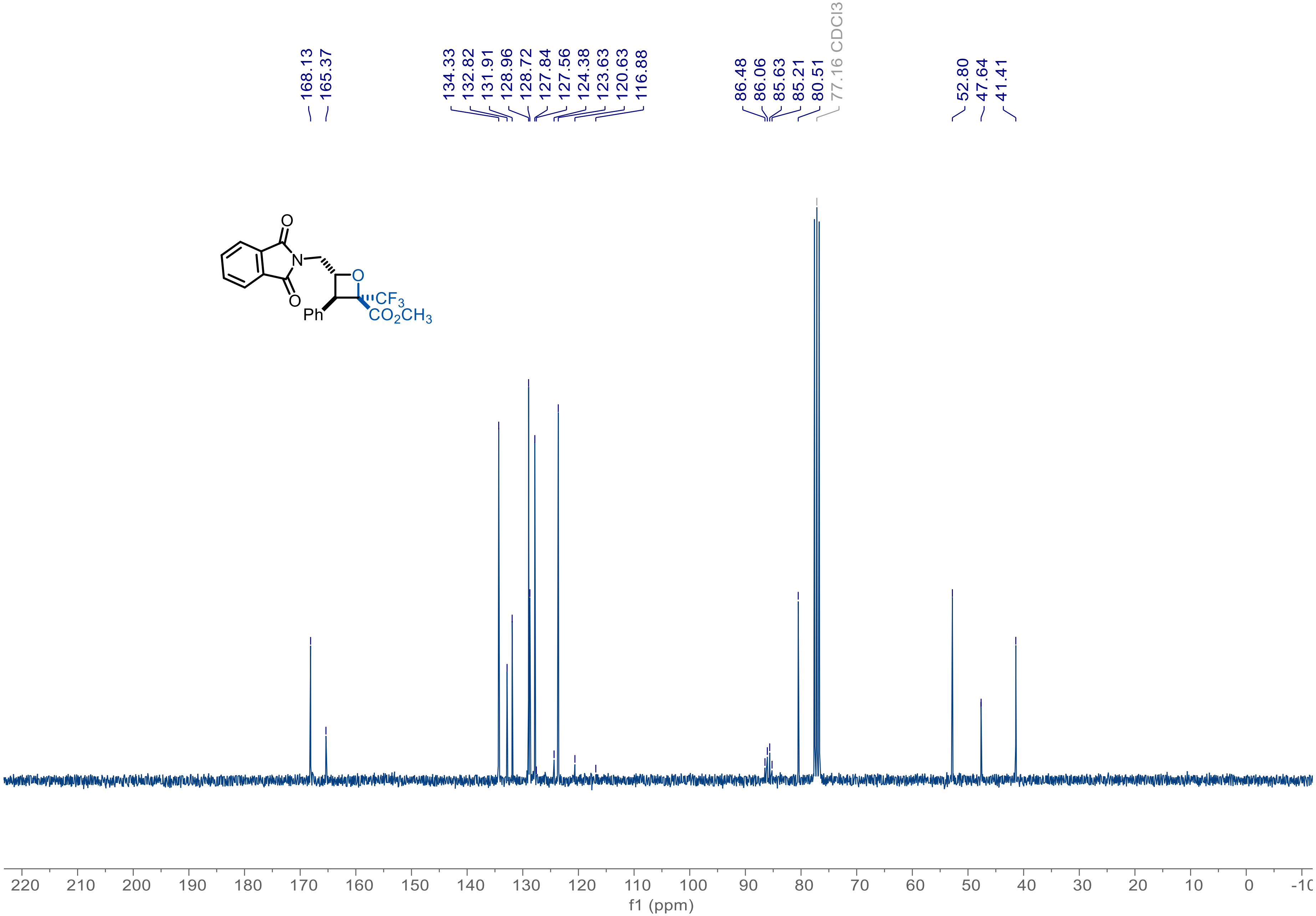

^{19}F NMR (282 MHz, $CDCl_3$) of **14a**

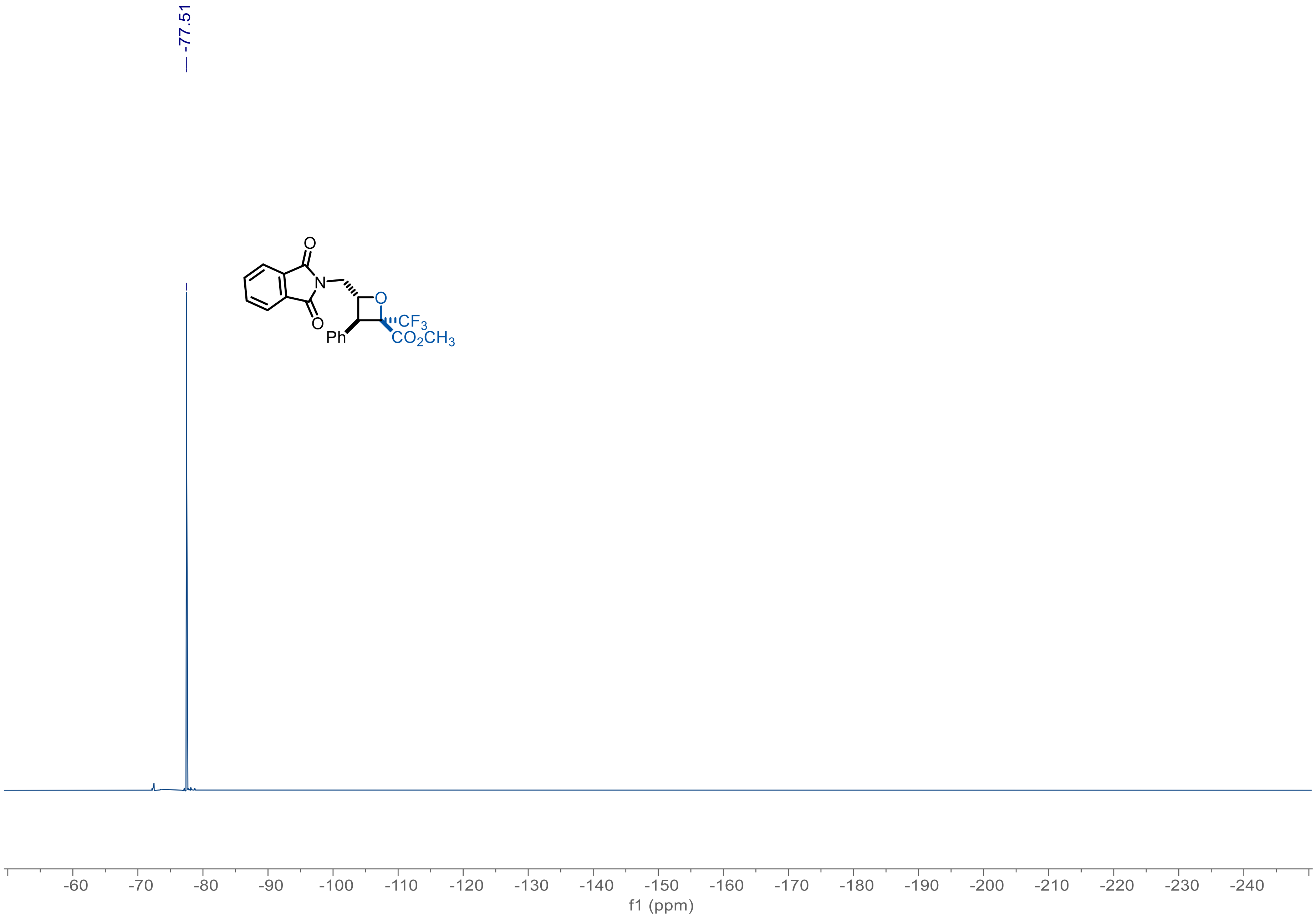

^{1}H NMR (300 MHz, $CDCl_3$) of **15a**

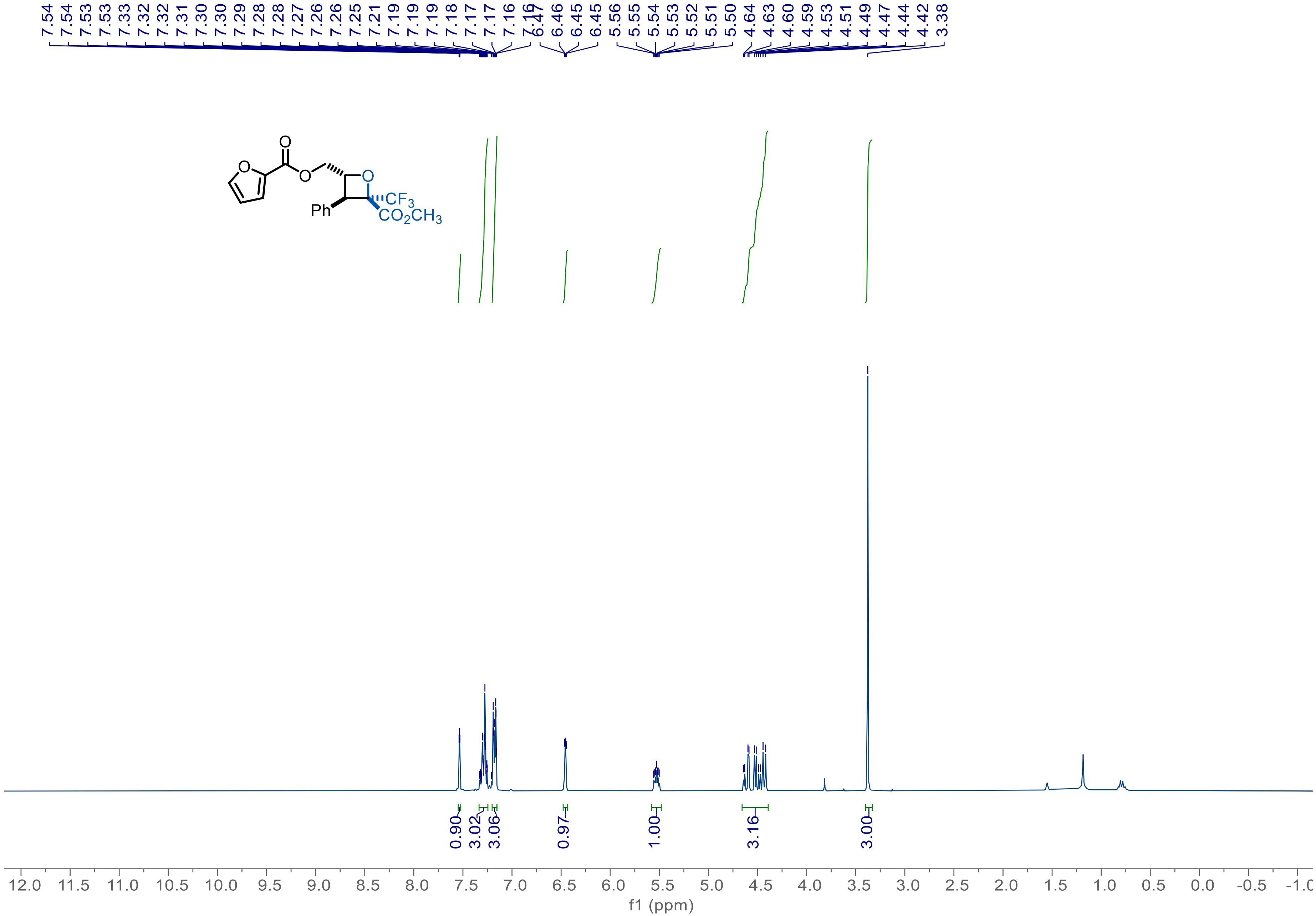

^{13}C NMR (75 MHz, $CDCl_3$) of **15a**

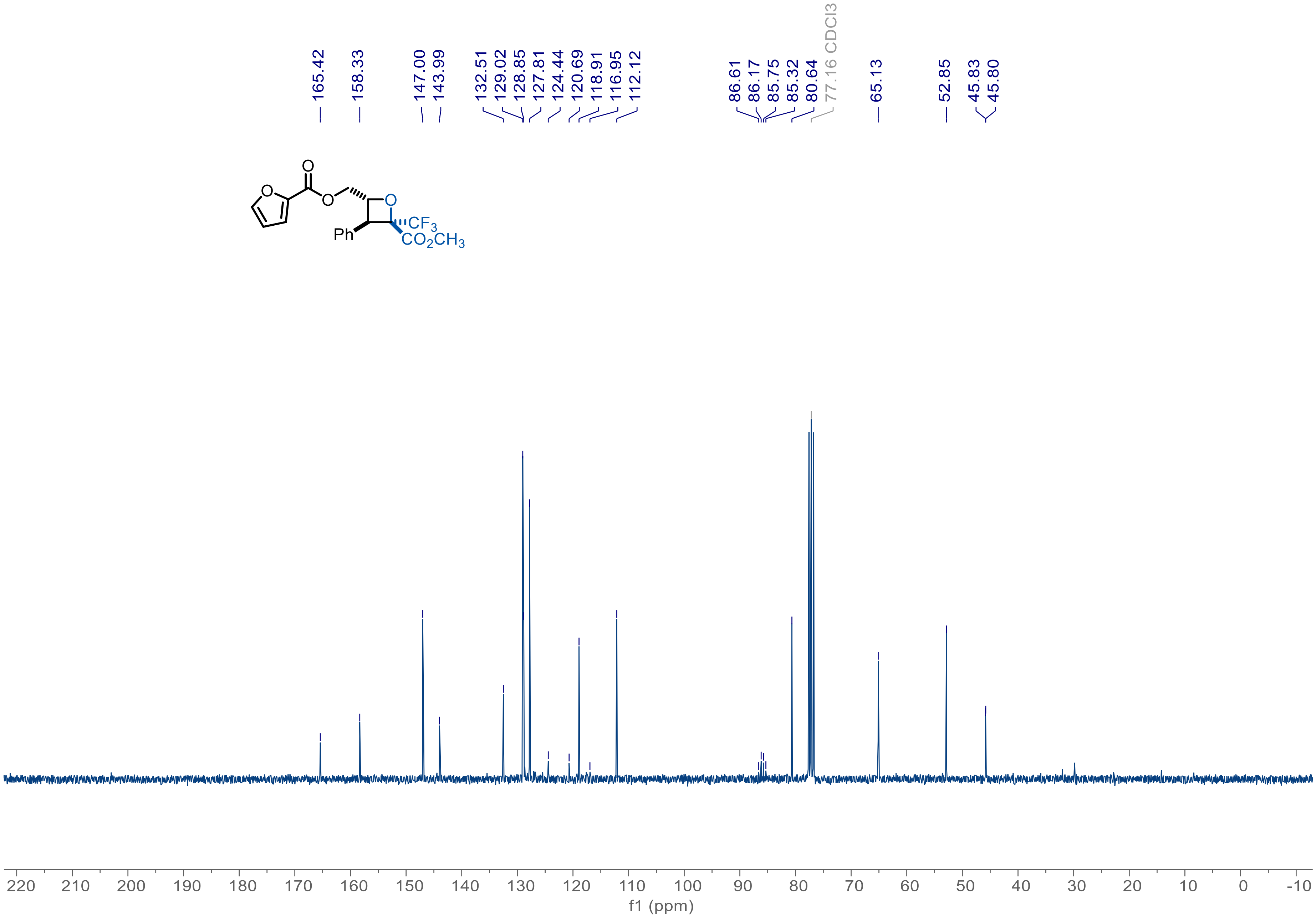

^{19}F NMR (282 MHz, $CDCl_3$) of **15a**

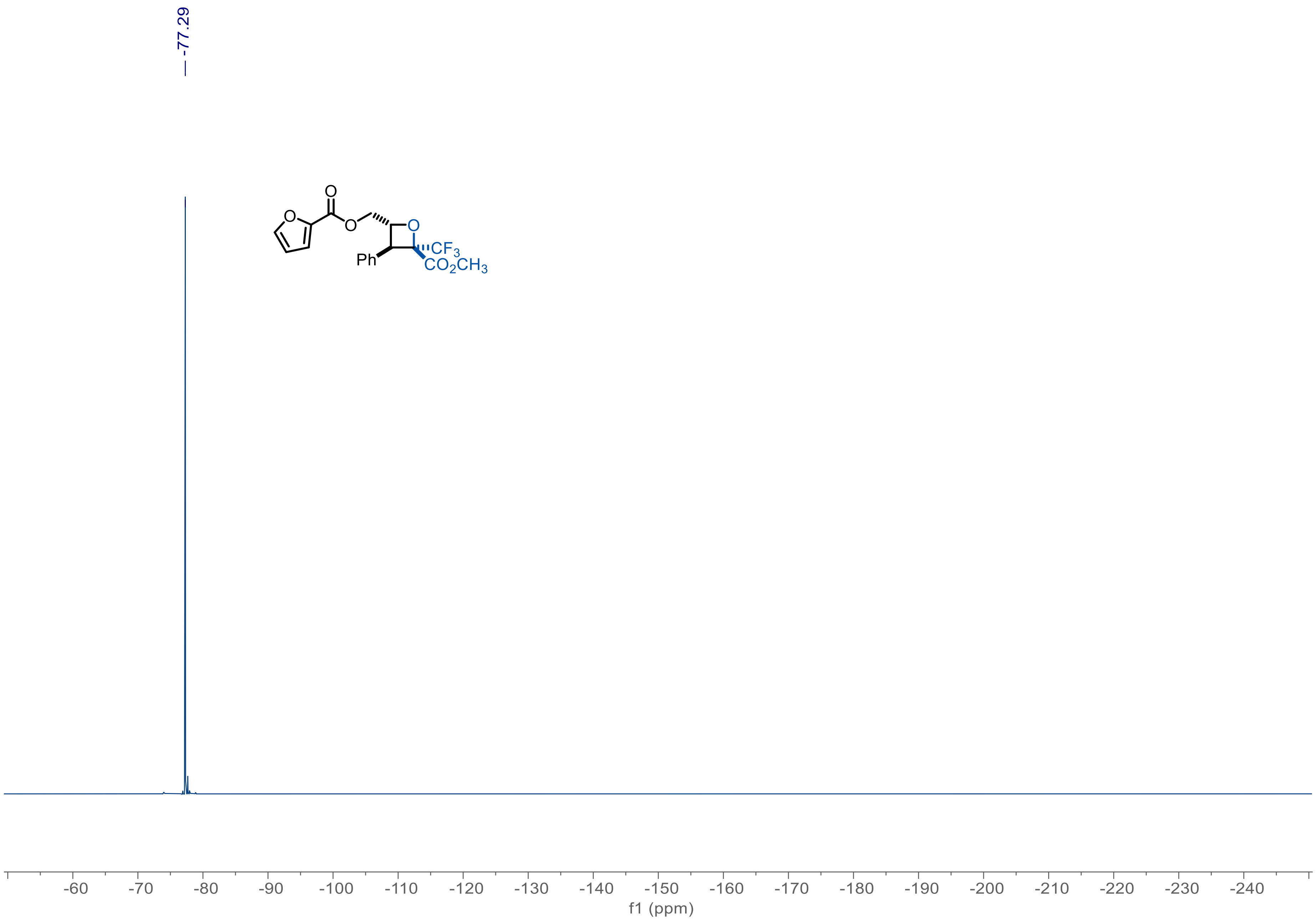

^{1}H NMR (300 MHz, $CDCl_3$) of **16a**

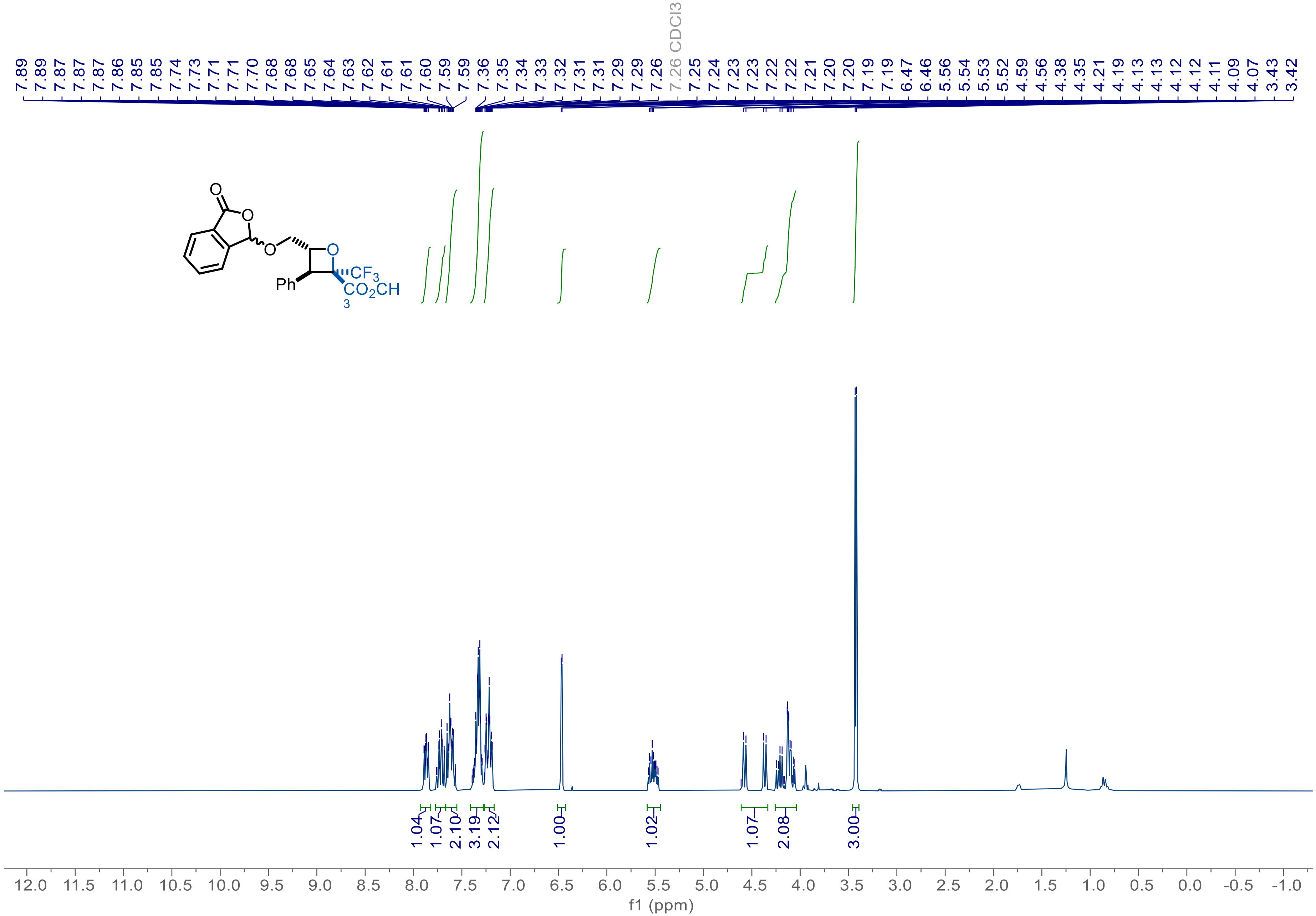

^{13}C NMR (75 MHz, $CDCl_3$) of **16a**

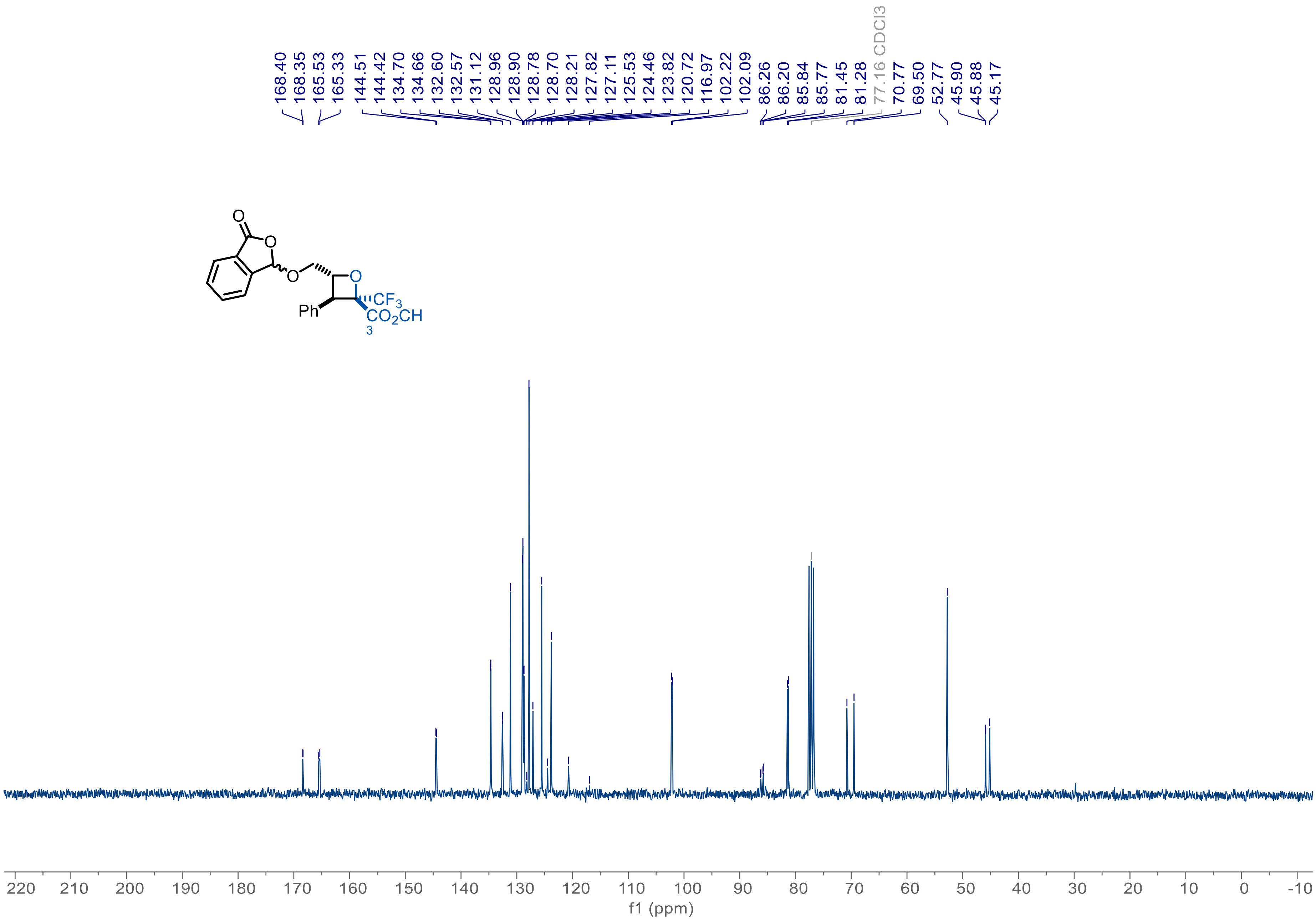

^{19}F NMR (282 MHz, $CDCl_3$) of **16a**

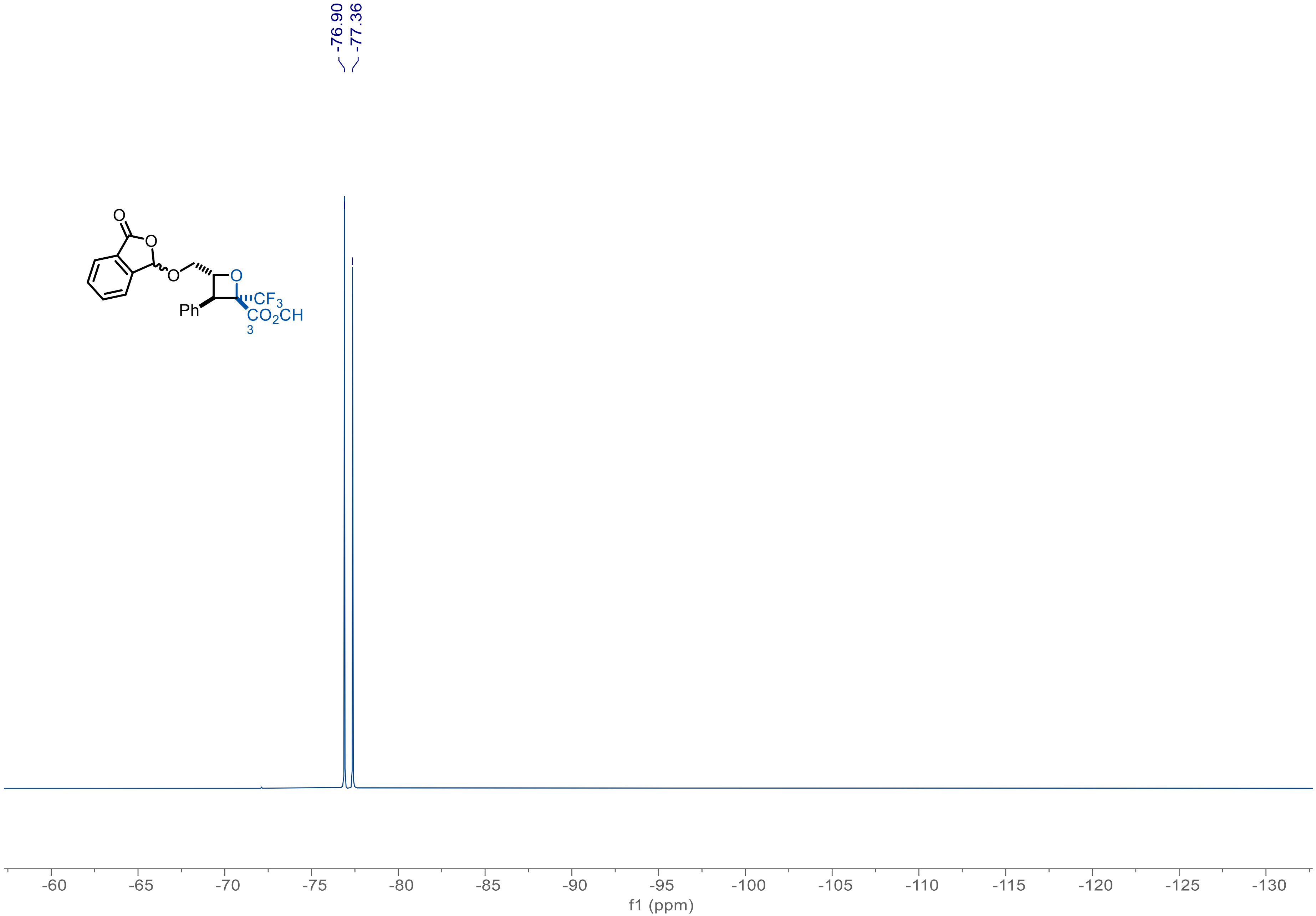

^{1}H NMR (300 MHz, $CDCl_3$) of **17a**

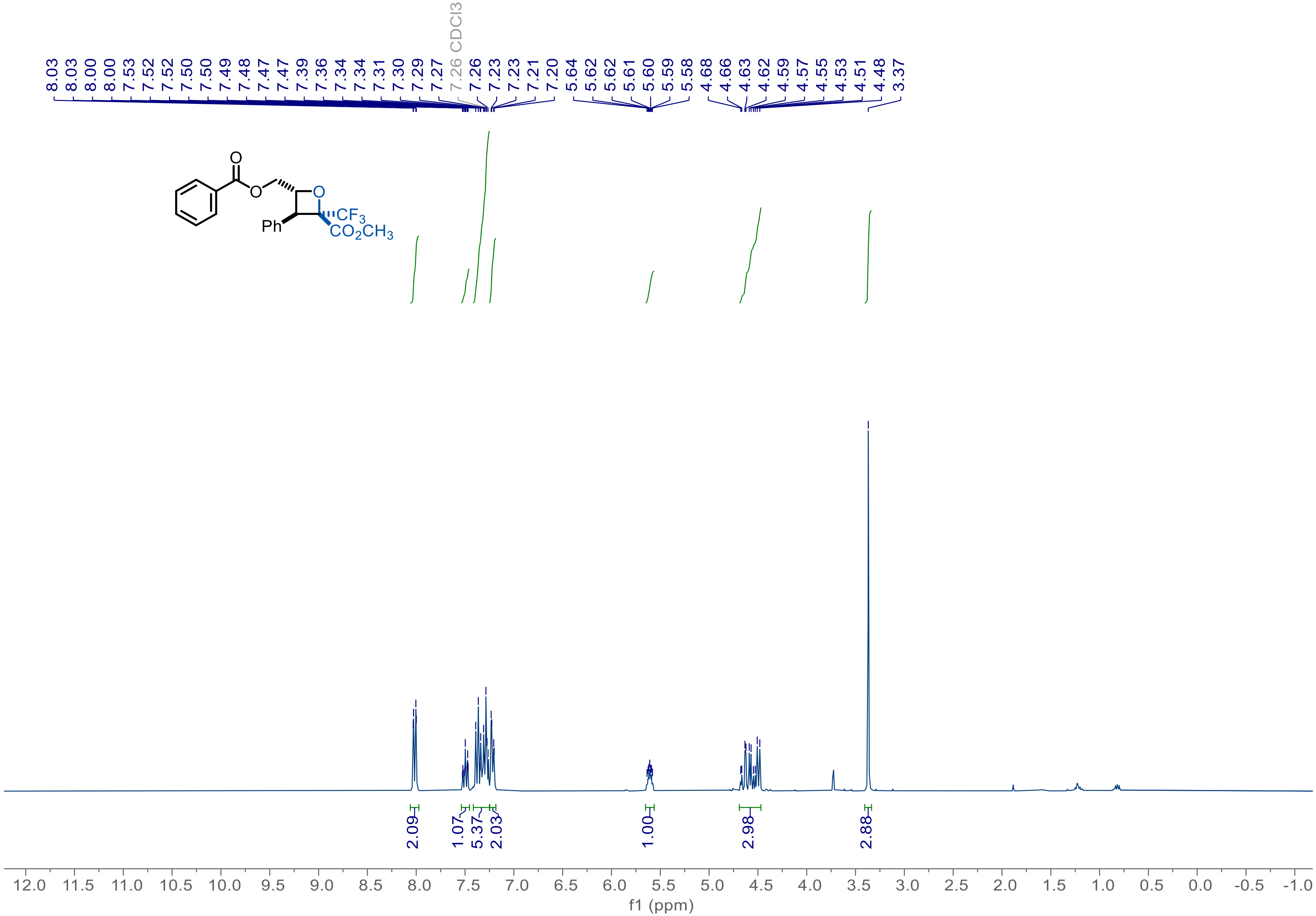

^{13}C NMR (75 MHz, $CDCl_3$) of **17a**

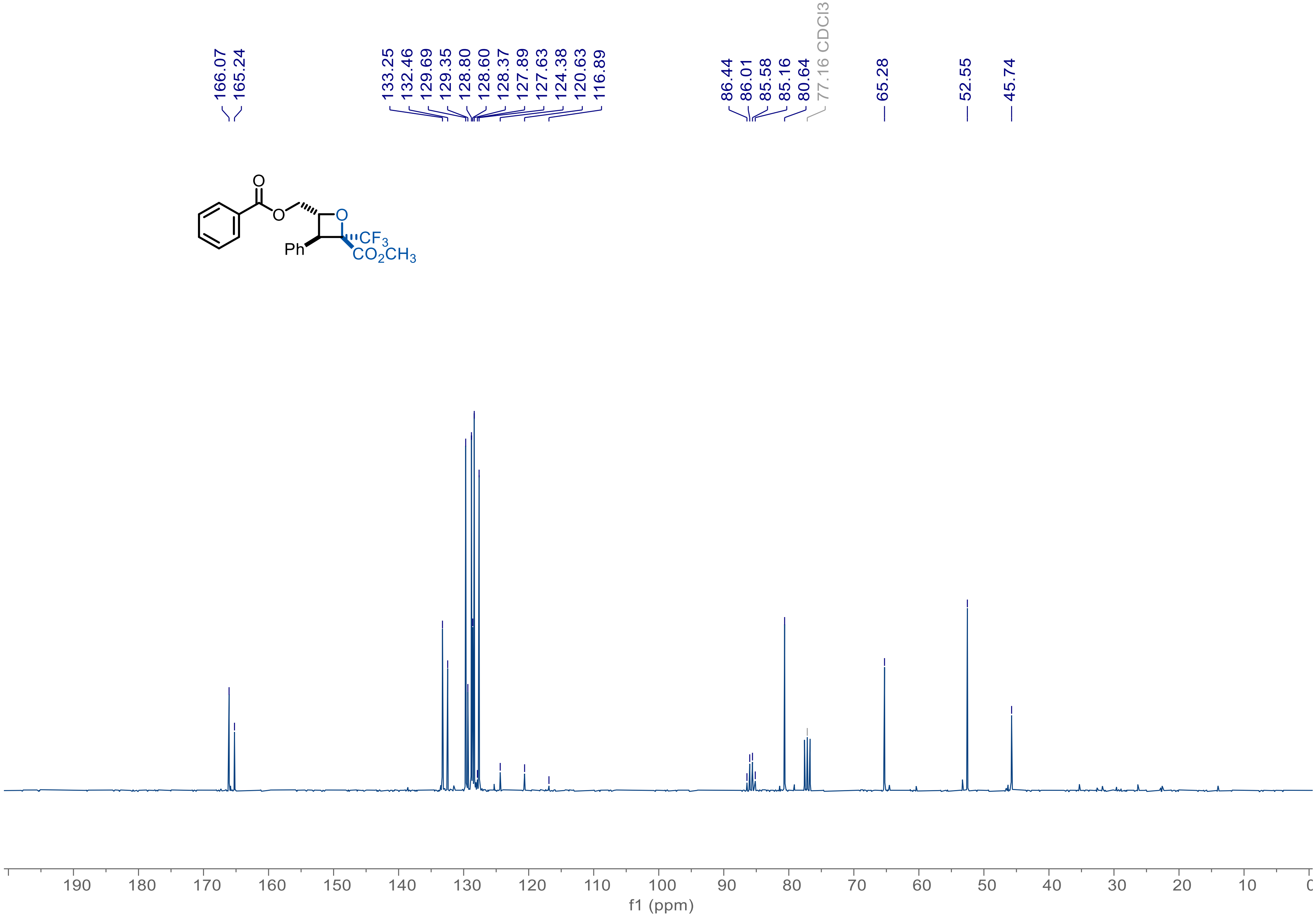

^{19}F NMR (282 MHz, $CDCl_3$) of **17a**

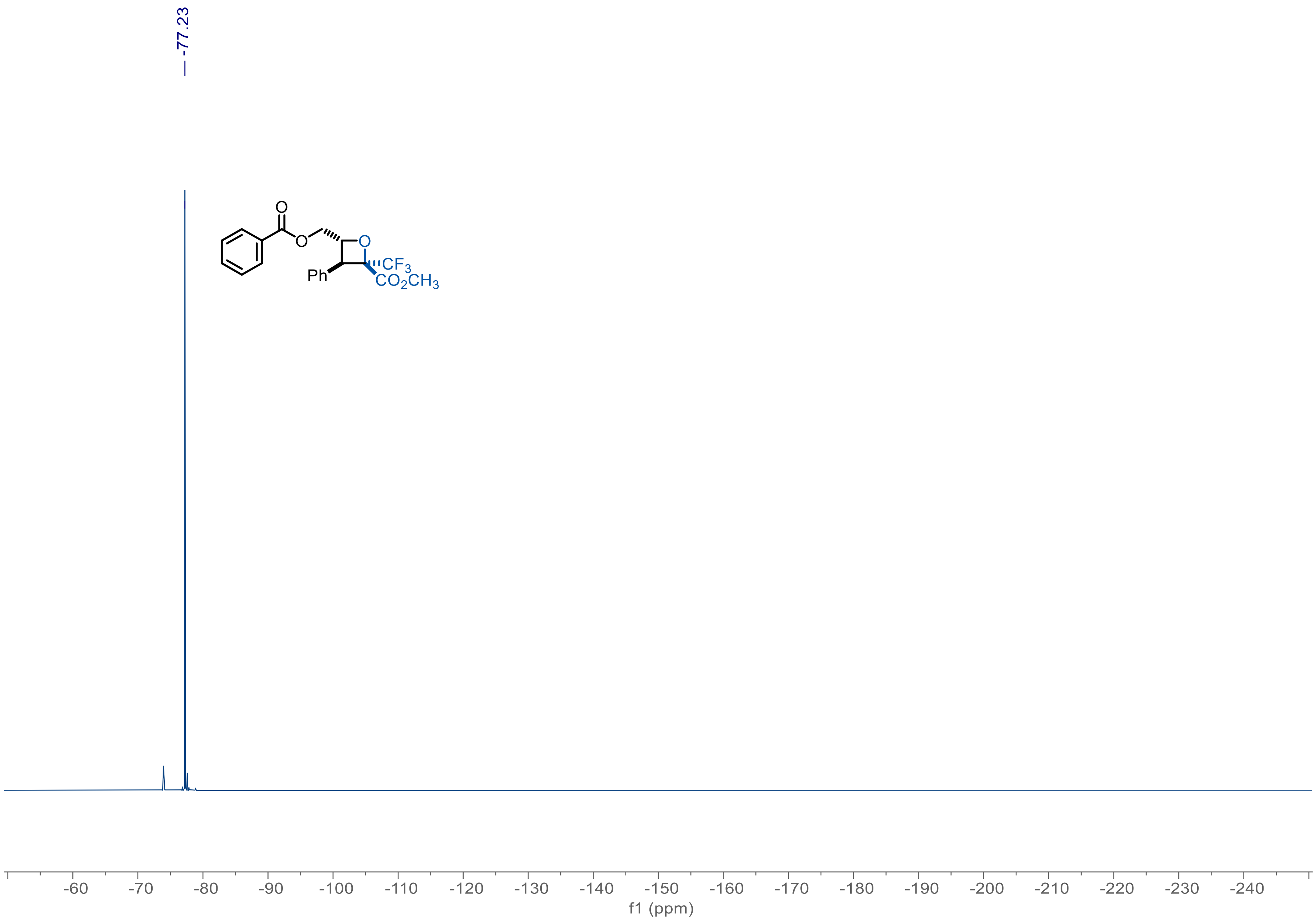

^{1}H NMR (300 MHz, $CDCl_3$) of **18a**

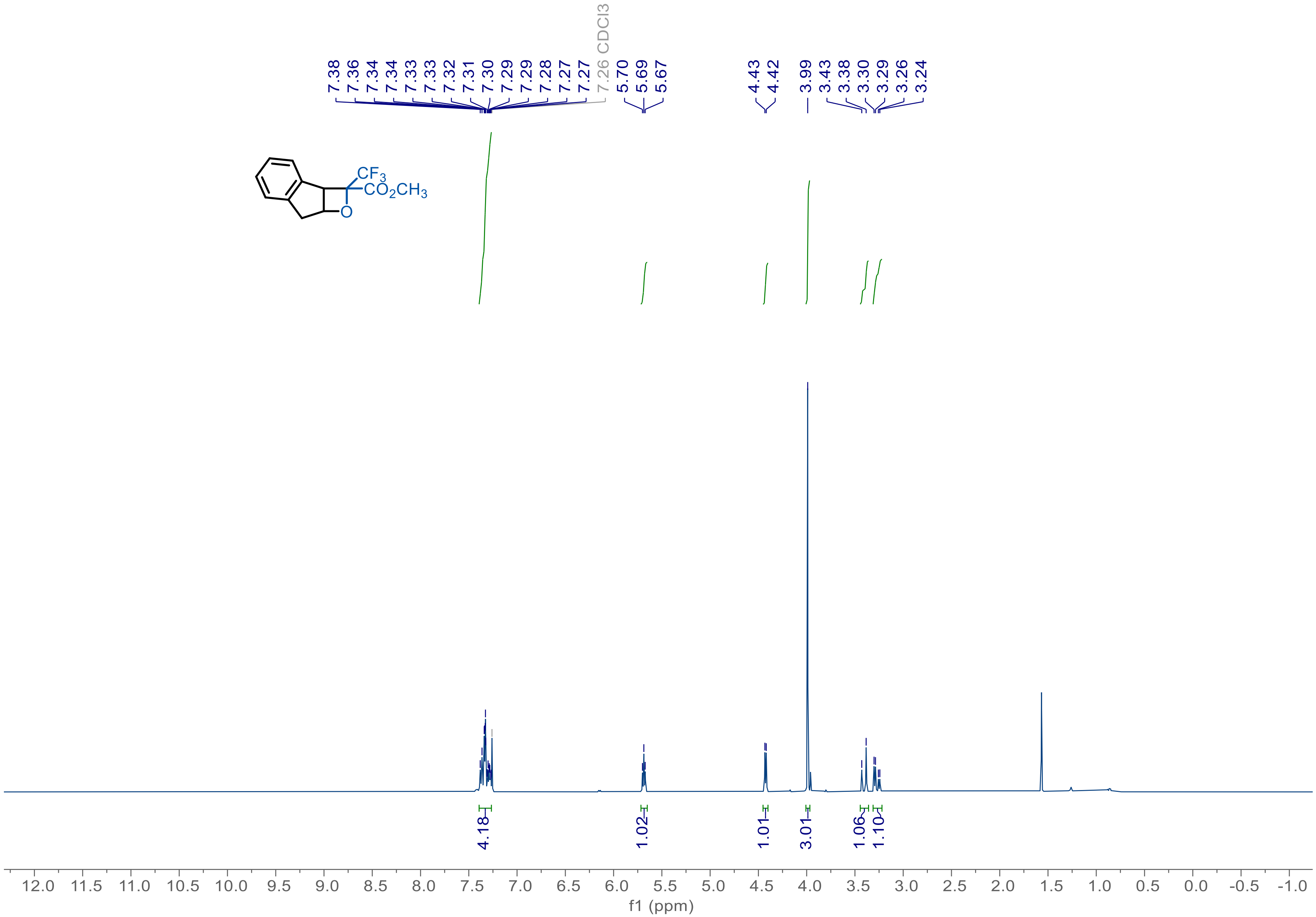

^{13}C NMR (101 MHz, $CDCl_3$) of **18a**

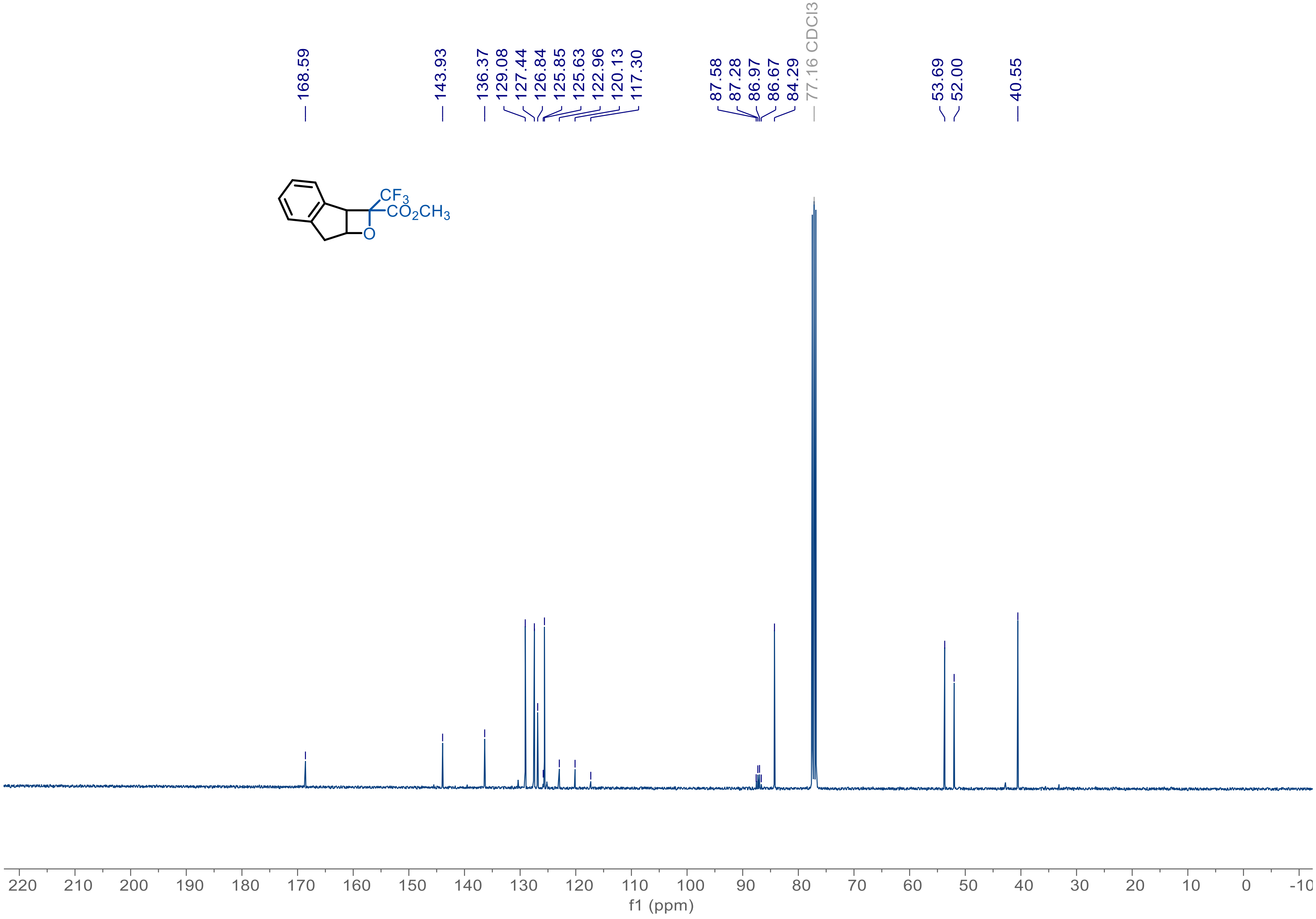

^{19}F NMR (282 MHz, $CDCl_3$) of **18a**

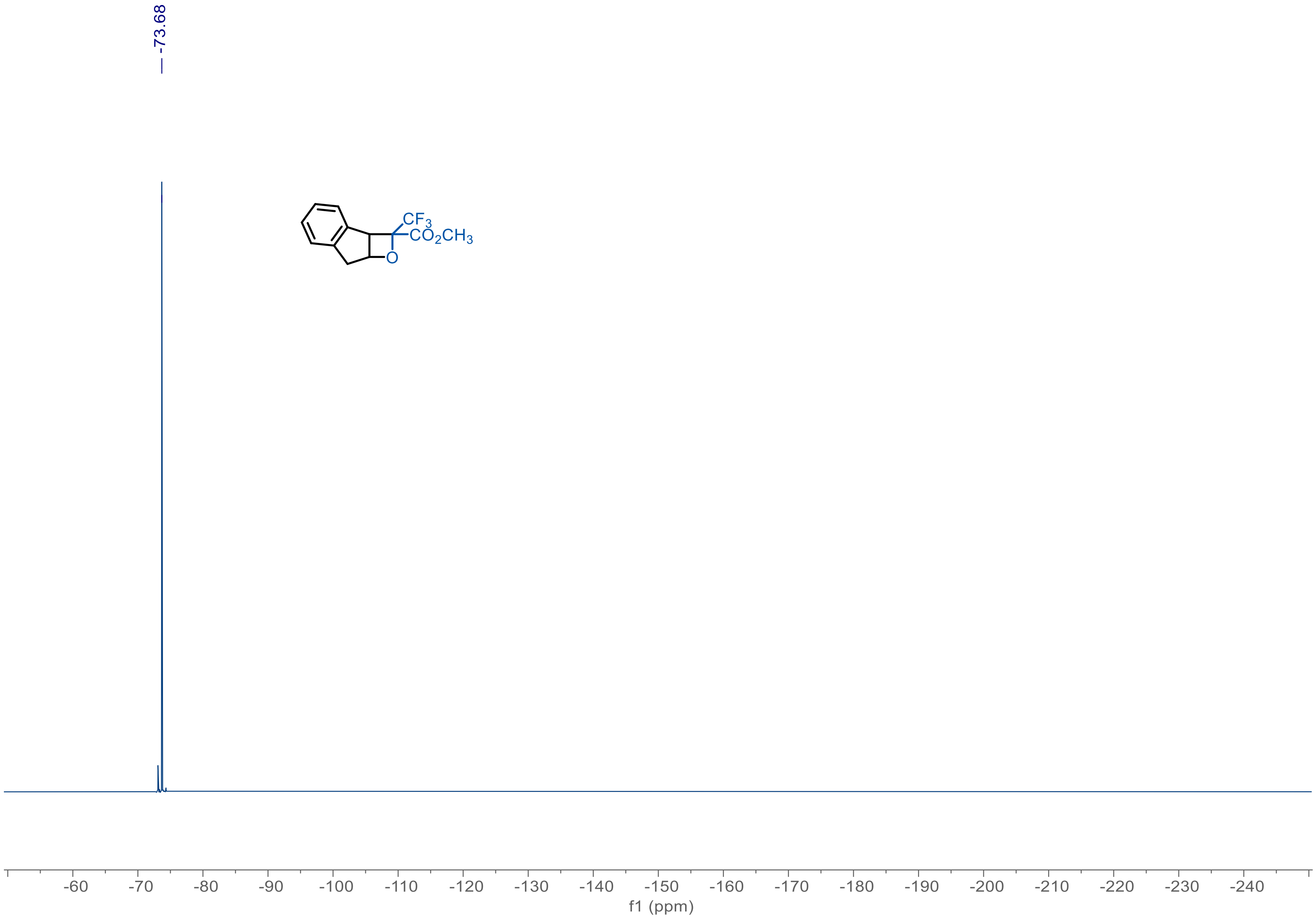

^{1}H NMR (300 MHz, $CDCl_3$) of **19a**

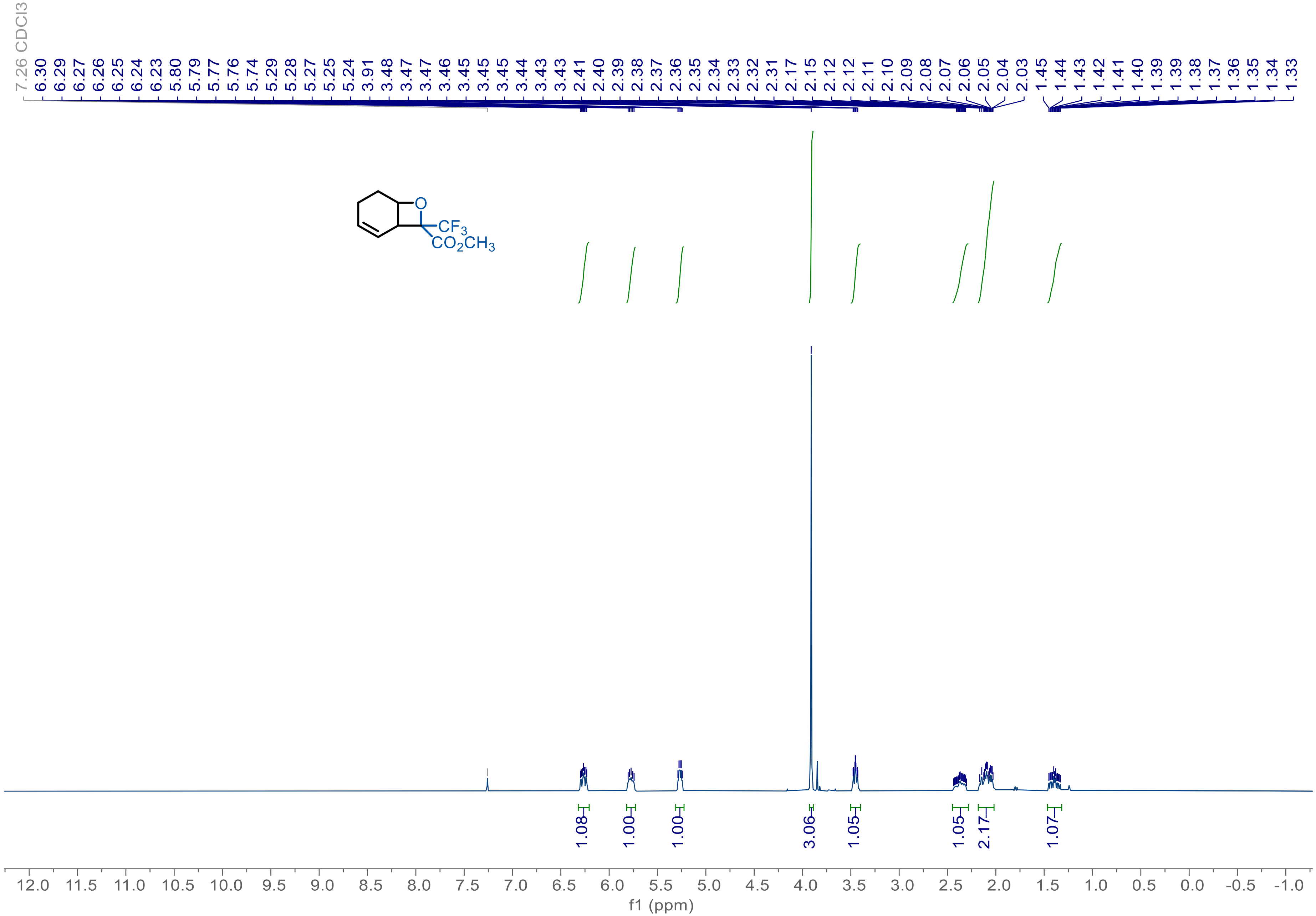

^{13}C NMR (75 MHz, $CDCl_3$) of **19a**

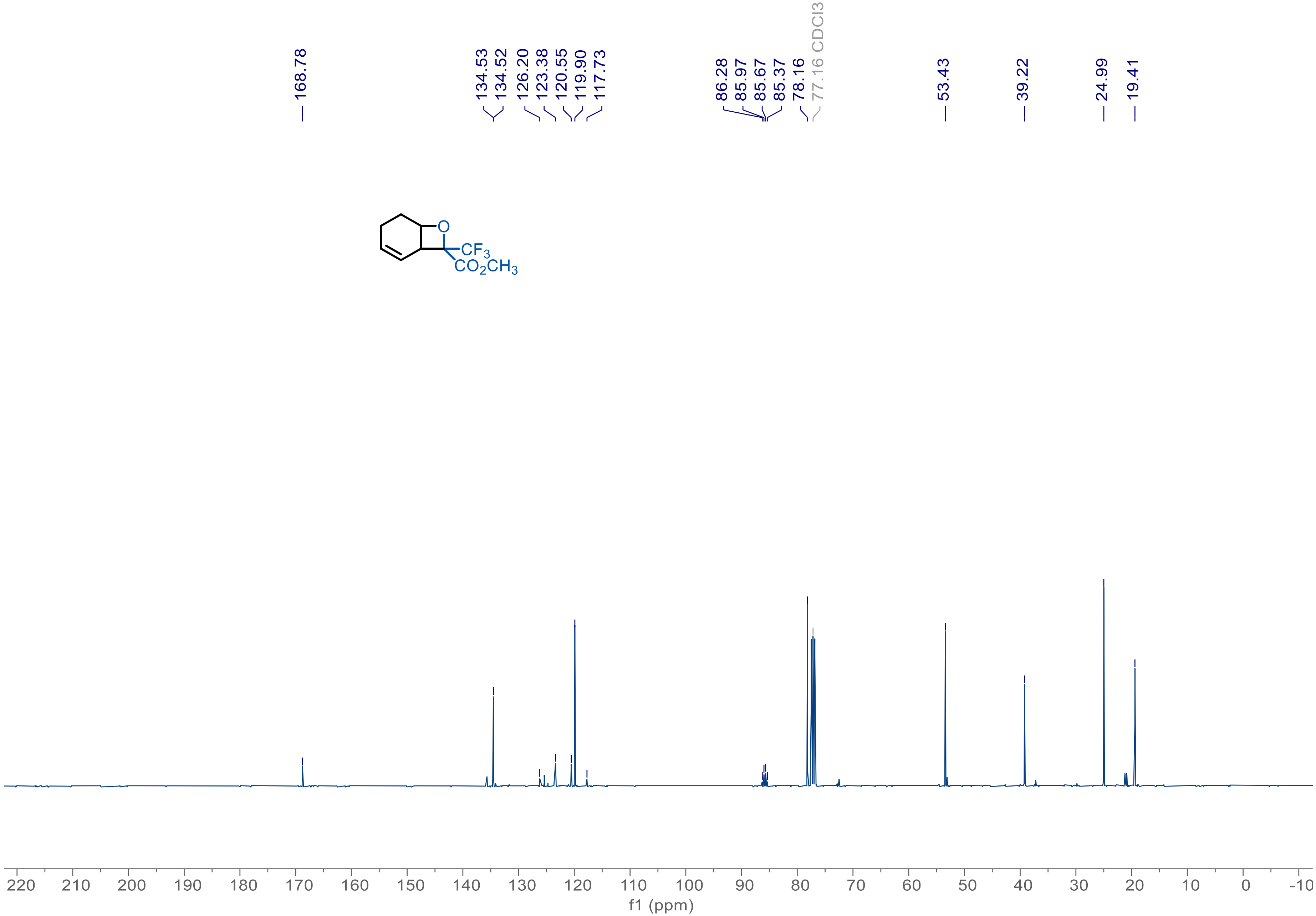

^{19}F NMR (282 MHz, $CDCl_3$) of **19a**

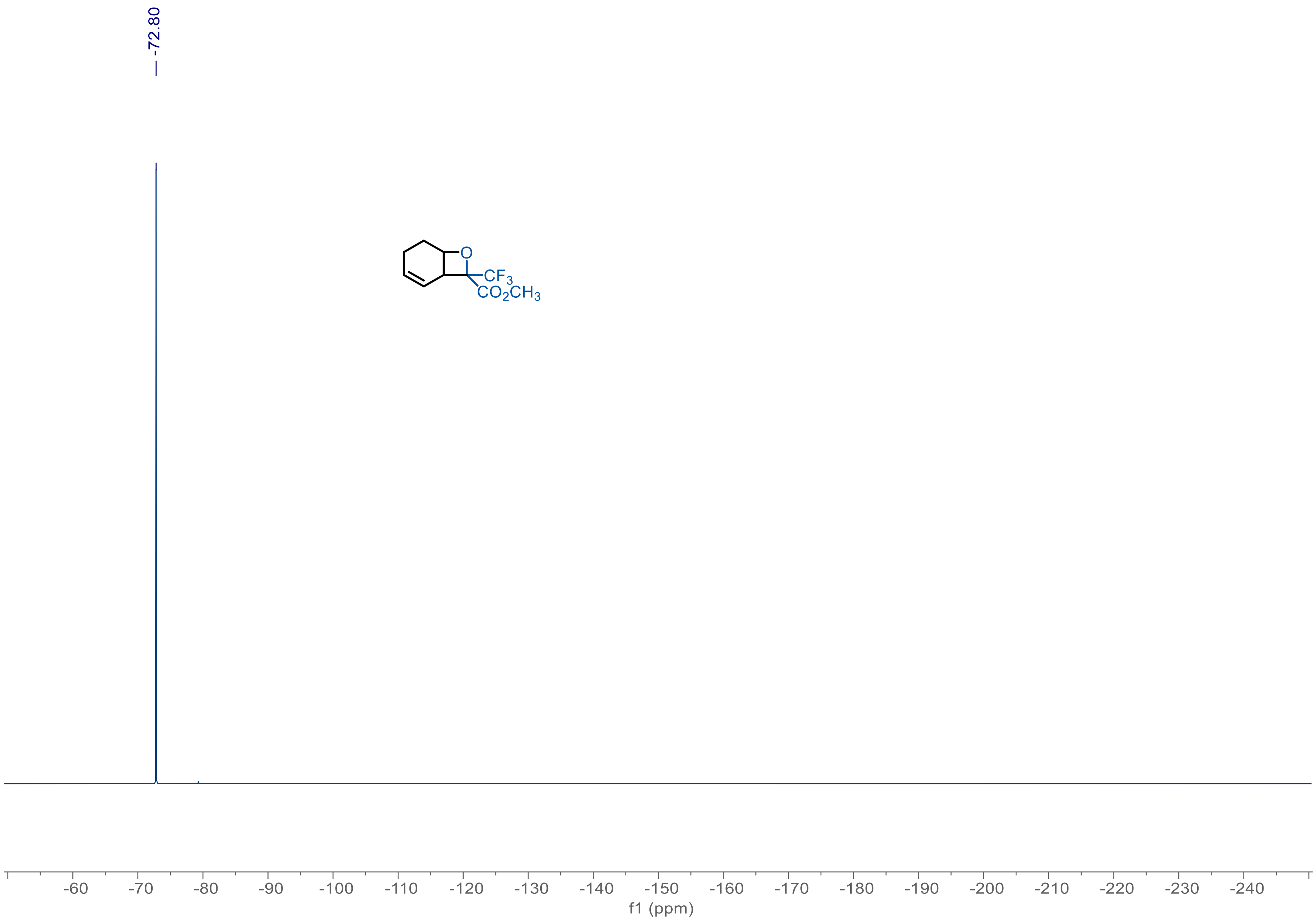

^{1}H NMR (300 MHz, $CDCl_3$) of **20a**

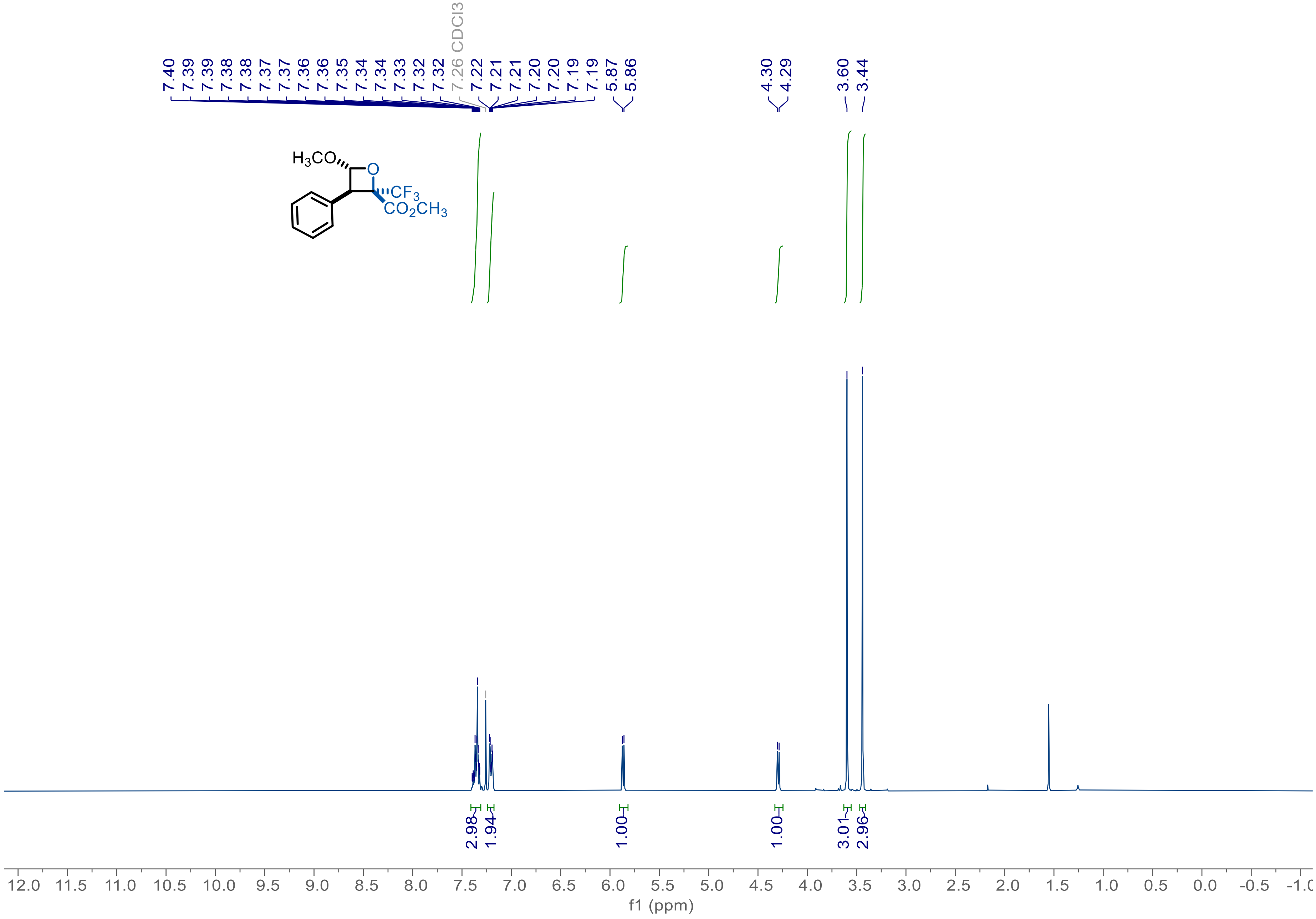

^{13}C NMR (101 MHz, $CDCl_3$) of **20a**

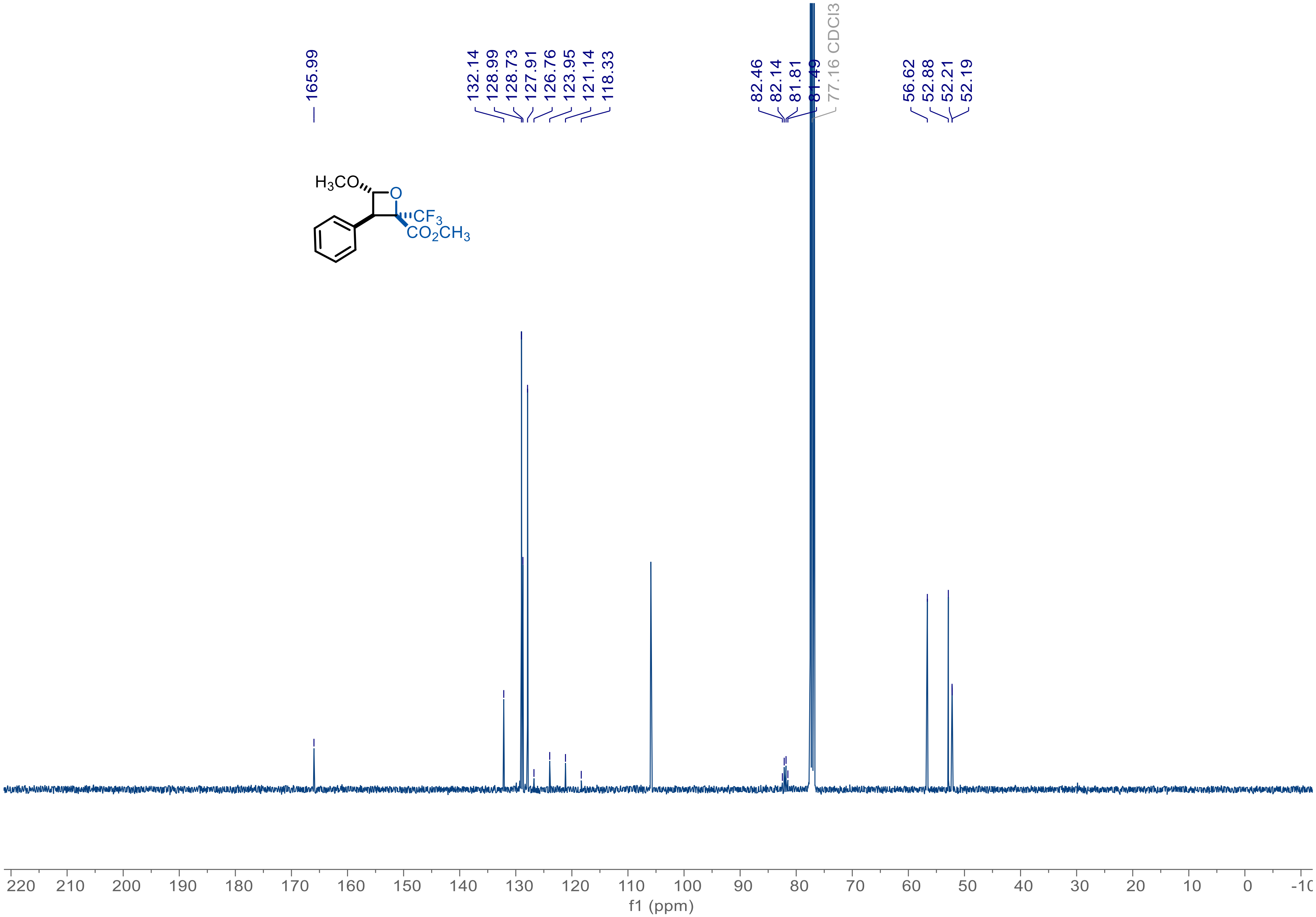

^{19}F NMR (282 MHz, $CDCl_3$) of **20a**

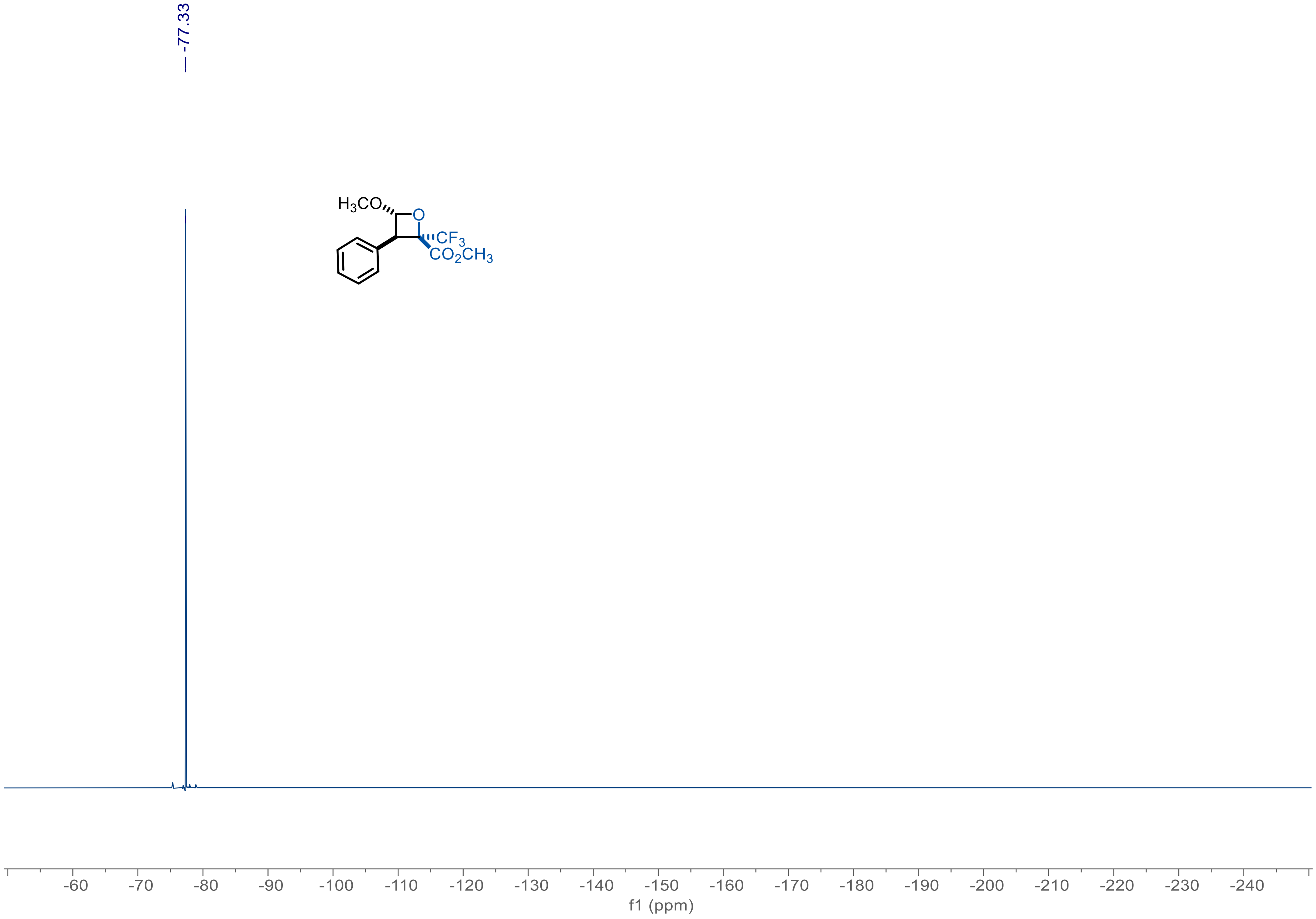

^{1}H NMR (300 MHz, $CDCl_3$) of **21a**

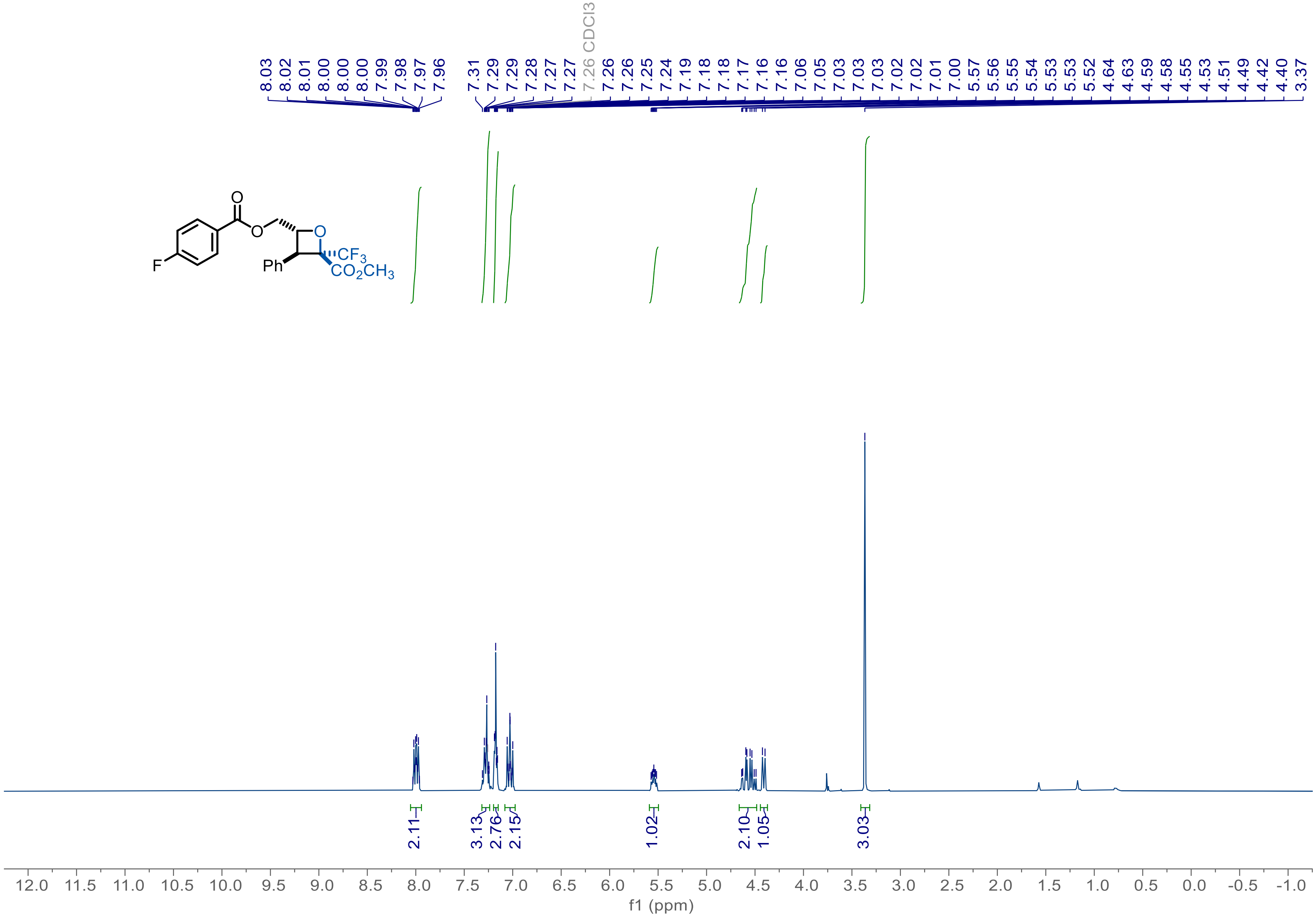

^{13}C NMR (75 MHz, $CDCl_3$) of **21a**

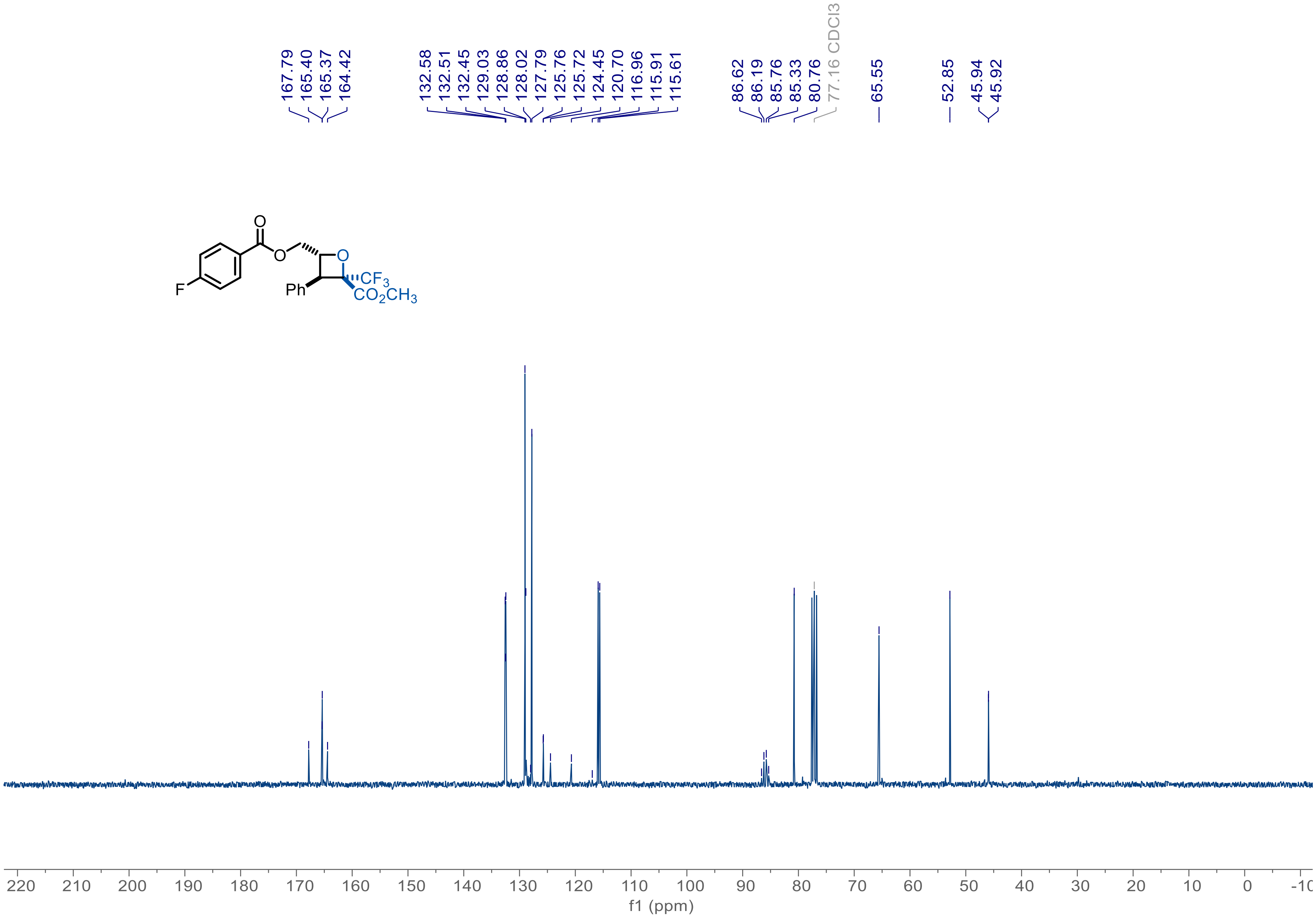

^{19}F NMR (282 MHz, $CDCl_3$) of **21a**

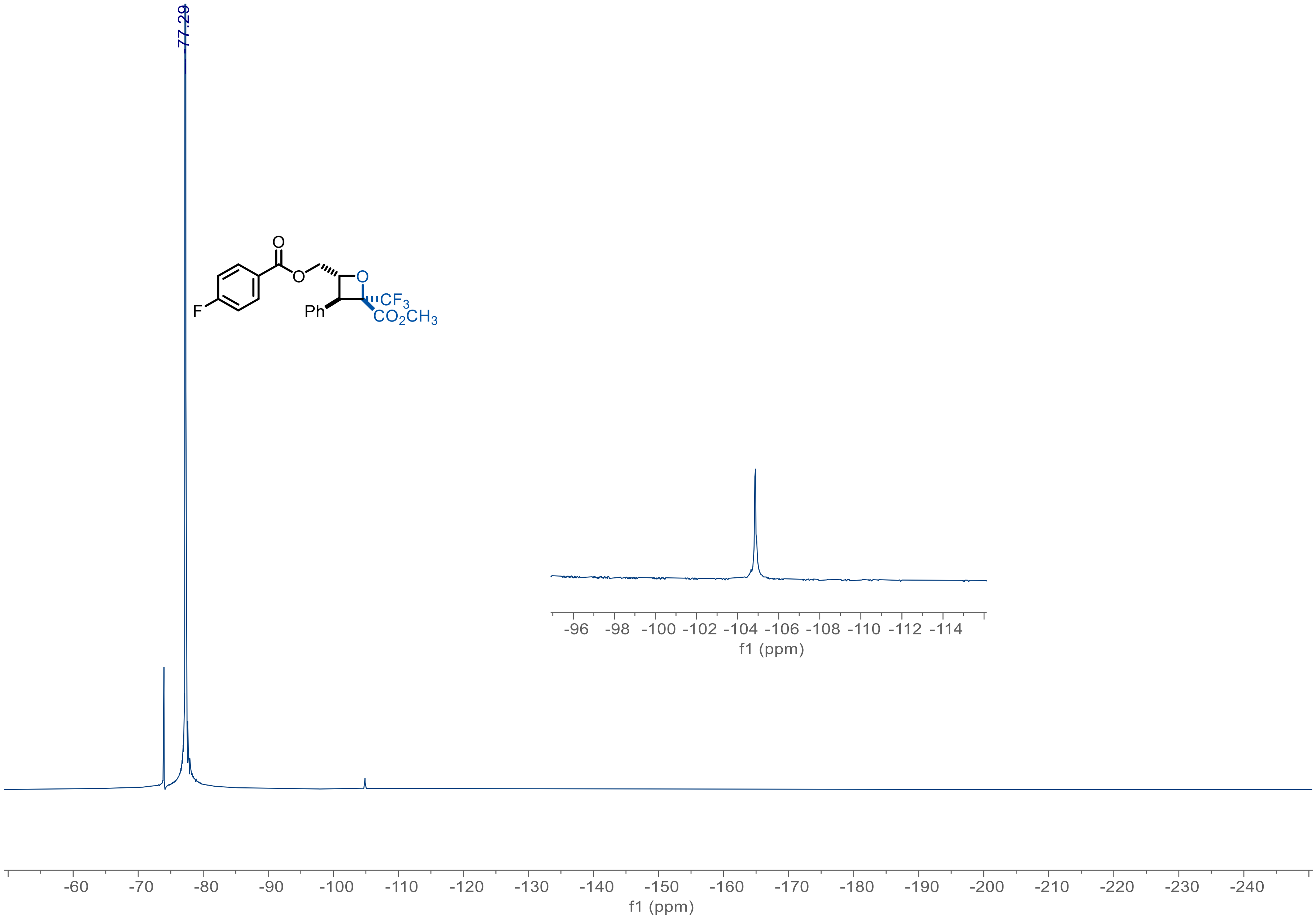

^{1}H NMR (300 MHz, $CDCl_3$) of **22a**

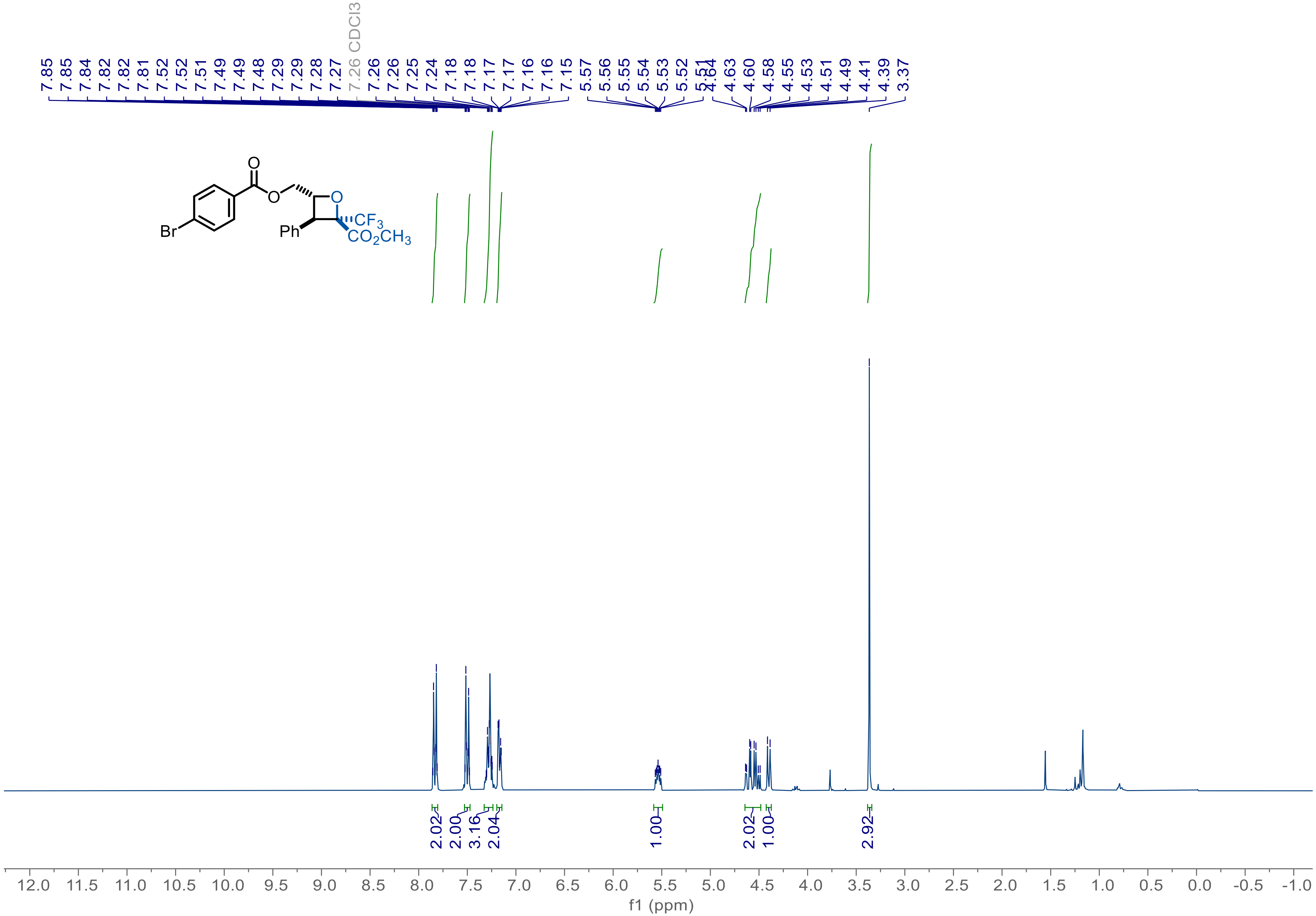

^{13}C NMR (75 MHz, CDCl$_3$) of **22a**

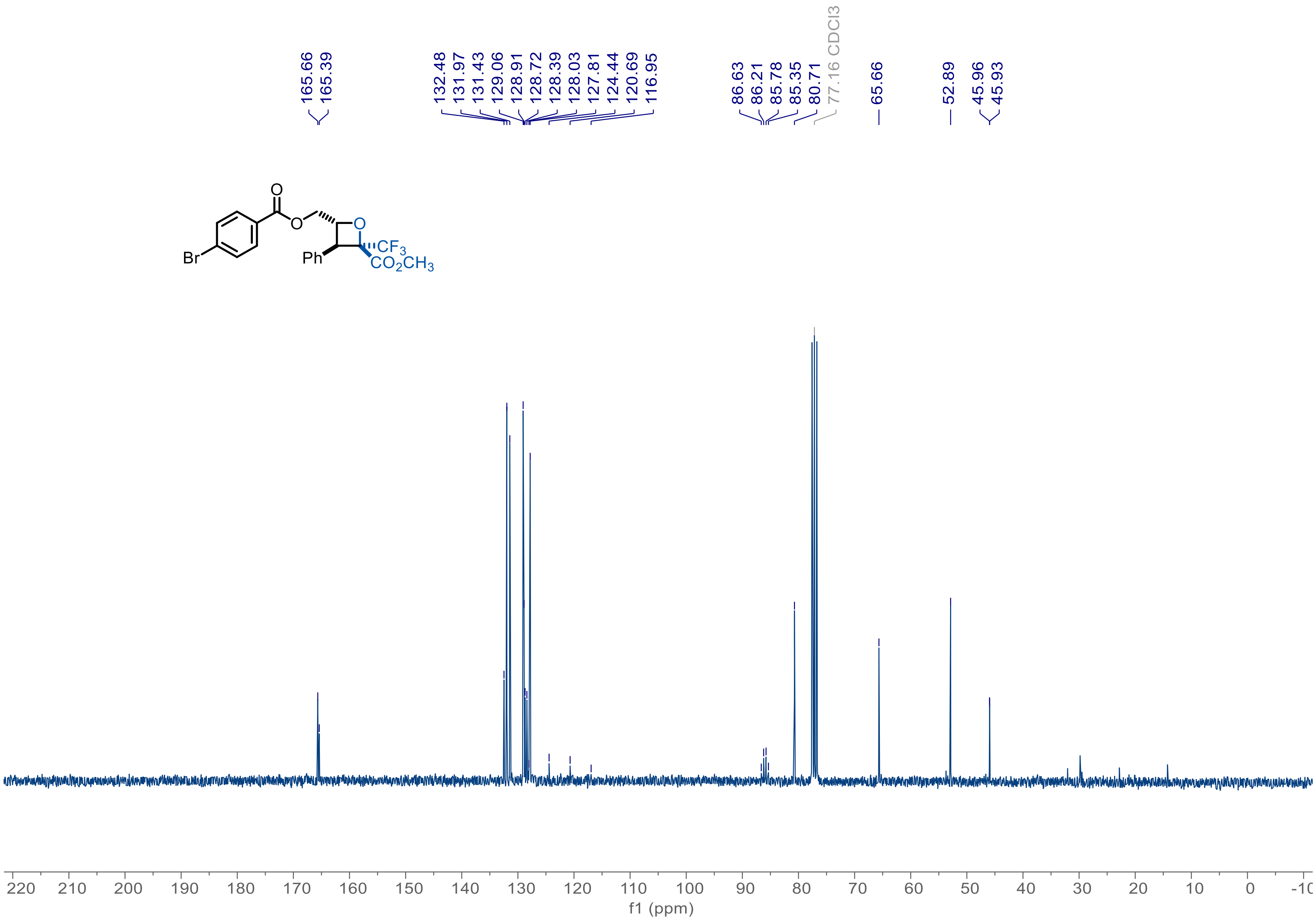

^{19}F NMR (282 MHz, $CDCl_3$) of **22a**

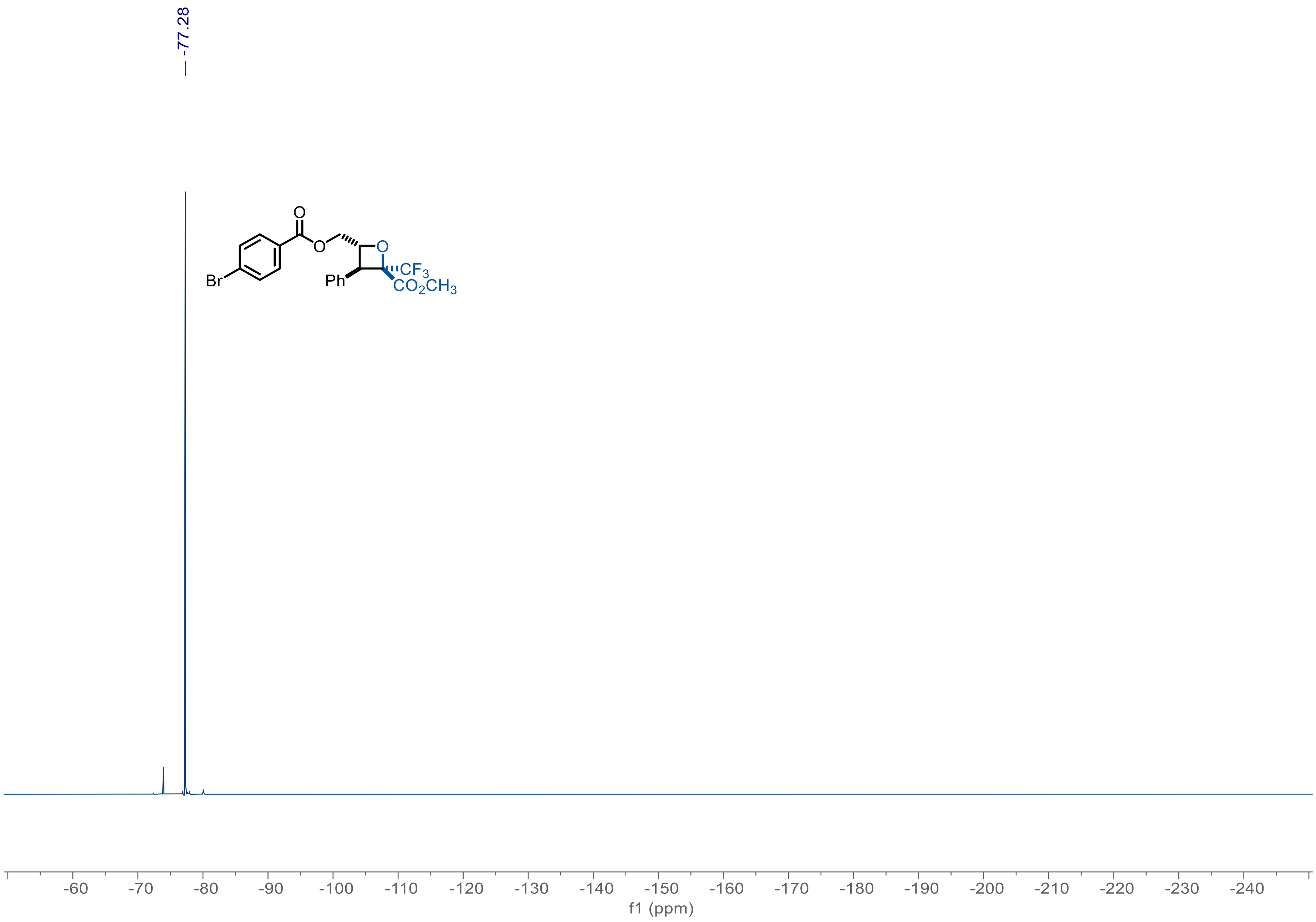

^{1}H NMR (300 MHz, $CDCl_3$) of **23a**

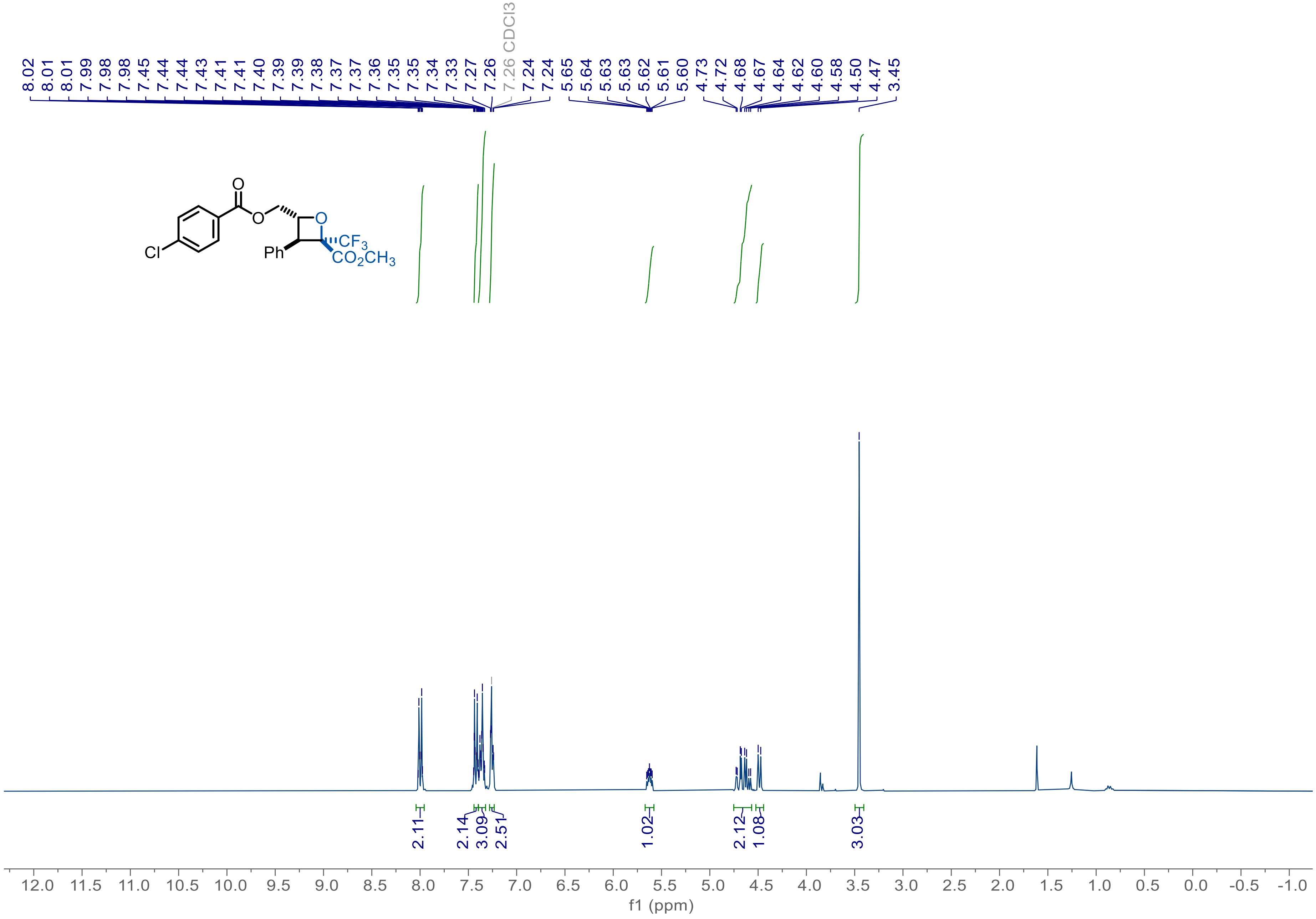

^{13}C NMR (75 MHz, $CDCl_3$) of **23a**

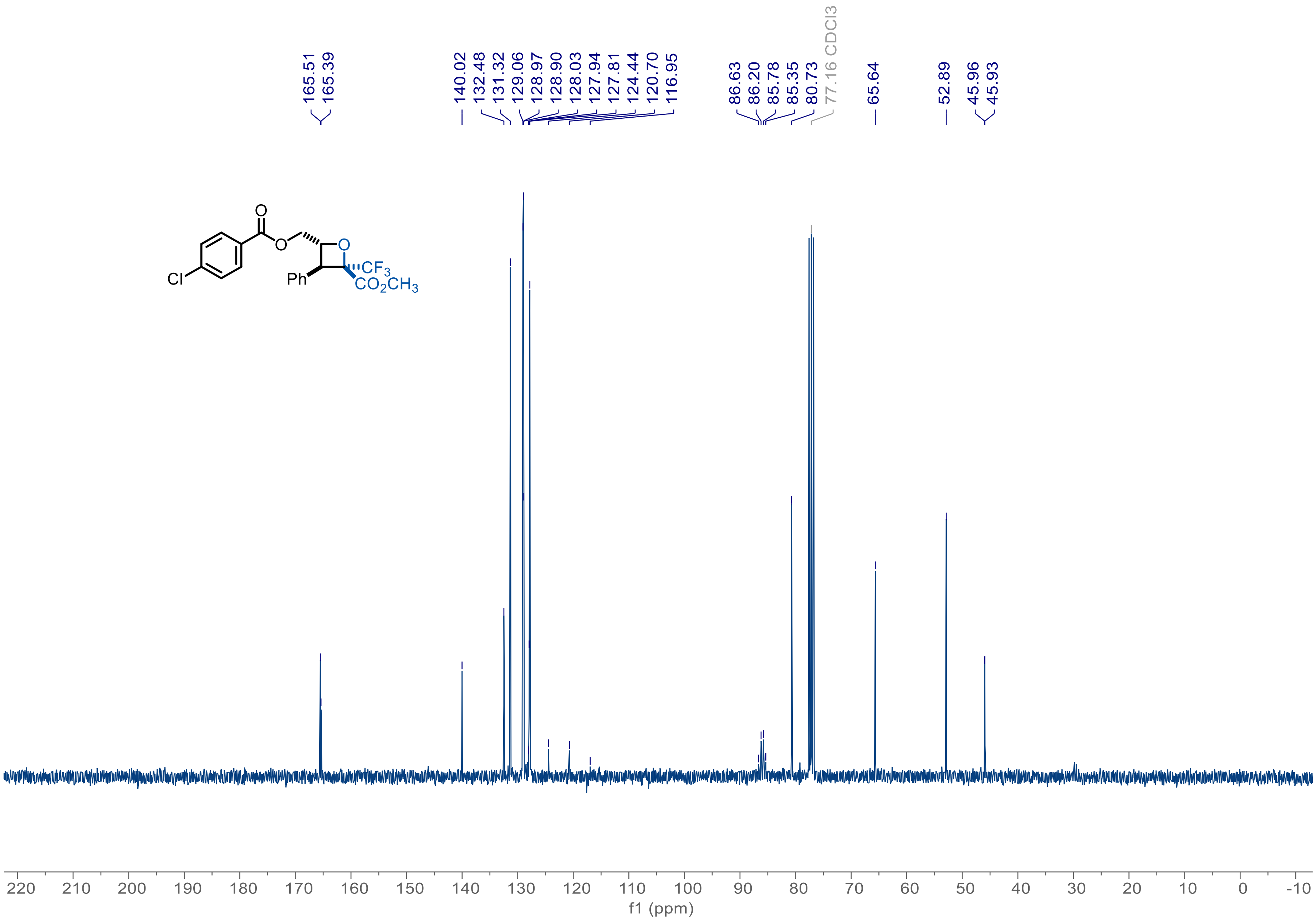

^{19}F NMR (282 MHz, $CDCl_3$) of **23a**

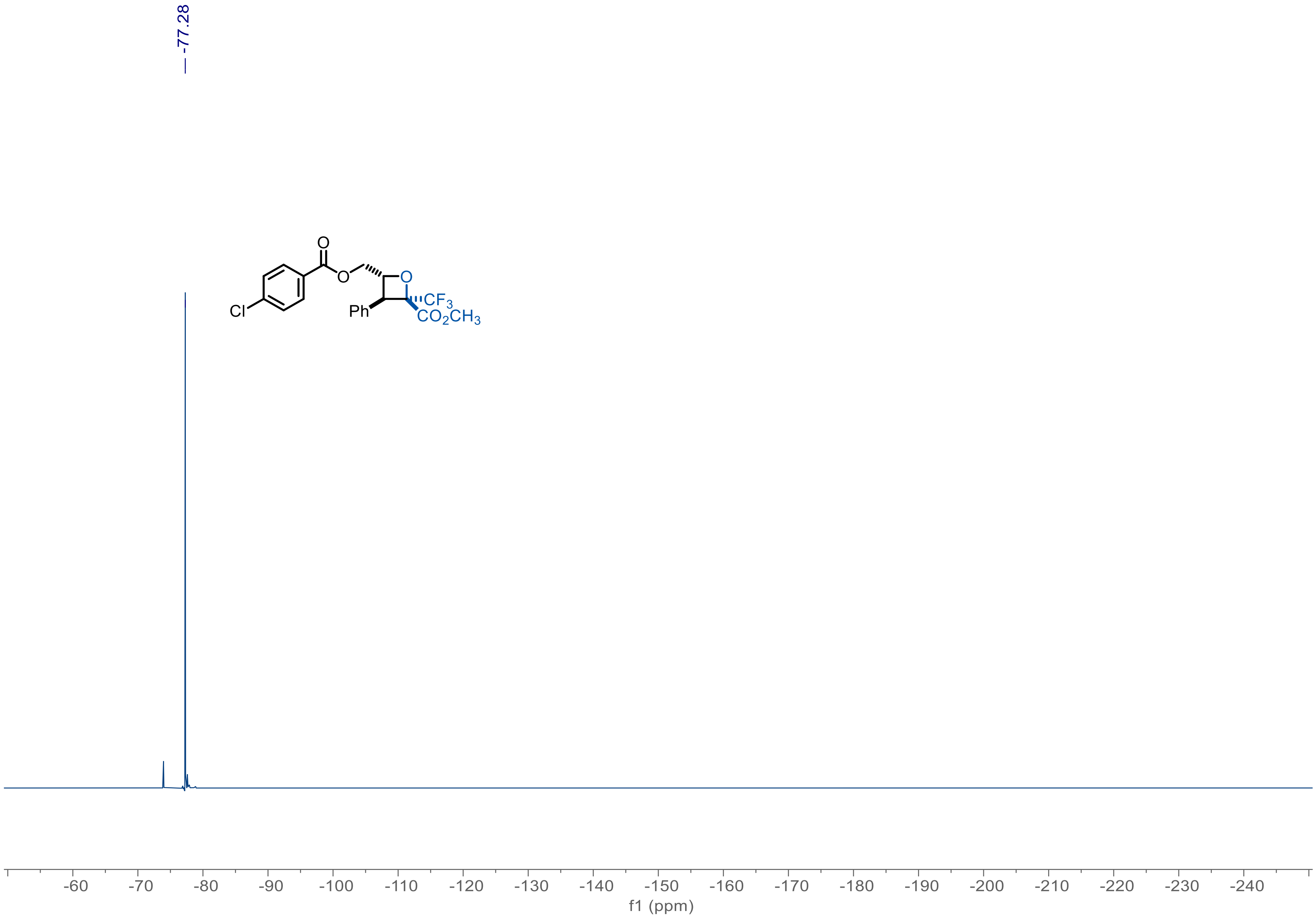

^{1}H NMR (300 MHz, $CDCl_3$) of **24a**

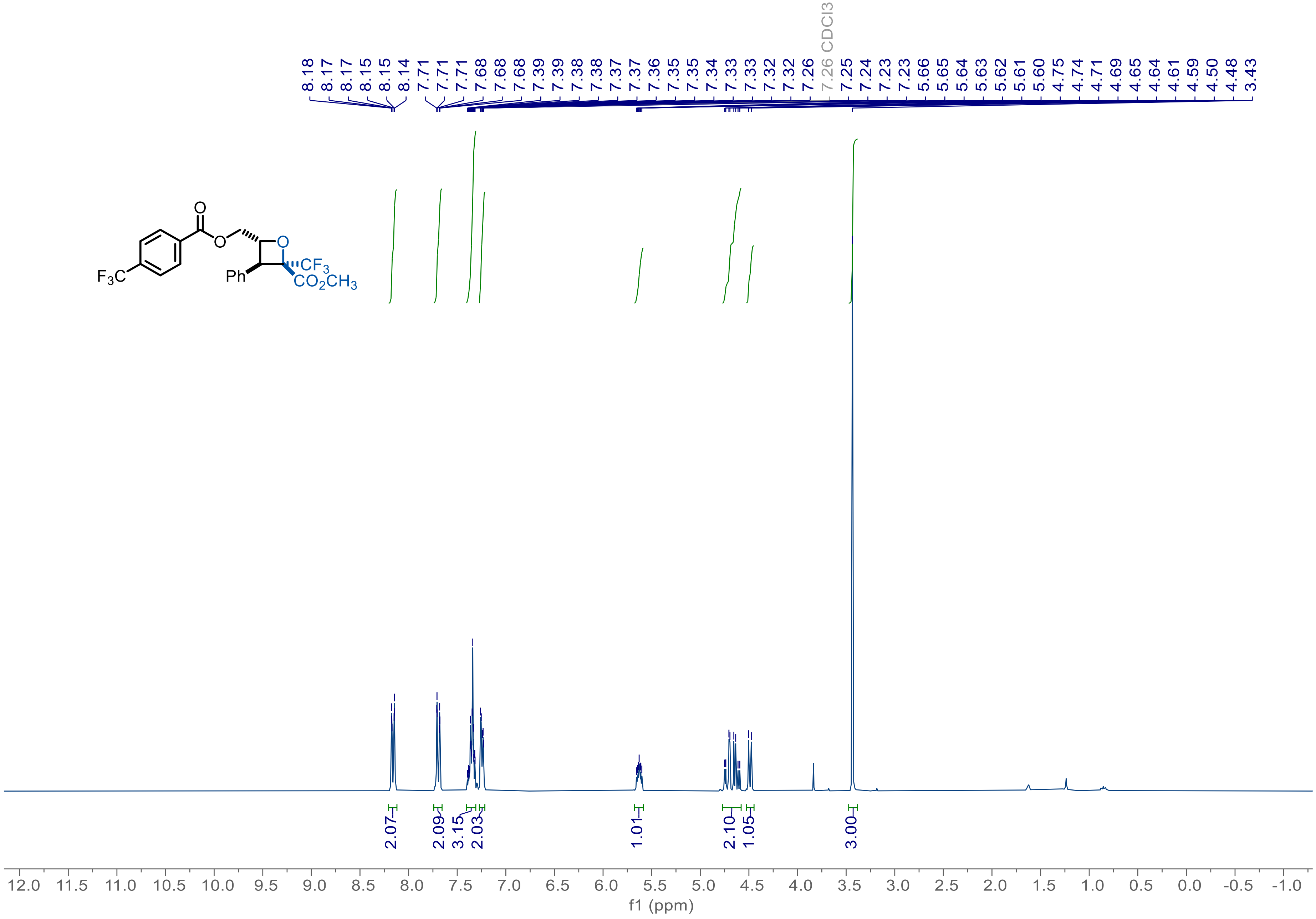

^{13}C NMR (75 MHz, $CDCl_3$) of **24a**

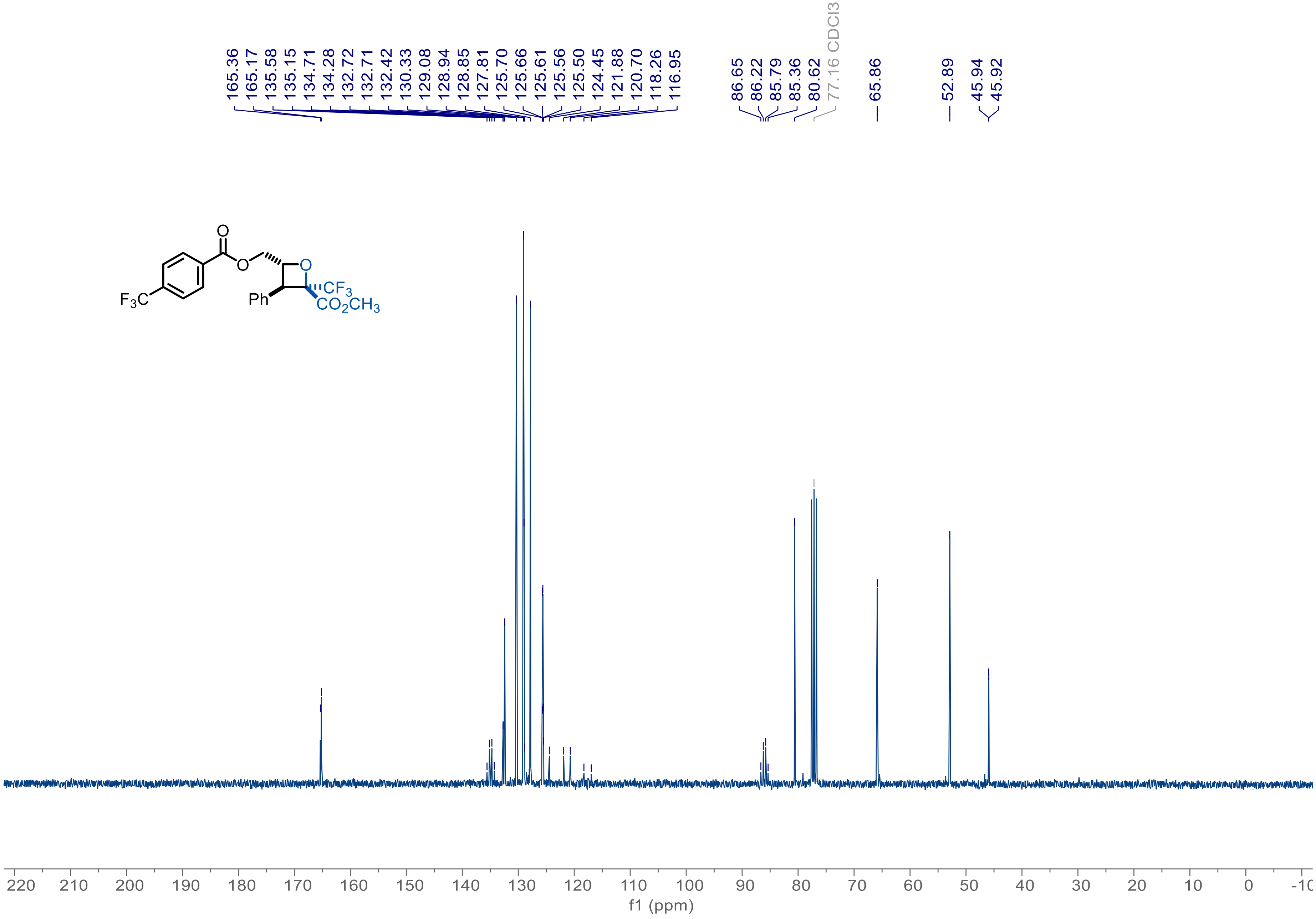

^{19}F NMR (282 MHz, $CDCl_3$) of **24a**

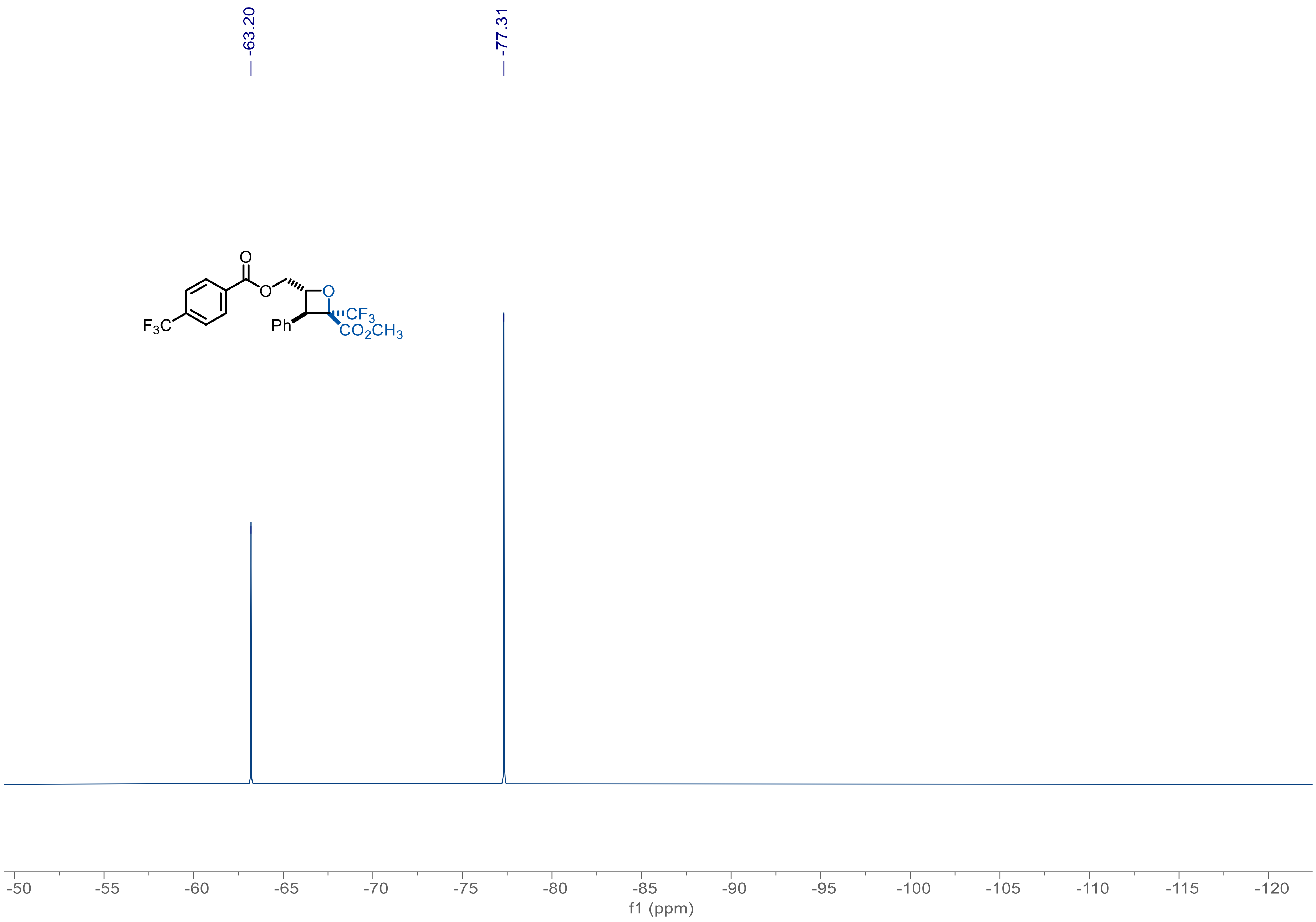

^{1}H NMR (300 MHz, $CDCl_3$) of **25a**

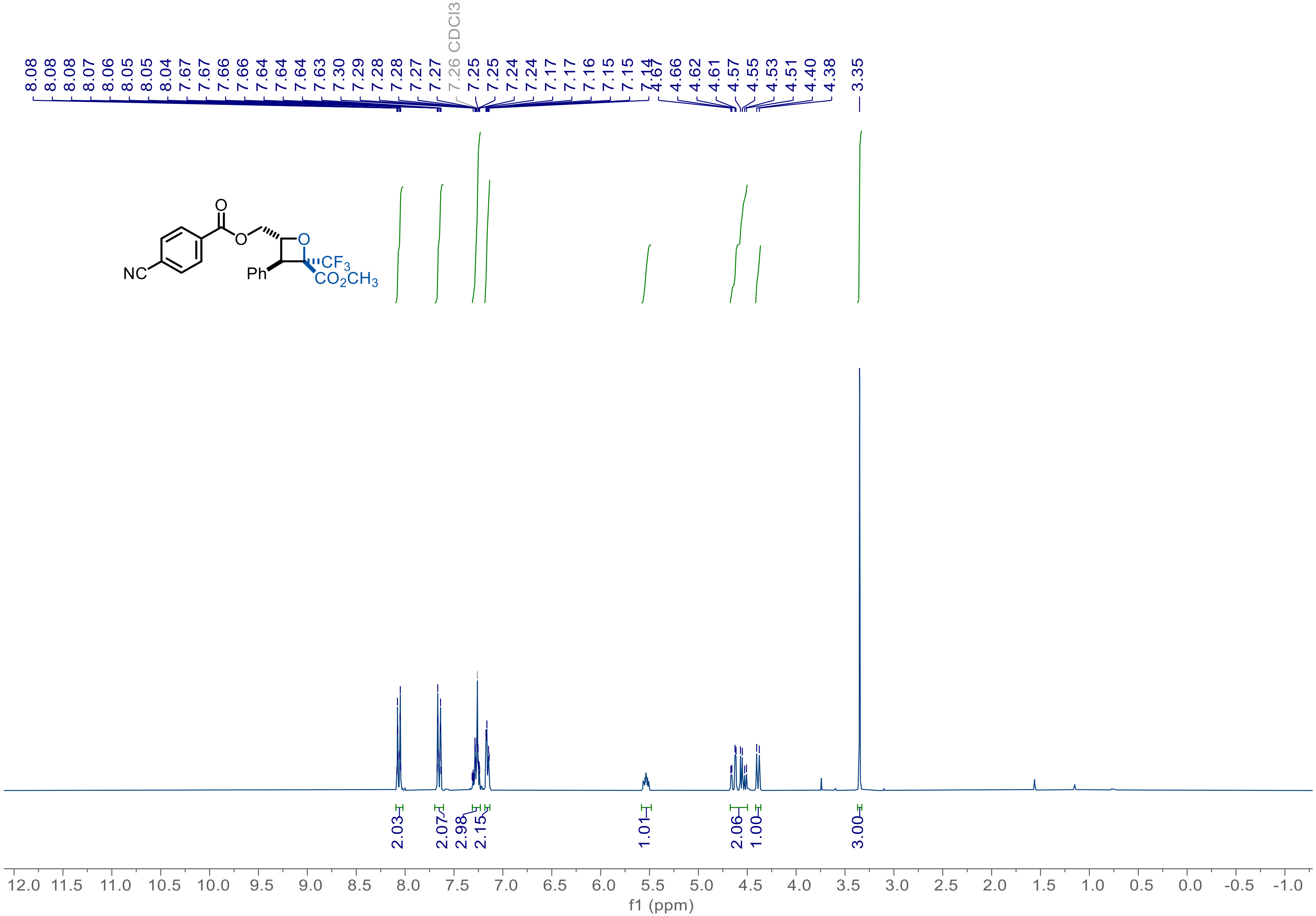

^{13}C NMR (75 MHz, $CDCl_3$) of **25a**

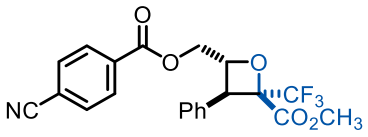

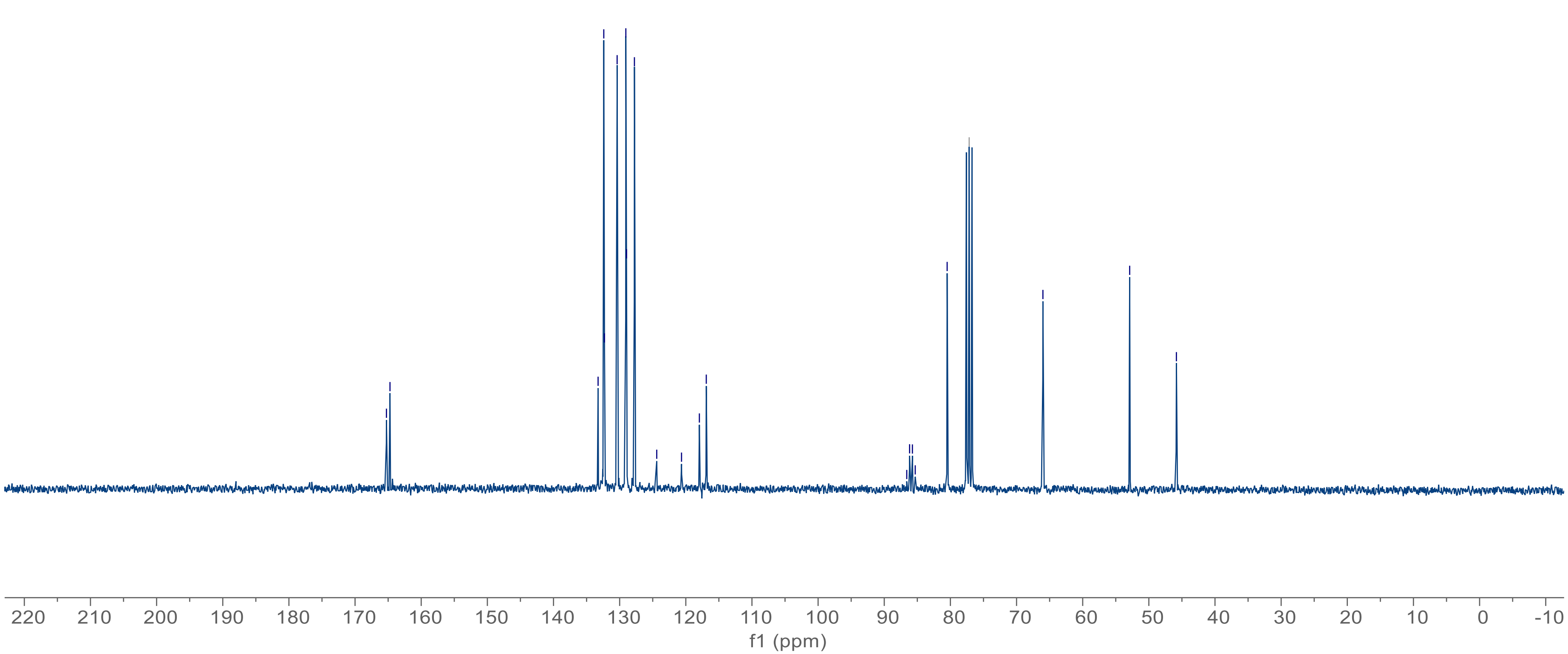

^{19}F NMR (282 MHz, $CDCl_3$) of **25a**

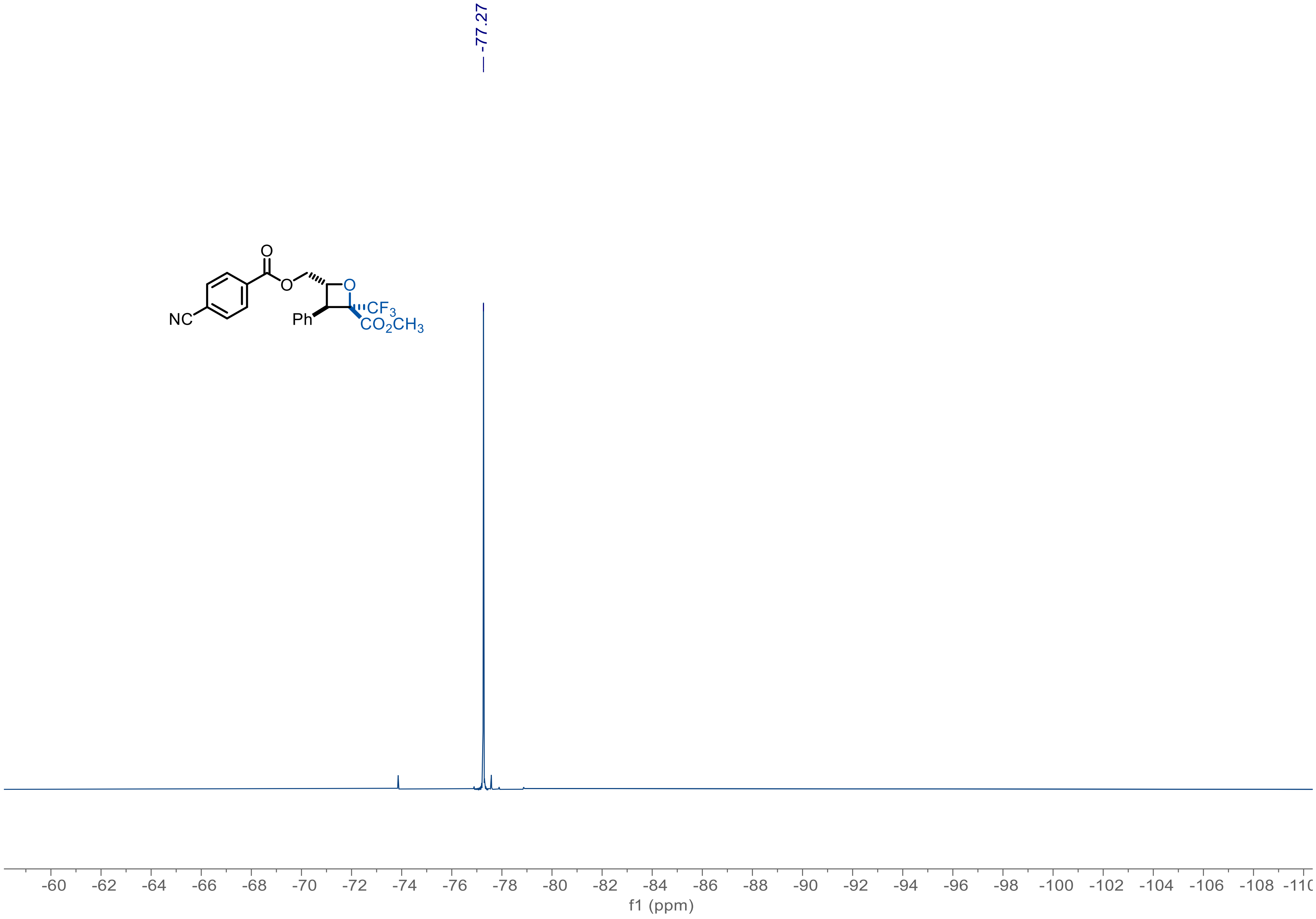

^{1}H NMR (300 MHz, $CDCl_3$) of **26a**

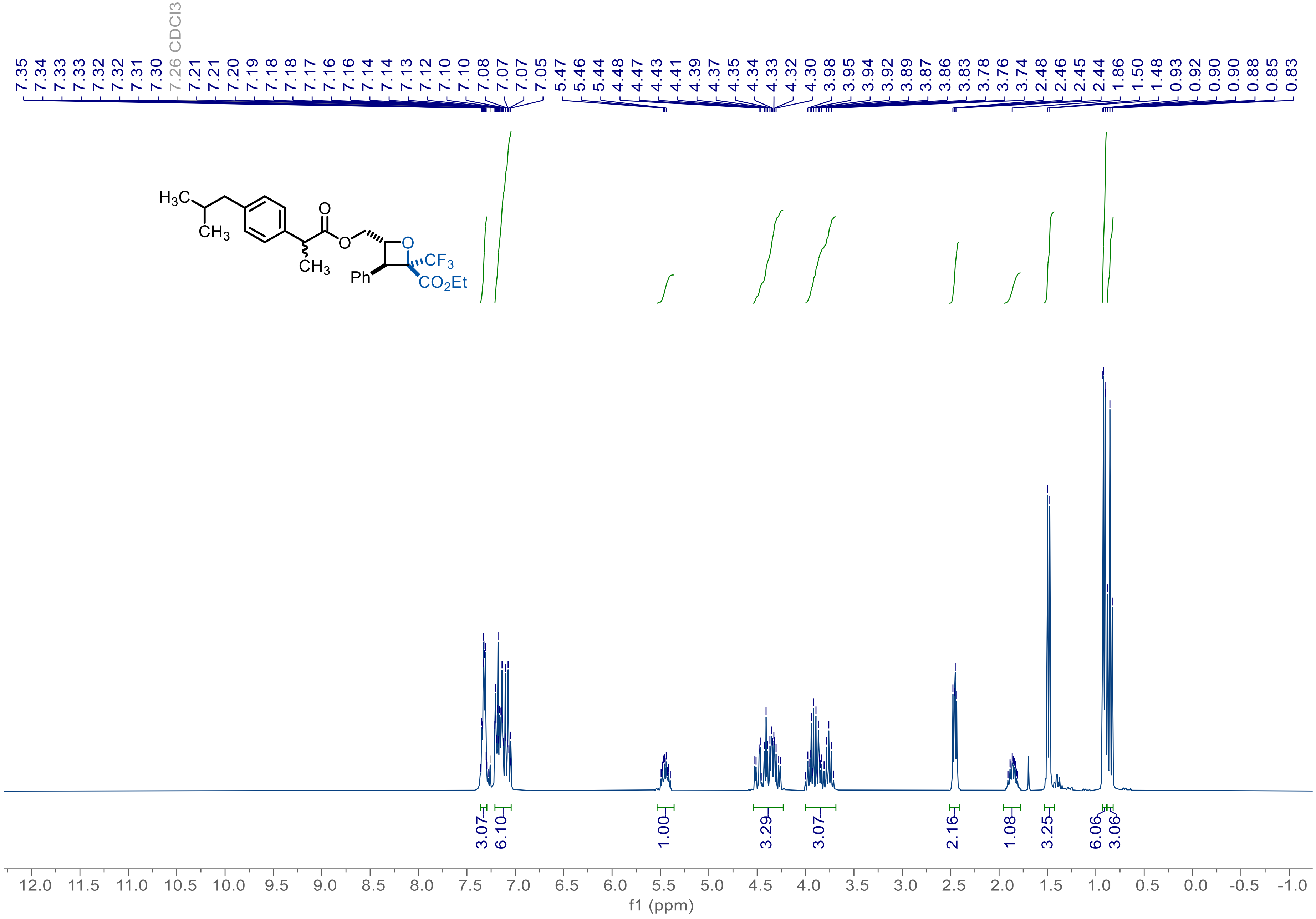

^{13}C NMR (75 MHz, $CDCl_3$) of **26a**

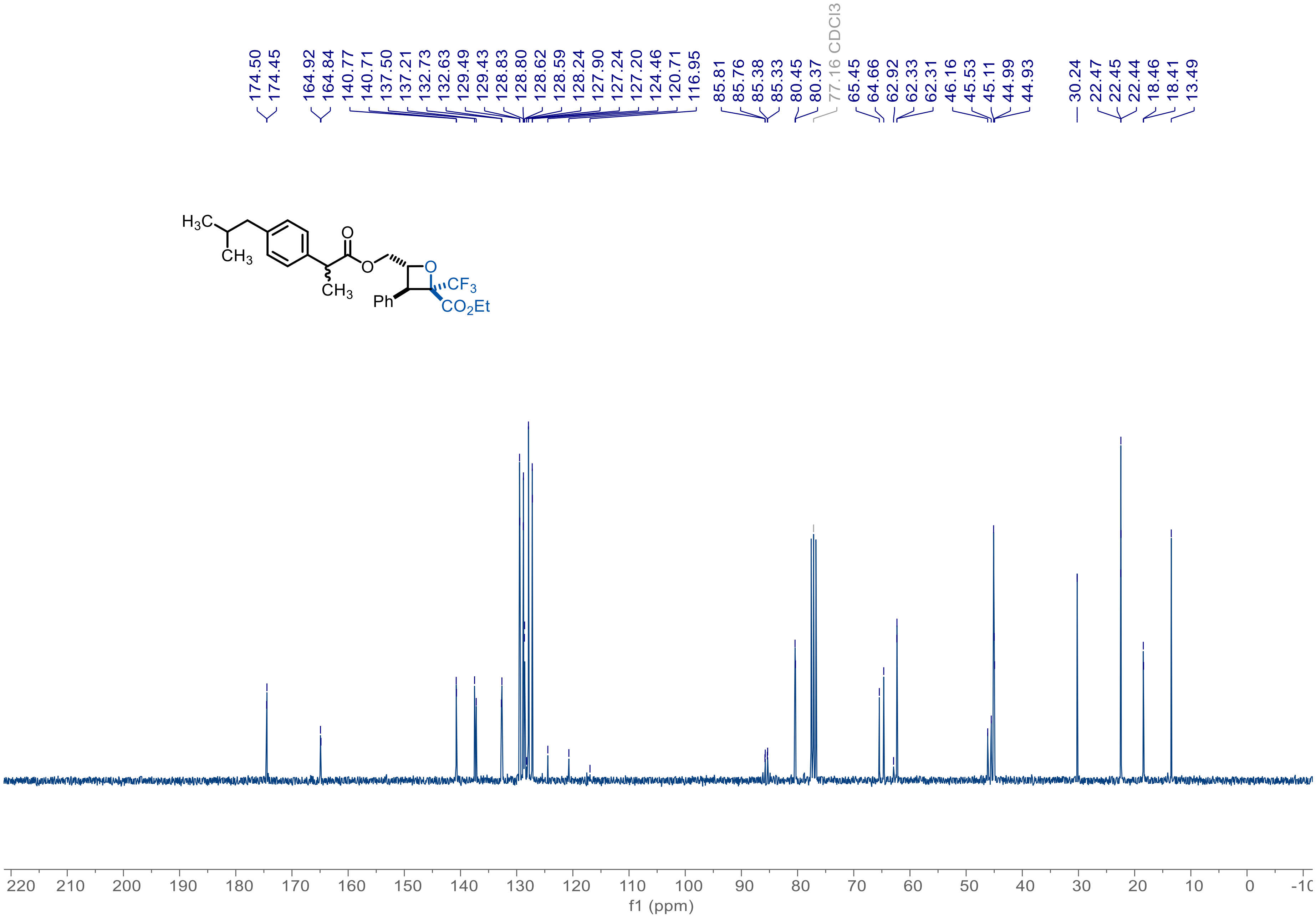

^{19}F NMR (282 MHz, $CDCl_3$) of **26a**

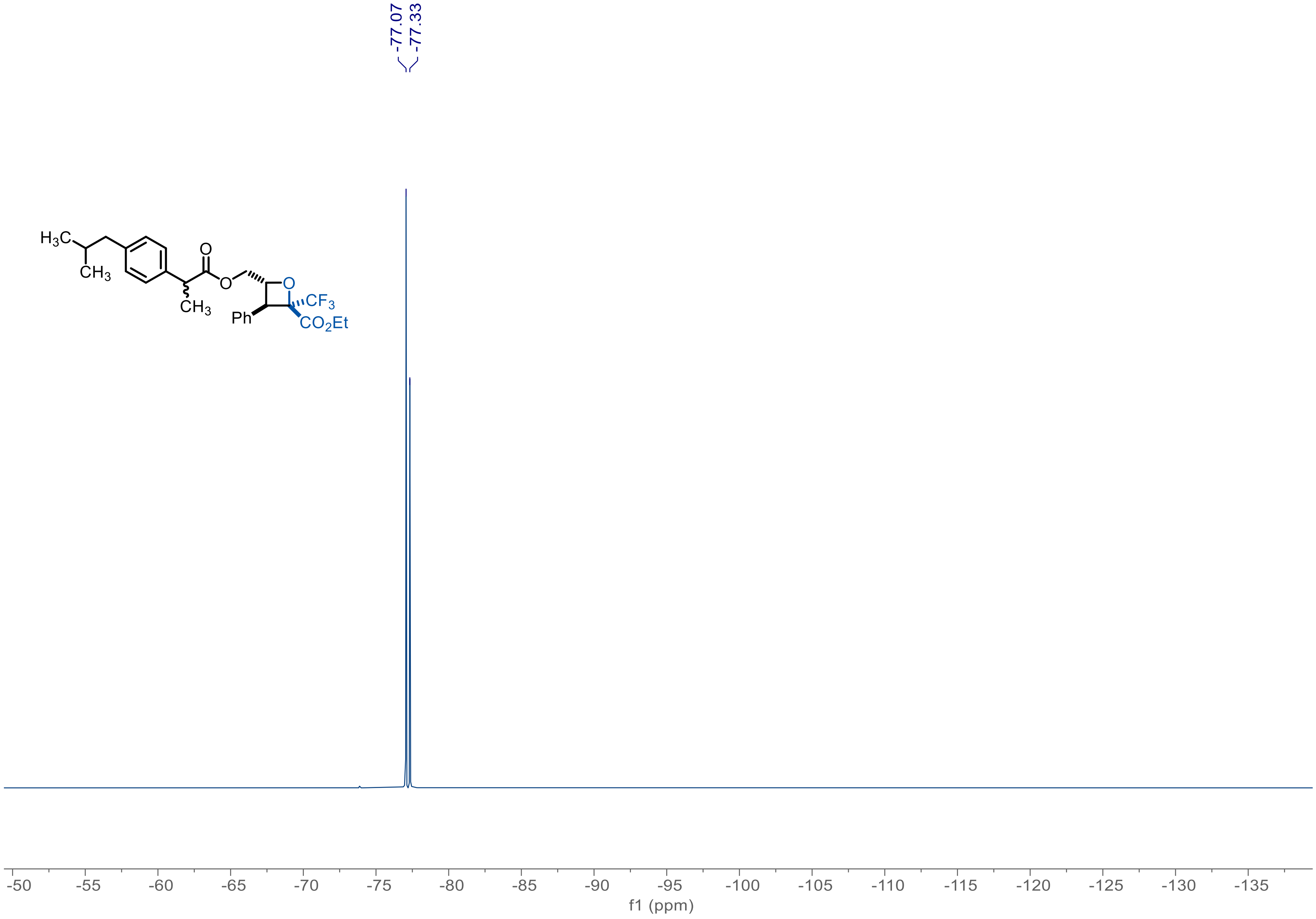

^{1}H NMR (300 MHz, $CDCl_3$) of **(Z)-27**

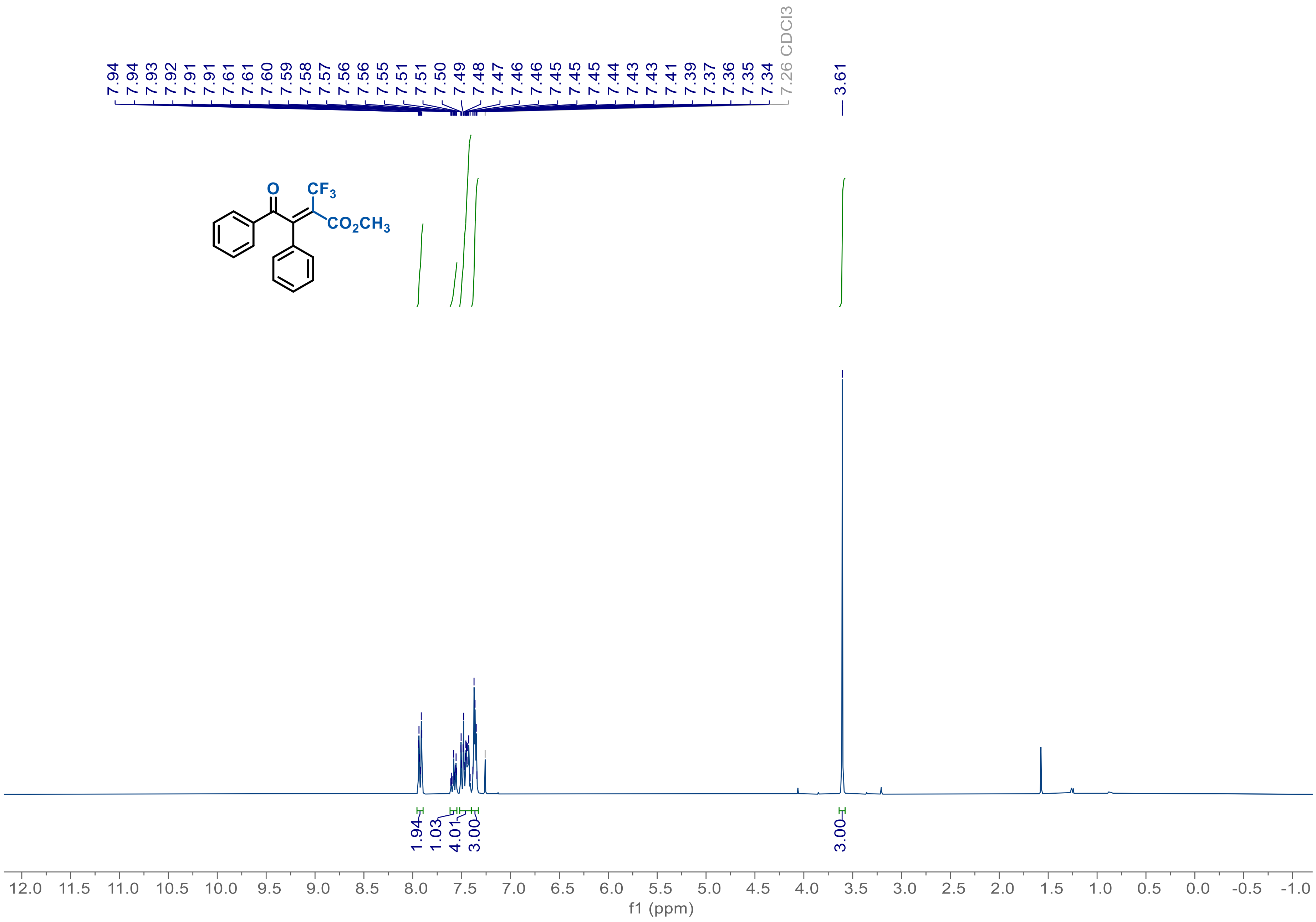

^{13}C NMR (75 MHz, $CDCl_3$) of **(Z)-27**

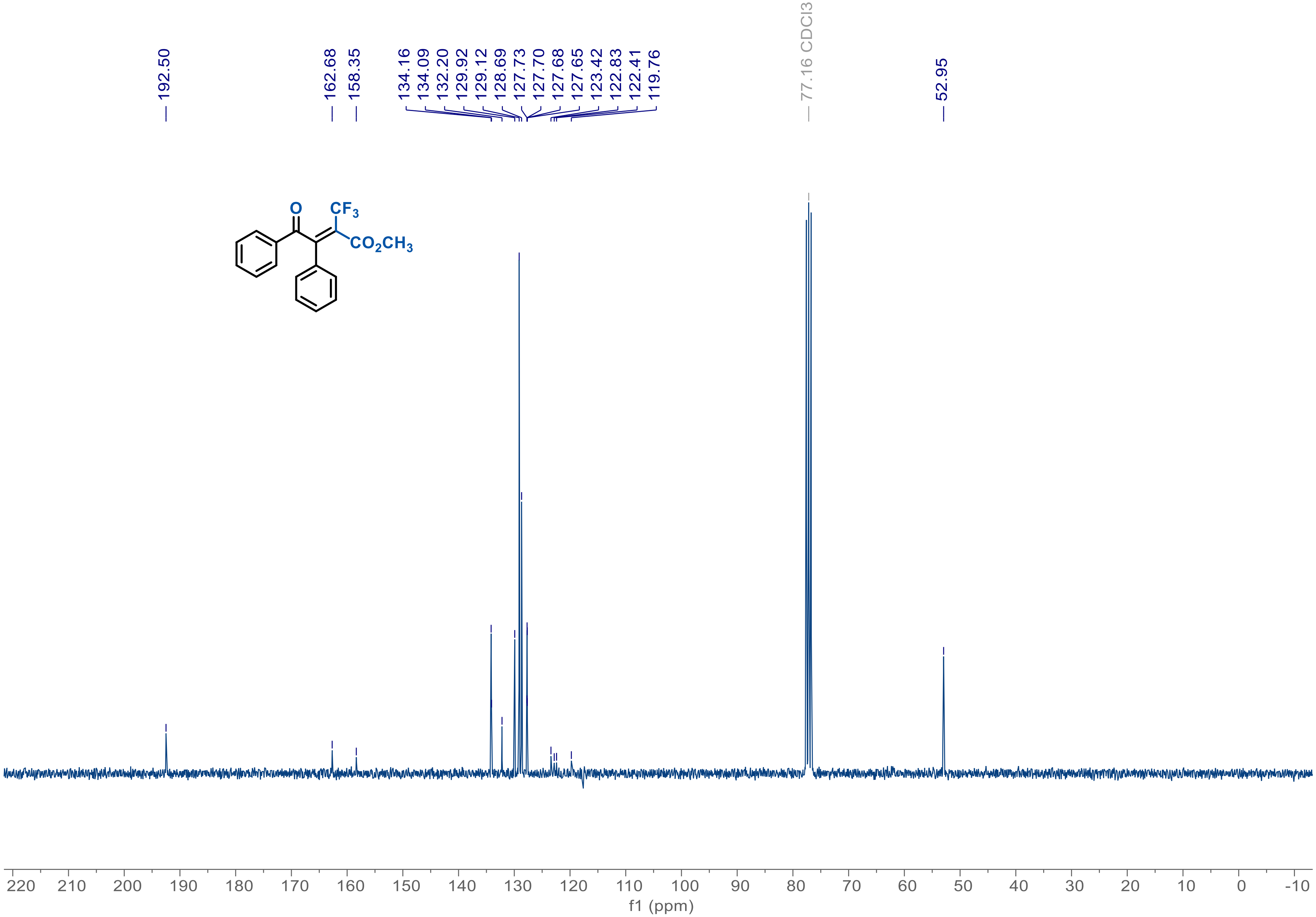

^{19}F NMR (282 MHz, $CDCl_3$) of **(Z)-27**

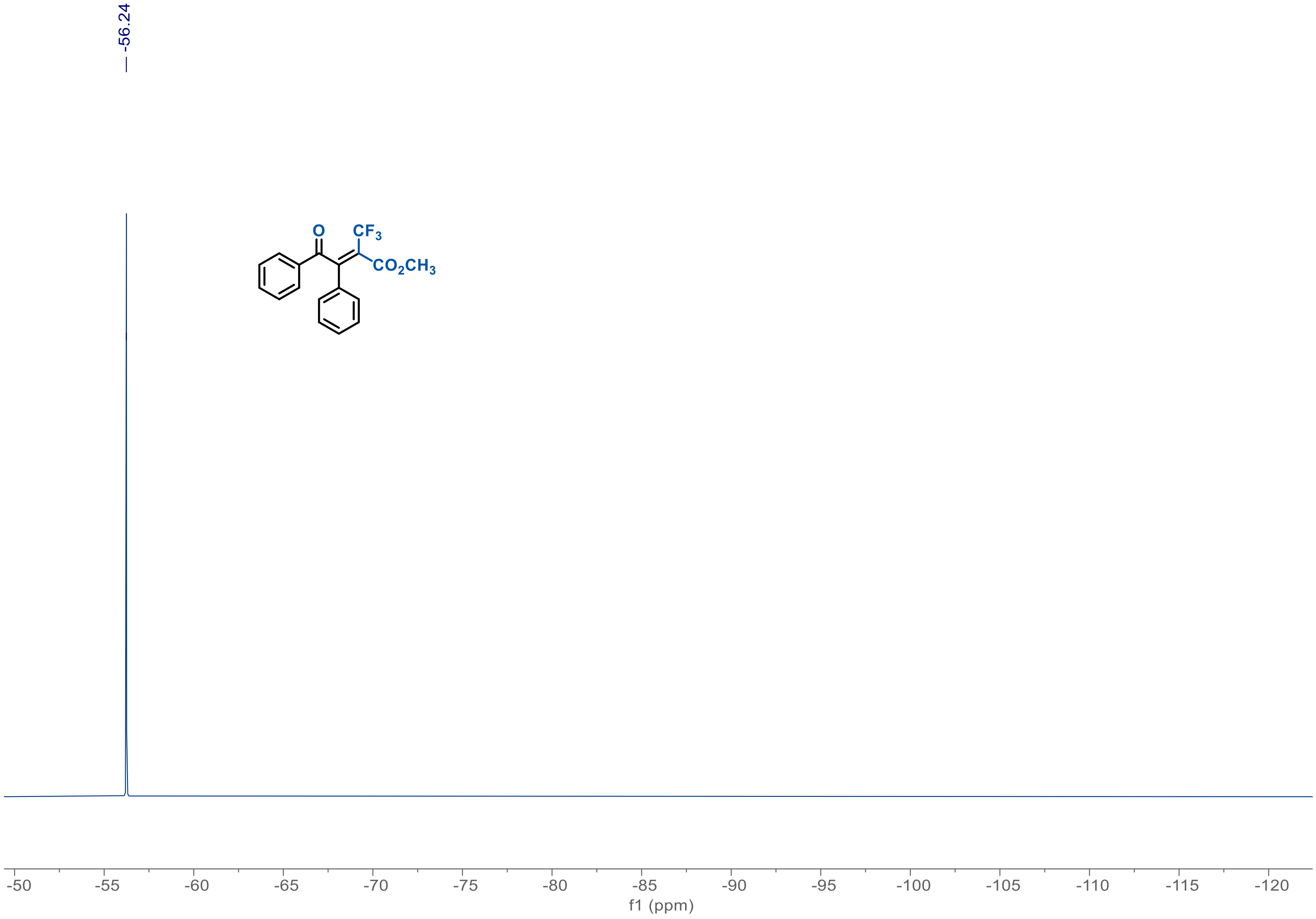

^{1}H NMR (300 MHz, $CDCl_3$) of **(E)-27**

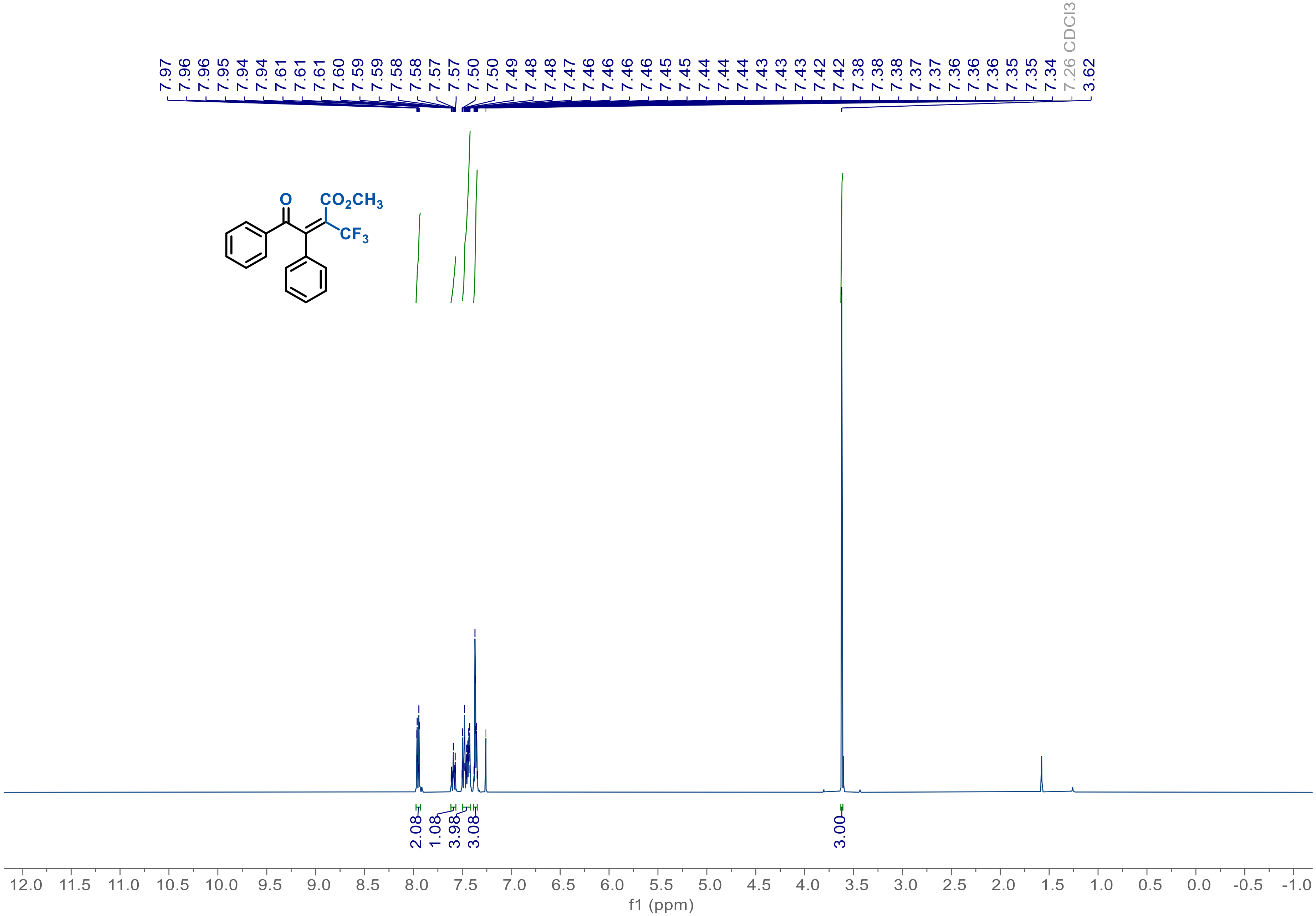

^{13}C NMR (101 MHz, $CDCl_3$) of **(E)-27**

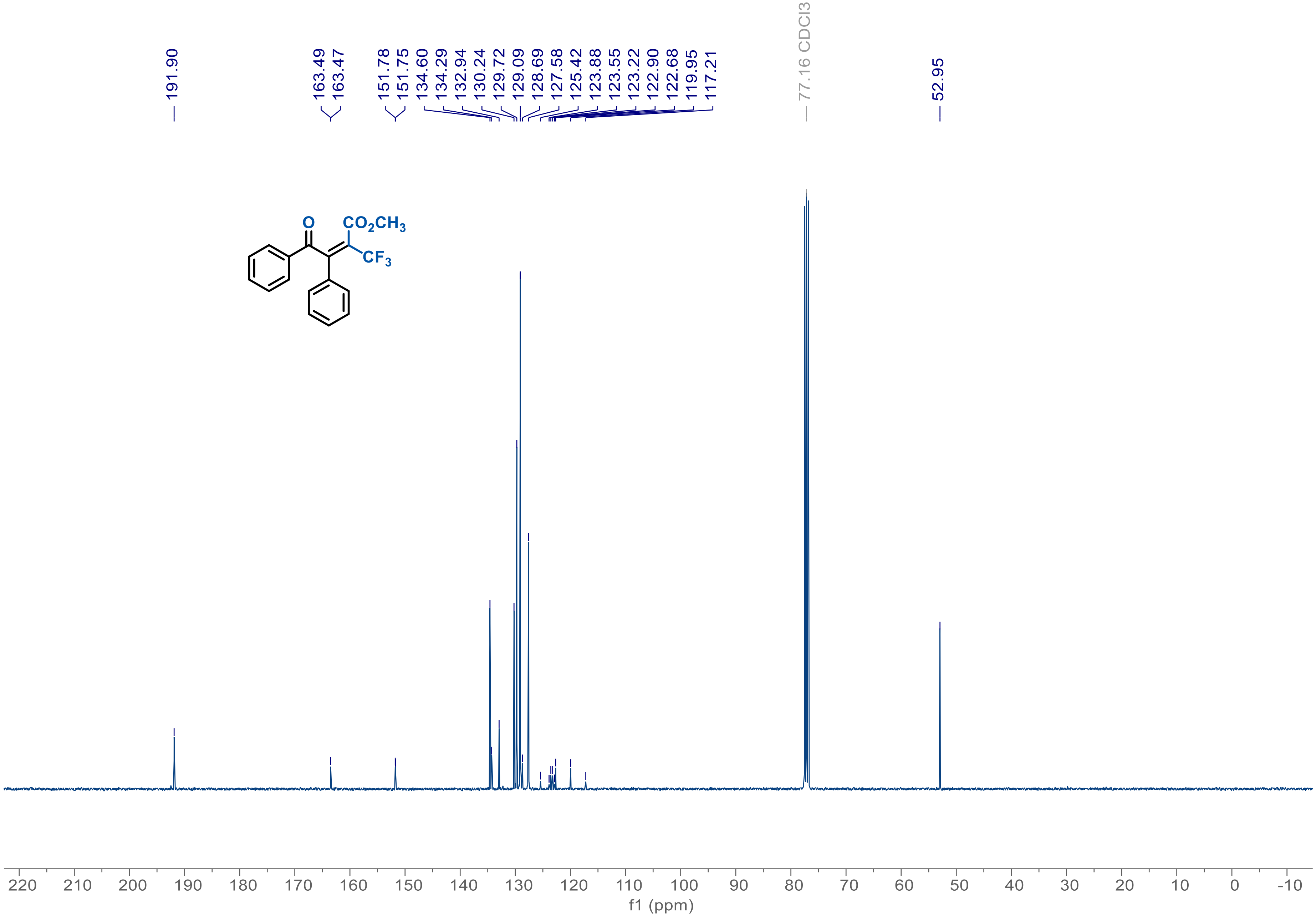

^{19}F NMR (282 MHz, CDCl$_3$) of **(E)-27**

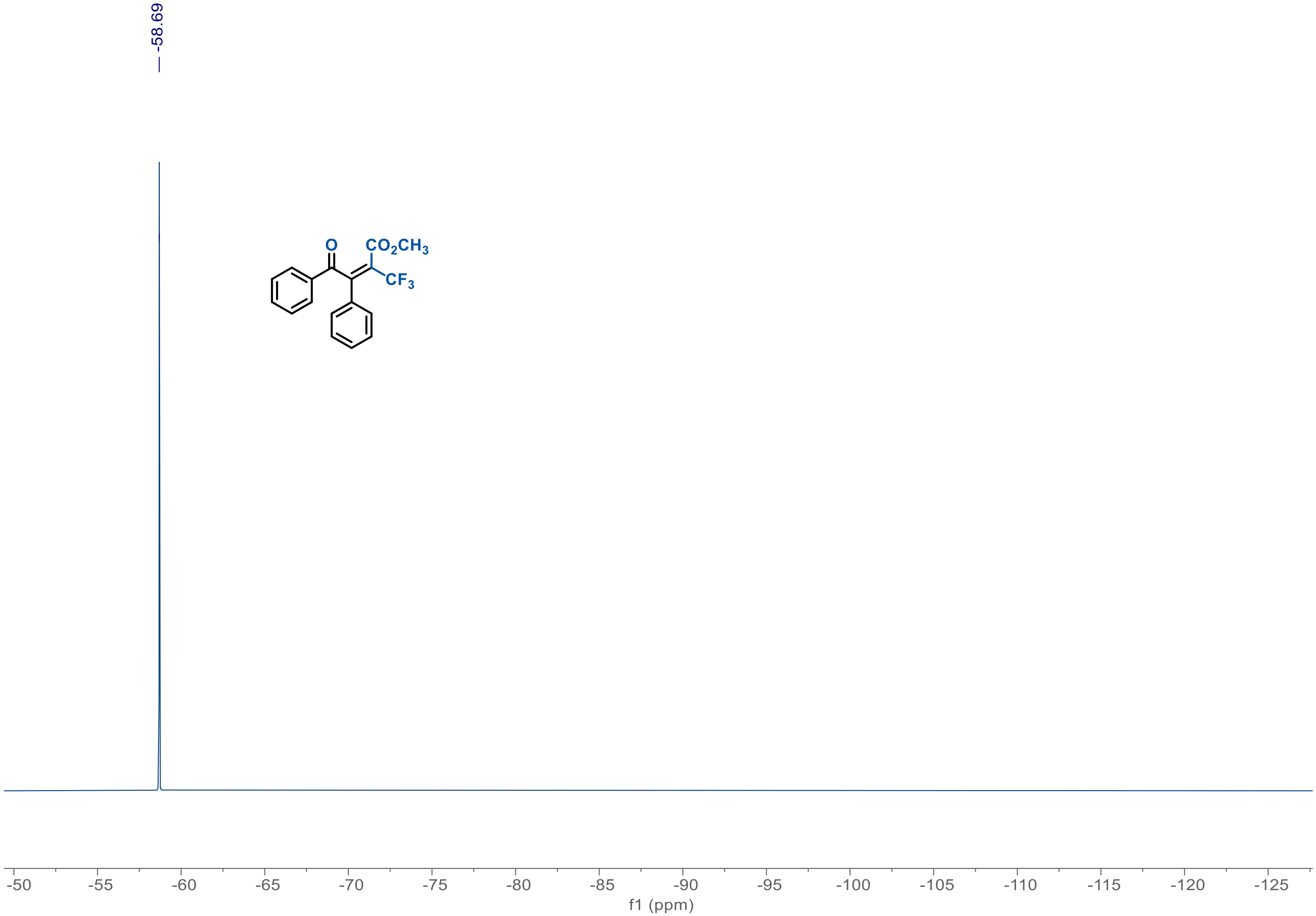

^{1}H NMR (300 MHz, $CDCl_3$) of **28**

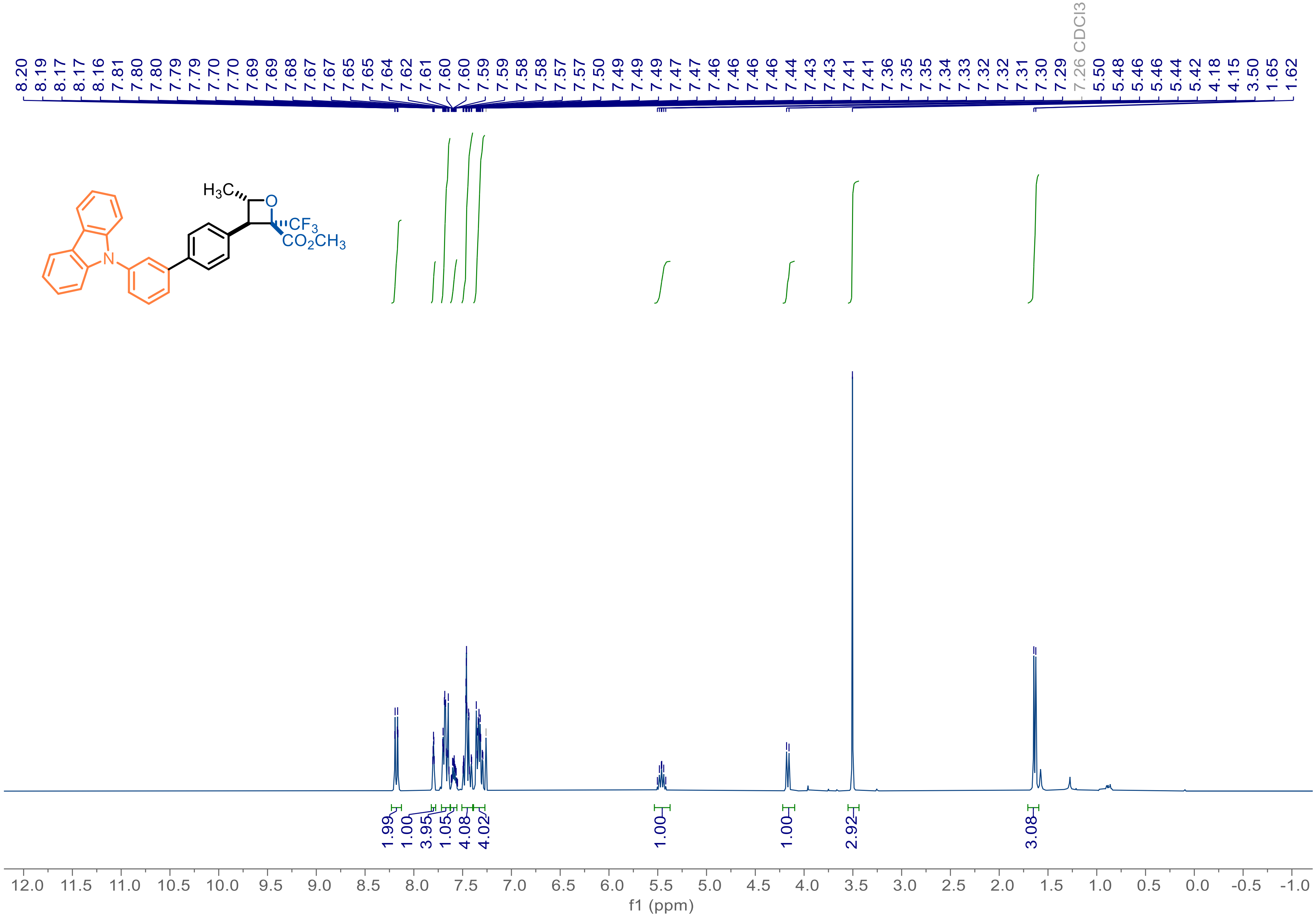

^{13}C NMR (75 MHz, $CDCl_3$) of **28**

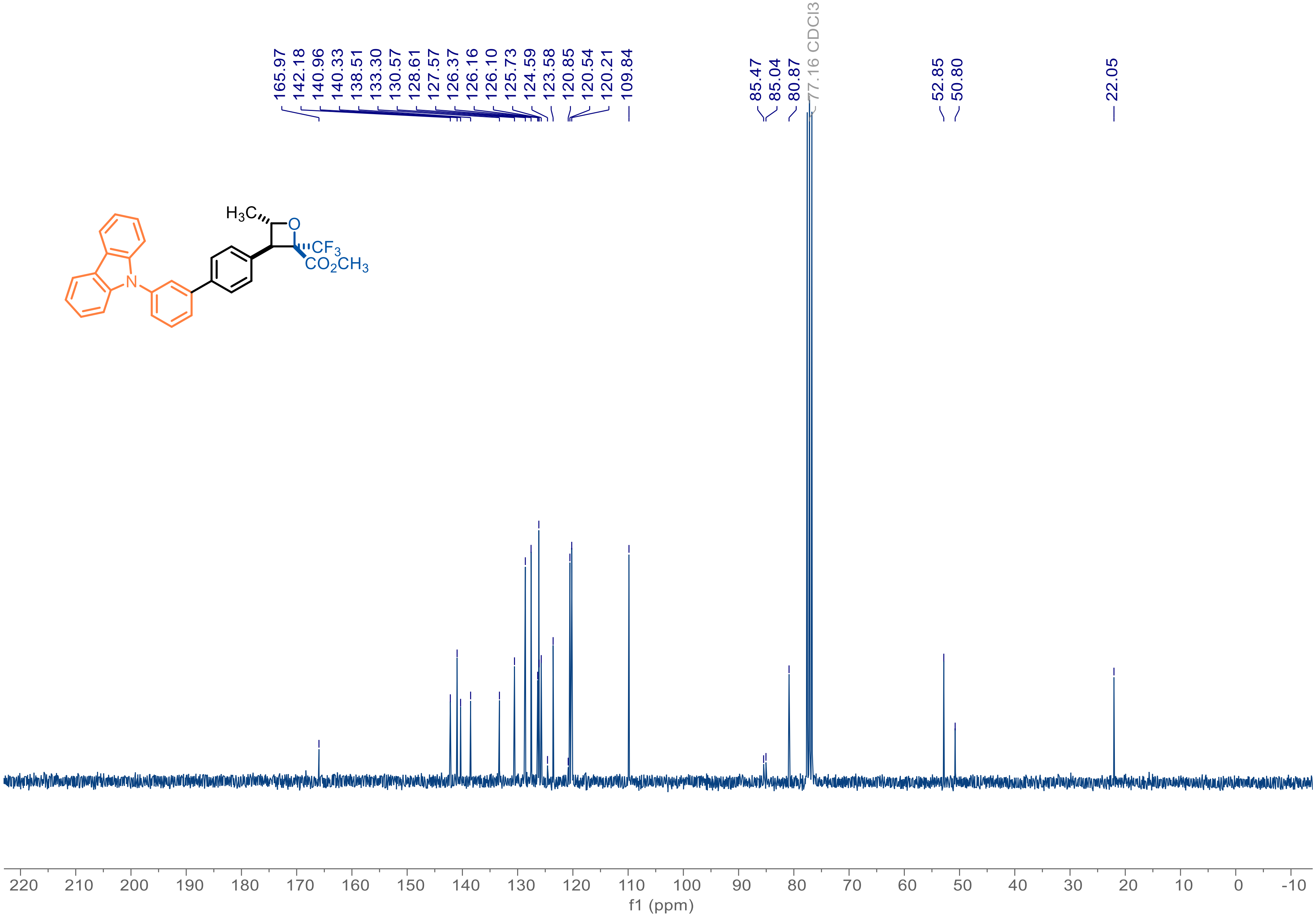

^{19}F NMR (282 MHz, $CDCl_3$) of **28**

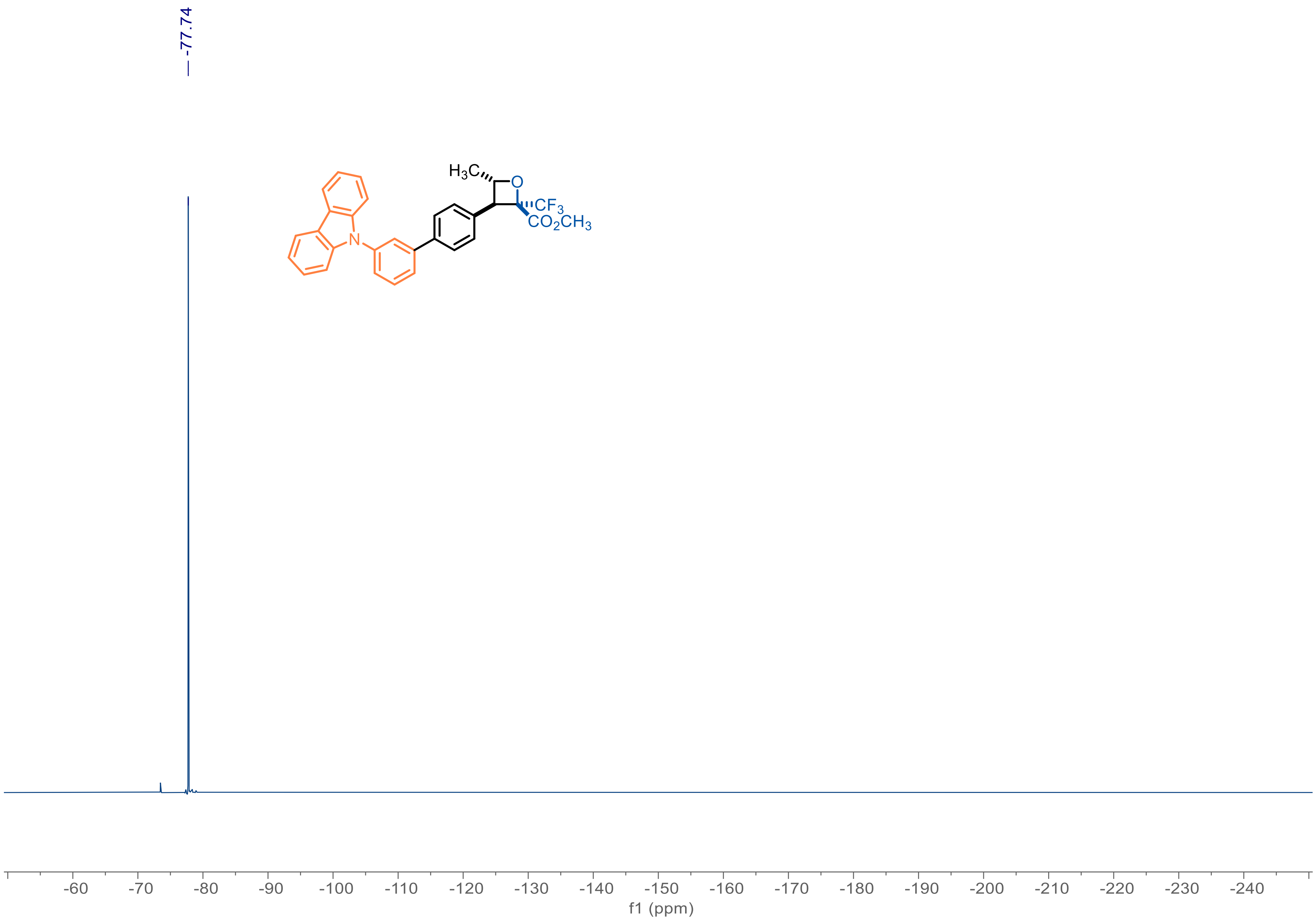

^{1}H NMR (300 MHz, CDCl3) of **29**

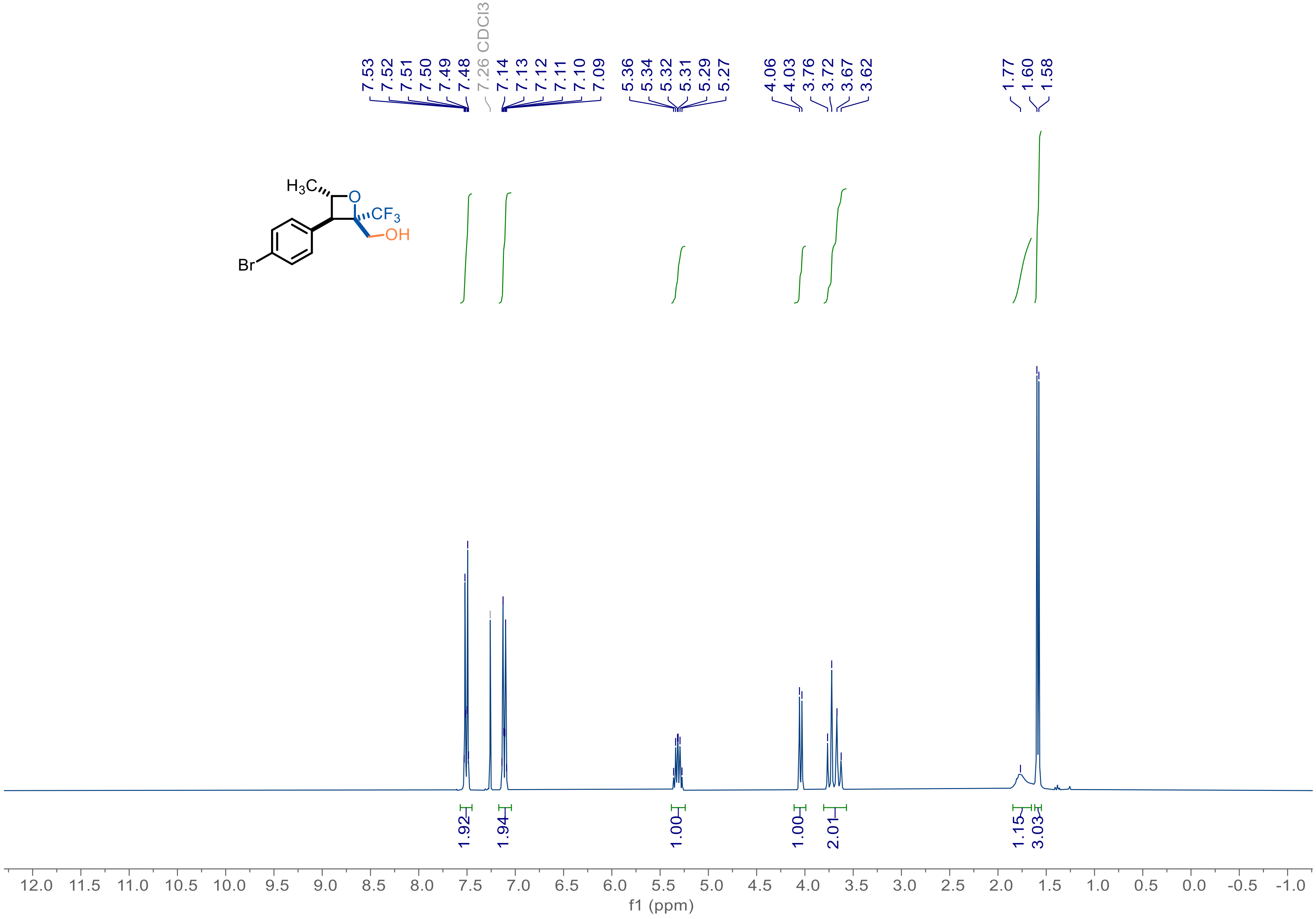

^{13}C NMR (75 MHz, $CDCl_3$) of **29**

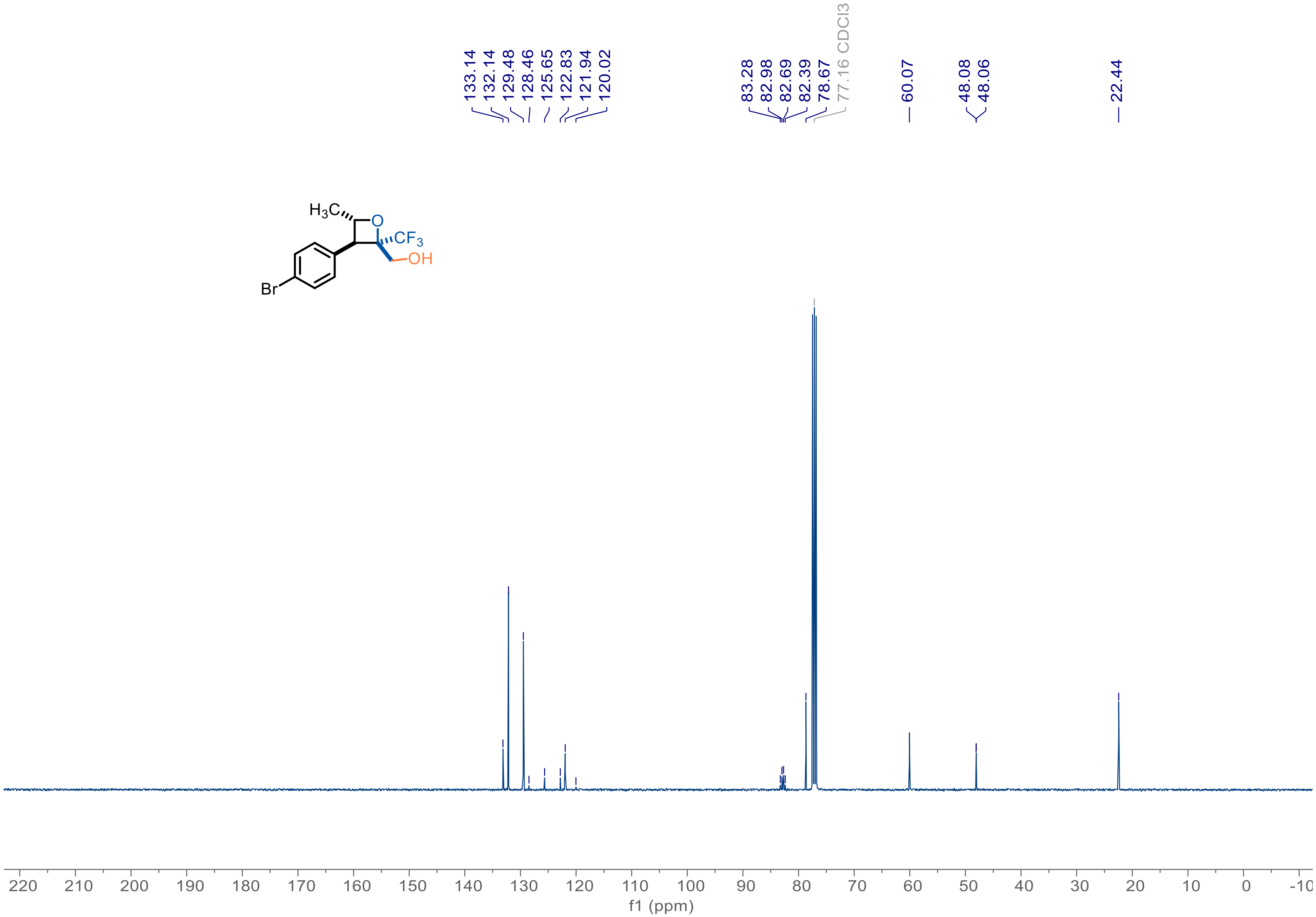

¹⁹F NMR (282 MHz, $CDCl_3$) of **29**

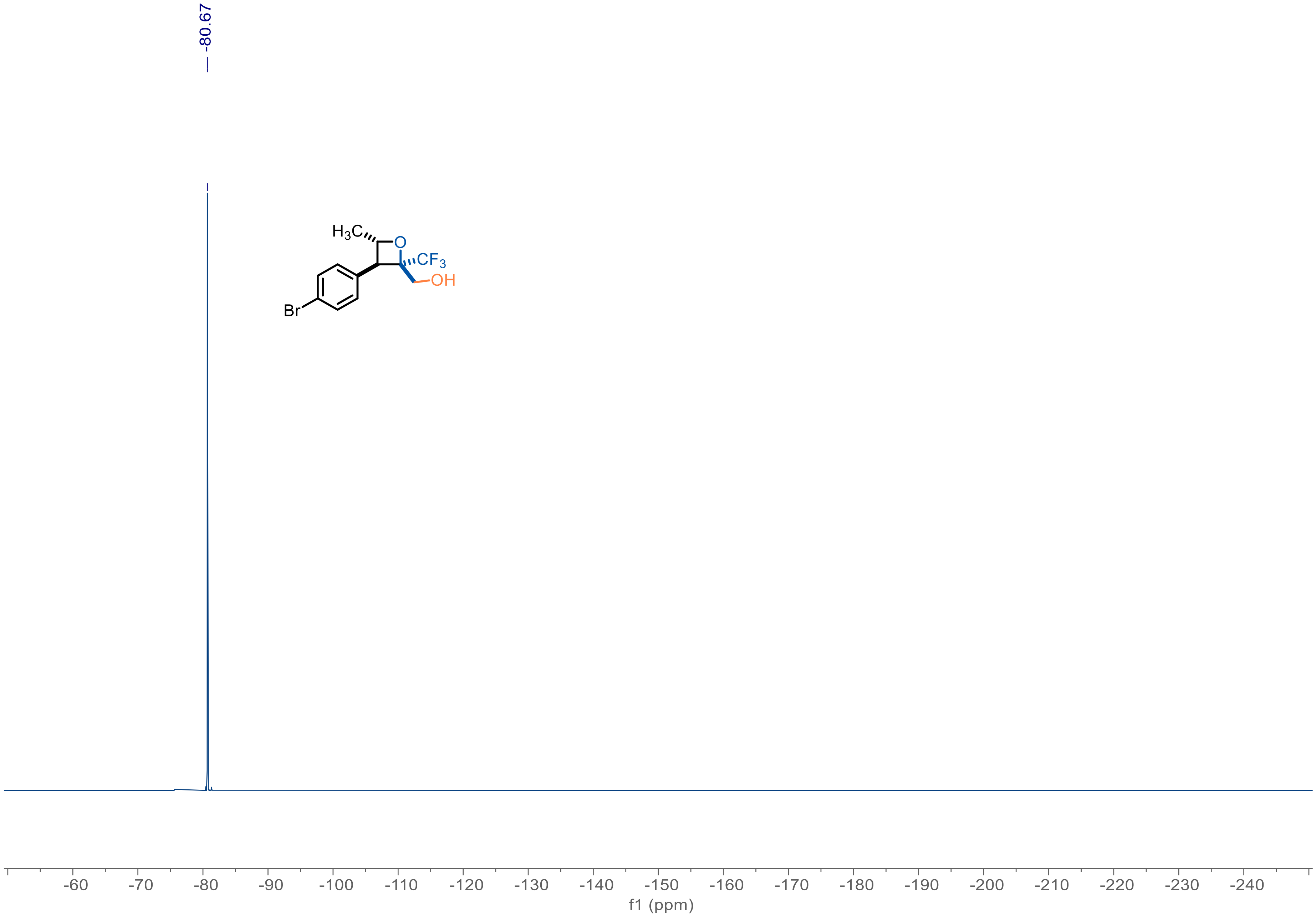

11. References

(1) K. Zhang, B. Jelier, A. Passera, G. Jeschke, D. Katayev, *Chem. – Eur. J.* 2019, **25**, 12929–12939.

(2) S. Lim, M. Ji, X. Wang, X. C. Lee, H.-Y. Jang, *Eur. J. Org. Chem.* 2015, **2015** 591–595.

(3) X. Huang, B. Fulton, K. White, A. Bugarin, *Org. Lett.* 2015, **17**, 2594–2597.

(4) B. Maji, S. Vedachalan, X. Ge, S. Cai, X.-W. Liu, *J. Org. Chem.* 2011, **76**, 3016–3023.

(5) T. Matsuda, K. Suzuki, S. Abe, H. Kirikae, N. Okada, *Tetrahedron* 2015, **71**, 9264–9270.

(6) P. Theodosis-Nobelos, M. Kourti, P. Tziona, P. N. Kourounakis, E. A. Rekka, *Bioorg. Med. Chem. Lett.* 2015, **25**, 5028–5031.

(7) K. Singh, S. J. Staig, J. D. Weaver, *J. Am. Chem. Soc.* 2014, **136**, 5275–5278.

(8) N. G. Connelly, W. E. Geiger, *Chem. Rev.* **1996**, *96*, 877–910.

(9) M. S. Lowry, J. I. Goldsmith, J. D. Slinker, R. Rohl, R. A. Pascal, G. G. Malliaras, S. Bernhard, *Chem. Mater.* 2005, **17** (23), 5712–5719

(10) M. J. Frisch, G. W. Trucks, H. B. Schlegel, G. E. Scuseria, M. A. Robb, J. R. Cheeseman, G. Scalmani, V. Barone, B. Mennucci, G. A. Petersson, H. Nakatsuji, M. Caricato, X. Li, H. P. Hratchian, A. F. Izmaylov, J. Bloino, G. Zheng, J. L. Sonnenberg, M. Hada, M. Ehara, K. Toyota, R. Fukuda, J. Hasegawa, M. Ishida, T. Nakajima, Y. Honda, O. Kitao, H. Nakai, T. Vreven, J. A. Montgomery, Jr., J. E. Peralta, F. Ogliaro, M. Bearpark, J. J. Heyd, E. Brothers, K. N. Kudin, V. N. Staroverov, R. Kobayashi, J. Normand, K. Raghavachari, A. Rendell, J. C. Burant, S. S. Iyengar, J. Tomasi, M. Cossi, N. Rega, J. M. Millam, M. Klene, J. E. Knox, J. B. Cross, V. Bakken, C. Adamo, J. Jaramillo, R. Gomperts, R. E. Stratmann, O. Yazyev, A. J. Austin, R. Cammi, C. Pomelli, J. W. Ochterski, R. L. Martin, K. Morokuma, V. G. Zakrzewski, G. A. Voth, P. Salvador, J. J. Dannenberg, S. Dapprich, A. D. Daniels, Ö. Farkas, J. B. Foresman, J. V. Ortiz, J. Cioslowski, D. J. Fox, Gaussian 9 Rev. D.01. 2013, *Wallingford, CT.*

(11) P. Pracht, F. Bohle, S. Grimme, S. *Phys. Chem. Chem. Phys.* 2020, **22** , 7169–7192.

(12) J.-D. Chai, M. Head-Gordon, *Phys. Chem. Chem. Phys.* 2008, **10**, 6615–6620.

(13) F. Weigend, R. Ahlrichs, *Phys. Chem. Chem. Phys.* 2005, *7*, 3297–3305.

(14) D. Rappoport, F. Furche, *J. Chem. Phys.* 2010, **133**, 134105.

(15) E. Cancès, B. Mennucci, J. Tomasi, *J. Chem. Phys.* 1997, **107**, 3032–3041.

(16) A. V. Marenich, C. J. Cramer, D. G. Truhlar, *J. Phys. Chem. B* 2009, **113**, 6378–6396.

(17) A. Tena Meza, C. A. Rivera, H. Shao, A. V. Kelleghan, K. N. Houk, N. K. Garg, *Nature* 2025, **640**, 683–690.

(18) *CYLview20; Legault, C. Y., Université de Sherbrooke, 2020. http://www.cylview.org/ (accessed 2025-12-03)*
